# Hydrogen Bonding and Vaporization Thermodynamics in Hexafluoroisopropanol‐Acetone and ‐Methanol Mixtures. A Joined Cluster Analysis and Molecular Dynamic Study

**DOI:** 10.1002/cphc.202100620

**Published:** 2021-11-11

**Authors:** Gwydyon Marchelli, Johannes Ingenmey, Oldamur Hollóczki, Alain Chaumont, Barbara Kirchner

**Affiliations:** ^1^ Mulliken Center for Theoretical Chemistry Rheinische Friedrich-Wilhelms-Universität Bonn Beringstr. 4+6 D-53115 Bonn Germany; ^2^ Université de Strasbourg CNRS, CMC UMR 7140 Laboratoire MSM F-67000 Strasbourg France

**Keywords:** enthalpy of vaporization, hexafluoroisopropanol, mixtures, molecular dynamics simulations, quantum cluster equilibrium

## Abstract

Binary mixtures of hexafluoroisopropanol with either methanol or acetone are analyzed via classical molecular dynamics simulations and quantum cluster equilibrium calculations. In particular, their populations and thermodynamic properties are investigated with the binary quantum cluster equilibrium method, using our in‐house code peacemaker 2.8, upgraded with temperature‐dependent parameters. A novel approach, where the final density from classical molecular dynamics, has been used to generate the necessary reference isobars. The hydrogen bond network in both type of mixtures at molar fraction of hexafluoroisopropanol of 0.2, 0.5, and 0.8 respectively is investigated via the molecular dynamics trajectories and the cluster results. In particular, the populations show that mixed clusters are preferred in both systems even at 0.2 molar fractions of hexafluoroisopropanol. Enthalpies and entropies of vaporization are calculated for the neat and mixed systems and found to be in good agreement with experimental values.

## Graphical abstract

**The thermodynamics properties** and the hydrogen bond networks in hexafluoroisopropanol's binary mixtures with methanol or acetone are investigated with a combination of molecular dynamics simulations and cluster weighting. Even at low concentration of hexafluoroisopropanol mixed clusters are found to be preferred, indicating two deeply interacting mixtures.

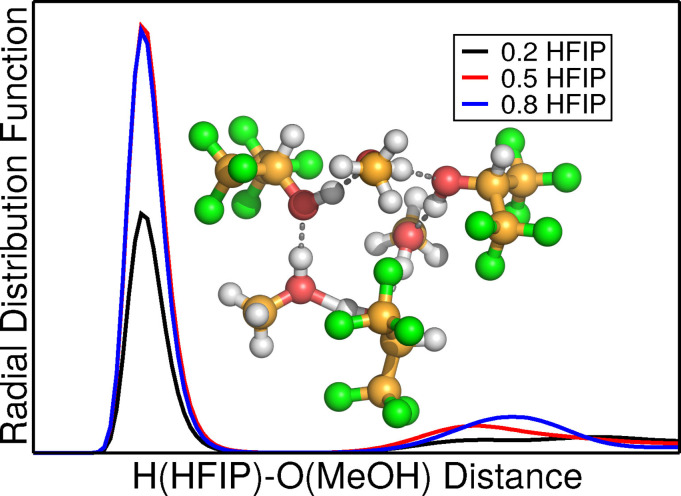

## 1 Introduction

The investigation of the mechanisms and interactions that regulate both neat fluids as well as their mixtures plays a fundamental role in the design of novel solvents. Over the last decades the interest in developing sustainable chemical processes has grown, and with it the need for new solvents that follow the fundamentals of green chemistry.^[1]^ For example liquid‐liquid – i. e. solvent – metal extraction offers a number of advantages compared to other techniques,^[2]^ but the employed solvents are often toxic and expensive to dispose of. The last few years have seen an increasing demand of novel sustainable solvents for metal ions extraction.^[3]^ Another approach is the use of the so‐called deep eutectic solvents (DES), which are low‐melting eutectics formed by the mixture of two or more substances whose eutectic point temperature is much lower than that of an ideal mixture.[^4^, ^5^] With properties similar to those of ionic liquids, such as a low vapor pressure, low melting point, and high thermal stability,[^6^, ^7^] they have come to be known as an economic and eco‐friendly alternative for conventional organic solvents. A new kind of DESs, called Type V DESs, has recently been introduced.^[8]^ It is formed by the combination of two non‐ionic moieties which establish a strong hydrogen bond network, and has been proven to be more sustainable than traditional organic solvents.^[8]^ In recent works, hydrophobic DES formed by menthol with different organic acids have been proved to present a strong hydrogen bond between the two components, stronger than the hydrogen bond network in the neat system.[^9^, ^10^] Different hydrogen bond donors and acceptors have been investigated as components of hydrophobic or Type V DESs,^[8]^ such as decanoic acid and lidocaine,^[11]^ menthol with different natural acids,^[12]^ and 1,1,1,3,3,3‐hexafluoroisopropanol (HFIP) with betaine and L‐carnitine.^[13]^ HFIP in particular is an extraordinary solvent used in many different applications, including the activation of organic functionalities such as the intramolecular Schmidt reaction using a Lewis acid in HFIP,^[14]^ the activation of carbonyl and epoxide substrates,[^15^, ^16^] or the activation of hydrogen peroxide in the Baeyer−Villiger oxidation reaction.[^17^, ^18^] It can also serve as a proton donor in dihydrogen bonding with different transition metal hydrides, as non‐classical hydrogen bond.[^15^, ^19^] Its widespread use is due to a number of beneficial properties, such as its thermal stability, transparency to UV radiation, as well as its remarkable solvent properties, allowing it to dissolve a wide range of polymers, as well as most common polar and non‐polar solutes.[^20^, ^21^] In aqueous solutions, its acidity range is comparable to the one of formic acid.^[15]^ Furthermore, its low boiling point facilitates its recovery via distillation. However computational studies on HFIP remain sparse. For instance HFIP has been investigated with molecular dynamics by some of the present authors to prove its catalytic effect on C,C coupling reactions on aromatic compounds with positive results.^[16]^ In 2019, Deng *et al*. were the first to study HFIP‐based DESs as the non‐polar phase in liquid‐liquid micro‐extractions.^[13]^ To be able to understand the mechanisms behind the formation of novel mixtures it is imperative to study their structure and in particular the hydrogen bond network which may exist in these liquids. From an experimental prospective, hydrogen bonds can be investigated via IR and NMR.[^22^, ^23^] However, since their experimental detection and analysis can still be challenging and expensive, computational tools may be valuable for this kind of investigation.[^24^, ^25^, ^26^] We were able to calculate the activity coefficients of methanol in its binary mixtures with a set of small alcohols, which allowed some insight into how the chain length and branching modify the hydrogen bond network and consequently affect their behavior.^[27]^ Since it is fundamental to study the hydrogen bond network in binary organic solvents, it is interesting to investigate the behavior of HFIP with both an hydrogen bond acceptor (acetone) and a compound able to work both as hydrogen bond acceptor and donor (methanol). The binary mixtures of HFIP with acetone or methanol have already been investigated experimentally in the past, but so far their liquid structure has never been investigated in detail.[^16^, ^28^] In the current article, we investigate the inter‐molecular interactions present in the binary mixtures of HFIP with either solvents. The study is based on a combined computational procedure of both classical molecular dynamics simulations (MD) and quantum chemical calculations. Furthermore, we use the binary quantum cluster equilibrium (bQCE) method to investigate the distribution of inter‐molecular interaction motifs in the system. The method assigns populations to a set of clusters, enabling the weighting of properties according to the cluster distribution similar to Boltzmann weighting. In contrast to it, however, the bQCE method can weight clusters of different sizes and compositions and considers not only their electronic energies but also the particle volume and inter‐cluster interactions. Boltzmann and bQCE weighting were compared in the past by our group in the field of the computational calculation of vibrational circular dichroism spectra of bulk phase, showing the advantages of our method over Boltzmann weighting.^[29]^ For this purpose we use our in‐house code peacemaker 2.8, which has proven to be a valuable tool to investigate the thermodynamic properties of both neat and mixed systems.[^27^, ^30^, ^31^, ^32^, ^33^, ^34^, ^35^, ^36^, ^37^, ^38^] The standard bQCE method involves optimization of two empirical parameters by fitting them to a set of experimental data such as boiling points or isobars; however, even for simple mixtures of organic solvents, these data are often unavailable in the literature. Hence we follow a different approach, namely, the isobars are instead obtained via MD simulations. In the following we will first outline and discuss the computational methods used, after which we will present and investigate the results we have obtained.

## Computational details

### MD simulations

Initial configurations of the different systems were generated using the PACKMOL package (version 17.039).^[39]^ Molecules were randomly placed in a cell with an initial cell vector of 40–50 Å, depending on the composition of the system. MD simulations were performed using the LAMMPS program package (version 11 Aug 2017).^[40]^ OPLS‐AA force field parameters were used for methanol (MeOH) and acetone, whereas for HFIP we adopted the force field developed by Fioroni *et al*., which was optimized to reproduce the experimental density.^[41]^ A Lennard‐Jones 6–12 potential was used to describe van der Waals (vdW) interactions. Lorentz–Berthelot mixing rules were used to describe non‐bonded interactions.^[42]^ A cutoff of 1 nm was applied for vdW and Coulombic interactions. In the first step the SHAKE algorithm^[43]^ was used to constrain the bonds involving hydrogen atoms and followed by energy minimization. This process was repeated 3–5 times to let the systems mix correctly and eliminate energetic hot spots in order to stabilize the systems. Afterwards, the boxes were deformed to reach a preliminary and fixed cell volume, calculated to reflect a density of 0.8 g/cm^3^ for each system. Following this, the systems were simulated for 1.5 ns in the *NpT* ensemble, using Nosé–Hover thermostat and barostat, to let the volume converge. The cell volumes over the last 0.5 ns were found to remain constant, as can be seen in Figure S2 in the SI. The system volume was then set to the average volume taken over the last 0.5 ns in the *NpT* ensemble. A further 1 ns of simulation time in the *NVT* ensemble was performed to further equilibrate the system. Finally, a production run of 20 ns was performed in the *NVT* ensemble using the same conditions as during equilibration. The time step was set to 0.5 fs for the pre‐equilibration processes (shake, minimization, and deformation of the box) and increased to 1 fs afterwards. The simulations were analyzed with our in‐house trajectory analysis code travis.[^44^, ^45^] The angular distribution functions (ADFs) were calculated using the cone correction included in travis with a cut off of 250 pm as maximum distance between the reference and observed molecules.^[46]^ Along with the Radial Distribution Functions (RDFs), the coordination numbers (CN) are calculated as well $\left(CN=\rho_{bulk}\int g\left(r\right)r^{2}dr\right)$ , where *g*(*r*) is the RDF's intensity and *ρ_bulk_* is the bulk density of the system. Please note that, since the RDF is strictly dependent on the number of molecules that respect the given condition, the intensity of the systems cannot be compared. The peak's position, however, is not dependent on the number of molecules and they can be compared. Hydrogen bond lifetimes are analyzed using the dimer existence auto correlation function (DACF) implemented in the travis code with the default curve fitting and approximations included in the code and described in literature.^[47]^ For this analysis, the distance condition of 350 pm for the O−O distance and an angular condition of 135°–180° for the O−H−O angle were applied.

### Cluster generation

The construction of a cluster set that is representative of the investigated system is a crucial step in the bQCE procedure and has been discussed in previous works.[^27^, ^32^, ^38^, ^48^, ^49^] As a first step, to find clusters, we performed a global minimum structure search for each cluster size and composition by running the genetic optimization algorithm OGOLEM[^50^, ^51^] at the force field level of theory, using the AMBER 2016 program package^[52]^ and the GAFF force field^[53]^ implemented therein. The AMBER/OGOLEM combination is optimized to screen a great number of individual clusters.[^27^, ^32^, ^38^] The number of individuals per generation was varied between 80 and 240 in accordance to the cluster size to adjust for the increasing complexity. In total, a number of 2000–6000 individuals, i. e. clusters, were generated and evaluated for every possible composition up to a cluster size of six molecules. As the search for the global minimum structure by a genetic algorithm is performed on the classical force field level and the enormous configuration space poses a great challenge, the obtained structures are not necessarily identical to the global minimum on the quantum chemical level of theory. Instead, they can be understood as good candidate solutions to the global minimum and generally represent stable clusters that cover a range of enthalpically or entropically favored configurations. In addition, many of the obtained structures are expected to collapse towards the same geometry during quantum chemical optimization. Therefore, we select a number of ten clusters evenly distributed in the energetic range of the final generation for subsequent quantum chemical optimization for each cluster size. These clusters were then optimized at the BP86/def2‐TVZP^[54]^ level of theory with Turbomole (version 7.41) and an energy convergence threshold of 10^–9^.^[55]^ The BP86 functional is proven to deliver good structural results and, if combined with a triple‐*ζ* basis set, to reproduce with good accuracy the measured vibrational fundamentals.[^56^, ^57^] The London dispersion energy was taken into account by applying the D3 dispersion correction.[^58^, ^59^] Frequency calculations were performed for all clusters and those with imaginary frequencies were excluded. To avoid duplicate clusters in the cluster set, the structural similarity of all optimized clusters was quantified by their geometrical distance^[60]^ *d*:

(1) $$d\left(𝒫,{𝒫}^{'}\right)={\left[{\left(\frac{I_{A}-I_{A^{'}}}{I_{A}}\right)}^{2}+{\left(\frac{I_{B}-I_{B^{'}}}{I_{B}}\right)}^{2}+{\left(\frac{I_{C}-I_{C^{'}}}{I_{C}}\right)}^{2}\right]}^{\frac{1}{2}},$$

wherein *I* and $I^{'}$ are the principal moments of inertia of the clusters 𝒫 and ${𝒫}^{'}$ , respectively. Clusters ${𝒫}^{'}$ with a geometrical distance of $d\left(𝒫,{𝒫}^{'}\right)<0.01$ were removed from the cluster set. At the end of this procedure, 10 clusters of neat HFIP, 11 of neat acetone, 13 of neat methanol, 45 of the mixed HFIP–acetone system and 73 of HFIP–methanol were ready to be analyzed and to be included as inputs for the further bQCE calculations. Their interaction energy, size, and composition are tabulated in the supporting information. In the following, clusters will be given unique labels of the form hXsY–Z, where h stands for HFIP, s can be either acetone (a) or methanol (m), X is the number of monomers of HFIP, Y is the number of monomers of s, and Z is a label to differentiate clusters of the same composition.

### The bQCE method

The theory of bQCE methods has been extensively detailed in several earlier works.[^35^, ^38^, ^49^, ^61^] Through this method, we are able to calculate the cluster distribution and the thermodynamic properties of the system (for instance vaporization enthalpies) for a selected temperature range. Here, only a short overview of the bQCE method will be presented. As a first step, a system of non‐interacting clusters in thermodynamic equilibrium is considered. Each cluster in that system is built up from either one (neat systems) or two (binary systems) monomers.

In thermodynamic equilibrium these clusters can transform into each other. We can write the equilibrium reaction between the clusters as

(2) $$i\left(𝒫\right)C_{1}+j\left(𝒫\right)C_{2}\overset{⇀}{↽}C_{𝒫},$$

where $i\left(𝒫\right)$ and $j\left(𝒫\right)$ are the number of monomers of each component $C_{1}$ and $C_{2}$ that form the cluster 𝒫 . The system's total partition function $Q^{\mathrm{tot}}$ at volume *V* and temperature *T* is given by

(3) $$Q^{\mathrm{tot}}\left(\left\{N_{𝒫}\right\},V,T\right)=\prod_{𝒫=1}^{N}\frac{1}{N_{𝒫}!}{\left[q_{𝒫}^{\mathrm{tot}}\left(V,T\right)\right]}^{N_{𝒫}},$$

where $q_{𝒫}^{\mathrm{tot}}$ is the partition function of the single cluster 𝒫 and $\left\{N_{𝒫}\right\}$ is the full set of total cluster populations $N_{𝒫}$ . This cluster partition function can be evaluated as product of partition functions corresponding to the different degrees of freedom:

(4) $$q_{𝒫}^{\mathrm{tot}}\left(V,T\right)=q_{𝒫}^{\mathrm{trans}}\left(V,T\right)q_{𝒫}^{\mathrm{rot}}\left(T\right)q_{𝒫}^{\mathrm{vib}}\left(T\right)q_{𝒫}^{\mathrm{elec}}\left(T\right).$$

Here, $q_{𝒫}^{\mathrm{trans}}$ is the translational, $q_{𝒫}^{\mathrm{rot}}$ the rotational, and $q_{𝒫}^{\mathrm{vib}}$ the vibrational partition function. They are calculated from standard equations for the particle in a box, rigid rotator, and harmonic oscillator, respectively.[^61^, ^62^] The electronic partition function $q_{𝒫}^{\mathrm{elec}}$ is calculated from the adiabatic binding energy $\Delta_{\mathrm{bind}}E_{𝒫}^{\mathrm{elec}}$ of the cluster.^[63]^

Until here, we considered our system to consist of non‐interacting clusters. However, in order to describe a liquid, not only the binding energy within a cluster but also the inter‐cluster interactions must be considered. First, in order to take the volume into account that is taken up by the clusters themselves and is inaccessible to translation, an exclusion volume *V*_ex_ must be subtracted from the phase volume *V* in $q_{𝒫}^{\mathrm{trans}}$ . The exclusion volume is calculated as

(5) $$V_{\mathrm{ex}}=b_{\mathrm{xv}}\sum_{𝒫=1}^{N}N_{𝒫}v_{𝒫},$$

wherein *b*_xv_ is the empirical exclusion volume parameter to correctly scale the cluster volume $v_{𝒫}$ , which is sensitive to the choice of the volume method and the atomic radii used therein. In previous works, *b*_xv_ was treated as temperature independent.[^27^, ^32^, ^33^, ^35^, ^38^] Here, we introduce a linear temperature dependence of *b*_xv_

(6) $$b_{\mathrm{xv}}\left(T\right)=T\cdot \beta_{\mathrm{xv}}+b_{\mathrm{xv}}^{0},$$

where $\beta_{\mathrm{xv}}$ is the exclusion volume expansion coefficient and $b_{\mathrm{xv}}^{0}$ is the base of the intercept. A similar approach was used in the past by Kelterer and coworkers.^[64]^

Finally, the inter‐cluster interactions must be taken into account. The electronic partition function $q_{𝒫}^{\mathrm{elec}}$ is extended by a volume dependent mean‐field‐like correction term:

(7) $$q_{𝒫}^{\mathrm{elec}}\left(V,T\right)=\exp\left\{-\frac{\Delta_{\mathrm{int}}E_{𝒫}^{\mathrm{elec}}-\left[i\left(𝒫\right)+j\left(𝒫\right)\right]\frac{a_{\mathrm{mf}}}{V}}{k_{B}T}\right\},$$

where *k*_B_ is the Boltzmann constant and the mean‐field parameter *a*_mf_ is an empirical parameter, that scales the strength of the inter‐cluster interactions.

When performing a bQCE calculation, the empirical parameters $b_{\mathrm{xv}}^{0}$ , $\beta_{\mathrm{xv}}$ , and *a*_mf_ are chosen such that the deviation from a given reference property, such as densities and phase transition temperatures, is minimized. In earlier works, a simple grid sampling algorithm was used to optimize the empirical parameters. With the introduction of a third parameter $\beta_{\mathrm{xv}}$ this method is no longer feasible. Here, the Differential Evolution algorithm^[65]^ as implemented in the SciPy library^[66]^ for python 3.4 is interfaced with the peacemaker 2.8 code to find the best solution. The script is available upon request.

## 2 Results and Discussion

Here, we will discuss the results of our investigation of the binary mixtures of HFIP with acetone and methanol, respectively. These were obtained by employing the bQCE method to a set of quantum chemically optimized clusters depicting different binding motifs, that are representative for the specific interactions in each system. Instead of employing experimental data, in this work, we use isobars obtained from a set of classical molecular dynamics simulations of the mixed systems that we conducted at different temperatures and mixture compositions as detailed in the computational details section.

### 2.1 Classical Molecular Dynamics Simulations

Classical molecular dynamics simulations of both mixed systems HFIP/acetone and HFIP/methanol were carried out at defined temperatures ranging from 298.15–338.15 K in intervals of 10 K. These simulations were repeated at different compositions with mole fractions of HFIP of 0.2, 0.5, and 0.8. The results are listed in Table 1 for HFIP/methanol (top) and HFIP/acetone (bottom). Both binary mixtures show similar behavior. Due to the high density of HFIP, the density increases with the mole fraction of HFIP but decreases with rising temperature. In addition, Table 1 lists the experimental densities of the HFIP/acetone mixture at 298.15 K, which were measured by Evans *et al*. using a single neck capillary tube pycnometer.^[28]^ No experimental density could be found for the system HFIP/methanol. Our calculated densities for the system HFIP/acetone deviate by 2.5 %, 10.5 %, and 10.8 % from the experimentally measured values at mole fractions 0.2, 0.5, and 0.8, respectively. This deviation can have an impact on the thermodynamic properties of mixing calculated via the bQCE approach, however we demonstrate in the supporting information (SI) that a deviation in this range only slightly affects the cluster population and thermodynamical properties such as entropies and enthalpies of vaporization. Approximated thermodynamic properties of mixing are included in the SI for sake of completeness.

**Table 1:** **Top**: Calculated densities $\rho^{\mathrm{calc}}$ of the HFIP/methanol mixture in g/cm^3^ at different temperatures and mole fractions. **Bottom**: Experimental^[28]^ densities $\rho^{\exp}$ and calculated densities $\rho^{\mathrm{calc}}$ of the HFIP/acetone mixture in g/cm^3^ at different temperatures and mole fractions.

	**HFIP/MeOH**					
XHFIP	$\rho_{298}^{\exp}$	$\rho_{298}^{\mathrm{calc}}$	$\rho_{308}^{\mathrm{calc}}$	$\rho_{318}^{\mathrm{calc}}$	$\rho_{328}^{\mathrm{calc}}$	$\rho_{338}^{\mathrm{calc}}$
0.2	–	1.044	1.028	1.013	0.993	0.975
0.5	–	1.290	1.270	1.252	1.232	1.216
0.8	–	1.513	1.492	1.471	1.446	1.427
						
	**HFIP/Acetone**					
XHFIP	$\rho_{298}^{\exp}$	$\rho_{298}^{\mathrm{calc}}$	$\rho_{308}^{\mathrm{calc}}$	$\rho_{318}^{\mathrm{calc}}$	$\rho_{328}^{\mathrm{calc}}$	$\rho_{338}^{\mathrm{calc}}$
0.2	0.996	0.971	0.957	0.943	0.927	0.914
0.5	1.274	1.140	1.124	1.101	1.089	1.066
0.8	1.479	1.320	1.298	1.273	1.251	1.231

In order to characterize the hydrogen bonds present in the mixtures, additional analyses were carried out including Radial Distribution Functions (RDFs), Coordination Numbers (CNs), and Angle Distribution Functions (ADFs) of the simulated systems at 298.15 K averaged over 19.8 ns of production run.

**Figure 1:**
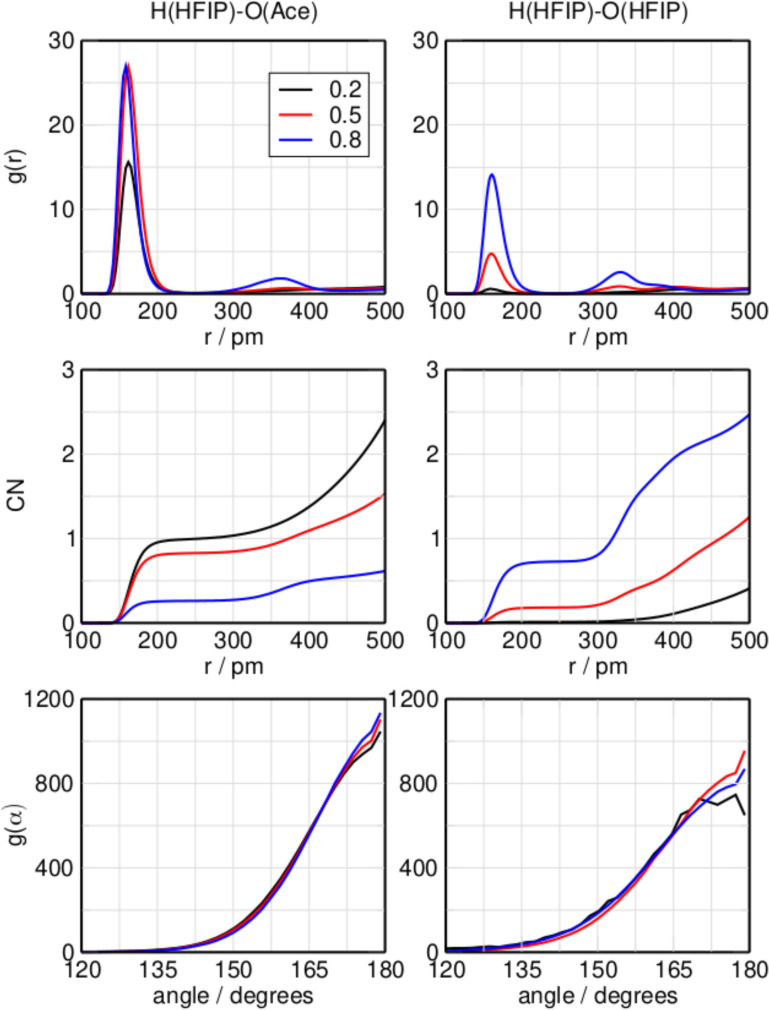
Radial distribution function, coordination number, and angular distribution function of the hydrogen bond for the system HFIP/acetone increasing the molar fraction of HFIP at 298.15 K with HFIP bond donor and acetone acceptor (left) or HFIP (right).

Figure 1 shows (from top to bottom) the RDF, the CN, and ADF of the O−H⋯O hydrogen bond between HFIP and acetone (left side) and between HFIP and HFIP (right side). Please note, HFIP is treated as the reference molecule. The observed molecule can be either acetone or another molecule of HFIP. The reference atom is the hydrogen of the hydroxy group, and the observed atom is the oxygen of the observed molecule. First, the inter‐species hydrogen bond between HFIP and acetone will be considered. A sharp peak is present in the RDF at 161 pm at mole fractions of 0.2 and 0.5. The bond length decreases to 159 pm at a mole fraction of 0.8. An additional peak emerges around 350 pm at high concentrations of HFIP. The coordination number (CN) displayed in Figure 1 provides insight into the average number of molecules participating in a hydrogen bond. A single hydrogen bond is possible between the two molecules. At a distance of 250 pm, which is the location of the first minimum in the RDF and can be considered the maximum hydrogen bond length, the CN is 1.0 for the mole fraction of 0.2, meaning all the possible hydrogen bonds are filled by this interaction. With an increasing mole fraction of HFIP of 0.5 and 0.8, the CN decreases to 0.83 and to 0.26, respectively. In the RDF of the same‐species hydrogen bond between two molecules of HFIP, a peak is present at 164 pm, which becomes sharper as the mole fraction of HFIP increases. At mole fractions of 0.5 and 0.8 an additional peak is visible at 326 pm, indicating an involved hydrogen bond network. From the CN plot, it is immediately clear that inter‐species interactions are preferred over neat ones at mole fractions of 0.2 and 0.5, whereas same‐species interactions become predominant at a mole fraction of 0.8. In particular, at a mole fraction of 0.2 same‐species interactions are almost absent with a CN close to 0, but the CN increases to 0.17 and 0.73 at mole fractions of 0.5 and 0.8, respectively. This is due to a smaller number of acetone molecules able to be coordinated by the HFIP, forcing the same‐species interaction to happen more frequently. The CNs for different binding motifs sum up neatly to 1.0, excluding the possibility of a significant presence of non‐associated monomeric species in the system. Considering the CNs at 450 pm, which is the location of the second minimum in the RDFs, the inter‐species interactions are preferred with a value of 1.3 against 0.9 of the H(HFIP)‐O(HFIP) interaction at a mole fraction of 0.5. However at a mole fraction of 0.8 the situation is different, with the same‐species interaction winning over the mixed ones with CNs of 2.2 and 0.6, respectively. A third peak is not visible at larger distances (RDFs up to 15 Å are included in the SI). This might indicate that circular configurations of neat HFIPs are present and maybe even preferred over chains at this mole fraction; however, this argument cannot exclude the presence of these linear formations. The bottom panels in Figure 1 show the ADFs of the hydrogen bond angle. Both the inter‐species and same‐species hydrogen bonds show similar behaviors and a clear preference for a linear arrangements.

**Figure 2:**
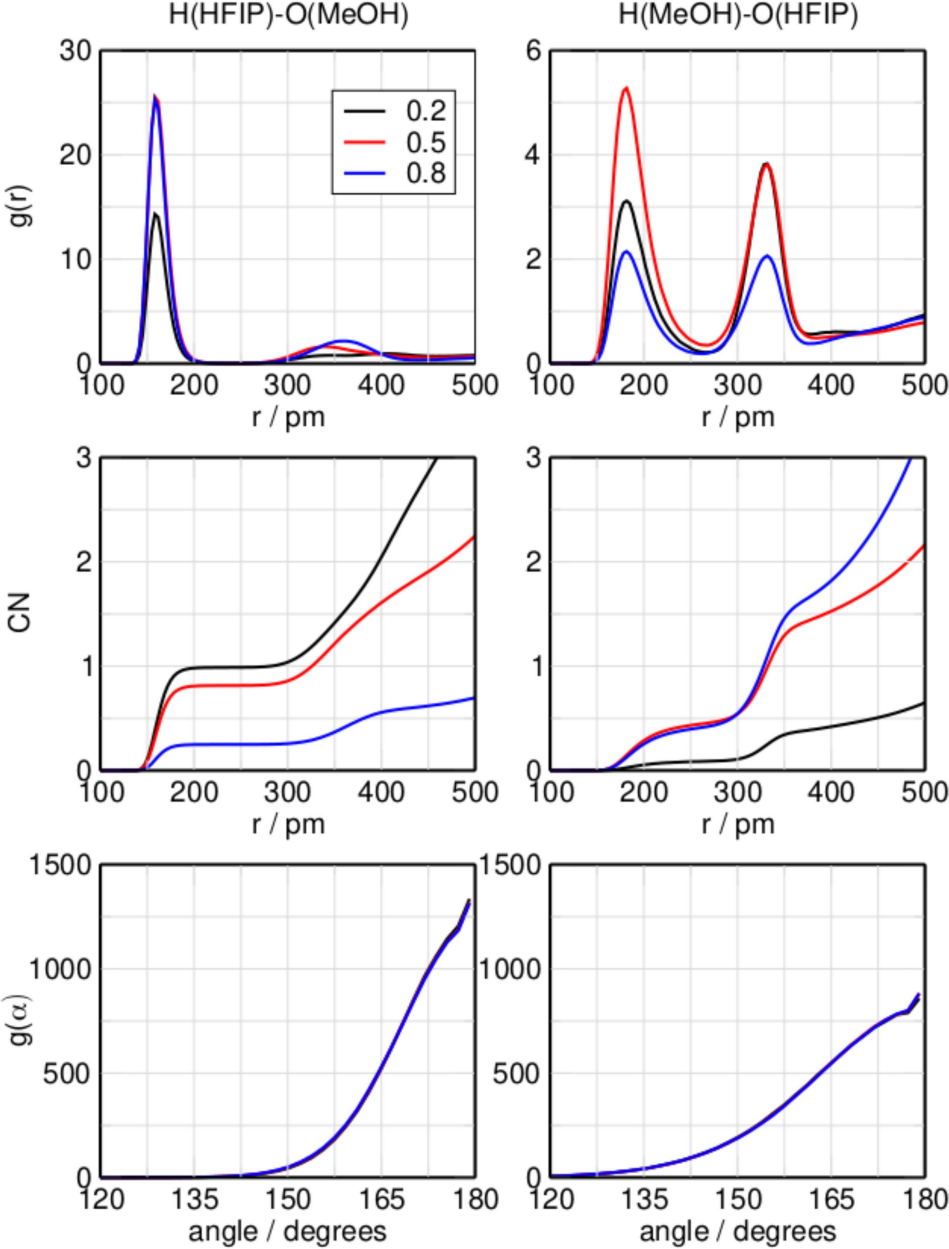
Radial distribution function, coordination number, and angular distribution function of the hydrogen bond for the system HFIP/methanol increasing the molar fraction of HFIP at 298.15 K with HFIP bond donor and MeOH acceptor (left), or MeOH donor and HFIP acceptor (right).

**Figure 3:**
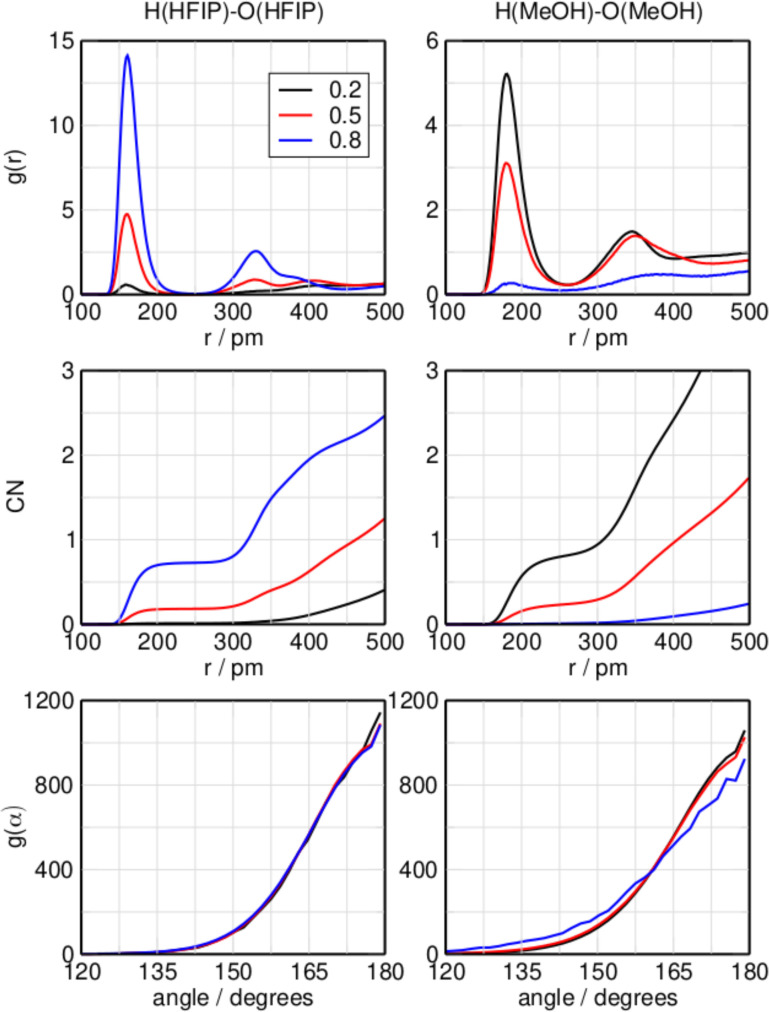
Radial distribution function, coordination number, and angular distribution function of the hydrogen bond for the system HFIP/methanol increasing the molar fraction of HFIP at 298.15 K, with HFIP donor and acceptor (left) or MeOH donor and acceptor (right).

In contrast to the HFIP/acetone mixture, both compounds in the HFIP/methanol can act as hydrogen bond donor or acceptor. Figure 2 shows the RDFs of the inter‐species hydrogen bond length with HFIP acting as donor and acceptor, respectively. Looking at the (H)HFIP‐(O)MeOH interaction first, the average hydrogen bond length is located at 160 pm. A second peak emerges at 340 pm as the mole fraction increases from 0.2 to 0.5, showing the possible presence of MeOH in the second structure coordination shell of HFIP. This peak shifts to a slightly larger distance of 360 pm at higher HFIP concentrations. Here, the CNs are 0.98, 0.82, and 0.25 at mole fractions of 0.2, 0.5, and 0.8 respectively, showing a similar behavior with an increasing concentration of HFIP as in the HFIP/acetone system. The ADFs show a similar preference for linear hydrogen bonds as observed for the HFIP/acetone mixture. Moving on to the (H)MeOH‐(O)HFIP interaction, which has HFIP acting as hydrogen bond acceptor and methanol as donor, it is immediately clear that interactions of this kind are much less prevalent in the mixture than hydrogen bonds of the opposite direction. At all three mole fractions two peaks are present at 181 pm and 333 pm, showing the first and second solvation shell of HFIP, respectively. The larger distance of 181 pm shows that the hydrogen bond donated by methanol is weaker than that donated by HFIP. Their position remains the same independent of the mixture's composition. The left and right side of Figure 3 show the RDF, CN, and ADF of the same‐species hydrogen bonds shared between two molecules of HFIP and two molecules of methanol, respectively. The RDF of the (H)HFIP‐(O)HFIP hydrogen bond shows a prominent peak at 161 pm and, similar to the same interaction in the mixture HFIP/acetone, as well as an additional peak at 324 pm at mole fractions of 0.5 and 0.8. Also similar to the previous mixture, the CN plot shows that only at mole fractions greater than 0.5 this interaction becomes predominant. In the RDF of the (H)MeOH‐(O)MeOH interaction a peak is present at 180 pm. A second peak at 340 pm can be observed at mole fractions of 0.2 and 0.5. This peak is still weakly present at 0.8 around 380 pm. With an increasing mole fraction of HFIP, the same‐species interaction of methanol with itself are concentrated in the first solvation shell, with a second solvation shell being present but less populated. The CN suggests that the MeOH‐MeOH hydrogen bond is more significant in the equimolar mixture than the HFIP‐HFIP hydrogen bond. As for the previous system, the CN values for the biding motifs sum up to 1. The CNs of the different interaction motifs sum up neatly to 1.0, which shows that non‐associated monomeric species aren't present in significant amounts. Considering the CNs at 400 pm, which is the location of the second minimum in the RDFs shown in Figures 2 and 3, it is possible to get some insight over the geometric structures of these interactions, as for the previous system. At a mole fraction of HFIP of 0.2, the CNs the inter‐species interactions H(HFIP)‐O(MeOH) and H(MeOH)‐O(HFIP) are 2.0 and 0.4, respectively, whereas the CN of the same‐species interaction H(MeOH)‐O(MeOH) is 2.4. The same‐species interaction between two molecules of HFIP is negligible. The situation is different at a mole fraction of 0.5, where the CNs of both the mixed interactions show similar values of 1.6 for H(HFIP)‐O(MeOH) and 1.5 for H(MeOH)‐O(HFIP). The same‐species hydrogen bonds formed by pairs of HFIP or methanol have a CN of 0.6 and 1.0, respectively. At a mole fraction of 0.8, the CN of the H(HFIP)‐O(MeOH) interaction drops to 0.6 and the CN of the H(HFIP)‐O(HFIP) interaction has a value of 2.0. For this reason it is possible to assume that at this mole fraction, the neat interaction of HFIP with itself is dominant; however, a high CN of 1.8 for the H(MeOH)‐O(HFIP) interaction suggests the presence of clusters where a single or several methanol molecules are coordinated by a larger number of HFIP molecules. As for the HFIP/acetone system, circular clusters seem to be a good description of the system; however, linear oligomers cannot be ruled out, and there is proof in literature of chain configuration in pure methanol.^[67]^

Overall, all the ADFs of this mixture at all mole fractions show that linear interactions are preferred.

The lifetimes *τ* of the hydrogen bonds in both mixtures were calculated as explained in the computational details and are presented in Table 2. A longer lifetime means a stronger bond, as it requires more time to break. In the top part of the table, the lifetimes of hydrogen bonds for the system HFIP/Acetone are listed. The H(HFIP)‐O(Ace) hydrogen bond has a lifetime of 9 ps, whereas the same‐species H(HFIP)‐(O)HFIP hydrogen bond has a significantly shorter lifetime of 3 ps. With an increasing mole fraction of HFIP, the lifetimes of the inter‐species hydrogen bonds increase, meanwhile the same‐species interaction remains stable. In the bottom part of Table 2, the hydrogen bond lifetimes for the system HFIP/MeOH are presented. All four interaction types are considered. At a mole fraction of 0.2, the inter‐species H(HFIP)‐O(MeOH) hydrogen bond has a lifetime of 48 ps, which indicates this interactions as the preferred one in this system. With an increasing mole fraction of HFIP, the lifetime of this hydrogen bond decreases. The (H)MeOH‐(O)MeOH hydrogen bond lifetimes are in good agreement with both experimental and calculated data from literature.[^68^, ^69^, ^70^] H(MeOH)‐O(HFIP) is confirmed to be weaker than the other inter‐species interaction.

**Table 2:** Lifetimes of the hydrogen bonds in both HFIP/Acetone and HFIP/MeOH increasing the molar fraction of HFIP at 298.15 K.

	**HFIP/Acetone**		
	*τ*/ps(0.2)	*τ*/ps(0.5)	*τ*/ps(0.8)
H(HFIP)‐O(Ace)	9	11	16
H(HFIP)‐O(HFIP)	3	4	4
			
	**HFIP/MeOH**		
	*τ*/ps(0.2)	*τ*/ps(0.5)	*τ*/ps(0.8)
H(HFIP)‐O(MeOH)	48	45	43
H(MeOH)‐O(HFIP)	2	2	2
H(HFIP)‐O(HFIP)	11	11	11
H(MeOH)‐O(MeOH)	4	4	2

### 2.2 Cluster analysis

Multiple clusters are optimized following the procedure described in the computational details section above to build the cluster sets which serve as input for the bQCE method. One of the most important features of the bQCE method is that it assigns populations to all clusters in the cluster set. The goal is to find the equilibrium distribution of all clusters so that they reproduce the reference isobars. The monomer‐normalized population gives a measure of the importance of specific clusters and interaction motifs in the system. These populations are available for each temperature in the investigated temperature range.

**Figure 4:**
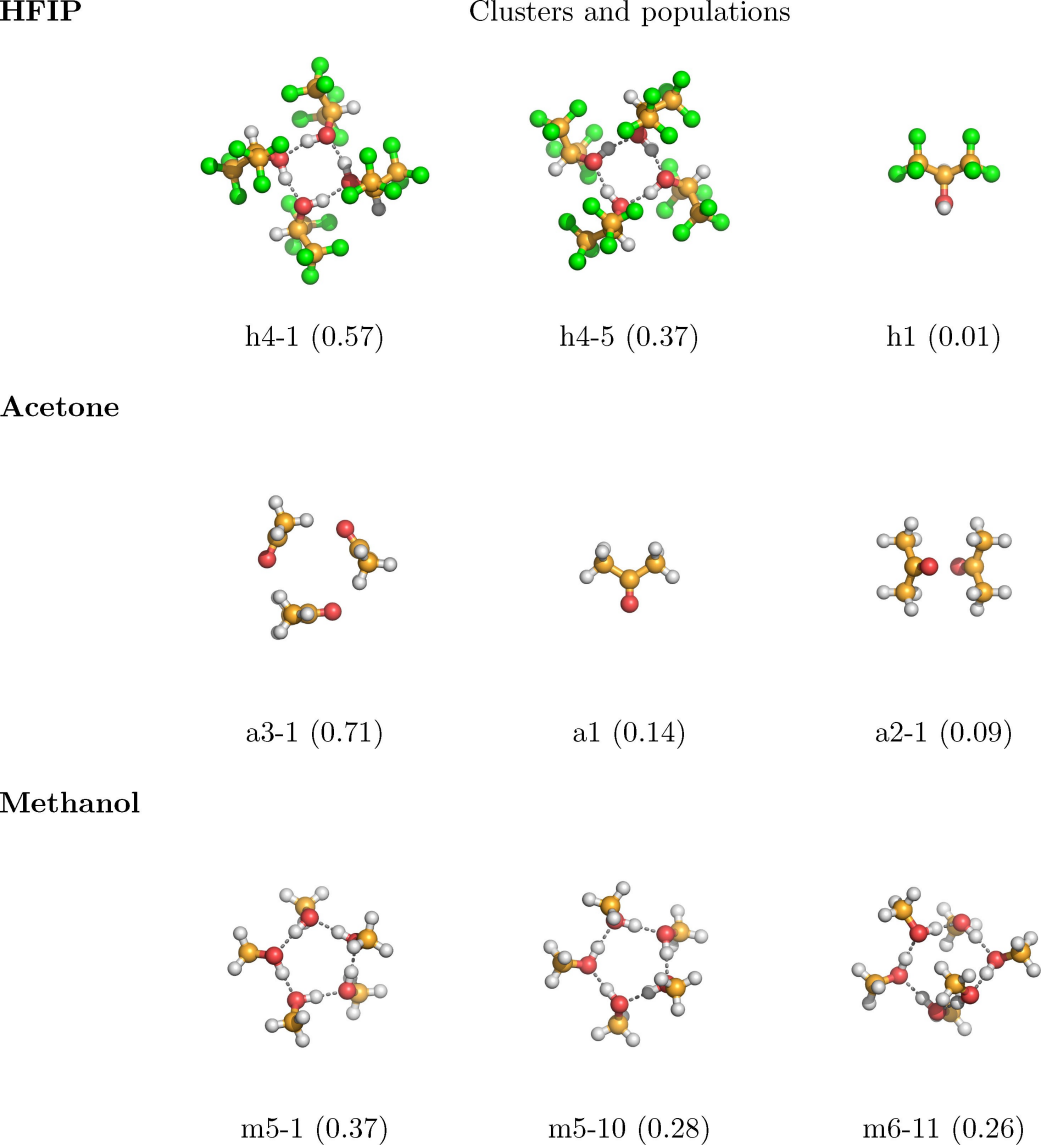
Structures and relative populations of the most populated clusters of the neat systems acetone (a), HFIP (h), methanol (m) at 298.15 K.

In Figure 4 the most populated clusters in neat systems of pure HFIP, methanol, and acetone are shown for the temperature of 298.15 K. In the HFIP system, the tetramers h4‐1 and h4‐5 dominate the neat solvent, with populations of 0.57 and 0.37, respectively. In acetone the cyclic trimer a3‐1 is the highest populated cluster with a population of 0.71. However, a significant amount of acetone molecules in the system exist in the form of non‐associated monomers. The methanol system is dominated by ring formations, where the cyclic pentamers m5‐1, m5‐10, and hexamer m6‐11 are the preferred geometries with values 0.37, 0.28, and 0.26, respectively.

**Figure 5:**
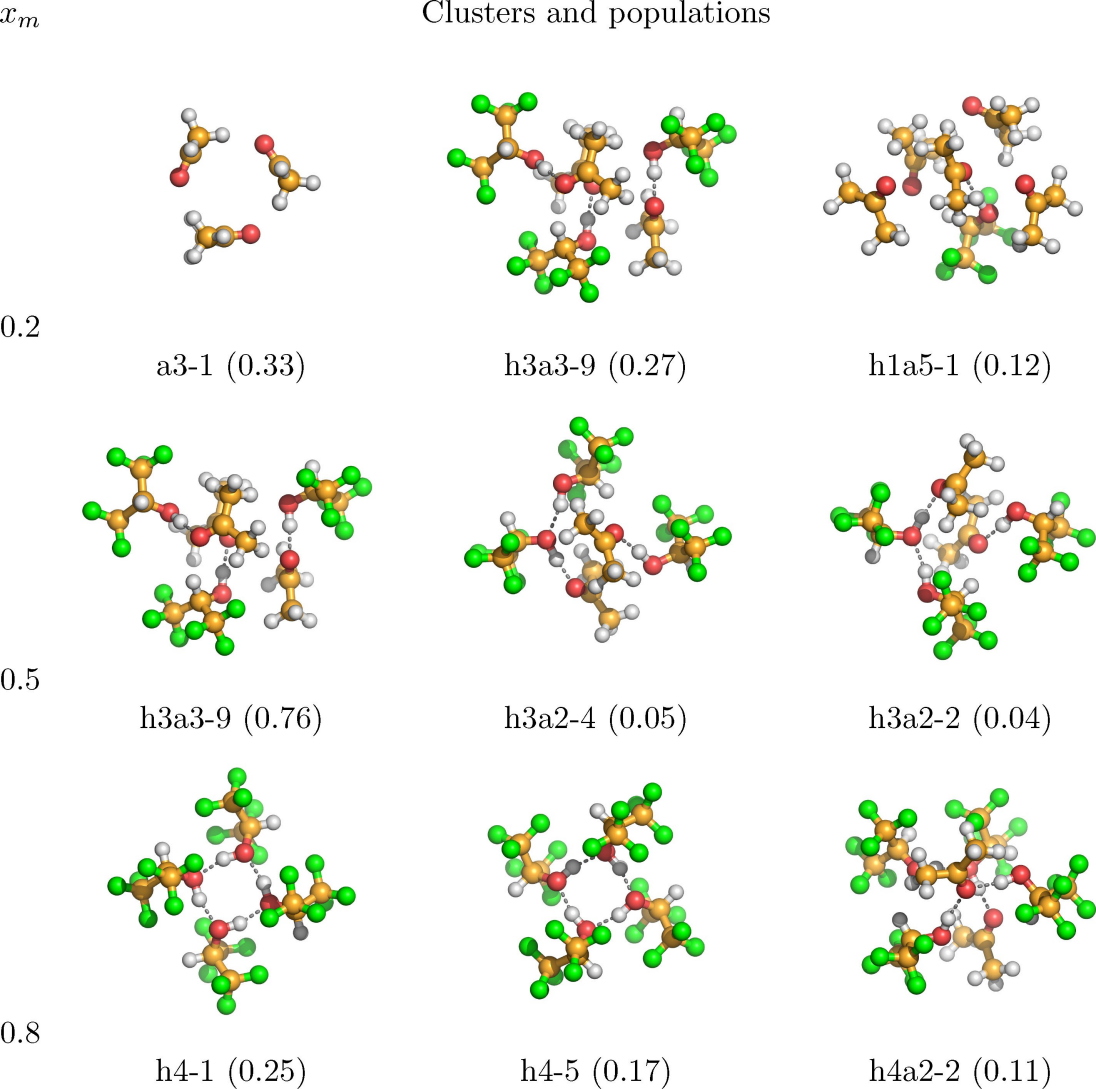
Structures and relative populations of the most populated clusters of the system HFIP/acetone at the molar fraction of HFIP 0.2, 0.5, 0.8 at 298.15 K.

**Figure 6:**
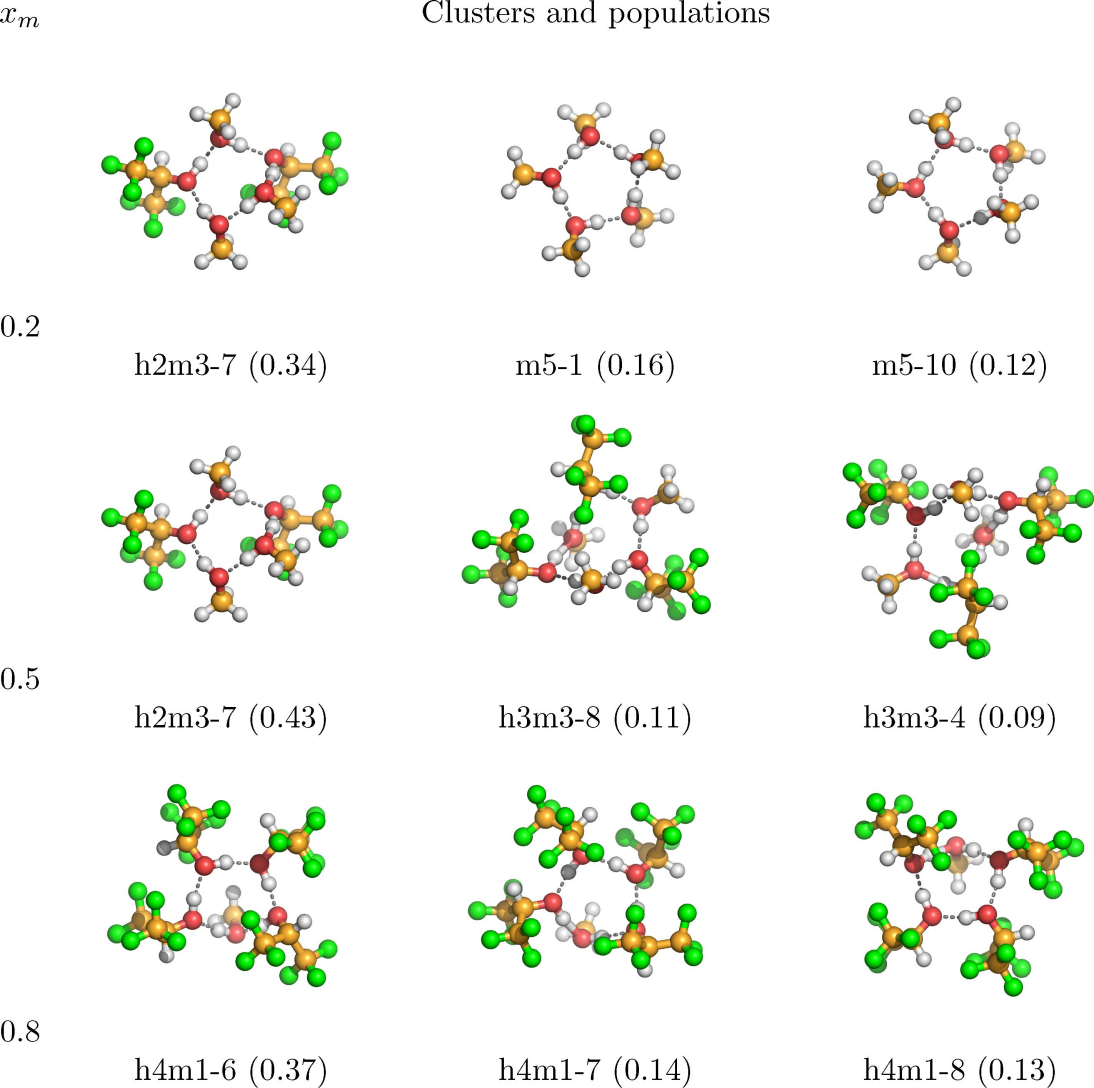
Structures and relative populations of the most populated clusters of the system HFIP/methanol at the molar fraction of HFIP 0.2, 0.5, 0.8 at 298.15 K.

Figures 5 and 6 show visualizations of the most populated clusters in the binary mixtures HFIP/acetone and HFIP/methanol at 298.15 K, respectively. Since dozens of clusters have been considered for both systems, here only the most populated ones are presented. Cartesian coordinates and visualizations of all cluster geometries are available in the supporting information. Focusing on Figure 5 first, at the mole fraction 0.2 the neat acetone trimer a3‐1, which consists of three acetone molecules arranged in a triangular ring structure, is the most populated cluster with a value of 0.33, followed by the mixed hexamer h3a3‐9 with 0.27. The same cluster, which can be described as aggregation of three alternating hydrogen bonded HFIP/acetone dimers, is the most populated cluster in the equimolar mixture of HFIP and acetone with a population of 0.76. Please note that clusters with compositions that differ from the system's molar composition can be populated as the bQCE method will automatically find a cluster distribution that is consistent with the system's molar composition. In agreement with the CNs measured from MD simulations of the same system (Figure 1), there are no same‐species bonds of HFIP in this cluster.

In contrast, at the higher mole fraction of 0.8, the neat HFIP tetramers are the most populated, which feature four HFIP molecules arranged in a quadratic ring of hydrogen bonds, each acting as both acceptor and donor. This indicates a mixture in which at lower concentrations of HFIP the inter‐species hydrogen bond between HFIP and acetone is the most common interaction, in agreement with the CN plots in Figure 1. There is a good agreement between the CN at 400 pm and the RDF of the neat HFIP interactions and the most populated clusters at this mole fraction; already the classical simulation suggests the presence of neat HFIP rings at a mole fraction of 0.8. The distance between one H atom and the O atom on the molecule opposite to it in the tetramer is 327 pm, really close to the second RDF peak for the same‐species interaction at 326 pm in Figure 1. At higher concentrations of HFIP, cooperative effects between multiple HFIP molecules become more important and the system is dominated by hydrogen bonded ring formations, while mixed interactions are still significant.

In Figure 6 the most populated clusters of the HFIP/methanol mixture are displayed. At low concentration of HFIP, the highly coordinated hydrogen bond ring formations of the neat methanol pentamers dominate the system. But already at the low mole fraction of 0.2, the mixed h2m3‐7 cluster, which contains hydrogen bonded HFIP and methanol molecules forming a ring in an alternating pattern, is highly populated. The possible presence of these kind of clusters was already suggested when discussing Figures 2 and 3. The significance of the mixed h2m3‐7 cluster carries over to the equimolar mixture, where it is still the highest populated cluster and more populated than the stoichiometrically favored clusters h3m3‐8 and h3m3‐4, which are the second and third highest populated clusters. At high concentrations of HFIP, and in contrast to the HFIP/acetone mixture, the system is dominated by the mixed clusters h4m1‐6, h4m1‐7, and h4m1‐8. This is in good agreement with the conclusions drawn from Figures 2 and 3.

Overall, there is a good agreement for both systems between the classical MD simulations and the populations calculated via the bQCE approach.

**Figure 7:**
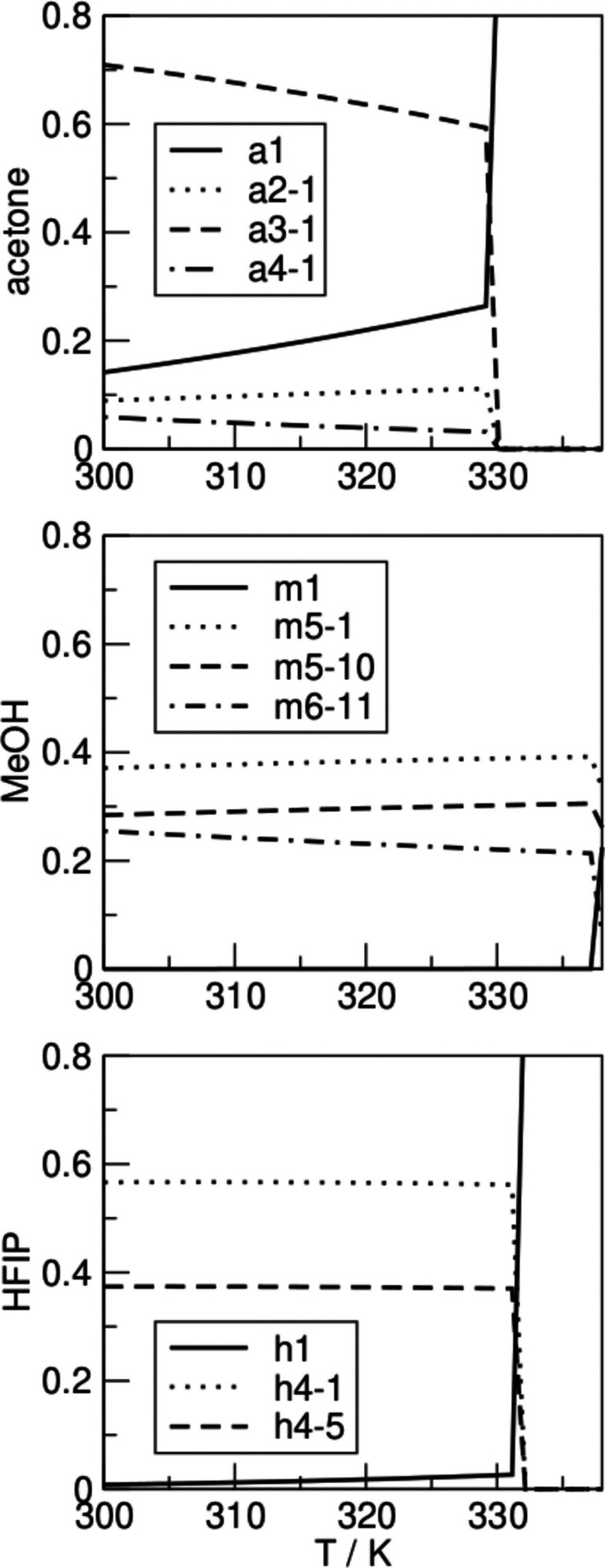
Populations of neat acetone (a), MeOH (m), and HFIP (h) (from top to bottom) in the temperature range of 298.15‐338.15 K.

**Figure 8:**
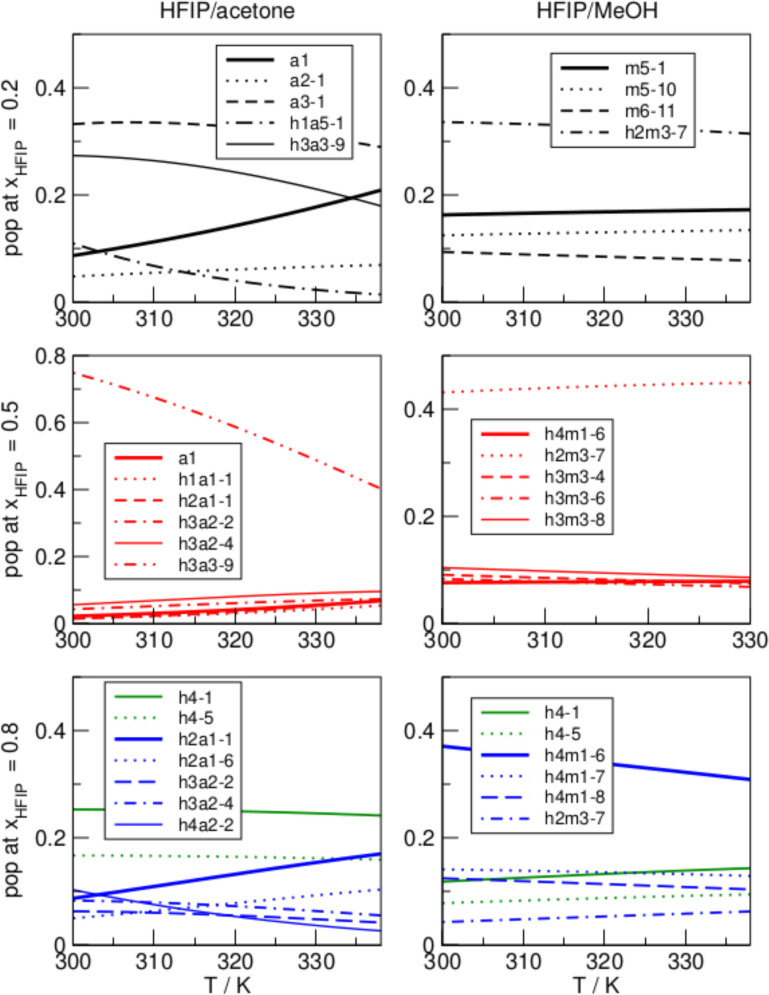
Populations of acetone (a), HFIP (h), MeOH (m) and mixed clusters at (from top to bottom) 0.2, 0.5, and 0.8 HFIP molar fraction, in the temperature range of 298.15–338.15 K.

For now, the most significant clusters were considered only at room temperature. However, as the temperature changes other cluster formations might emerge and begin to dominate the bulk structure. The bQCE model provides such information and allows insight into the temperature dependence of the cluster equilibrium distribution in the selected temperature range of 298.15–338.15 K. Here, we will take a look at the populations of neat and mixed clusters and their evolution with temperature, which are shown in Figures 7 and 8 for different mole fractions of HFIP in the mixtures HFIP/acetone and HFIP/methanol, where their population is significant (higher than 0.05). The top panel in Figure 7 shows the cluster populations in neat acetone. The trimeric a3‐1 cluster dominates the system, but with rising temperature its population decreases in favor of the acetone monomer. In the center panel, cluster populations in the methanol system are presented. The pentamers m5‐1 and m5‐10 together with the hexamer m6‐11 dominate the system until the boiling point is reached. Several research groups investigated neat methanol with the QCE approach. A recent work by Teh and coworkers,^[71]^ which extensively investigates methanol cluster populations with both DFT and MP2 geometry optimization, states the most populated cluster is the octamer, which is not investigated in this article due to the computational time required, as explained in the computational details section. An older work by Kelterer and coworkers^[64]^ finds instead that the most populated cluster size is the hexamer, followed by the pentamer, regardless that also clusters up to the octamer were investigated. The differences between those works and this article can be imputed to the geometry optimization and frequency calculation at different levels of theory,^[71]^ or to a different procedure to generate the clusters.^[64]^ However, there is a general agreement that multi‐molecular cyclic clusters are preferred until the boiling point, while in the gas phase the monomer dominates the population. In the bottom panel, the cluster populations in neat HFIP show that the tetramers h4‐1 and h4‐5 dominate the system until the boiling point.

Figure 8 shows the cluster populations in the mixed systems at different mole fractions of HFIP. At low concentrations of HFIP, the neat acetone trimer is the preferred cluster at 298.15 K, whereas the monomer population increases significantly with rising temperature. This is due to the breaking of the enthalpically favored inter‐species hydrogen bonds (calculated interaction energy of −53.6 kJ/mol) and the rise of entropically favored small clusters such as the acetone dimer (calculated interaction energy of −26.8 kJ/mol) and the non‐associated acetone monomer. Pure HFIP clusters are not populated even at higher temperature. Instead, HFIP is bound in mixed clusters, of which the hexamer h3a3‐9 is the most populated. In the equimolar HFIP/acetone mixture, the hexamer h3a3‐9 is highly populated at 298.15 K, but with rising temperature decreases in population in favor of the acetone monomer and smaller mixed clusters dominated by HFIP. At high concentrations of HFIP, neat acetone clusters are almost absent. Meanwhile, the tetrameric neat HFIP clusters h4‐1 and h4‐5 are significantly populated, which is in agreement with earlier observations of the emergence of cooperativity effects at high HFIP concentrations. With rising temperature, the population of mixed clusters increases.

The right column of Figure 8 shows the cluster populations in the HFIP/methanol. In contrast to the other mixture, the methanol‐dominated mixed cluster h2m3‐7 is more populated at low concentrations of HFIP and the populations are nearly constant over temperature. The equimolar mixture is again composed mainly by mixed clusters, in particular the methanol‐dominated h2m3‐7, whereas neat clusters are absent. At high concentrations of HFIP, the HFIP‐dominant mixed cluster h4m1‐6 dominates the system, but with rising temperature its population decreases in favor of pure HFIP clusters. Neat HFIP clusters are significantly populated, but less than in the HFIP/acetone mixture.

For both systems, the formation of close inter‐species interactions is favored over interactions of the same species. This behavior is more pronounced for the HFIP/methanol mixture.

To complete the quantum cluster equilibrium analysis, the interaction energies, the hydrogen bond lengths, and the hydrogen bond angles of the dimers, are presented in Table 3, wherein for mixed dimers the first named species is the hydrogen bond donor and the last named species is the hydrogen bond acceptor. It is apparent that the interaction energies of the mixed dimers, where HFIP acts as the donor, are significantly stronger than that of any neat dimer. Both the bond lengths and interaction energies of the isolated dimers indicate the importance of inter‐species interactions over same‐species interactions if HFIP is the hydrogen bond donor, in line with the MD results presented before, where there are exclusively H(HFIP)‐O(Ace) and H(HFIP)‐O(MeOH) interactions at mole fractions of HFIP of 0.2 and 0.5.

**Table 3:** Hydrogen bond distances, angles, and interaction energy of different dimers Δ*E* at the BP86/TZVP level of theory in kJ/mol. The hydrogen bond donor is written before the acceptor.

Dimer	Δ*E*	*r* [pm]	$\alpha \left[{}^{\circ }\right]$
(HFIP)_2_	−21.6	184	168.74
(MeOH)_2_	−23.6	183	169.78
(Ace)_2_	−23.1	–	–
HFIP‐MeOH	−36.8	170	175.44
MeOH‐HFIP	−12.8	201	165.52
HFIP‐Ace	−37.6	170	170.46

### 2.3 Thermodynamic properties of neat and mixed systems

Through calculating the system's total partition function based on the equilibrium distribution of a set of representative clusters, the bQCE method grants access to the absolute thermodynamic functions such as the Gibbs energy *G*, enthalpy *H*, and entropy *S* at any investigated temperature. Using the bQCE method, we can thus calculate properties such as the enthalpy and entropy of vaporization $\Delta_{\mathrm{vap}}H$ and $\Delta_{\mathrm{vap}}S$ as simple difference of the liquid phase and a gas phase reference. Already in earlier works we were able to establish our procedure of using a so‐called QCE^0^ calculation, wherein *a*_mf_ is set to 0 removing all inter‐cluster interactions, as gas phase reference.[^27^, ^32^, ^72^] Table 4 compares the calculated vaporization enthalpies and entropies of the neat systems to their experimental reference values at room temperature and the boiling point. Overall, good to excellent agreement with the experimental reference is achieved for all systems. This is true both at room temperature and at boiling points of the solvents. In all cases, $\Delta_{\mathrm{vap}}H$ is slightly overestimated, possibly indicating an over‐stabilization of the liquid phase. The largest deviation is observed for methanol at its boiling point. A likely explanation is the experimentally observed aggregation of methanol molecules to small clusters in the gas phase,^[73]^ which is not properly sampled by the QCE^0^ calculation, as the monomers are populated with 99 %. This is in agreement with our already published calculated $\Delta_{\mathrm{vap}}H$ of 39.33 kJ/mol of methanol at room temperature presented in a previous work, calculated at the same level of theory.^[27]^ Here, we obtain a slightly different value due to the changes in methodology.

**Table 4:** Calculated and experimental enthalpy $\Delta_{\mathrm{vap}}H$ in kJ/mol and entropy $\Delta_{\mathrm{vap}}S$ in J/mol K of vaporization of the neat substances at 298.15 K and at the boiling point temperature. Experimental values, where present, were taken from the NIST Chemistry WebBook.^[74]^

Solvent	T	$\Delta_{\mathrm{vap}}^{\mathrm{calc}}H$	$\Delta_{\mathrm{vap}}^{\exp}H$	$\Delta_{\mathrm{vap}}^{\mathrm{calc}}S$	$\Delta_{\mathrm{vap}}^{\exp}S$
Acetone	298.15	32.65	31.27	98.38	95.00
	329.30	30.11	29.10	91.45	88.37
HFIP	298.15	42.25	41.60	126.42	–
	331.35	40.96	–	123.62	–
Methanol	298.15	40.14	37.6	99.45	114.89
	337.70	39.89	35.21	114.23	104.26

Enthalpies and entropies of vaporization at 298.15 K were calculated for the mixed systems as well, see Table 5. For HFIP/acetone the calculated vaporization enthalpies are higher than those of the neat components, with the highest value calculated for a mole fraction of 0.5. In contrast, the entropies of vaporization are always higher than that of pure acetone, but lower than that of pure HFIP. In HFIP/methanol at a mole fraction of 0.2 the calculated enthalpy of vaporization is lower than those of the neat components. At higher mole fractions, the enthalpy of vaporization is higher than those in either of the neat components, similar to the HFIP/acetone system. The same behavior is observed for the entropy of vaporization.

**Table 5:** Enthalpies and entropies of vaporization at 298.15 K for the HFIP/Acetone mixture (left) and the HFIP/MeOH mixture (right) at the *x*_m_ molar fraction of HFIP. Enthalpies in kJ/mol, Entropies in J/mol K.

	HFIP/Acetone			HFIP/MeOH	
*x*_m_	$\Delta_{\mathrm{vap}}^{\mathrm{calc}}H$	$\Delta_{\mathrm{vap}}^{\mathrm{calc}}S$		$\Delta_{\mathrm{vap}}^{\mathrm{calc}}H$	$\Delta_{\mathrm{vap}}^{\mathrm{calc}}S$
0.2	43.39	111.75		35.75	102.12
0.5	46.57	125.08		55.62	111.37
0.8	46.01	121.06		42.65	119.51

## 3 Conclusions

The mixtures of HFIP with methanol and acetone were investigated. First, molecular dynamics simulations were performed to get the isobars of the systems for the temperatures 298.15, 308.15, 318.15, 328.15, and 338.15 K at mole fractions of HFIP of 0.2, 0.5, and 0.8, respectively. Then the structure of the hydrogen bond from the simulations were analyzed and discussed. Molecular dynamics simulations and hydrogen bond evaluation from the dimer clusters calculated at DFT level are in good agreement. Together with the population analysis and the evaluation of the most populated clusters, we are presenting two strongly interacting mixtures, with some hints that the HFIP/methanol system presents a stronger hydrogen bond framework. Clusters of up to six molecules were optimized at DFT level for both the mixtures and the neat systems. The hydrogen bonds of the dimers were analyzed. The calculated clusters, from simulated isobars and experimental data, where available, were used as input for the binary quantum cluster equilibrium method to get the thermodynamical properties. The enthalpies and entropies of vaporization of the neat systems are in good agreement with previous theoretical results and with experimental values. In addition, enthalpies and entropies of vaporization were calculated for the mixed systems.

## Conflict of interest

The authors declare no conflict of interest.

## Supporting information

As a service to our authors and readers, this journal provides supporting information supplied by the authors. Such materials are peer reviewed and may be re‐organized for online delivery, but are not copy‐edited or typeset. Technical support issues arising from supporting information (other than missing files) should be addressed to the authors.

Supporting Information
