# Supplementary material for: Hydrogen Bonding and Vaporization Thermodynamics in Hexafluoroisopropanol‐Acetone and ‐Methanol Mixtures. A Joined Cluster Analysis and Molecular Dynamic Study
Source: Chemphyschem. 2021 Nov 11;23(1):e202100620. doi: 10.1002/cphc.202100620 (PMC9298724; doi:10.1002/cphc.202100620)
Supplement: Supplementary file 1 — Supporting Information [file CPHC-23-0-s001.pdf]

ChemPhysChem

Supporting Information

Hydrogen Bonding and Vaporization Thermodynamics in Hexafluoroisopropanol-Acetone and -Methanol Mixtures. A Joined Cluster Analysis and Molecular Dynamic Study

Gwydyon Marchelli, Johannes Ingenmey, Oldamur Hollóczki, Alain Chaumont, and Barbara Kirchner*

Supporting Information

I. MOLECULAR DYNAMICS SIMULATIONS

A. Force field parameters

ϵ (kcal/mol)	σ (Å)	atom type
HFIP		
0.097098	3.3611	CT
0.071041	3.1578	F
0.000000	0.0000	HO
0.028321	2.3734	HC
0.203255	2.9548	OH
Acetone		
0.105000	3.7500	C
0.066000	3.5000	CT
0.030000	2.5000	HC
0.210000	2.9600	O
MeOH		
0.066000	3.5000	CT
0.170000	3.1200	OH
0.000000	0.0000	HO
0.030000	2.5000	HC

TABLE I: Pair coefficients.

K_r (kcal/(molÅ^2))	r (Å)	Bond type
HFIP		
734.000	1.3600	CT-F
268.000	1.5300	CT-CT
320.000	1.3600	CT-OH
2000.000	1.0900	CT-HC (must be fixed)
2000.000	1.0000	OH-HO (must be fixed)
Acetone		
317.000	1.5220	C - CT
570.000	1.2290	C - O
340.000	1.0900	CT - HC
MeOH		
320.000	1.4100	CT-OH
340.000	1.0900	CT-HC
553.000	0.9600	OH-HO

TABLE II: Bond coefficients.

K_r (kcal/(mol rad^2))	r (θ)	Angle type
HFIP		
55.048	107.600	F -CT- F
55.048	111.000	F -CT-CT
55.048	110.000	CT-CT-CT
55.048	111.000	CT-CT-OH
55.048	109.500	CT-CT-HC
47.548	109.500	OH-CT-HC
47.548	109.500	CT-OH-HO
Acetone		
70.000	116.000	CT - C - CT
80.000	120.400	O - C - CT
35.000	109.500	C - CT - HC
33.000	107.800	HC - CT - HC
MeOH		
50.000	109.500	OH-CT-HC
35.000	109.500	HC-CT-HC
55.000	108.500	CT-OH-HO

TABLE III: Angle coefficients.

HFIP					
all dihedrals set as harmonic 0 1 0					
Acetone					
Style	V1	V2	V3	V4	torsion type
opls	0.000	0.000	0.275	0.000	CT - C - CT - HC
opls	0.000	0.000	0.000	0.000	O - C - CT - *
MeOH					
opls	0.000	0.000	4.500	0.000	HO-OH-CT-HC

TABLE IV: Dihedral Coefficients.

q (Coulomb)	atom type
HFIP	
0.60000	CT
-0.20000	F
-0.07000	CT
-0.59500	OH
0.17000	HC
0.49500	HO
Acetone	
0.47000	C
-0.18000	CT
0.06000	HC
-0.47000	O
MeOH	
0.14500	CT
-0.68300	OH
0.41800	HO
0.04000	HC

TABLE V: Charges

B. Number of molecules in the molecular dynamics simulations

System	x_m	HFIP molecules	Ace molecules	MeOH molecules
HFIP	–	350	–	–
MeOH	–	–	–	1720
Ace	–	–	950	–
HFIP/Ace	0.2	100	400	–
HFIP/Ace	0.5	210	210	–
HFIP/Ace	0.8	280	70	–
HFIP/MeOH	0.2	192	–	768
HFIP/MeOH	0.5	480	–	480
HFIP/MeOH	0.8	768	–	192

TABLE VI: Number of molecules of neat and mixed simulated systems at each molar fraction of HFIP

C. Cell volumes

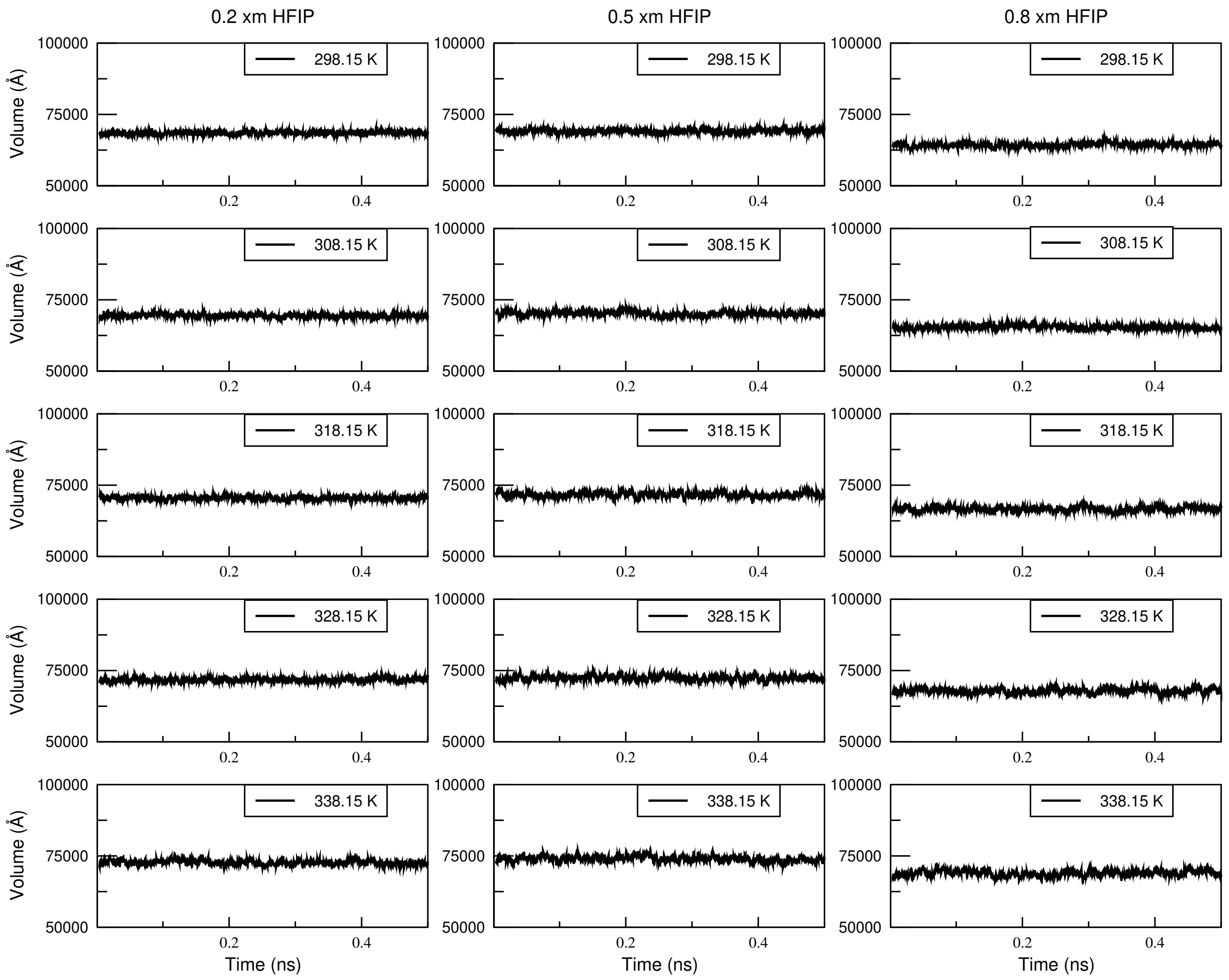

FIG. 1: Cell volume with respect to the simulation time in the last 0.5 ns of npt run for the systems HFIP/acetone

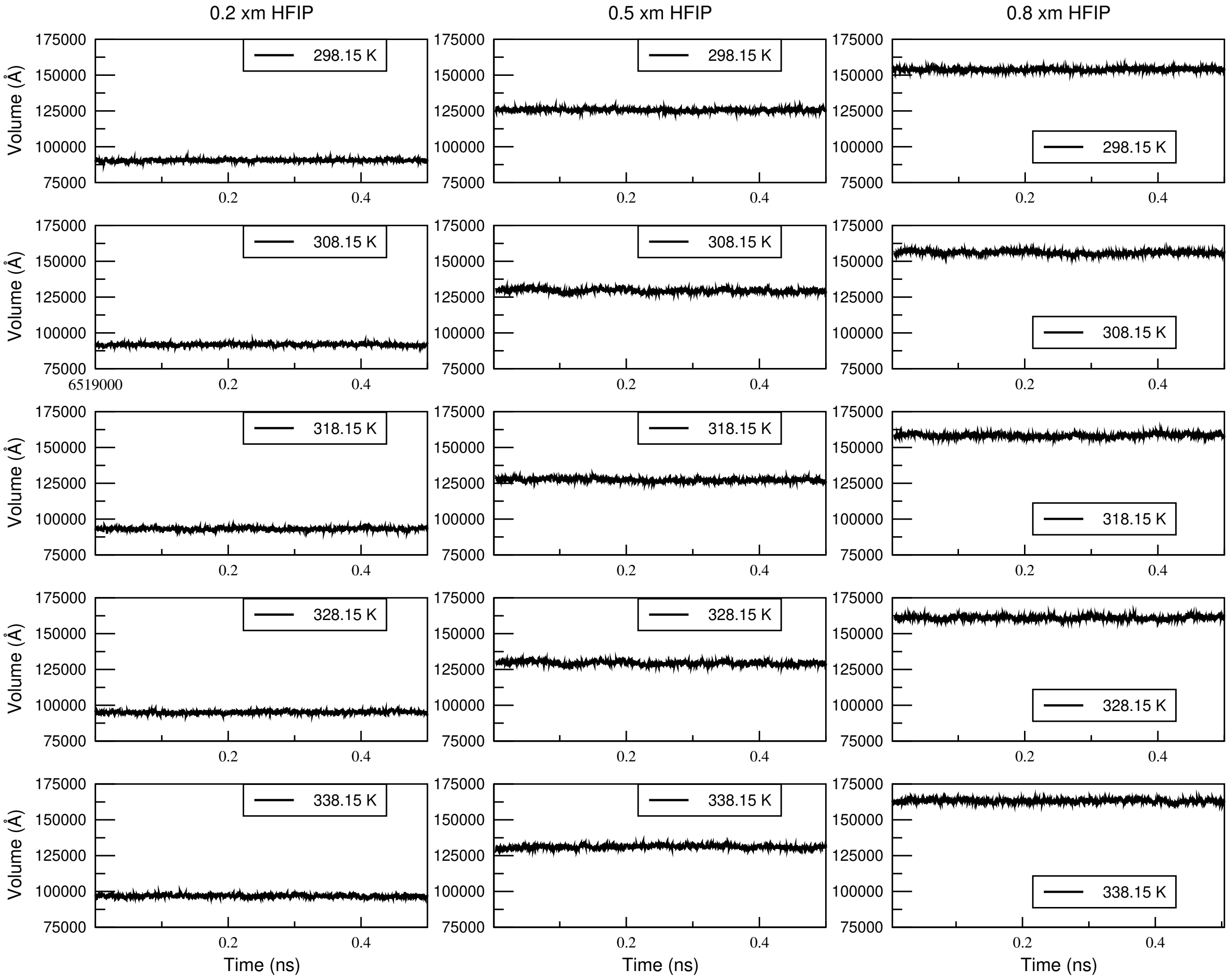

FIG. 2: Cell volume with respect to the simulation time in the last 0.5 ns of npt run for the systems HFIP/methanol

D. Neat systems

System	$\rho_{298}^{\mathrm{exp}}$	$\rho_{298}^{\mathrm{calc}}$
HFIP	1.596	1.521
MeOH	0.792	0.753
Acetone	0.784	0.777

TABLE VII: Experimental densities ρ^{exp} and calculated densities ρ^{calc} of the neat systems HFIP, MeOH, and acetone in g/cm^3. The densities are calculated from molecular dynamics simulations, at the same conditions as described in the computational details.

E. Molecular dynamics analysis at different temperatures

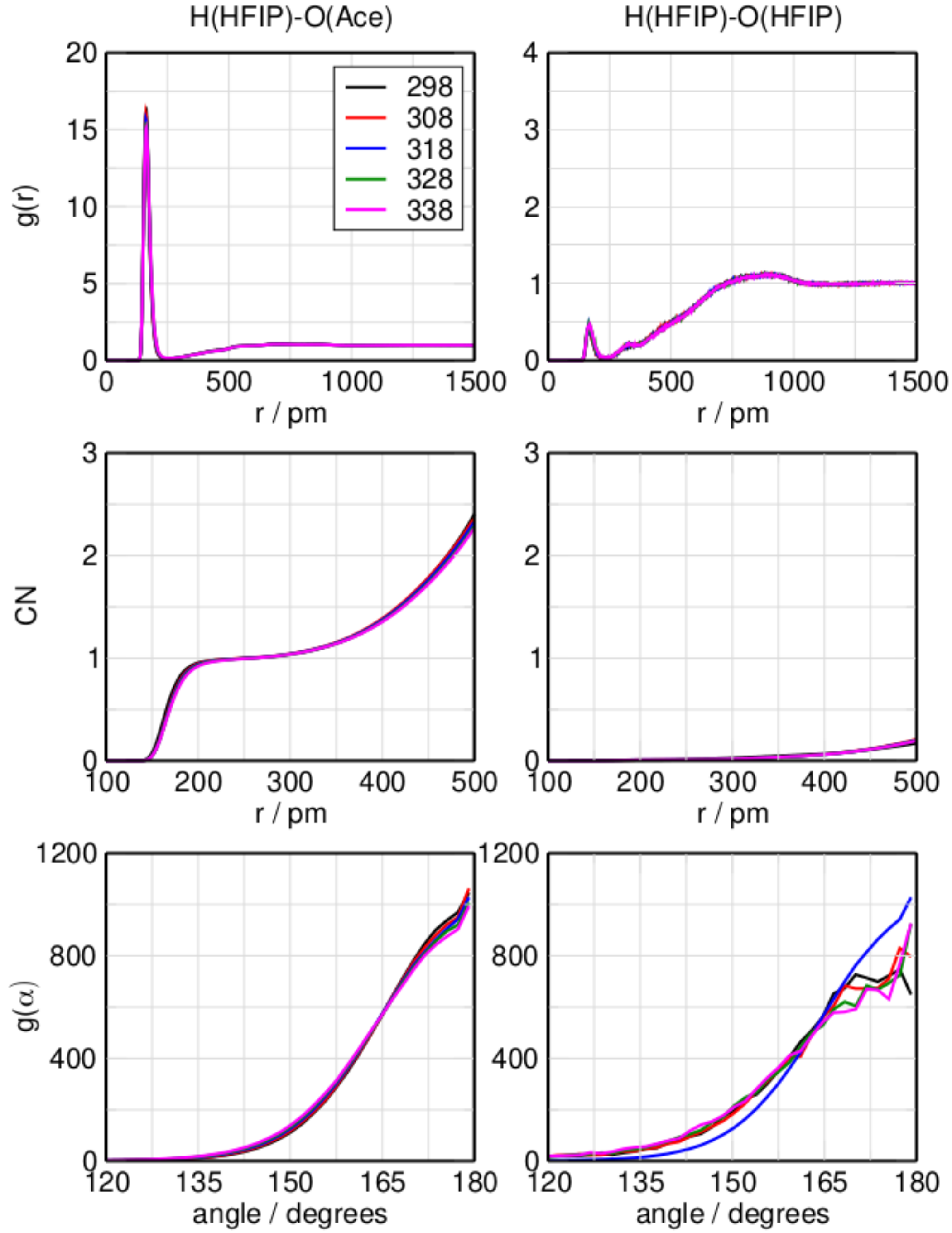

FIG. 3: Radial distribution function, numbers of integral, and angular distribution function of the hydrogen bond for the system HFIP/acetone at molar fraction of HFIP 0.2 at the temperatures 298.15 K, 308.15 K, 318.15 K, 328.15 K, and 338.15 K with HFIP bond donor and acetone acceptor (left) or HFIP (right).

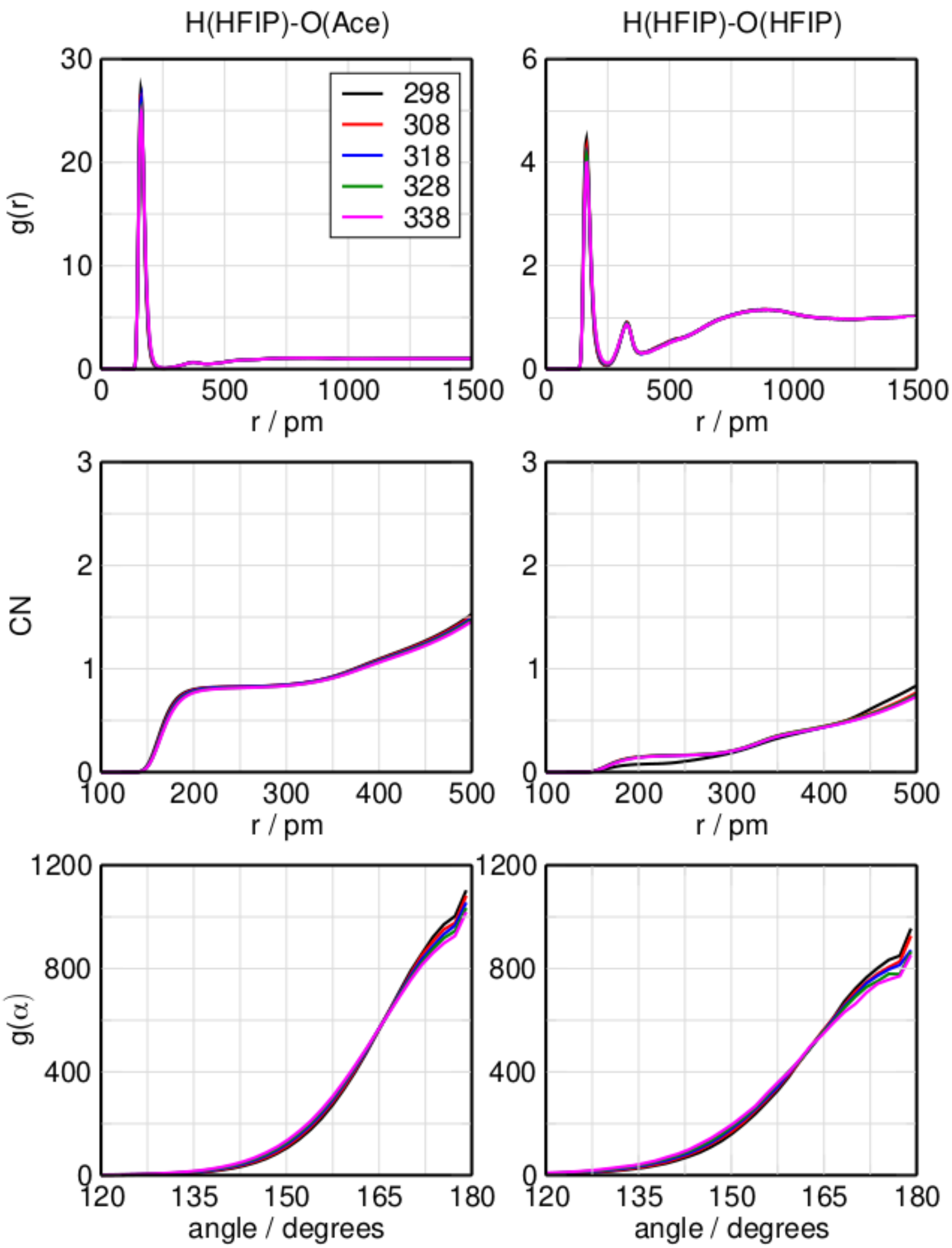

FIG. 4: Radial distribution function, numbers of integral, and angular distribution function of the hydrogen bond for the system HFIP/acetone at molar fraction of HFIP 0.5 at the temperatures 298.15 K, 308.15 K, 318.15 K, 328.15 K, and 338.15 K with HFIP bond donor and acetone acceptor (left) or HFIP (right).

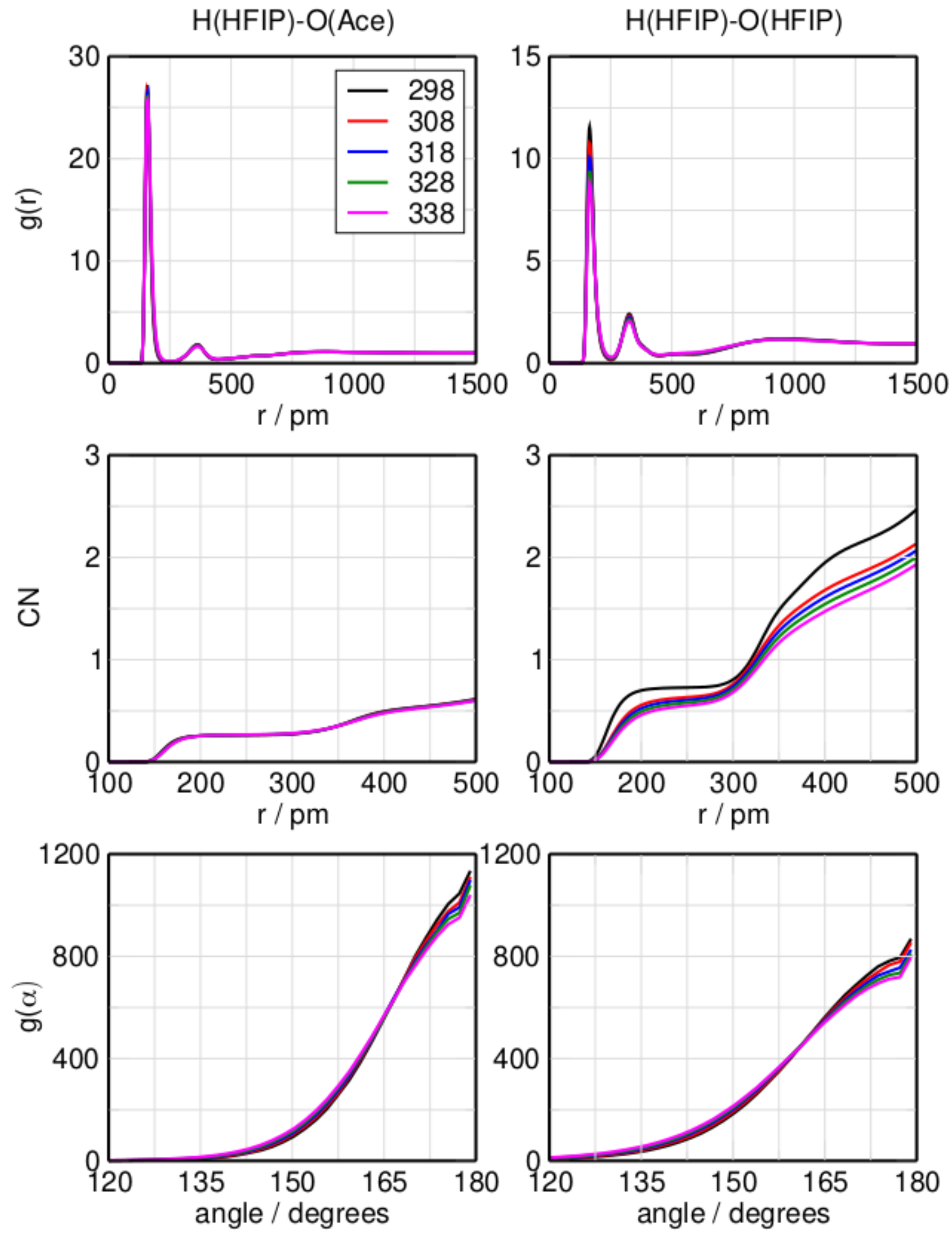

FIG. 5: Radial distribution function, numbers of integral, and angular distribution function of the hydrogen bond for the system HFIP/acetone at molar fraction of HFIP 0.8 at the temperatures 298.15 K, 308.15 K, 318.15 K, 328.15 K, and 338.15 K with HFIP bond donor and acetone acceptor (left) or HFIP (right).

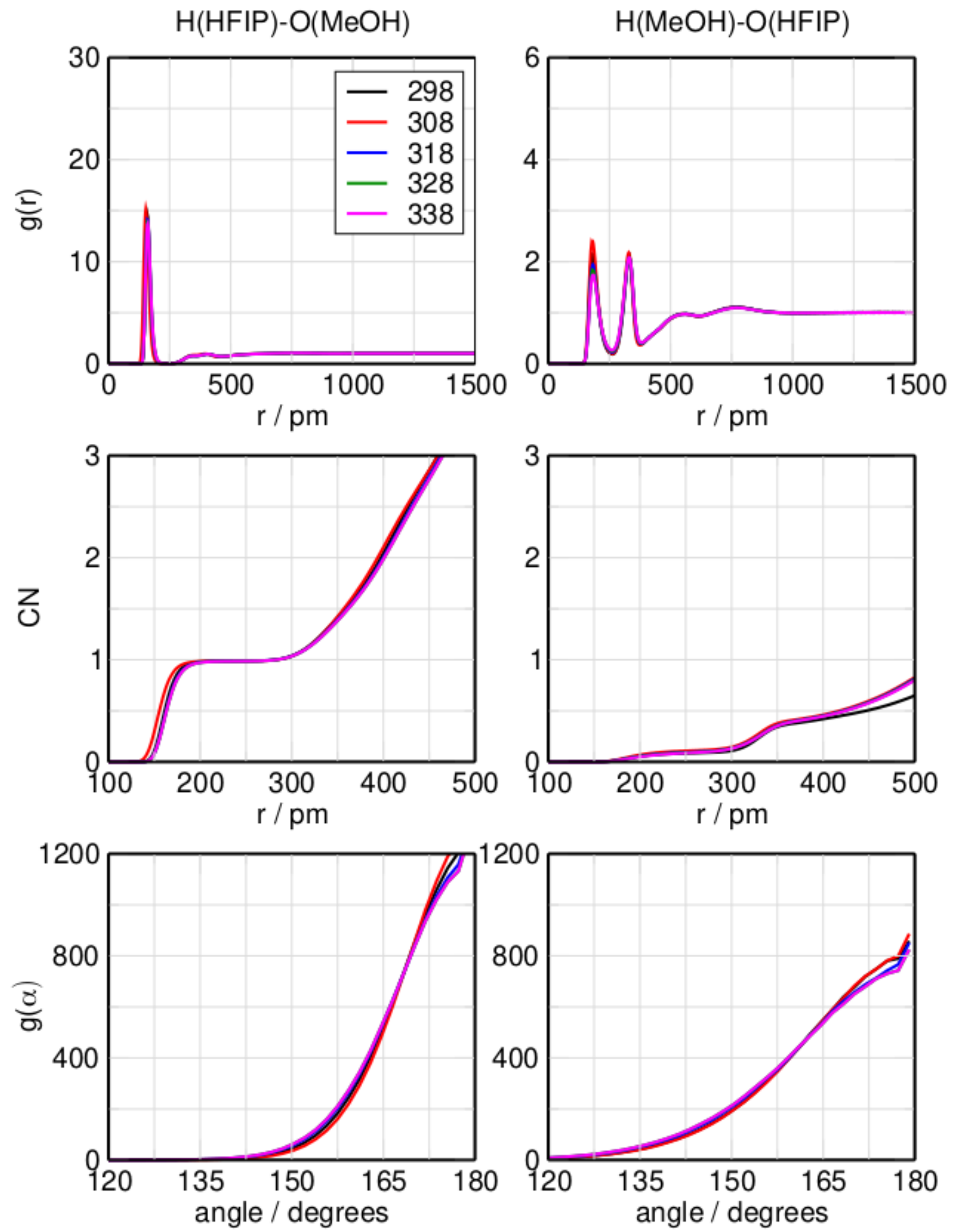

FIG. 6: Radial distribution function, numbers of integral, and angular distribution function of the hydrogen bond for the system HFIP/methanol at molar fraction of HFIP 0.2 at the temperatures 298.15 K, 308.15 K, 318.15 K, 328.15 K, and 338.15 K with HFIP bond donor and MeOH acceptor (left), or MeOH donor and HFIP acceptor (right).

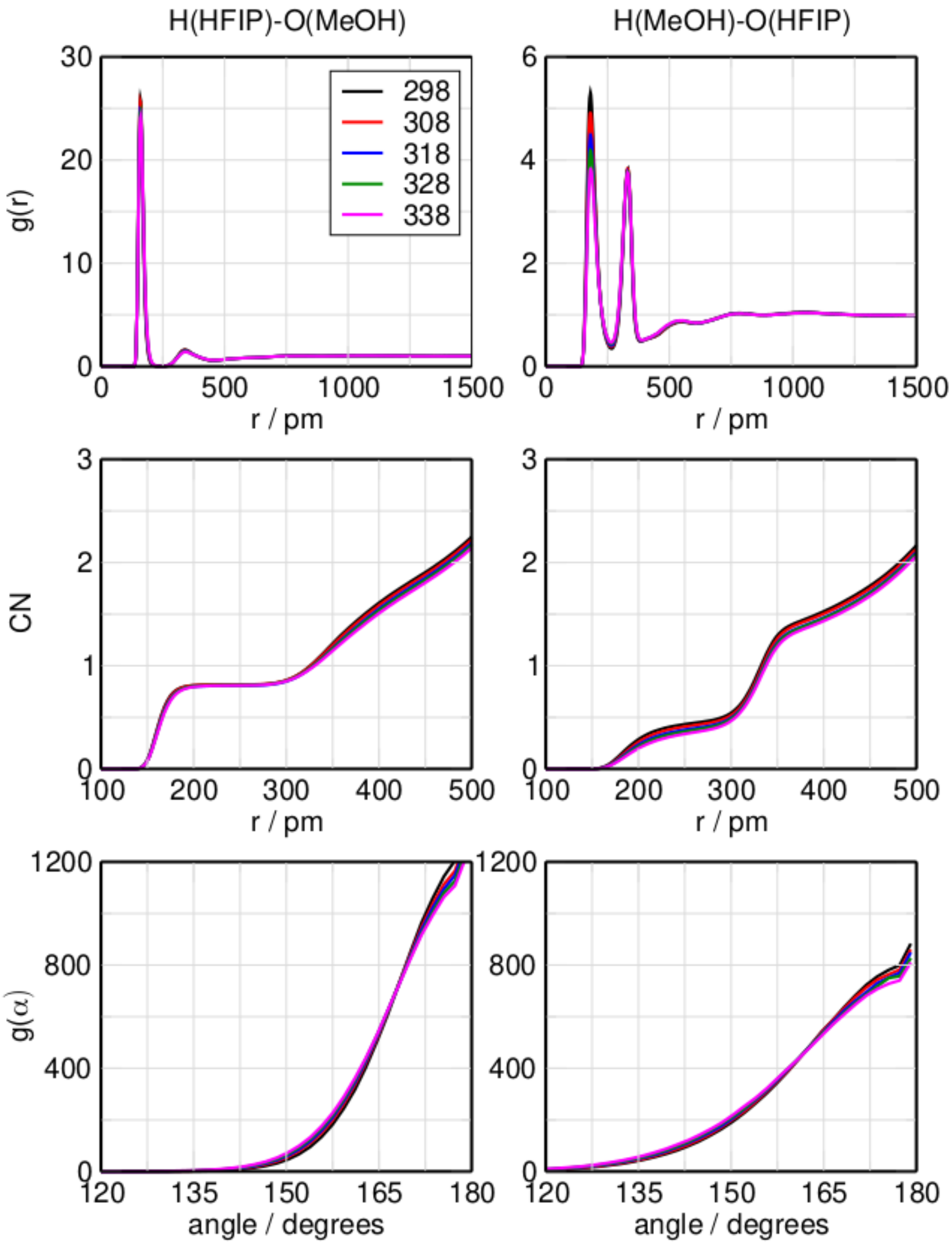

FIG. 7: Radial distribution function, numbers of integral, and angular distribution function of the hydrogen bond for the system HFIP/methanol at molar fraction of HFIP 0.5 at the temperatures 298.15 K, 308.15 K, 318.15 K, 328.15 K, and 338.15 K with HFIP bond donor and MeOH acceptor (left), or MeOH donor and HFIP acceptor (right).

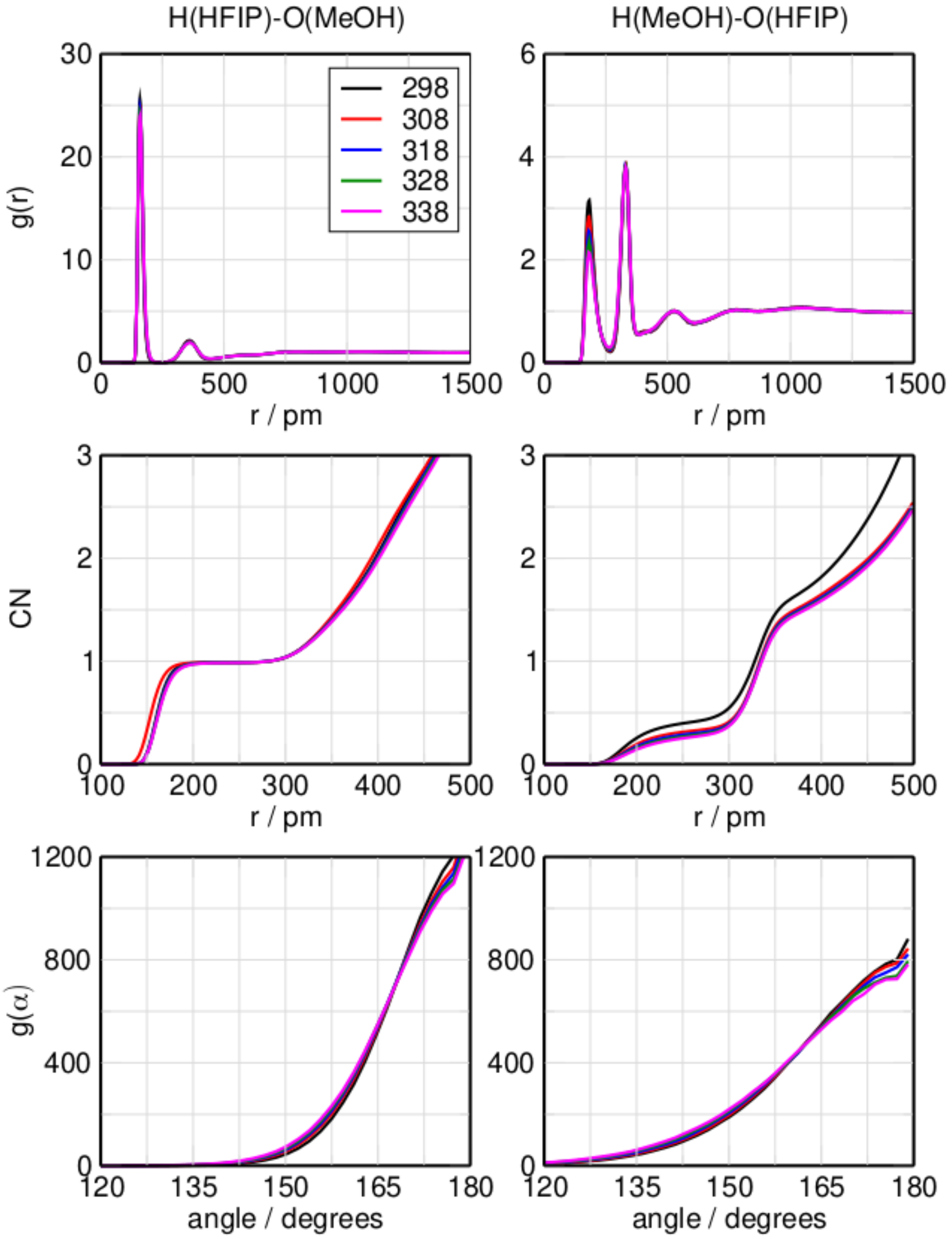

FIG. 8: Radial distribution function, numbers of integral, and angular distribution function of the hydrogen bond for the system HFIP/methanol at molar fraction of HFIP 0.8 at the temperatures 298.15 K, 308.15 K, 318.15 K, 328.15 K, and 338.15 K with HFIP bond donor and MeOH acceptor (left), or MeOH donor and HFIP acceptor (right).

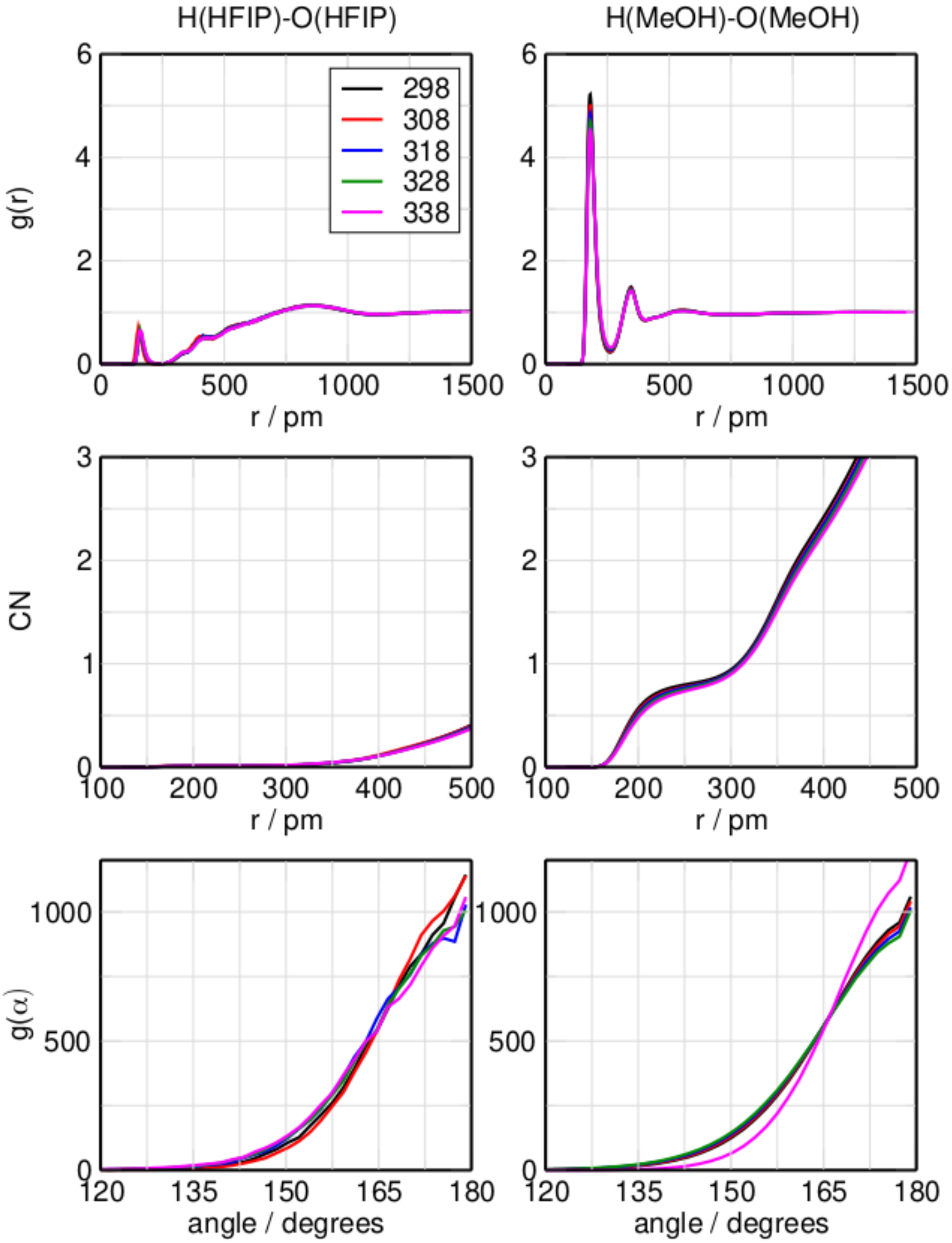

FIG. 9: Radial distribution function, numbers of integral, and angular distribution function of the hydrogen bond for the system HFIP/methanol at molar fraction of HFIP 0.2 at the temperatures 298.15 K, 308.15 K, 318.15 K, 328.15 K, and 338.15 K with HFIP donor and acceptor (left) or MeOH donor and acceptor (right).

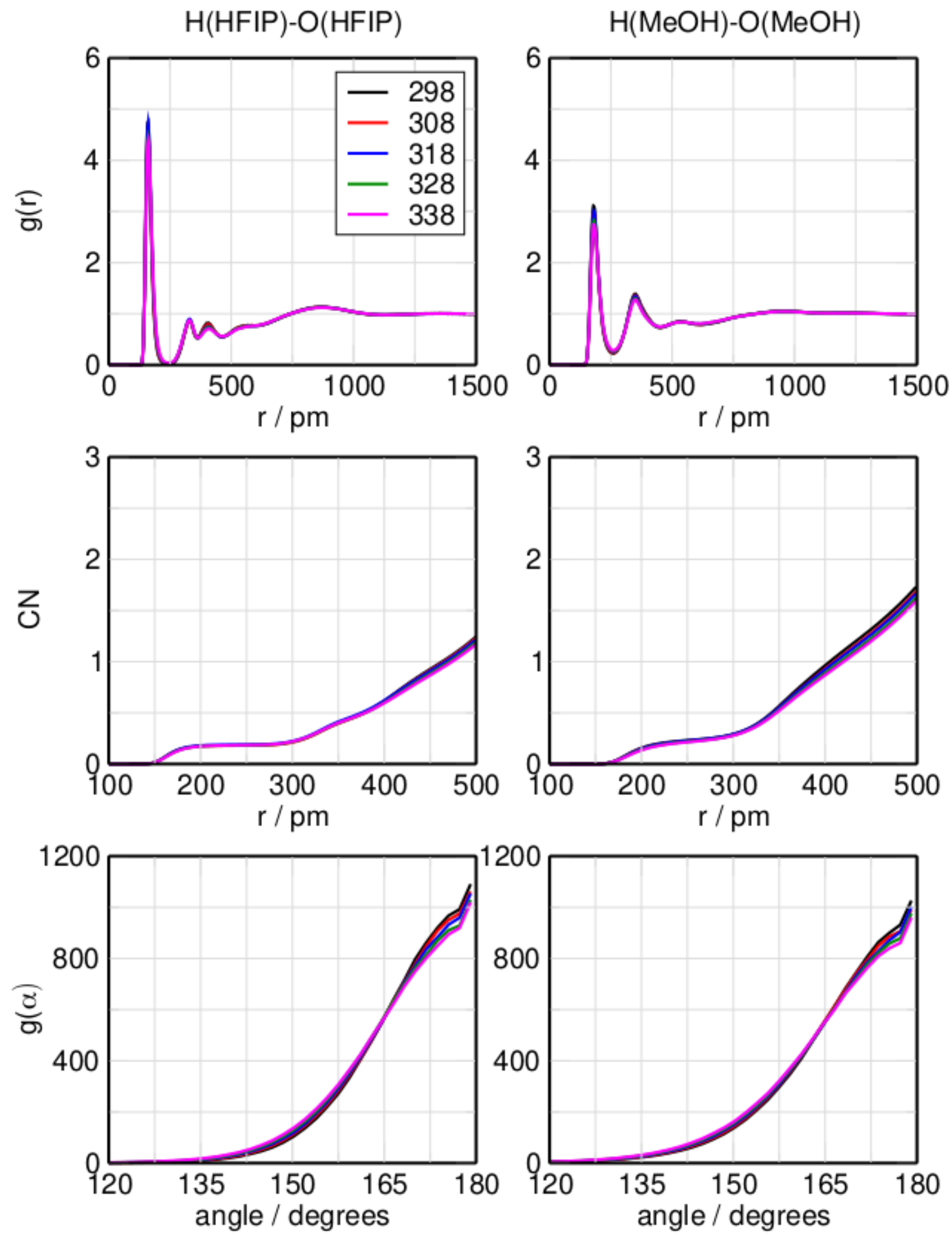

FIG. 10: Radial distribution function, numbers of integral, and angular distribution function of the hydrogen bond for the system HFIP/methanol at molar fraction of HFIP 0.5 at the temperatures 298.15 K, 308.15 K, 318.15 K, 328.15 K, and 338.15 K with HFIP donor and acceptor (left) or MeOH donor and acceptor (right).

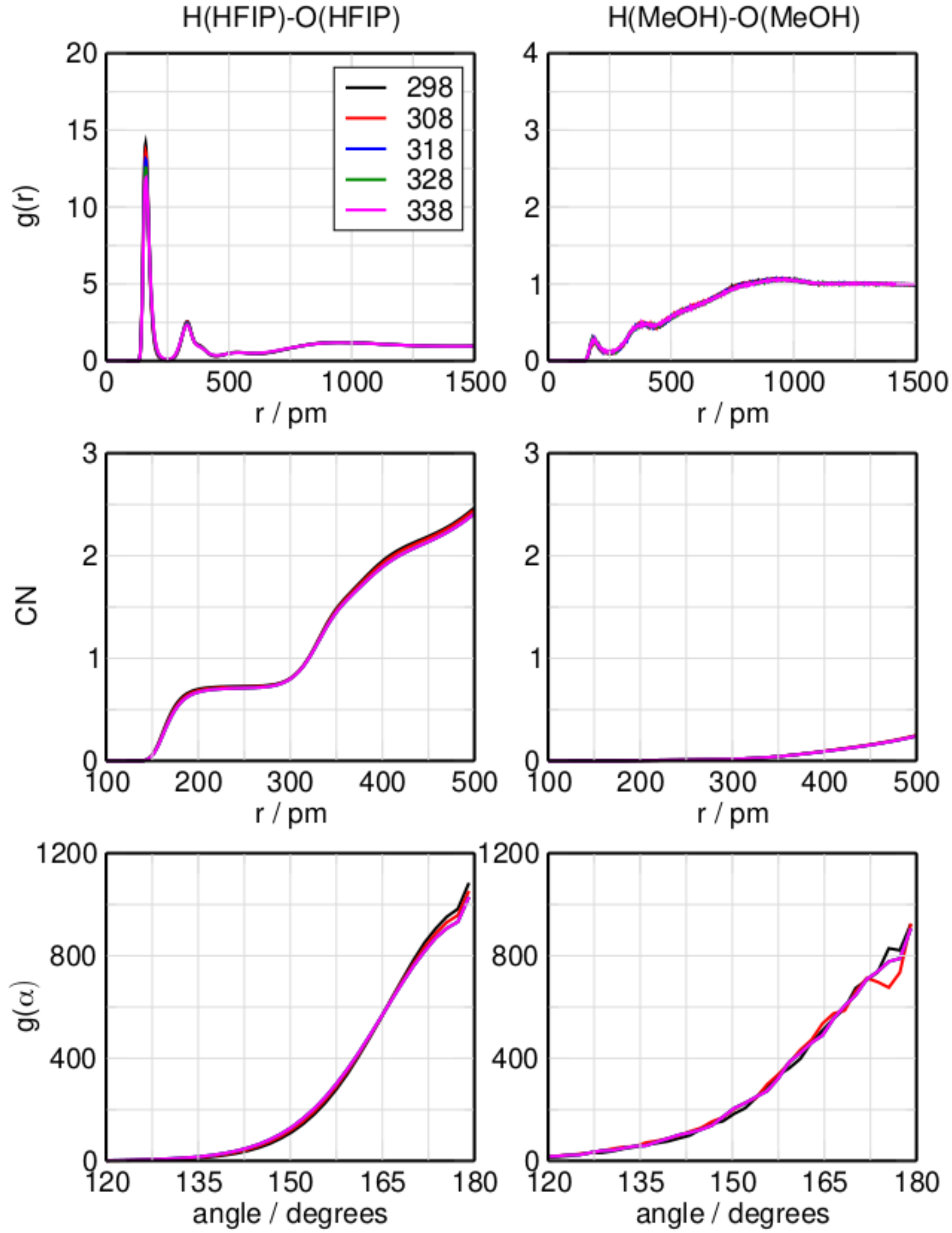

FIG. 11: Radial distribution function, numbers of integral, and angular distribution function of the hydrogen bond for the system HFIP/methanol at molar fraction of HFIP 0.8 at the temperatures 298.15 K, 308.15 K, 318.15 K, 328.15 K, and 338.15 K with HFIP donor and acceptor (left) or MeOH donor and acceptor (right).

II. CLUSTERS INTERACTION ENERGIES

cluster	size	E_{int}	E_{int}/m
h2-10	2	-25.2	-12.6
h3-1	3	-62.4	-20.8
h3-2	3	-62.3	-20.8
h3-6	3	-56.2	-18.7
h3-10	3	-61.6	-20.5
h4-1	4	-129.0	-32.2
h4-5	4	-129.0	-32.3
h5-1	5	-169.1	-33.8
h6-1	6	-207.6	-34.6
h6-2	6	-207.5	-34.6

TABLE VIII: Interaction energies and interaction energies per monomer of the system HFIP, in kJ/mol.

cluster	size	E_{int}	E_{int}/m
a2-1	2	-26.8	-13.4
a3-1	3	-53.6	-17.9
a4-1	4	-81.2	-20.3
a5-1	5	-114.7	-22.9
a5-8	5	-111.0	-22.2
a6-1	6	-143.8	-24.0
a6-4	6	-139.2	-23.2
a6-5	6	-139.9	-23.3
a6-8	6	-138.7	-23.1
a6-9	6	-135.3	-22.6
a6-10	6	-137.7	-23.0

TABLE IX: Interaction energies and interaction energies per monomer of the system acetone, in kJ/mol.

cluster	size	E_{int}	E_{int}/m
m2-1	2	-27.4	-13.7
m2-14	2	-27.4	-13.7
m3-1	3	-86.5	-28.8
m4-1	4	-152.3	-38.1
m4-7	4	-149.0	-37.2
m5-1	5	-201.3	-40.3
m5-10	5	-201.0	-40.2
m6-1	6	-246.6	-41.1
m6-2	6	-248.9	-41.5
m6-3	6	-248.8	-41.5
m6-4	6	-249.0	-41.5
m6-6	6	-247.5	-41.3
m6-11	6	-247.7	-41.3

TABLE X: Interaction energies and interaction energies per monomer of the system methanol, in kJ/mol.

cluster	size	HFIP	Ace	E_{int}	E_{int}/m	cluster	size	HFIP	Ace	E_{int}	E_{int}/m
h1a1-1	2	1	1	-43.6	-21.8	h2a4-2	6	2	4	-190.3	-31.7
h1a1-2	2	1	1	-38.0	-19.0	h2a4-9	6	2	4	-181.1	-30.2
h1a2-10	3	1	2	-71.0	-23.7	h3a1-1	4	3	1	-120.1	-30.0
h1a3-1	4	1	3	-108.2	-27.0	h3a1-4	4	3	1	-120.0	-30.0
h1a3-9	4	1	3	-108.2	-27.0	h3a1-10	4	3	1	-120.1	-30.0
h1a3-10	4	1	3	-108.2	-27.0	h3a2-1	5	3	2	-174.6	-34.9
h1a4-1	5	1	4	-134.1	-26.8	h3a2-2	5	3	2	-174.6	-34.9
h1a4-4	5	1	4	-138.4	-27.7	h3a2-4	5	3	2	-174.6	-34.9
h1a4-5	5	1	4	-138.5	-27.7	h3a2-10	5	3	2	-172.8	-34.6
h1a5-1	6	1	5	-180.9	-30.2	h3a3-4	6	3	3	-202.0	-33.7
h1a5-3	6	1	5	-173.6	-28.9	h3a3-7	6	3	3	-202.1	-33.7
h1a5-4	6	1	5	-165.5	-27.6	h3a3-8	6	3	3	-207.0	-34.5
h1a5-7	6	1	5	-175.1	-29.2	h3a3-9	6	3	3	-221.1	-36.9
h2a1-1	3	2	1	-84.1	-28.0	h4a1-1	5	4	1	-166.0	-33.2
h2a1-6	3	2	1	-82.8	-27.6	h4a1-3	5	4	1	-166.0	-33.2
h2a1-10	3	2	1	-85.2	-28.4	h4a1-4	5	4	1	-166.3	-33.3
h2a2-1	4	2	2	-117.1	-29.3	h4a1-5	5	4	1	-166.2	-33.2
h2a2-4	4	2	2	-117.2	-29.3	h4a1-6	5	4	1	-166.2	-33.2
h2a2-6	4	2	2	-117.1	-29.3	h4a2-1	6	4	2	-228.4	-38.1
h2a2-7	4	2	2	-117.1	-29.3	h4a2-2	6	4	2	-228.2	-38.0
h2a2-10	4	2	2	-117.2	-29.3	h5a1-1	6	5	1	-204.4	-34.1
h2a3-1	5	2	3	-164.8	-33.0	h5a1-3	6	5	1	-203.5	-33.9
h2a4-1	6	2	4	-187.2	-31.2						

TABLE XI: Interaction energies and interaction energies per monomer of the system HFIP-acetone, in kJ/mol.

cluster	size	HFIP	MeOH	E_{int}	E_{int}/m	cluster	size	HFIP	MeOH	E_{int}	E_{int}/m
h1m1-1	2	1	1	-42.7	-21.3	h2m3-1	5	2	3	-197.7	-39.5
h1m1-3	2	1	1	-42.7	-21.3	h2m3-2	5	2	3	-197.7	-39.5
h1m1-6	2	1	1	-42.7	-21.3	h2m3-6	5	2	3	-197.7	-39.5
h1m2-1	3	1	2	-85.0	-28.3	h2m3-7	5	2	3	-204.5	-40.9
h1m2-2	3	1	2	-85.0	-28.3	h2m3-8	5	2	3	-199.8	-40
h1m2-6	3	1	2	-85.0	-28.3	h2m4-1	6	2	4	-256.6	-42.8
h1m2-7	3	1	2	-84.2	-28.1	h2m4-4	6	2	4	-251.2	-41.9
h1m2-8	3	1	2	-84.2	-28.1	h2m4-6	6	2	4	-256.7	-42.8
h1m3-1	4	1	3	-150.6	-37.6	h2m4-7	6	2	4	-252.8	-42.1
h1m3-2	4	1	3	-150.6	-37.7	h3m1-1	4	3	1	-121.6	-30.4
h1m3-3	4	1	3	-147.8	-37	h3m1-6	4	3	1	-121.6	-30.4
h1m3-4	4	1	3	-150.6	-37.7	h3m1-7	4	3	1	-120.4	-30.1
h1m3-5	4	1	3	-150.6	-37.7	h3m2-1	5	3	2	-194.6	-38.9
h1m3-6	4	1	3	-150.6	-37.7	h3m2-3	5	3	2	-195.2	-39.0
h1m3-7	4	1	3	-150.9	-37.7	h3m2-4	5	3	2	-194.0	-38.8
h1m3-8	4	1	3	-150.9	-37.7	h3m2-7	5	3	2	-190.1	-38.0
h1m4-1	5	1	4	-202.8	-40.6	h3m2-8	5	3	2	-191.0	-38.2
h1m4-2	5	1	4	-202.8	-40.6	h3m3-1	6	3	3	-249.6	-41.6
h1m4-4	5	1	4	-202.8	-40.6	h3m3-3	6	3	3	-246.9	-41.1
h1m4-5	5	1	4	-202.8	-40.6	h3m3-4	6	3	3	-246.9	-41.1
h1m4-6	5	1	4	-202.8	-40.6	h3m3-8	6	3	3	-246.9	-41.1
h1m4-7	5	1	4	-201.9	-40.4	h4m1-1	5	4	1	-160.5	-32.1
h1m4-8	5	1	4	-199.5	-39.9	h4m1-2	5	4	1	-182.4	-36.5
h1m5-1	6	1	5	-256.5	-42.8	h4m1-6	5	4	1	-185.1	-37.0
h1m5-2	6	1	5	-255.6	-42.6	h4m1-7	5	4	1	-183.4	-36.7
h1m5-3	6	1	5	-255.6	-42.6	h4m1-8	5	4	1	-185.1	-37.0
h1m5-6	6	1	5	-255.7	-42.6	h4m2-1	6	4	2	-224.8	-37.5
h2m1-1	3	2	1	-78.6	-26.2	h4m2-2	6	4	2	-225.7	-37.6
h2m1-6	3	2	1	-78.6	-26.2	h4m2-4	6	4	2	-225.7	-37.6
h2m2-1	4	2	2	-123.5	-30.9	h4m2-6	6	4	2	-225.6	-37.6
h2m2-3	4	2	2	-123.5	-30.9	h4m2-7	6	4	2	-231.2	-38.5
h2m2-5	4	2	2	-123.5	-30.9	h4m2-8	6	4	2	-230.2	-38.4
h2m2-6	4	2	2	-122.1	-30.5	h5m1-2	6	5	1	-219.1	-36.5
h2m2-7	4	2	2	-122.1	-30.5	h5m1-5	6	5	1	-219.0	-36.5
h2m2-8	4	2	2	-122.1	-30.5	h5m1-6	6	5	1	-219.0	-36.5

TABLE XII: Interaction energies and interaction energies per monomer of the system methanol-HFIP, in kJ/mol.

III. THERMODYNAMICAL PROPERTIES OF THE NEAT SYSTEMS

Solvent	ΔH_{vap}^{exp}	ΔH_{vap}	$\Delta H_{vap}^{99\%}$	$\Delta H_{vap}^{95\%}$	$\Delta H_{vap}^{90\%}$
Acetone	31.27	32.65	32.69	32.84	33.03
HFIP	41.60	42.25	42.26	42.30	42.37
Methanol	37.60	40.14	40.15	40.18	40.22

TABLE XIII: Enthalpies of vaporization at 298.15 K for the neat substances calculated at experimental density, and with the decrease of 1%,5%, 10% in kJ/mol.

As can be seen in table in XIII, with small variations of the density for each system, the enthalpy of vaporization remains constant within 0.02-0.1 % (1 %), 0.1-0.5 % (5 %) and 0.3-1 (10 %) .

IV. ACTIVITY COEFFICIENTS OF MIXED SYSTEMS

For a binary mixture, we calculate the $\Delta_{\text{mix}}G$ as

$$\Delta_{\text{mix}}G = G_x - xG(1) - (1-x)G(2) \tag{1}$$

Where G_x is the Gibbs energy of the system at the x molar fraction of component 1, $G(1)$ is the Gibbs energy of component 1 and $G(2)$ is the one of component 2. From this, we define the excess Gibbs energy of mixing G^{e} as

$$G^{\text{e}} = \Delta_{\text{mix}}G - \Delta_{\text{mix}}G^{\text{id}}, \tag{2}$$

where $\Delta_{\text{mix}}G^{\text{id}}$ is the ideal Gibbs energy of mixing. The activity coefficients f_i are directly related to G^{e} by

$$f_i = \exp\left(\frac{1}{RT}\frac{\partial G^{\text{e}}}{\partial N_i}\right), \tag{3}$$

where R is the ideal gas constant, T is the temperature, and N_i is the particle number of component i. No analytical expression for G^{e} is available, so we calculate its derivative numerically through a Redlich–Kister (RK) style polynomial.[1]

x_{m}	G_{mix}	H_{mix}	S_{mix}	f_{HFIP}	f_{Acetone}
0.00	0.00	0.00	0.00	0.000	1.000
0.20	-7.85	-10.30	-8.22	0.011	0.110
0.50	-7.24	-11.96	-15.81	0.171	0.068
0.80	-6.97	-7.77	-2.68	0.153	0.017
1.00	0.00	0.00	0.00	1.000	0.000

TABLE XIV: Activity coefficients of HFIP and acetone, gibbs, enthapy and entropy of mixing for the system HFIP/acetone at 298.15 K increasing the molar fraction of HFIP.

x_{m}	G_{mix}	H_{mix}	S_{mix}	f_{HFIP}	f_{MeOH}
0.00	0.00	0.00	0.00	0.000	1.000
0.20	-1.38	-1.55	-0.55	0.000	300.720
0.50	-19.49	-21.27	-5.98	0.001	0.003
0.80	-3.77	-4.53	-2.54	124.264	0.000
1.00	0.00	0.00	0.00	1.000	0.000

TABLE XV: Activity coefficients of HFIP and methanol, gibbs, enthapy and entropy of mixing for the system HFIP/methanol at 298.15 K increasing the molar fraction of HFIP.

Via binary quantum cluster approach we are able to calculate the Gibbs energy of mixing G_{mix}, the enthalpy of mixing H_{mix}, and the entropy of mixing S_{mix}. As explained earlier the activity coefficients can be calculated from the excess Gibbs energy of mixing G^{e}. Since no experimental boiling point is present in literature for the mixture HFIP acetone and HFIP methanol, with the exception of the boiling point at 0.5 molar fraction of HFIP for the system

HFIP acetone, the isobars calculated via molecular dynamics are used as input for the calculations. Since there is not experimental value of activity coefficient nor the other properties in literature, our results are to be considered as qualitatively and not quantitatively. In tables XIV and XV the calculated G_{mix}, H_{mix}, S_{mix} and activity coefficients are shown for the systems HFIP acetone and HFIP methanol respectively. In both cases the strong negative values of G_{mix} means the systems are strongly interactive, and the activity coefficients shows strong interactions of one solvent within the other one.

V. CALCULATED BOILING POINT OF NEAT AND MIXED SYSTEMS

System	x_m	T_{bol}^{calc} (K)	T_{bol}^{exp} (K)
HFIP	1.0	342	331
MeOH	0.0	438	337
Ace	0.0	392	329
HFIP-ACE	0.2	385	–
HFIP-ACE	0.5	382	367
HFIP-ACE	0.8	369	–
HFIP-MeOH	0.2	338	–
HFIP-MeOH	0.5	449	–
HFIP-MeOH	0.8	349	–

TABLE XVI: Experimental and calculated boiling point of the neat and mixed systems

At each investigated temperature the Peacemaker code performs two full QCE iterations with either a gas-like or liquid-like initial volume guess, respectively. The iterations will typically converge to different solutions of the population and volume polynomial, one resembling the gas-phase state and one resembling the liquid-phase state. At each temperature, the Gibbs energies of gas and liquid phase are compared. When the Gibbs energy of the gas phase becomes lower than that of the liquid phase, the Newton-Raphson algorithm is used to find the temperature at which $G(g) - G(l) = 0$. This temperature is then treated as the QCE boiling point.

VI. CLUSTERS PICTURES

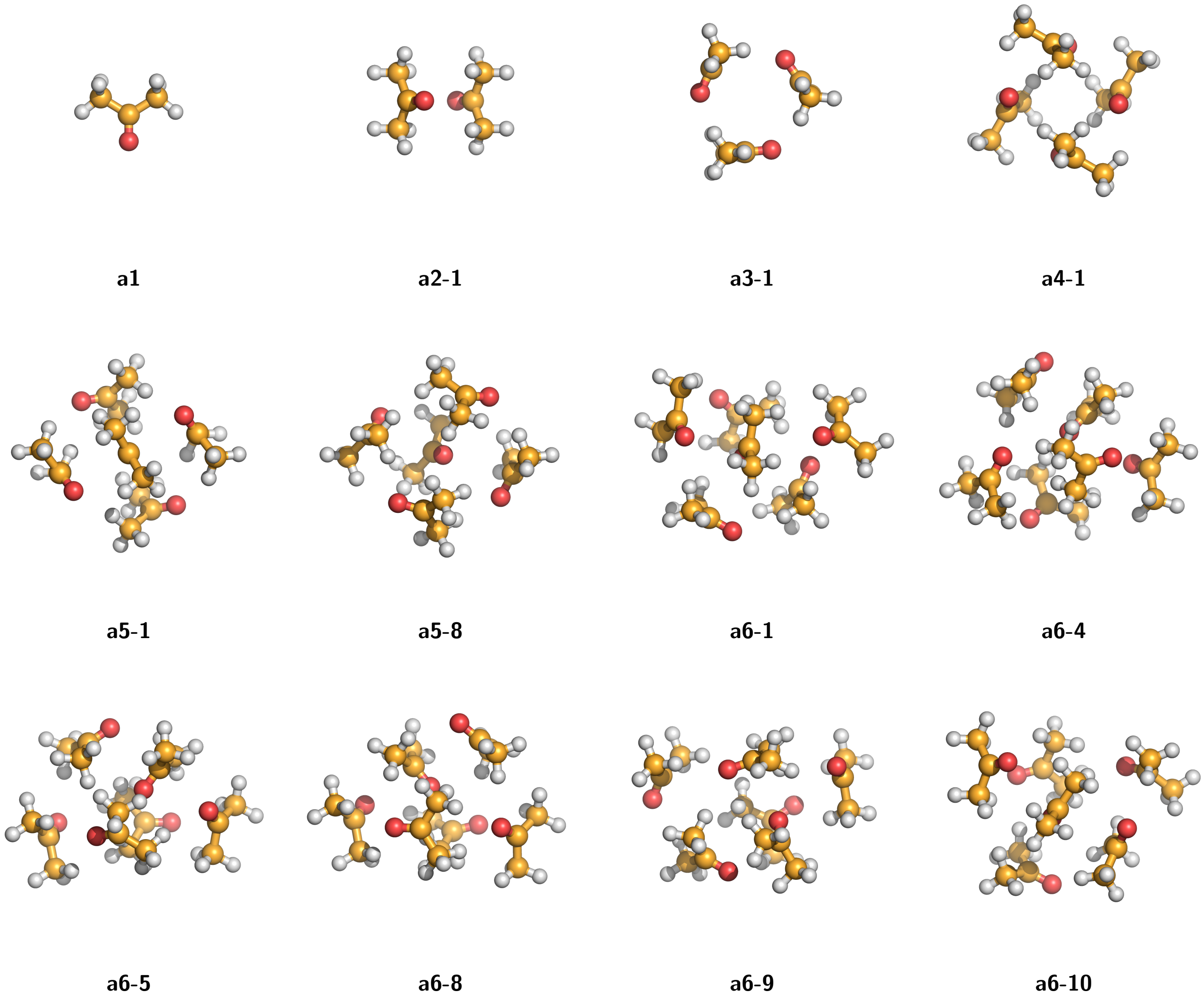

FIG. 12: Pure acetone clusters after BP86 optimization.

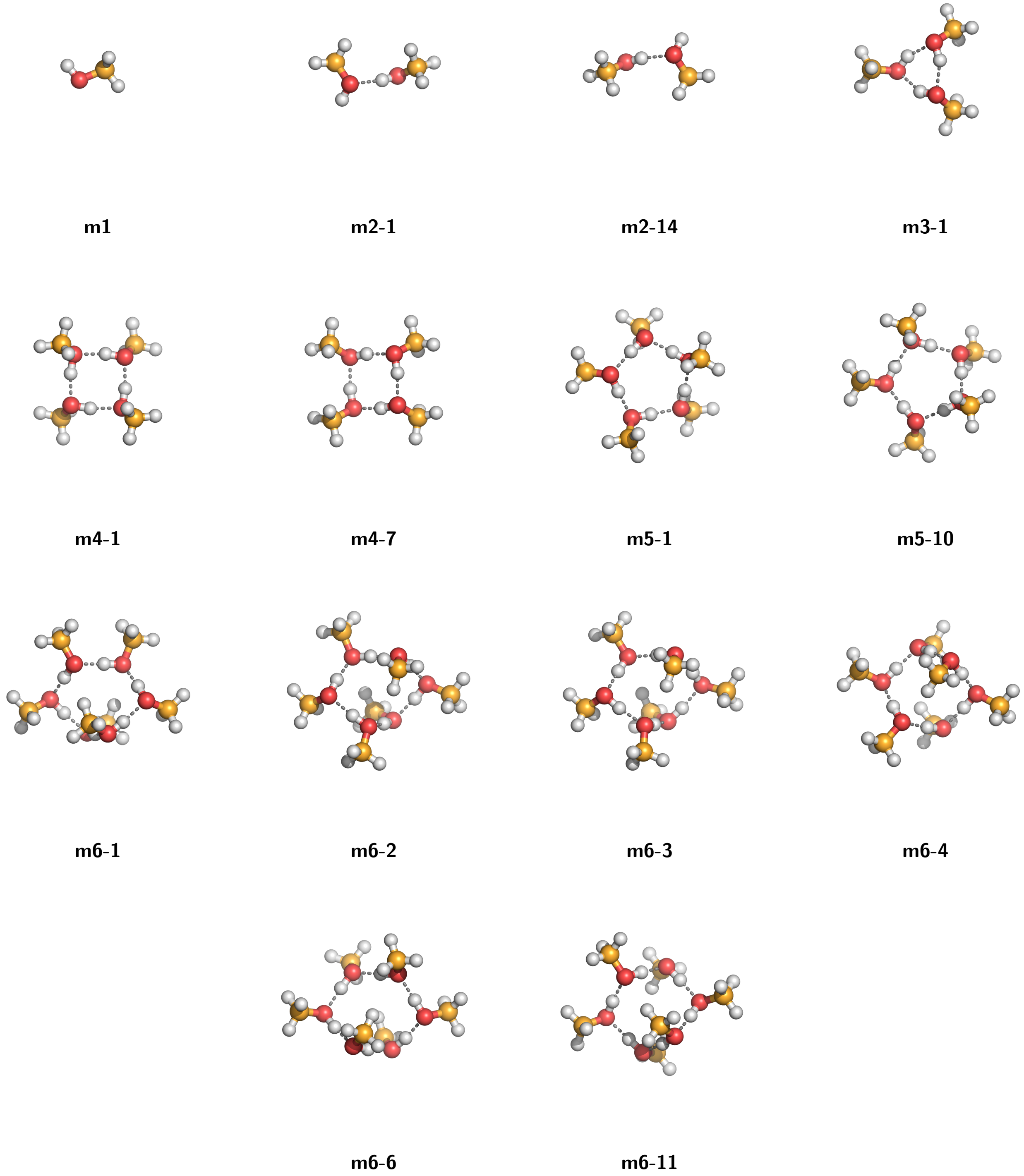

FIG. 13: Pure methanol clusters after BP86 optimization.

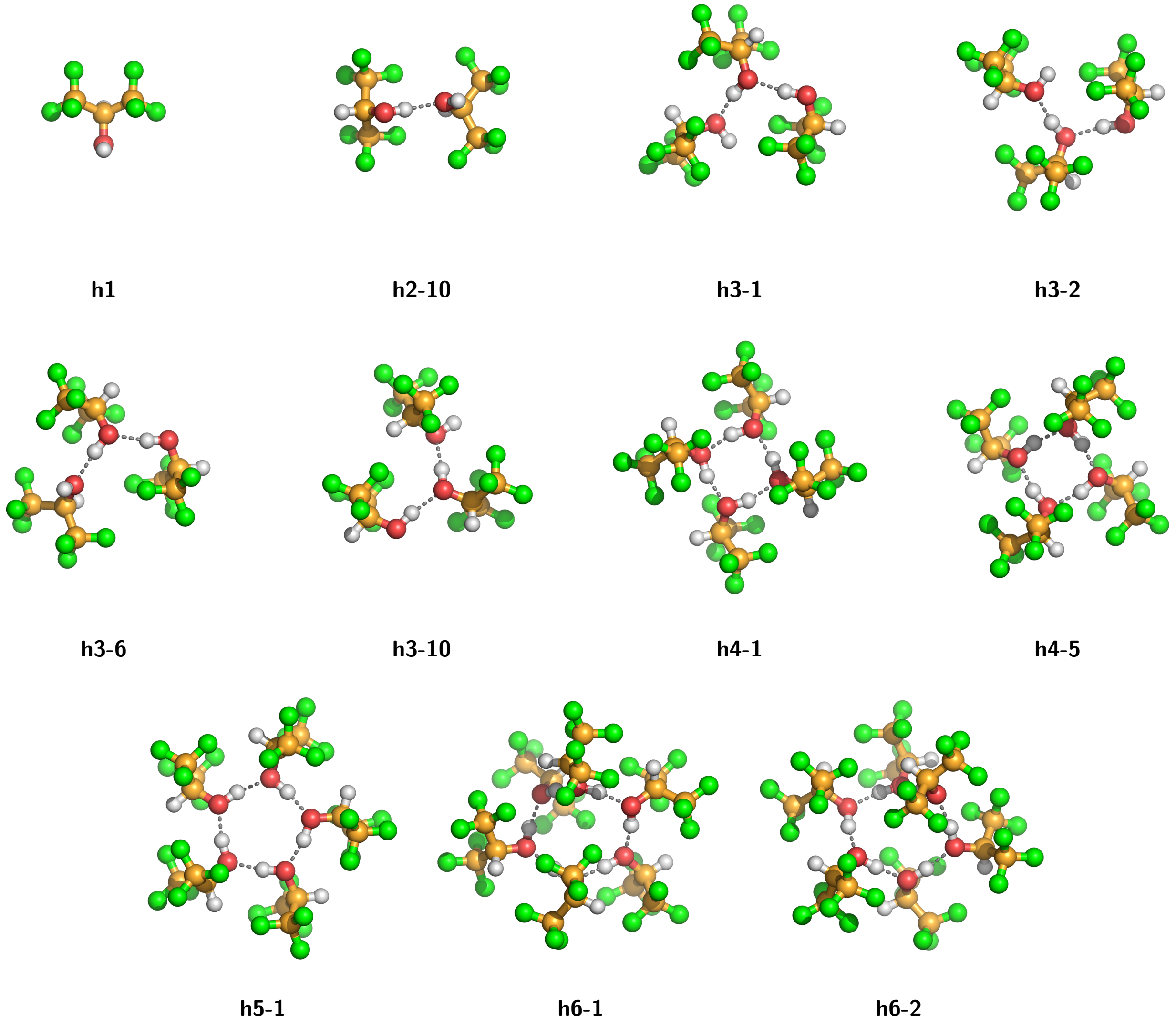

FIG. 14: Pure HFIP clusters after BP86 optimization.

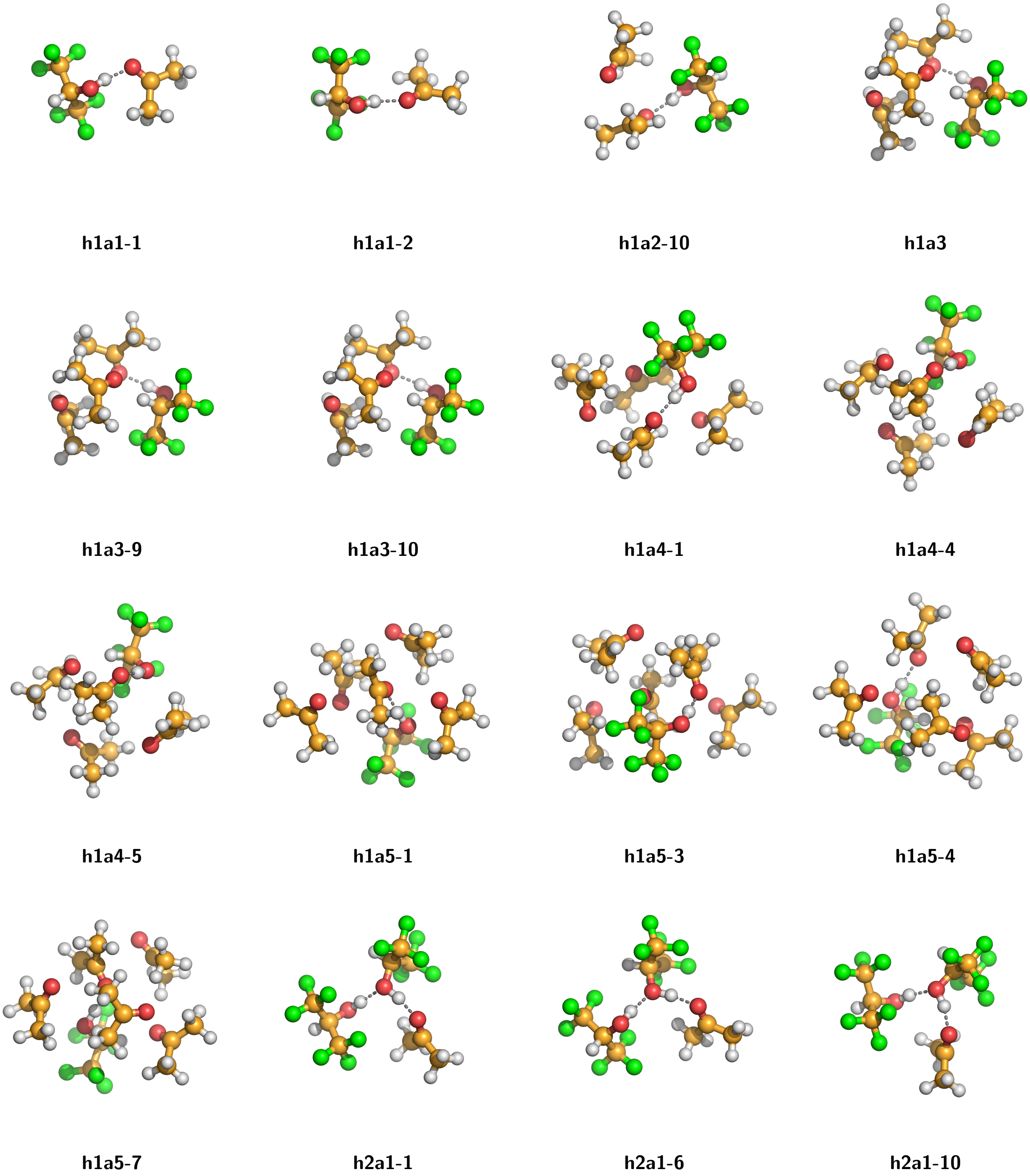

FIG. 15: HFIP/acetone clusters after BP86 optimization (1/3).

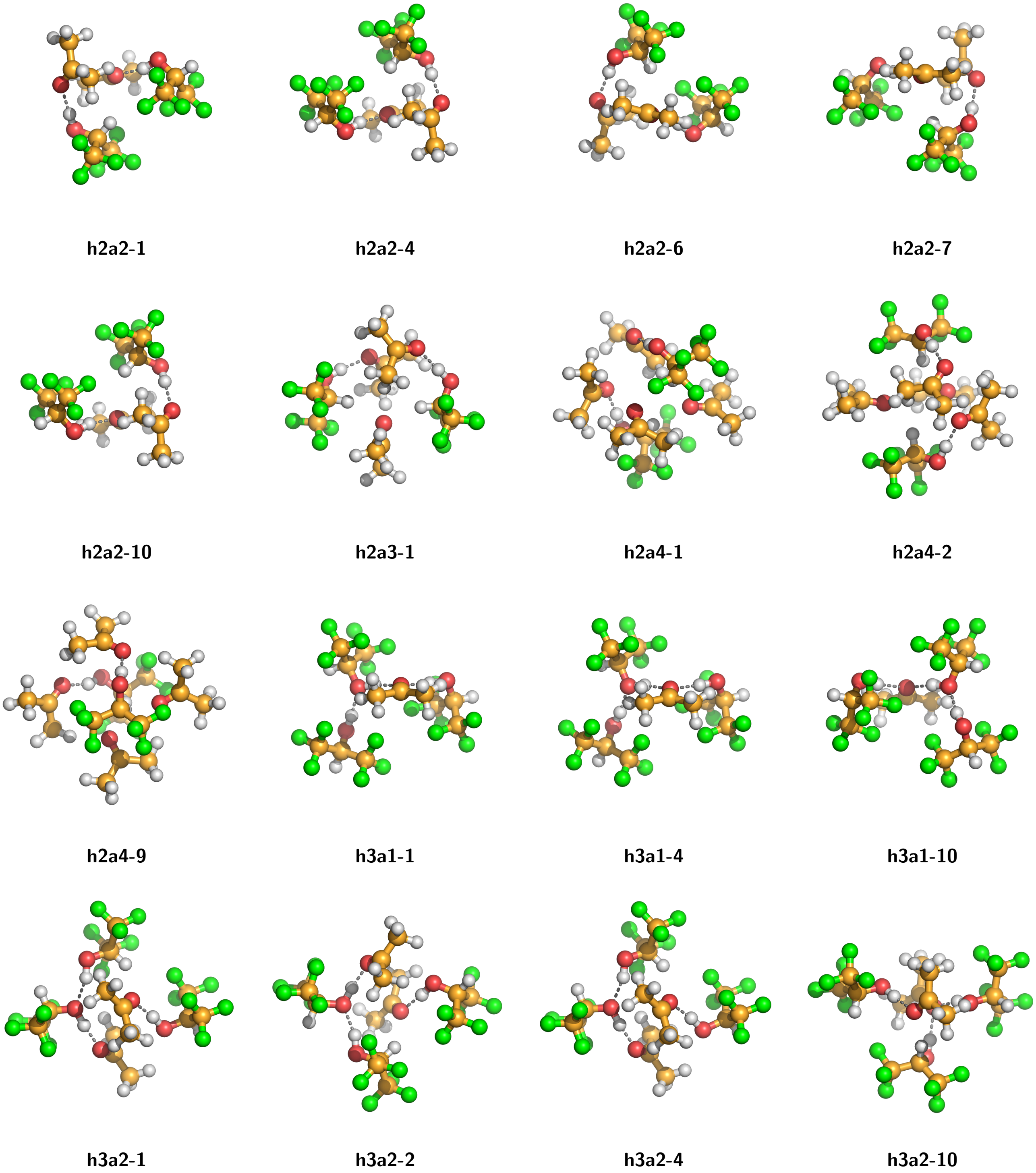

FIG. 16: HFIP/acetone clusters after BP86 optimization (2/3).

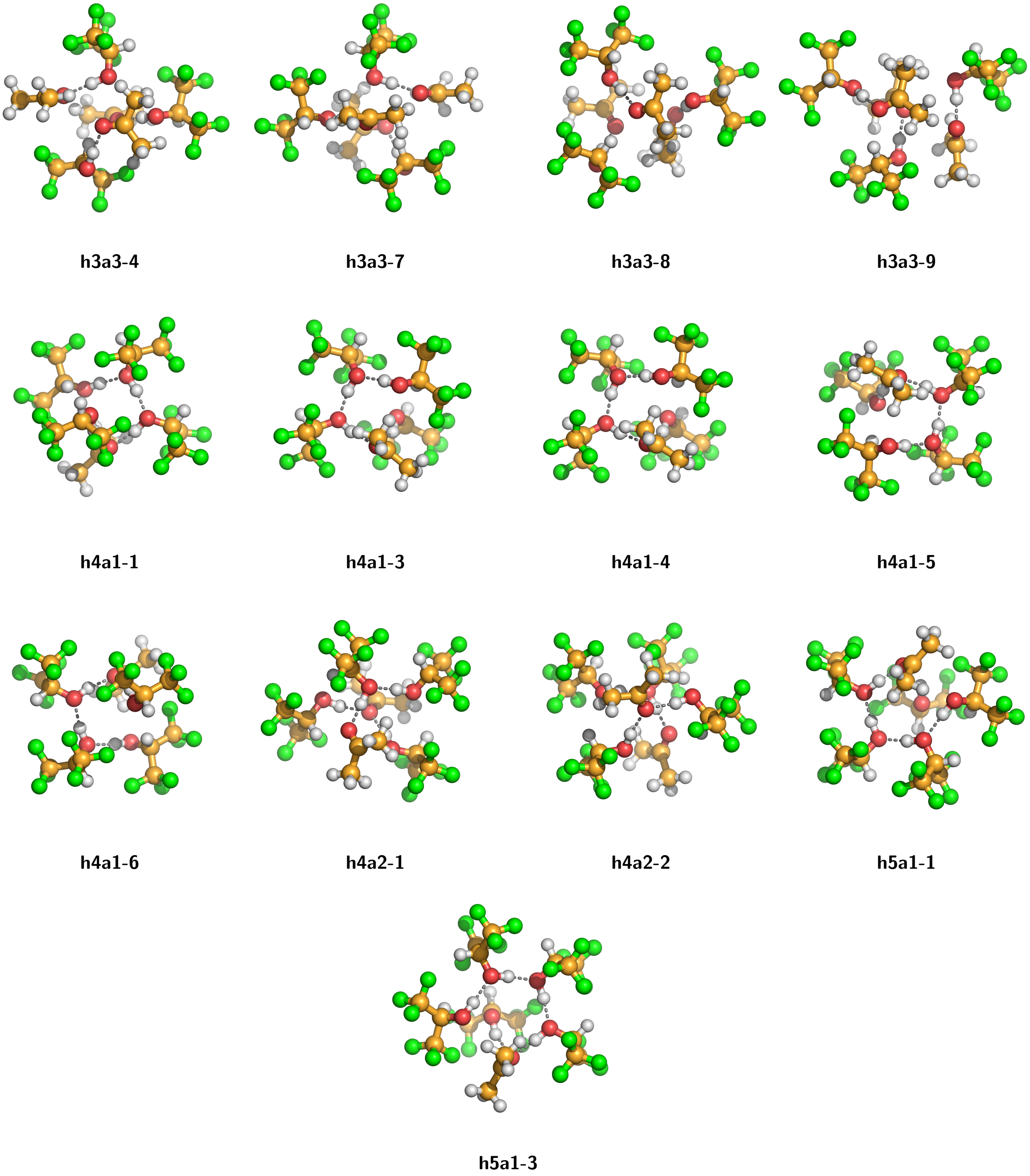

FIG. 17: HFIP/acetone clusters after BP86 optimization (3/3).

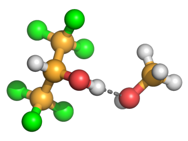

h1m1-1

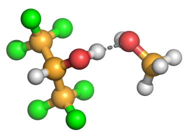

h1m1-3

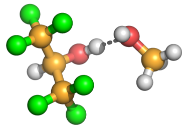

h1m1-6

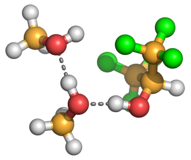

h1m2-1

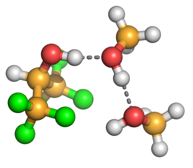

h1m2-2

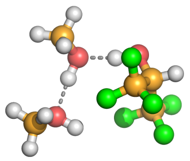

h1m2-6

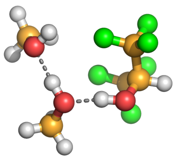

h1m2-7

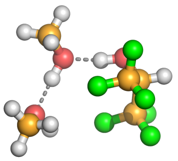

h1m2-8

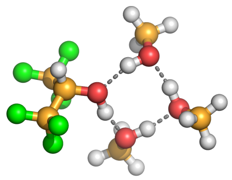

h1m3-1

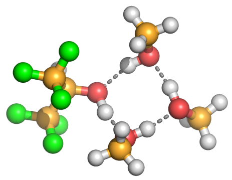

h1m3-2

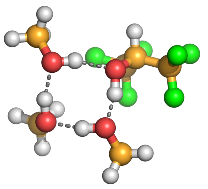

h1m3-3

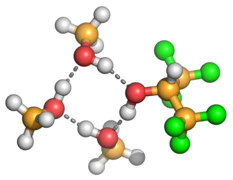

h1m3-4

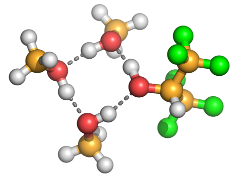

h1m3-5

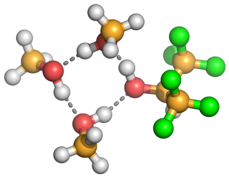

h1m3-6

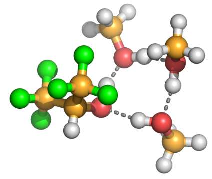

h1m3-7

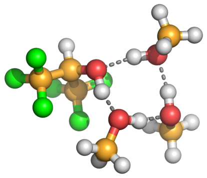

h1m3-8

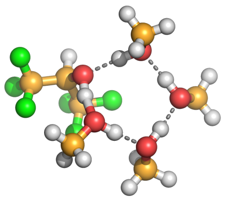

h1m4-1

FIG. 18: HFIP/methanol clusters after BP86 optimization (1/4).

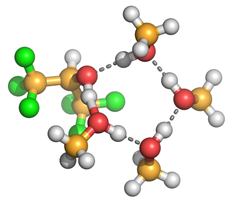

h1m4-2

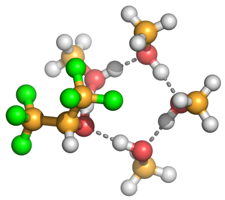

h1m4-4

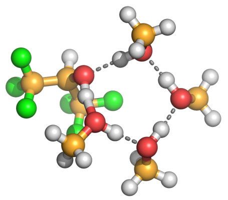

h1m4-5

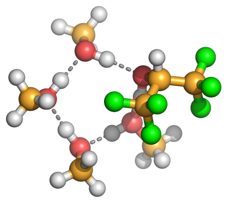

h1m4-6

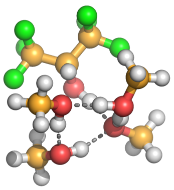

h1m4-7

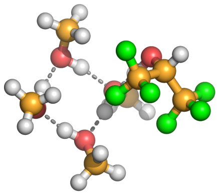

h1m4-8

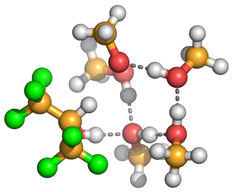

h1m5-1

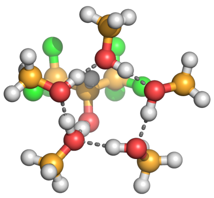

h1m5-2

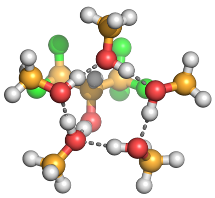

h1m5-3

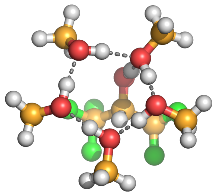

h1m5-6

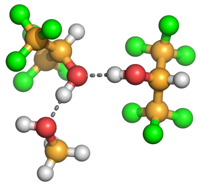

h2m1-1

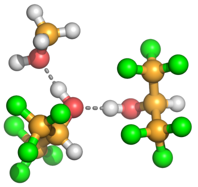

h2m1-6

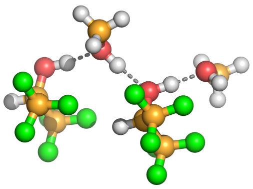

h2m2-1

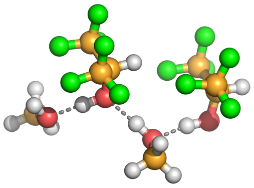

h2m2-3

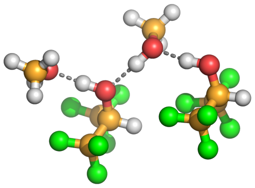

h2m2-5

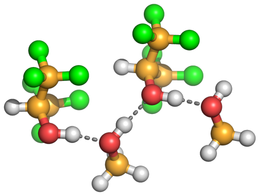

h2m2-6

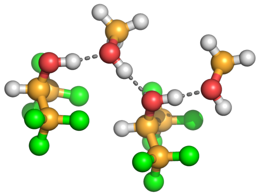

h2m2-7

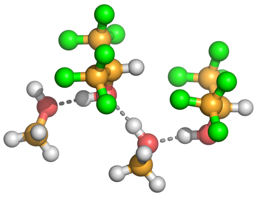

h2m2-8

FIG. 19: HFIP/methanol clusters after BP86 optimization (2/4).

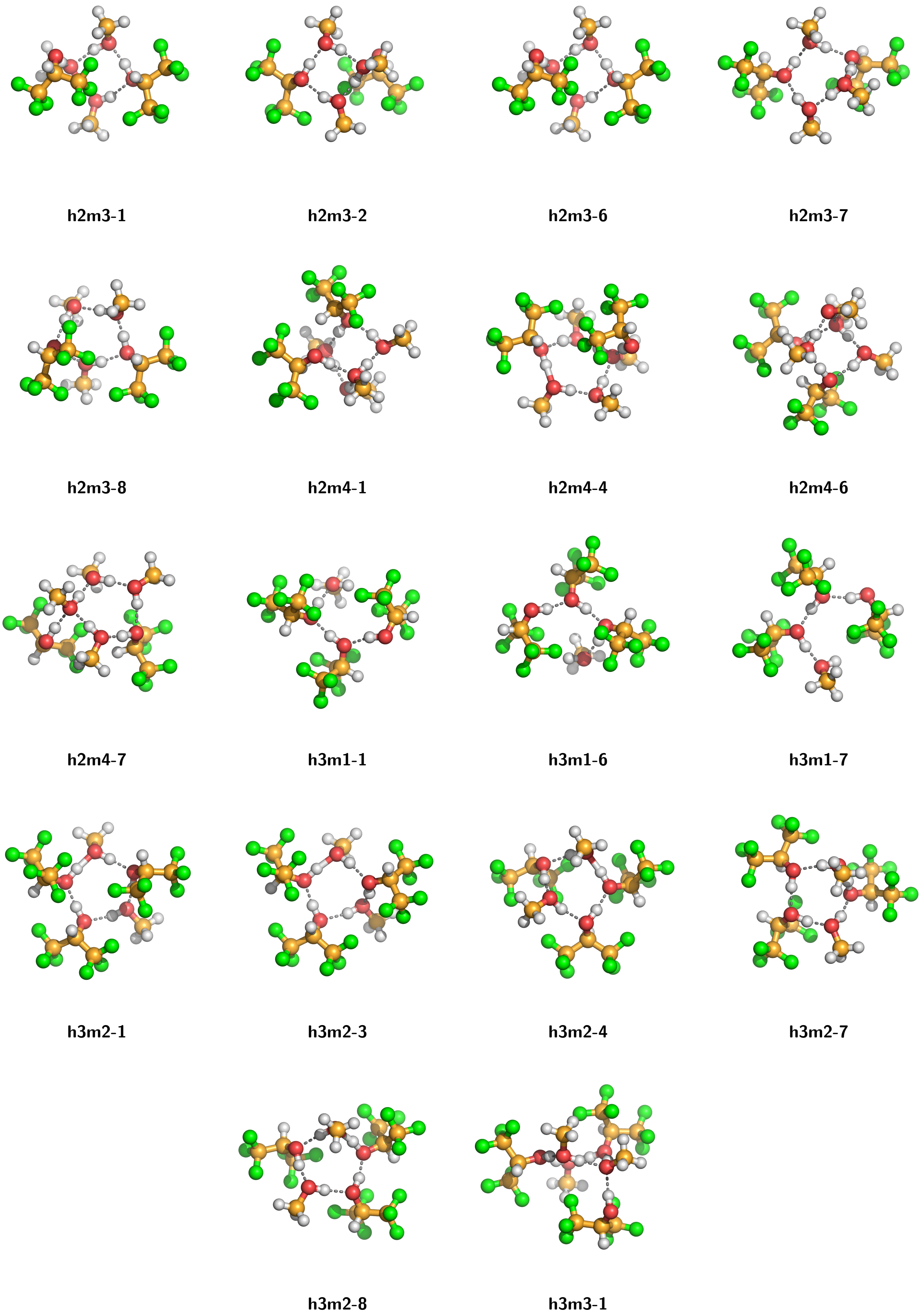

FIG. 20: HFIP/methanol clusters after BP86 optimization (3/4).

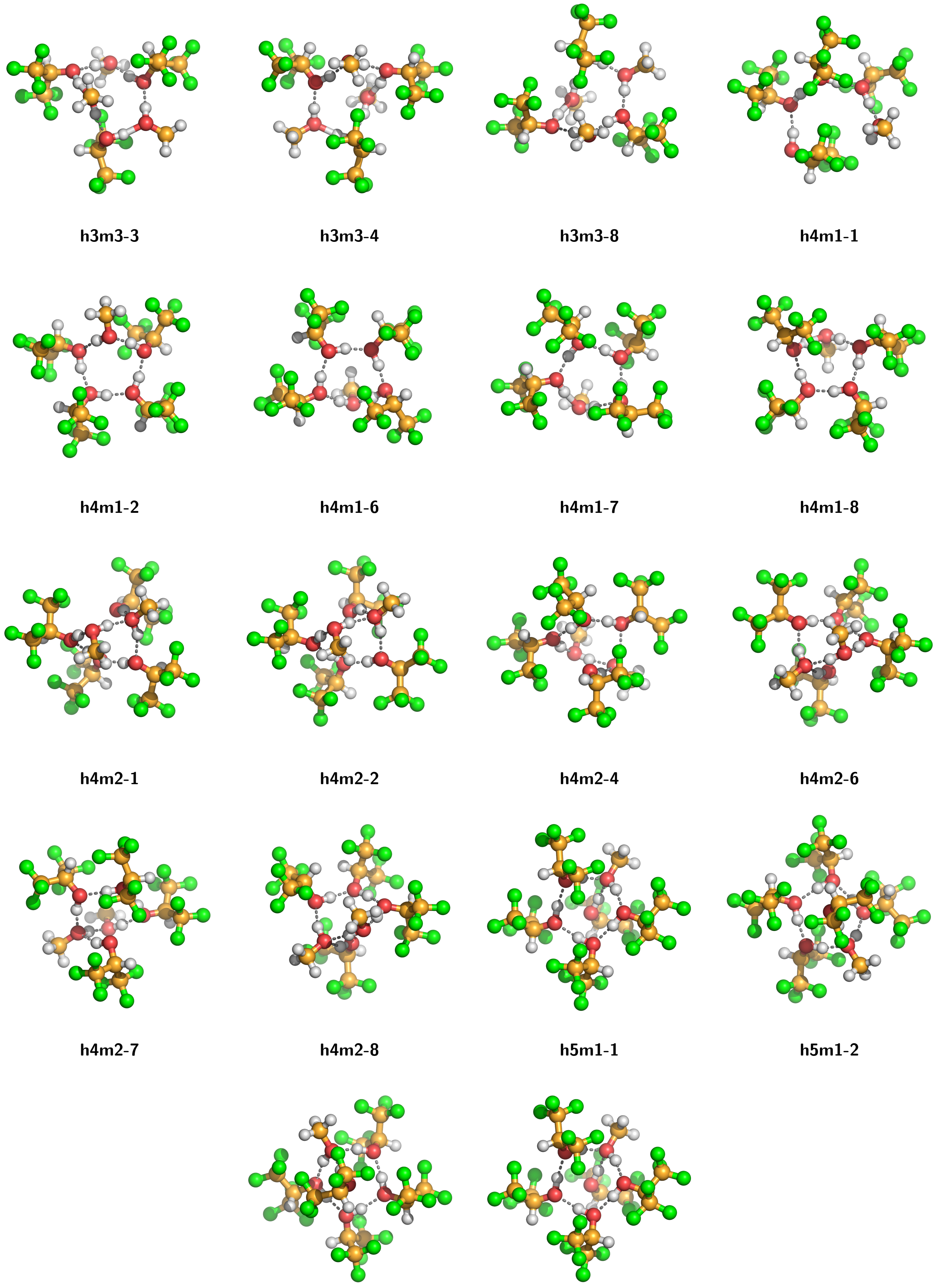

FIG. 21: HFIP/methanol clusters after BP86 optimization (4/4).

VII. CLUSTERS PICTURES

a1.xyz

```
10
-193.25001312668
C  -0.00000315  0.10046722  0.00013134
O  -0.00003155  1.32008046 -0.00003149
C   1.28930626 -0.69978147 -0.00116156
C  -1.28925614 -0.69984725  0.00117381
H  -2.14966520 -0.02898767  0.09496874
H  -1.29169905 -1.43393081  0.82105300
H  -1.37195229 -1.27346837 -0.93531358
H   2.14983496 -0.02855808 -0.09096213
H   1.29317098 -1.43056003 -0.82401846
H   1.37025162 -1.27730748  0.93306124
```

a2-1.xyz

```
20
-386.51022511688
C   1.59662589  0.00000000  0.25758712
O   1.11947469  0.00000000  1.38528677
C   1.88000278  1.28647280 -0.48556820
C   1.88000278 -1.28647280 -0.48556820
H   1.74215702 -2.14703500  0.17766354
H   1.17902714 -1.35833100 -1.33216485
H   2.89689506 -1.28805540 -0.90418815
H   1.74215702  2.14703500  0.17766354
H   2.89689506  1.28805540 -0.90418815
H   1.17902714  1.35833100 -1.33216485
C  -1.59662589  0.00000000 -0.25758712
O  -1.11947469 -0.00000000 -1.38528677
C  -1.88000278 -1.28647280  0.48556820
C  -1.88000278  1.28647280  0.48556820
H  -1.74215702  2.14703500 -0.17766354
H  -2.89689506  1.28805540  0.90418815
H  -1.17902714  1.35833100  1.33216485
H  -1.74215702 -2.14703500 -0.17766354
H  -1.17902714 -1.35833100  1.33216485
H  -2.89689506 -1.28805540  0.90418815
```

a3-1.xyz

```
30
-579.77045390463
C   0.36710758 -2.36108967 -0.00045063
O  -0.85435656 -2.26329385 -0.05207372
C   1.21153573 -2.43445162 -1.25015390
C   1.10818032 -2.37341102  1.31553593
H   0.40263800 -2.35976752  2.15317593
H   1.75830919 -3.25838410  1.38530242
H   1.76552969 -1.49009289  1.35447876
H   0.57513962 -2.48145491 -2.14027568
H   1.85491257 -1.54055081 -1.28683088
H   1.88036588 -3.30747867 -1.21686053
C  -2.23320514  0.86311336  0.00304127
O  -1.53617659  1.87221286  0.00737170
C  -2.65203877  0.18091233  1.28359922
C  -2.68303098  0.21460804 -1.28452193
H  -2.38727422  0.82486564 -2.14462361
H  -2.22215963 -0.78418876 -1.34911485
H  -3.77275459  0.06325263 -1.28488628
H  -2.30567186  0.75073616  2.15248830
H  -3.74579750  0.06602105  1.32046426
H  -2.22291130 -0.83388378  1.29452193
C   1.86630689  1.49813514 -0.00439598
O   2.39672061  0.39242847  0.01246170
C   1.50215103  2.18473014 -1.29933506
C   1.50664623  2.22609333  1.26880353
H   1.86136505  1.66862333  2.14230763
H   1.93360472  3.24022549  1.26966910
H   0.41136404  2.34064039  1.30664040
H   1.84884351  1.59635900 -2.15563212
H   0.40738957  2.30404490 -1.33507075
H   1.93452616  3.19584978 -1.33677524
```

a4-1.xyz

```
40
-773.03099294928
C  -0.15551459 -2.65906650 -0.51490930
O   0.47219319 -2.47908720  0.52262410
C   0.21177711 -1.97095000 -1.80590800
C  -1.35515459 -3.57126520 -0.55707190
H  -1.44190904 -4.12979070  0.38098650
H  -2.24946699 -2.94177070 -0.70076010
H  -1.29717901 -4.26280110 -1.41061490
H   1.05212486 -1.28894180 -1.65717750
H   0.46792162 -2.72038060 -2.57226080
H  -0.66368958 -1.41225330 -2.17350290
C   2.65981649 -0.16785080  0.51490930
O   2.47692352  0.46069860 -0.52262410
C   3.57758974 -1.37173510  0.55707190
C   1.96998884  0.20263680  1.80590800
H   1.28407521  1.04615720  1.65717750
H   1.41534713 -0.67024770  2.17350290
H   2.71823933  0.45530770  2.57226080
H   4.13652367 -1.46108130 -0.38098650
H   4.26886415 -1.31696670  1.41061490
H   2.95223689 -2.26313710  0.70076010
C  -2.65981649  0.16785080  0.51490930
O  -2.47692352 -0.46069860 -0.52262410
C  -3.57758974  1.37173510  0.55707190
C  -1.96998884 -0.20263680  1.80590800
H  -1.28407521 -1.04615720  1.65717750
H  -1.41534713  0.67024770  2.17350290
H  -2.71823933 -0.45530770  2.57226080
H  -4.13652367  1.46108130 -0.38098650
H  -4.26886415  1.31696670  1.41061490
H  -2.95223689  2.26313710  0.70076010
C   0.15551459  2.65906650 -0.51490930
O  -0.47219319  2.47908720  0.52262410
C  -0.21177711  1.97095000 -1.80590800
C   1.35515459  3.57126520 -0.55707190
H   1.44190904  4.12979070  0.38098650
H   2.24946699  2.94177070 -0.70076010
H   1.29717901  4.26280110 -1.41061490
H  -1.05212486  1.28894180 -1.65717750
H  -0.46792162  2.72038060 -2.57226080
H   0.66368958  1.41225330 -2.17350290
```

a5-1.xyz

50
-966.29375226518
C 0.00000000 -0.00000000 1.36444759
O 0.00000000 -0.00000000 0.13816169
C 0.67651009 1.08696783 2.16116819
C -0.67651009 -1.08696783 2.16116819
H -1.37744107 -1.65145674 1.53791659
H 0.11040727 -1.76669889 2.52556119
H -1.18691240 -0.67049711 3.04032529
H 1.37744107 1.65145674 1.53791659
H -0.11040727 1.76669889 2.52556119
H 1.18691240 0.67049711 3.04032529
C -3.14184005 1.00919481 0.52074549
O -2.48714705 1.81552822 1.17124499
C -3.99913144 -0.04422439 1.18368899
C -3.12021844 0.98613071 -0.98806551
H -2.53462781 1.82343534 -1.38071601
H -2.68595543 0.02517133 -1.30726771
H -4.14306425 1.01796279 -1.39240311
H -3.89558362 0.00579877 2.27270489
H -5.05438956 0.10545649 0.90678059
H -3.71330022 -1.03939152 0.81067679
C 3.14184005 -1.00919481 0.52074549
O 2.48714705 -1.81552822 1.17124499
C 3.99913144 0.04422439 1.18368899
C 3.12021844 -0.98613071 -0.98806551
H 2.53462781 -1.82343534 -1.38071601
H 2.68595543 -0.02517133 -1.30726771
H 4.14306425 -1.01796279 -1.39240311
H 3.89558362 -0.00579877 2.27270489
H 5.05438956 -0.10545649 0.90678059
H 3.71330022 1.03939152 0.81067679
C -1.10463284 -2.55627147 -1.17158891
O -2.24938342 -2.32287524 -0.80005321
C -0.18228831 -3.46888812 -0.40207661
C -0.53493833 -1.92571389 -2.42242191
H -1.31760216 -1.39769907 -2.97811911
H 0.24220052 -1.20985764 -2.11748141
H -0.05782144 -2.67808871 -3.06697871
H -0.72082547 -3.95208187 0.42013899
H 0.24850052 -4.23293993 -1.06659651
H 0.66324909 -2.88876191 0.00628399
C 1.10463284 2.55627147 -1.17158891
O 2.24938342 2.32287524 -0.80005321
C 0.18228831 3.46888812 -0.40207661
C 0.53493833 1.92571389 -2.42242191
H 1.31760216 1.39769907 -2.97811911
H -0.24220052 1.20985764 -2.11748141
H 0.05782144 2.67808871 -3.06697871
H -0.24850052 4.23293993 -1.06659651
H -0.66324909 2.88876191 0.00628399
H 0.72082547 3.95208187 0.42013899

a5-8.xyz
50
-966.29235142208
C 1.09141063 2.59867500 -0.96801880
O 2.16604037 2.62257178 -0.37814064
C 0.85306261 1.67794976 -2.14348201
C -0.07229734 3.45686093 -0.53842597
H 0.21550288 4.09940579 0.30063160
H -0.41680388 4.07705940 -1.38022833
H -0.92542272 2.82171378 -0.24346557
H 0.32226461 0.79564022 -1.75466962
H 0.22068687 2.14341944 -2.91160074
H 1.80333258 1.35051714 -2.57905070
C -0.33576567 0.17423517 1.61914981
O 0.10207808 -0.04020003 0.49586625
C -1.23927321 -0.81456434 2.31990076
C -0.00253887 1.43393627 2.38091705
H 0.80943437 1.98449145 1.89485353
H -0.90772394 2.06081623 2.40066378
H 0.25689728 1.20634069 3.42526337
H -1.66647276 -1.52161387 1.60145049
H -0.64174064 -1.37285424 3.05898170
H -2.03469827 -0.29497048 2.87077701
C 3.31972611 -0.85411859 0.32334992
O 2.78102280 -1.94730730 0.20341871
C 3.31649266 -0.09773636 1.63165202
C 4.02055312 -0.17494895 -0.82864336
H 3.93983699 -0.78060251 -1.73791079
H 3.58114485 0.82456752 -0.97776707
H 5.08241374 -0.01807351 -0.58229679
H 2.74851923 -0.64692570 2.38991265
H 4.34911213 0.05690523 1.98134712
H 2.88384949 0.90004161 1.46994064
C -0.75476393 -2.70684824 -0.84755570
O -1.93711449 -2.38357134 -0.78320674
C -0.10385121 -3.57014538 0.20330780
C 0.13126204 -2.27476302 -1.99057823
H -0.38510377 -1.54860264 -2.62750061
H 1.06798072 -1.85816823 -1.59460865
H 0.40546987 -3.15514566 -2.59354978
H -0.83550069 -3.87969677 0.95721959
H 0.35054267 -4.45730045 -0.26388806
H 0.72641207 -3.01187142 0.66309130
C -3.30332932 0.80084499 -0.12484271
O -2.80535379 1.65668778 0.59899486
C -4.28699932 -0.21551464 0.40579776
C -2.94079645 0.67277547 -1.58302704
H -2.27201136 1.48261379 -1.89053262
H -2.45430580 -0.30685452 -1.72412862
H -3.84483006 0.67012072 -2.21016644
H -4.48931470 -0.03758737 1.46722405
H -5.22640860 -0.17249757 -0.16589463
H -3.87137665 -1.22414975 0.25656009

a6-1.xyz
60
-1159.55483984343
C -0.15715051 -0.21393065 -1.12896979
O -0.39824777 0.18135075 0.00855247
C 0.00294039 0.73501572 -2.28903998
C 0.02265778 -1.67504685 -1.44643962
H -0.29136637 -2.30229199 -0.60634944
H 1.09060432 -1.83316952 -1.66965889
H -0.53825867 -1.93944955 -2.35327083
H -0.02179494 1.77549544 -1.95110952
H -0.81706968 0.55615375 -3.00120888
H 0.94655021 0.52288149 -2.81263844
C -1.90546486 3.01761547 -0.05795771
O -0.83235320 3.60866056 -0.06883686
C -2.47638787 2.44383545 1.22036794
C -2.71323276 2.79096922 -1.31101285
H -3.71790134 3.22638967 -1.19485044
H -2.85604112 1.70969985 -1.48010685

H	-2.21397471	3.24101047	-2.17561446
H	-1.98806024	2.88852193	2.09466839
H	-2.27383961	1.36212854	1.20911760
H	-3.56504449	2.57909981	1.28254780
C	4.01759465	-1.00579202	-1.12978576
O	3.20464591	-0.75013799	-2.00895443
C	5.20451213	-0.10886649	-0.85805177
C	3.89155781	-2.23063026	-0.25344044
H	3.07926652	-2.87526510	-0.60509835
H	3.67774403	-1.89767127	0.77264192
H	4.83556703	-2.79468580	-0.22743896
H	6.14254365	-0.67332515	-0.97303482
H	5.15834297	0.24225940	0.18286493
H	5.20128860	0.74642010	-1.54184626
C	-3.51387039	-1.38479984	-1.07956504
O	-3.14419120	-0.58460691	-1.93125527
C	-4.11771175	-0.94105089	0.23043646
C	-3.35677922	-2.87615042	-1.26332732
H	-2.92785661	-3.10056785	-2.24543691
H	-4.32987250	-3.37897425	-1.15620953
H	-2.70878083	-3.26659543	-0.46328028
H	-4.25253690	0.14487860	0.24676739
H	-3.44286165	-1.26066850	1.04149986
H	-5.08080750	-1.44349496	0.40505977
C	-0.69582324	-1.93420013	2.23219435
O	-1.56695591	-2.58363637	1.66417810
C	-1.03062302	-0.73542506	3.08925414
C	0.76791039	-2.27659788	2.11523189
H	0.91282954	-3.11466339	1.42564433
H	1.16508505	-2.54486564	3.10706243
H	1.33372075	-1.39456883	1.77715118
H	-2.11101882	-0.66756907	3.25709686
H	-0.68325372	0.16162969	2.55659502
H	-0.49887206	-0.77155122	4.05102024
C	2.22210276	1.54440936	1.07194226
O	2.68991158	0.42274167	1.24381227
C	2.43196860	2.30814453	-0.21344592
C	1.43221366	2.25151323	2.14312182
H	0.44802305	2.52927483	1.73736949
H	1.92829028	3.19934520	2.40616644
H	1.33587841	1.62328079	3.03488885
H	2.70934936	1.62748310	-1.02551648
H	3.25170330	3.02961314	-0.05965297
H	1.53673259	2.88866094	-0.47268572

a6-4.xyz

60

-1159.55309408218

C	-3.96910181	-0.61852003	0.34731268
O	-3.17898098	-0.44234546	1.26657522
C	-4.21501984	-1.98718169	-0.24523763
C	-4.73789301	0.52464145	-0.27116579
H	-4.69035835	1.41073586	0.37065943
H	-5.78402415	0.24980241	-0.46667607
H	-4.26934231	0.74911046	-1.24173431
H	-3.58882604	-2.73903383	0.24683791
H	-3.99357040	-1.95468540	-1.32203304
H	-5.27553045	-2.26147543	-0.13567835
C	3.34904055	-1.41409366	-0.81656408
O	2.82322294	-0.32172725	-0.99427122
C	4.33121385	-1.65393560	0.30576400
C	3.02428568	-2.60453703	-1.68790200
H	2.47381951	-2.28804119	-2.58010910
H	2.40400261	-3.29425918	-1.09395809
H	3.93404294	-3.15128761	-1.97434090
H	4.48355321	-0.73750773	0.88602283
H	5.29393515	-2.00378439	-0.09694353
H	3.93486047	-2.45171888	0.95174246
C	0.70084281	-2.44709678	1.81858311
O	1.45074059	-3.16959661	1.16839618
C	-0.74738681	-2.80409769	2.04803876
C	1.16590791	-1.13286209	2.39112741
H	2.25799546	-1.10471826	2.46864703
H	0.70297519	-0.92616438	3.36595407
H	0.83191536	-0.33841415	1.69702674
H	-1.00996652	-3.70712879	1.48578748
H	-1.40796475	-1.96632479	1.76956852
H	-0.91344109	-2.98263425	3.12254756
C	-0.95852310	1.78803437	0.62912496
O	-0.19087701	0.92884185	0.20490518
C	-1.14896342	2.03514489	2.10251843
C	-1.73289359	2.67549498	-0.31212034
H	-1.86591710	2.18608374	-1.28301780
H	-1.13857987	3.59544475	-0.44003307
H	-2.70257620	2.96934483	0.10989313
H	-0.38936049	1.50768602	2.68815804
H	-2.14535241	1.65722951	2.37950724
H	-1.11136601	3.11188194	2.31677325
C	-1.04916353	-0.51033507	-2.25454398
O	-2.21547140	-0.13071724	-2.25629362
C	-0.60171756	-1.72706057	-1.48459974
C	0.02001503	0.19092065	-3.05825567
H	-0.31547502	1.19054793	-3.35508167
H	0.21463553	-0.39817302	-3.97028841
H	0.96223950	0.23568305	-2.49451191
H	-1.43075182	-2.15968776	-0.91788245
H	0.21097238	-1.43870156	-0.80572149
H	-0.18926879	-2.48079524	-2.17286787
C	1.91478149	3.27745778	0.31626925
O	1.11964309	4.08652399	0.77876251
C	2.74219644	2.36973181	1.19653472
C	2.10028649	3.10492279	-1.17248730
H	1.61229149	3.91921450	-1.71898325
H	1.65019919	2.14057674	-1.45318189
H	3.16431552	3.03951320	-1.44071857
H	2.39231237	2.41871679	2.23325748
H	3.79627145	2.68875179	1.15409590
H	2.70544094	1.34138387	0.80827479

a6-5.xyz

60

-1159.55336750731

C	1.38998009	-1.70068451	0.48756237
O	0.66798265	-0.70938820	0.48833557
C	1.71029176	-2.45198194	1.75590754
C	1.99736742	-2.24412584	-0.78098889
H	2.07269979	-1.46352942	-1.54546765
H	2.97896596	-2.69835587	-0.59316074
H	1.32596751	-3.03870507	-1.14444483
H	1.03544654	-2.15462230	2.56503896
H	1.64926155	-3.53630083	1.59092323
H	2.74653042	-2.20758888	2.03856579
C	-1.94151483	-2.78732234	-0.11712839
O	-0.96056870	-3.50067766	-0.28957344
C	-2.94764402	-2.53234262	-1.21411378
C	-2.19001238	-2.09312763	1.20269194

H -1.59013581 -2.55870230 1.99207959
H -1.88249874 -1.03940266 1.10547577
H -3.25532392 -2.10118641 1.47180319
H -2.62045472 -3.00233610 -2.14777186
H -3.92475985 -2.94699797 -0.91830503
H -3.09784903 -1.45009077 -1.36278375
C 0.58947619 0.83051228 -2.27997765
O 1.78941883 0.81084319 -2.54119048
C -0.04192972 1.96076333 -1.51027589
C -0.33158680 -0.29114615 -2.68958098
H 0.13578840 -0.91464211 -3.45956006
H -0.51075004 -0.90429058 -1.79367194
H -1.30921099 0.08641546 -3.01642669
H 0.69689744 2.73655928 -1.28238454
H -0.87733969 2.38550881 -2.08492483
H -0.47285815 1.56757194 -0.57724726
C 4.09041466 0.76738418 -0.00772495
O 3.70545998 0.25664638 1.03756514
C 4.80331829 -0.03298267 -1.07333469
C 3.84122349 2.22100715 -0.33079481
H 4.76641365 2.71580475 -0.66055446
H 3.13562416 2.25385996 -1.17766912
H 3.41980718 2.74448340 0.53341746
H 4.98921081 -1.05509421 -0.72781433
H 4.17526447 -0.04904764 -1.97760248
H 5.75249595 0.44926494 -1.35047900
C -3.72862551 1.52138685 -0.59248350
O -3.39332030 0.88614629 -1.58491026
C -4.42976565 0.86690653 0.57532911
C -3.41724115 2.99288902 -0.44668720
H -3.07246133 3.40953140 -1.39889524
H -4.28932478 3.55214923 -0.07919348
H -2.62224834 3.09066594 0.30992461
H -4.70837286 -0.16257671 0.32750388
H -3.73781144 0.87187129 1.43249094
H -5.32155601 1.43858616 0.87052476
C -0.37102937 1.31341820 2.41343867
O -1.39743736 1.46389778 1.75833351
C 0.89015926 2.08101987 2.12366982
C -0.32048467 0.36382016 3.58888590
H -1.14387380 -0.35600377 3.53746374
H 0.64567610 -0.15434970 3.63502437
H -0.42052687 0.95083261 4.51671150
H 0.70360268 2.86958579 1.38749048
H 1.30414719 2.51155696 3.04802356
H 1.64826270 1.38067421 1.73526532

a6-8.xyz
60
-1159.55292381617
C 1.16606265 1.74609102 -0.44247569
O 0.42133211 0.78193663 -0.29676618
C 1.29641941 2.46310641 -1.76105333
C 1.97621476 2.29251773 0.70671328
H 2.18391726 1.50155522 1.43605087
H 2.90717304 2.76079181 0.36308042
H 1.36380739 3.07303589 1.18703495
H 0.54964932 2.10243981 -2.47510556
H 1.18662999 3.54636460 -1.61020691
H 2.30602483 2.27381242 -2.15678488
C -1.91706193 2.90689119 0.28359755
O -0.99945716 3.70495602 0.43113961
C -2.32867366 2.41178678 -1.08345663

C -2.68251508 2.34771486 1.46031979
H -2.22672179 2.67509505 2.40101372
H -3.72422048 2.70578022 1.41963092
H -2.72528459 1.24717245 1.41237796
H -1.94537419 1.38631739 -1.21090639
H -3.42212682 2.36604943 -1.18371169
H -1.90196298 3.05042872 -1.86433800
C 0.86334930 -1.01604769 2.60161054
O 2.05980822 -0.95251773 2.33604082
C 0.07232243 0.20331556 3.00136808
C 0.09122513 -2.30932326 2.51369412
H 0.68636958 -3.08684808 2.02234416
H -0.16326068 -2.63896262 3.53433900
H -0.86470695 -2.14350329 1.99261265
H 0.72375552 0.96354118 3.44620155
H -0.35346700 0.60980296 2.06985508
H -0.76330435 -0.04232082 3.66897229
C 3.96156558 -0.63725938 -0.51910261
O 3.47716844 -0.03040498 -1.46685780
C 3.73405410 -2.11631348 -0.31558208
C 4.78717506 0.05868614 0.53792909
H 4.95025957 1.10752594 0.26990966
H 4.25098319 -0.00687424 1.49741939
H 5.75279403 -0.45064009 0.67353573
H 3.22175049 -2.54879965 -1.18086712
H 4.68500191 -2.63919042 -0.13662831
H 3.12179988 -2.24237232 0.59264263
C -3.52193766 -1.55192984 0.27006893
O -2.81921094 -1.11711538 1.17476880
C -4.44157015 -0.65879269 -0.52736606
C -3.50685084 -3.01206037 -0.12060677
H -2.78524351 -3.56849401 0.48727884
H -4.50981056 -3.44699800 0.00941458
H -3.24926822 -3.09554028 -1.18648160
H -4.59330510 0.29575664 -0.01312453
H -3.95731696 -0.47700167 -1.49921665
H -5.40738122 -1.14511204 -0.72371015
C -0.50931489 -1.34956892 -2.19683989
O -1.70629439 -1.08891337 -2.25467383
C 0.47259441 -0.83969895 -3.22356723
C 0.06602163 -2.22056276 -1.10730207
H -0.72903205 -2.69785729 -0.52760596
H 0.74555445 -2.97787919 -1.52386194
H 0.65934936 -1.57371159 -0.44494309
H 0.02347038 -0.03504026 -3.81555451
H 1.40739100 -0.50933257 -2.74823352
H 0.73328394 -1.67146414 -3.89939517

a6-9.xyz
60
-1159.55161779602
C -4.24764319 0.96631280 -0.16438578
O -4.04509034 1.55297070 0.89154147
C -4.36639808 -0.53764810 -0.23544358
C -4.35351704 1.70301766 -1.48072343
H -4.27931350 2.78404460 -1.32169992
H -3.54991186 1.35713625 -2.14965426
H -5.30485122 1.46259771 -1.97917323
H -4.35788172 -0.96867426 0.77084059
H -5.28101186 -0.83328781 -0.77072921
H -3.51359587 -0.91972531 -0.81811432
C 4.08255394 1.67284014 -0.49958100
O 4.59557812 0.60636603 -0.81608897

C 3.15790683 2.42129070 -1.42974098
C 4.29527498 2.28428693 0.86629357
H 5.03390220 1.70769450 1.43333297
H 4.61971169 3.33195078 0.78014429
H 3.33079466 2.29071509 1.39842302
H 3.12775096 1.93856041 -2.41213719
H 2.15577511 2.41902182 -0.97097013
H 3.46565521 3.47244534 -1.53122315
C -0.24519747 2.11871168 1.02835201
O 0.94681379 1.81731624 1.03191860
C -0.96425265 2.46610036 -0.25107765
C -1.06346034 2.16647261 2.29064345
H -0.49002909 1.78730207 3.14278652
H -1.36154325 3.20980695 2.48312386
H -1.99921578 1.60480354 2.15313815
H -0.26735892 2.88789478 -0.98482547
H -1.37320400 1.53208119 -0.66678052
H -1.81028015 3.14214387 -0.07831095
C -1.17219979 -1.62248579 1.52196069
O -1.15684638 -0.75632395 0.65729827
C -0.56164036 -1.39576495 2.88518484
C -1.81695712 -2.96842452 1.29131736
H -2.43663075 -2.95232734 0.38918423
H -1.01063202 -3.70672067 1.16517304
H -2.41451684 -3.27793924 2.16101163
H 0.09764418 -0.52278282 2.86635052
H -1.37165506 -1.21974885 3.61171094
H -0.01039431 -2.28636855 3.21464243
C -0.76521060 -0.90685670 -2.30522685
O -1.81053784 -0.30861259 -2.53131626
C 0.53768497 -0.17018254 -2.09739905
C -0.70520376 -2.41243808 -2.22268199
H -1.71458385 -2.83757877 -2.21590113
H -0.15934793 -2.79597216 -3.10031956
H -0.14025393 -2.73045875 -1.33378832
H 0.48649752 0.82759470 -2.54551796
H 0.69039522 -0.05576105 -1.01251224
H 1.39526585 -0.72707196 -2.49653789
C 2.34529827 -2.22456560 0.40268689
O 1.33544741 -2.90099760 0.57441294
C 2.76187072 -1.16861222 1.39933592
C 3.22038122 -2.40004297 -0.81231249
H 2.72947290 -3.03846324 -1.55501277
H 3.50763953 -1.42580887 -1.23486570
H 4.16422134 -2.87395726 -0.49562434
H 2.52589266 -1.49803105 2.41820559
H 3.82123204 -0.90276583 1.30464968
H 2.17163594 -0.25729820 1.20161056

a6-10.xyz
60
-1159.55253472025
C 2.56388604 -1.71139141 0.66699971
O 2.97350793 -0.75061309 1.31235297
C 2.11808657 -2.98547327 1.34109855
C 2.49167636 -1.69018902 -0.84045491
H 2.56937075 -0.66349727 -1.21343817
H 1.56678252 -2.17067006 -1.18875256
H 3.32858172 -2.28426040 -1.24367005
H 2.21223467 -2.89961999 2.42900441
H 2.72503775 -3.83032895 0.97927500
H 1.08405027 -3.21779147 1.04456733
C -1.37580209 -3.21144807 -0.94071721
O -0.25068484 -3.69241774 -0.89186920
C -1.84934540 -2.39804144 -2.11846394
C -2.37126609 -3.41199004 0.18111916
H -1.89481020 -3.91705562 1.02829089
H -3.21695996 -4.01990021 -0.17712290
H -2.78003958 -2.44221816 0.49530190
H -1.14019080 -2.48441929 -2.94882554
H -1.92791137 -1.34014060 -1.82017920
H -2.85253315 -2.71242202 -2.44203679
C -0.26555071 -0.11661337 1.46264722
O -0.48957325 -0.56722220 0.34368696
C 0.44679739 1.19311790 1.66790571
C -0.71211803 -0.83877947 2.71191485
H -1.23365183 -1.76776313 2.45915702
H -1.37478991 -0.17626451 3.28864895
H 0.16027307 -1.06206179 3.34412017
H 0.96695925 1.49411754 0.75389536
H 1.15770421 1.12458862 2.50156606
H -0.31059763 1.94745996 1.93368075
C 3.70566526 1.87214822 -0.30210910
O 2.71162196 1.98819032 -1.00866495
C 4.91908748 1.09533173 -0.76011562
C 3.78185962 2.47800095 1.07924733
H 2.94912554 3.16950546 1.24423835
H 4.74153042 2.99070951 1.23765452
H 3.72407255 1.65446788 1.80741385
H 4.75844459 0.68977851 -1.76445247
H 5.10814633 0.27690177 -0.05005536
H 5.80931880 1.74270406 -0.75765448
C -3.55237223 1.63566282 1.12278724
O -2.74615989 1.88163606 2.01186212
C -4.18758935 2.72713625 0.29455059
C -3.92744443 0.21738666 0.76150830
H -3.58794742 -0.47562866 1.53831646
H -3.42911876 -0.01986586 -0.19148474
H -5.00939244 0.11299624 0.59843667
H -3.84196042 3.71174487 0.62767479
H -5.28467161 2.67228845 0.36352692
H -3.91737760 2.56206604 -0.76033563
C -1.08265501 1.54320198 -2.01920376
O -2.24055129 1.14082471 -1.95825210
C -0.01254851 0.77310679 -2.74816875
C -0.65271865 2.83706879 -1.36772474
H -1.32374483 3.10093889 -0.54325435
H -0.70517045 3.63257432 -2.13013149
H 0.39109209 2.78674320 -1.03047972
H -0.45483599 0.09576262 -3.48645369
H 0.51631858 0.17844378 -1.98675948
H 0.73216188 1.43565447 -3.20743289

h1.xyz
12
-790.21447533505
C 1.29870280 0.12594203 -0.05188515
C -1.29870280 0.12594203 -0.05188515
C 0.00000000 -0.56191478 -0.53531232
F 1.35206010 1.43338085 -0.38505113
F 2.36554180 -0.48772254 -0.61230846
F 1.43213910 0.02760572 1.29773278
F -1.35206010 1.43338085 -0.38505113
F -1.43213910 0.02760572 1.29773278
F -2.36554180 -0.48772254 -0.61230846
H 0.00000000 -1.98331178 0.79340452

H -0.00000000 -0.50926024 -1.63333088
O 0.00000000 -1.92162101 -0.18066960

h2-10.xyz
24
-1580.43853247044
C -2.43937385 1.29407749 0.08228492
C -2.58276659 -1.27335955 -0.30706775
C -1.68392899 -0.05230822 -0.00346234
F -3.11822731 1.57347759 -1.04757249
F -1.55542747 2.29114224 0.29991452
F -3.31860379 1.29281702 1.12049387
F -3.31558981 -1.10355234 -1.42585749
F -3.43182556 -1.51990396 0.72601729
F -1.81177318 -2.36877671 -0.47206923
H -1.57653608 -0.33550722 1.93692427
H -0.95403928 0.03022485 -0.81925229
O -0.95587227 -0.27823043 1.18578410
C 2.48648590 1.28187818 -0.29167506
C 2.62830332 -1.27381489 0.09663016
C 2.66211111 0.13603029 0.73417941
F 3.40575506 1.23898913 -1.28165127
F 2.60805672 2.47147506 0.33498856
F 1.25205159 1.25482102 -0.87298869
F 3.58896086 -1.43848868 -0.84103836
F 1.42720463 -1.53452932 -0.48849439
F 2.82223944 -2.20687153 1.05448791
H 0.82942127 0.09287046 1.45373219
H 3.67085325 0.25764325 1.15760340
O 1.74633929 0.24958323 1.78209577

h3-1.xyz
36
-2370.66718055721
C 3.29221899 -0.42975385 1.03116959
C 2.85451244 -1.51038100 -1.27698958
C 3.47565661 -0.32252919 -0.50295393
F 3.82195901 -1.56751971 1.53644869
F 3.89664051 0.61251687 1.63489587
F 1.97364867 -0.39783600 1.38830588
F 3.34098887 -2.70615065 -0.87715496
F 1.49336604 -1.55177912 -1.12881105
F 3.10536872 -1.37851492 -2.59415064
H 2.04669913 0.99320471 -0.86371243
H 4.56088350 -0.37992919 -0.68144992
O 3.02630739 0.90060493 -0.99476333
C -2.26888936 -1.89471629 -1.05716246
C -2.51332211 -2.15084480 1.51919799
C -2.16168907 -1.22430196 0.33260113
F -3.52965653 -2.29272327 -1.32395385
F -1.90000565 -1.00626647 -2.00690336
F -1.44907449 -2.96977570 -1.15569511
F -3.69614920 -2.77413331 1.34855583
F -1.55371394 -3.09568472 1.69439668
F -2.58175999 -1.42038719 2.65316801
H -0.20433915 -1.38780742 0.54058514
H -2.87374825 -0.38884836 0.34394517
O -0.87471494 -0.67551578 0.53044862
C -1.56551391 2.87326636 -1.10114047
C 0.01007390 3.07892960 0.95274454
C -0.13597659 2.69329727 -0.53938504
F -2.03671754 4.12336780 -0.91558847
F -1.56484096 2.61795046 -2.42630385
F -2.44227226 2.00617614 -0.51937598
F -0.30581970 4.37532743 1.16895928
F -0.77153127 2.31332791 1.75206310
F 1.29408894 2.89546852 1.33332364
H -0.23082834 0.71623170 -0.28825903
H 0.51085937 3.38082582 -1.10385042
O 0.31750095 1.38670480 -0.77946941

h3-2.xyz
36
-2370.66717111174
C 1.65147662 -2.79790458 -1.15786627
C 0.24822240 -3.02219987 1.01551107
C 0.25877175 -2.68352178 -0.49488729
F 2.21500017 -4.00801982 -0.96393806
F 1.53551360 -2.59790770 -2.48732798
F 2.51131558 -1.85533795 -0.67700035
F 0.65261578 -4.29078225 1.24751432
F 1.04442392 -2.18731814 1.72722442
F -1.01100889 -2.89425830 1.48931402
H 0.23131778 -0.69807552 -0.29504711
H -0.38321053 -3.42952232 -0.98586503
O -0.29220640 -1.41676428 -0.74309742
C 2.17709709 2.03236695 -1.08190415
C 2.43216905 2.21955088 1.50081614
C 2.09212066 1.32164441 0.28890096
F 3.42253961 2.48131287 -1.33748476
F 1.83780499 1.15851100 -2.05584988
F 1.31958333 3.08071037 -1.15078799
F 3.61750533 2.84485186 1.35459871
F 1.47268474 3.16079229 1.69212822
F 2.49109140 1.46085938 2.61662838
H 0.12907484 1.43577572 0.48827150
H 2.82083855 0.50075134 0.27374859
O 0.81757288 0.74146277 0.47488005
C -3.31401956 0.32893618 1.08492801
C -2.99680696 1.28218459 -1.29816264
C -3.53207695 0.11534047 -0.43342914
F -3.89287751 1.46585362 1.53521563
F -3.84286619 -0.70478933 1.76922026
F -1.98675973 0.39069901 1.40403485
F -3.53722481 2.47381266 -0.95925120
F -1.63723128 1.40486630 -1.19381419
F -3.27603214 1.05302956 -2.59637543
H -2.04237016 -1.13861655 -0.76572675
H -4.62324456 0.10410844 -0.58229740
O -3.03015569 -1.11159482 -0.86079223

h3-6.xyz
36
-2370.66483024341
C -2.25603897 2.66238840 -0.40877403
C 0.19548397 3.24681677 0.22658449
C -0.81604584 2.14528425 -0.17427825
F -2.30468052 3.61741945 -1.35840261
F -3.04515190 1.64099345 -0.80104704
F -2.78111181 3.17021228 0.73780989
F 0.20894959 4.27119319 -0.64891761
F -0.09980606 3.74190804 1.45901760

F 1.43642433 2.72512336 0.28269392
H -1.11737532 1.49085228 1.65515855
H -0.46573537 1.69850595 -1.11378679
O -0.82001972 1.12258302 0.80158770
C 2.80695892 -0.65971329 -1.34906653
C 3.44590406 -0.53269271 1.16324119
C 3.20190025 -1.43631878 -0.06987739
F 3.71279596 0.28358488 -1.69079792
F 2.69895877 -1.52040888 -2.38426929
F 1.59831971 -0.03962968 -1.21098857
F 4.35395255 0.44242200 0.92736595
F 2.29760880 0.05908217 1.58479609
F 3.91143495 -1.28800680 2.18451753
H 1.39566830 -2.03546116 0.42261802
H 4.16671794 -1.91694507 -0.29381560
O 2.27789611 -2.44024382 0.22022572
C -2.58781805 -2.29574770 1.20306642
C -1.56417882 -2.27402802 -1.18732569
C -1.31962913 -2.44868039 0.33077335
F -3.52433112 -3.21807941 0.89502522
F -2.25596679 -2.45909676 2.50448391
F -3.14576607 -1.06575166 1.07453592
F -2.57090797 -3.05085609 -1.63965214
F -1.85360322 -0.98444211 -1.49830890
F -0.44111620 -2.61237925 -1.85928347
H -0.56149696 -0.63695991 0.67286731
H -0.96739614 -3.48054777 0.47466079
O -0.30079455 -1.58853700 0.77471465

h3-10.xyz
36
-2370.66689465234
C 1.67023995 2.76109462 1.35921452
C 0.72332500 2.78270370 -1.06427695
C 0.99124492 1.96613309 0.22065932
F 2.81020705 3.35537295 0.95512750
F 1.96747029 1.92528315 2.37573187
F 0.83683906 3.71964632 1.84410887
F 1.85262945 3.29824552 -1.58547046
F -0.14329617 3.80176100 -0.82358826
F 0.15895663 1.97855164 -1.99197043
H -0.84335184 2.10690825 0.92291814
H 1.65818685 1.13660304 -0.04784001
O -0.21328491 1.39509402 0.69804062
C 1.93887098 -2.46265939 1.02316995
C 2.30993902 -2.07757432 -1.50944891
C 1.65475542 -2.95643859 -0.41622960
F 3.26222303 -2.39660075 1.29711474
F 1.37487571 -3.30675222 1.91173108
F 1.40947312 -1.22510560 1.24497176
F 3.64527549 -1.94085506 -1.34275433
F 1.76925069 -0.82298301 -1.53681597
F 2.09859962 -2.62933080 -2.72200602
H -0.17625174 -2.24128897 -0.56301759
H 2.12870666 -3.94706030 -0.49743163
O 0.28909651 -3.11387354 -0.64483798
C -3.40714596 0.15572344 -1.02573320
C -2.98509382 -1.15780328 1.17381277
C -2.63495833 -0.98690695 -0.32419679
F -4.74426665 0.01295134 -0.91068718
F -3.09870560 0.16715195 -2.33921118
F -3.07132494 1.37368961 -0.51591207
F -4.29583212 -1.42472710 1.35970572
F -2.67574515 -0.04703753 1.89379396
F -2.27275645 -2.18561737 1.68263185
H -0.92375498 -0.00555901 -0.05814413
H -2.93575347 -1.91984007 -0.82322460
O -1.25262357 -0.82969297 -0.50912634

h4-1.xyz
48
-3160.90702996118
C -0.12764466 -4.12991844 -0.03489137
C -0.65248654 -2.75324330 2.10193749
C -0.89768889 -2.94146309 0.58474603
F -0.31099821 -5.27387296 0.65633162
F -0.55197963 -4.33116228 -1.30092965
F 1.20903060 -3.87975792 -0.08685412
F -1.13150485 -3.79746833 2.81315997
F 0.65968052 -2.60444583 2.39567245
F -1.29727752 -1.63707186 2.51687766
H 0.35286939 -1.50701746 -0.06495876
H -1.96853750 -3.15971210 0.46663788
O -0.61681340 -1.76866922 -0.13618888
C 0.14059762 4.11629267 -0.01833259
C 0.62749536 2.74004822 2.12830438
C 0.89499950 2.92477776 0.61480907
F 0.32829961 5.26272479 0.66771785
F 0.57801569 4.30622749 -1.28149378
F -1.19770509 3.87816224 -0.08050806
F 1.09100047 3.78770133 2.84451578
F -0.68883168 2.58686679 2.40123894
F 1.26967474 1.62736480 2.55620951
H -0.35309679 1.49008046 -0.04030870
H 1.96864101 3.13675037 0.51101070
O 0.61701527 1.75196114 -0.10683320
C 2.82451020 -0.61642180 -2.13635540
C 4.11832757 -0.13253763 0.06190475
C 2.95544467 -0.89118785 -0.61840626
F 3.90077174 -1.06923722 -2.81631319
F 1.73242939 -1.26326012 -2.60832173
F 2.67288324 0.70050232 -2.40745626
F 5.28935521 -0.30303445 -0.58589564
F 3.86564433 1.20316807 0.12776164
F 4.26880869 -0.57932613 1.32706705
H 1.49460636 0.34655802 -0.00083726
H 3.17037442 -1.96411151 -0.51268657
O 1.75568154 -0.62410999 0.06116621
C -2.81311152 0.66345026 -2.13171387
C -4.11747385 0.11551382 0.04497747
C -2.95129359 0.89327769 -0.60704791
F -3.88357705 1.14135323 -2.80350413
F -1.71566581 1.31939936 -2.57754953
F -2.66544734 -0.64527045 -2.44169772
F -5.28522892 0.30704643 -0.60271221
F -3.86646460 -1.22191846 0.07151827
F -4.27318586 0.52417486 1.32238512
H -1.49499043 -0.36257488 -0.01313376
H -3.16674536 1.96267868 -0.47092316
O -1.75463984 0.60684700 0.06998400

h4-5.xyz
48
-3160.90704619897

C -2.62792386 0.86065193 2.20297109
C -3.59795282 1.99644528 0.08047919
C -2.95283180 0.73292371 0.69464831
F -3.75226851 0.98505956 2.94189548
F -1.98419355 -0.25863074 2.61131241
F -1.82564559 1.91730924 2.46739439
F -4.65949481 2.42592938 0.79374168
F -2.70386887 3.01908871 -0.00004735
F -4.01667157 1.72434299 -1.17414168
H -1.09432639 1.06855471 0.00013066
H -3.68935974 -0.07780427 0.60146373
O -1.81907028 0.37107548 -0.05124195
C 2.66864279 -0.81893984 2.16499774
C 3.59047686 -2.02915089 0.06166633
C 2.97325374 -0.73955934 0.64906220
F 3.80025363 -0.95172401 2.89132350
F 2.05940382 0.32733139 2.55144778
F 1.84385201 -1.84691533 2.46920246
F 4.65839411 -2.45304383 0.76840429
F 2.68097187 -3.04086467 0.02666050
F 3.99035141 -1.80003754 -1.20767213
H 1.10750677 -1.07704915 -0.02380780
H 3.71984525 0.05727193 0.52208452
O 1.83330852 -0.38166162 -0.08993424
C 2.02959452 3.57773582 -0.08831946
C 0.81001720 2.65221350 -2.18425745
C 0.73741159 2.95847003 -0.66819909
F 2.45351198 4.63927289 -0.80463198
F 1.80463134 3.98772729 1.17845630
F 3.03999500 2.66684639 -0.04918664
F 0.92207525 3.78526626 -2.91190275
F 1.84703502 1.84140466 -2.49565999
F -0.33032253 2.02698053 -2.56194537
H 1.07846076 1.09394220 0.00545005
H -0.05930622 3.70487909 -0.53886293
O 0.38367193 1.81991782 0.07444740
C -2.00387890 -3.59927038 -0.05499338
C -0.87007242 -2.63825807 -2.18284794
C -0.73917476 -2.96207566 -0.67447683
F -2.44524227 -4.65660080 -0.76699243
F -1.72841601 -4.02147119 1.19768472
F -3.01968665 -2.69768624 0.03207073
F -1.00616559 -3.76290547 -2.91956715
F -1.92090230 -1.82788973 -2.44487074
F 0.25312553 -2.00427630 -2.59577997
H -1.06568670 -1.10384031 0.02339416
H 0.06783506 -3.70300180 -0.58347994
O -0.36854769 -1.82948934 0.06910829

h5-1.xyz
60
-3951.13677861153
C -3.24971435 1.98066401 1.66979834
C -3.65995586 2.56863856 -0.82463901
C -3.27446196 1.48119398 0.20564452
F -4.46968611 2.39206343 2.07491803
F -2.85625941 0.97314533 2.47937905
F -2.37989671 3.00896744 1.84413400
F -4.85381162 3.12893870 -0.53601997
F -2.73535806 3.56617590 -0.88386650
F -3.73915902 2.01781445 -2.05237144
H -1.26484600 1.47106053 -0.01005038
H -4.06226018 0.71740941 0.15155692

O -2.06102949 0.86965386 -0.15275096
C 1.33545733 4.20692904 1.00954529
C 0.86062740 3.77061881 -1.50213505
C 0.39670302 3.56709526 -0.03949191
F 1.55646504 5.51422618 0.75436300
F 0.77909442 4.10494212 2.23364591
F 2.54236494 3.58052952 1.05628558
F 0.81575546 5.07198391 -1.85698647
F 2.12816784 3.32431992 -1.70551102
F 0.04896109 3.08039732 -2.33180089
H 1.00223876 1.64661984 0.08243904
H -0.56816534 4.08385854 0.05411793
O 0.18598496 2.21021866 0.25848442
C 4.05723949 0.26596698 1.22502087
C 4.26986510 0.07045399 -1.35140185
C 3.52863360 0.71765909 -0.15852733
F 5.31894252 0.69531199 1.43495553
F 3.27225106 0.77372498 2.19726299
F 4.04972968 -1.08906838 1.36124464
F 5.60710831 0.23645352 -1.26115235
F 4.01667850 -1.26219165 -1.43531201
F 3.85221671 0.63628073 -2.50258376
H 1.87744069 -0.45098566 -0.23828867
H 3.71833368 1.79793881 -0.22193020
O 2.14442879 0.51994199 -0.27645590
C 1.31073265 -3.48745881 1.64148214
C 1.53193774 -4.23579978 -0.82939367
C 1.75059148 -3.10759534 0.20639723
F 2.11976911 -4.43349006 2.16459408
F 1.36959859 -2.39783427 2.43674413
F 0.03695435 -3.95642475 1.68184368
F 2.06822297 -5.40545155 -0.42034358
F 0.21007954 -4.44607349 -1.07643359
F 2.11000253 -3.89263925 -1.99858927
H 0.14303453 -1.92897847 -0.12216962
H 2.83452524 -2.93485086 0.25101230
O 1.14418017 -1.92092851 -0.23725279
C -3.56954897 -2.74400669 0.73711903
C -2.88146660 -2.38602402 -1.73889702
C -2.41448942 -2.64609792 -0.28665441
F -4.48965307 -3.65989482 0.36736342
F -3.07793099 -3.10221955 1.94003069
F -4.20859176 -1.55227008 0.89727528
F -3.60499019 -3.41968435 -2.21851447
F -3.63518164 -1.26144142 -1.84039831
F -1.80036405 -2.22639502 -2.53394447
H -1.79687638 -0.74972938 0.04181839
H -1.92373542 -3.62909064 -0.29000004
O -1.46626202 -1.69727832 0.13323271

h6-1.xyz
72
-4741.36591041778
C -2.40370756 -2.57830436 -2.65011546
C -3.36368967 -3.28299719 -0.35296994
C -2.92475512 -2.11312426 -1.26758044
F -3.22255579 -3.50175487 -3.20475683
F -2.34401650 -1.51969858 -3.48768032
F -1.16402711 -3.10848016 -2.56099369
F -4.49870607 -3.85804734 -0.79602424
F -2.40982502 -4.24888818 -0.25814711
F -3.58913015 -2.82166750 0.90048114
H -1.13399798 -1.75808502 -0.39054446

H	-3.82705187	-1.51591360	-1.45571298
O	-1.98606660	-1.28015782	-0.63893076
C	4.68465649	-1.57271564	-0.82869396
C	4.28404114	0.13825886	1.07033736
C	3.76182722	-1.11705432	0.32753657
F	5.97171887	-1.68533323	-0.42568675
F	4.27730235	-2.78664663	-1.26718890
F	4.63918193	-0.72010418	-1.87458832
F	5.35991832	-0.16976946	1.82814215
F	4.63089541	1.14209277	0.23533730
F	3.31846131	0.61335081	1.89871815
H	2.30923029	-0.07818514	-0.60539786
H	3.75853278	-1.92840612	1.06755203
O	2.44799818	-0.95871107	-0.14303451
C	2.56486504	2.87422943	-2.69718297
C	0.82433933	0.98931526	-3.09944512
C	1.37508078	2.06503773	-2.13790981
F	2.24094107	3.47808366	-3.86435947
F	2.89634516	3.84186107	-1.80804055
F	3.65478172	2.10953069	-2.90288022
F	0.32178999	1.52934888	-4.22895307
F	1.75687942	0.07433724	-3.43919874
F	-0.18347257	0.33119719	-2.46803123
H	1.11384485	1.64137636	-0.20590383
H	0.55758219	2.78299811	-1.97101137
O	1.78012114	1.45496457	-0.93521476
C	-3.34088421	2.23011837	-1.14106213
C	-4.70740706	1.17617829	0.79855505
C	-3.37431678	1.81813917	0.34943731
F	-4.28123949	3.15037888	-1.43196228
F	-2.12788724	2.77612504	-1.41843302
F	-3.50911273	1.17268375	-1.97002637
F	-5.75930221	1.98552117	0.55422686
F	-4.93591337	-0.00818311	0.16891908
F	-4.66271275	0.93078076	2.12329880
H	-2.28876002	0.12296757	0.15089439
H	-3.27029157	2.74599607	0.92913915
O	-2.29122915	0.98628162	0.67463145
C	1.12919246	-4.31601790	1.42844625
C	0.26004305	-2.21082805	2.67924818
C	0.14501582	-3.12385391	1.43699371
F	1.01378358	-5.06789545	2.54375510
F	0.87446146	-5.10119766	0.36284779
F	2.42286344	-3.90514564	1.33385250
F	-0.02483087	-2.87673731	3.81796960
F	1.49484710	-1.66730261	2.80558142
F	-0.62701087	-1.19457247	2.55843286
H	1.12775427	-1.90320567	0.16144739
H	-0.86154005	-3.56362364	1.47354637
O	0.24857707	-2.38842460	0.24389770
C	-0.08176358	4.21868003	1.47638304
C	-0.03204539	2.48726767	3.40648994
C	0.45315260	2.85870979	1.98533206
F	0.36689003	5.24786413	2.21783683
F	0.33416775	4.41167960	0.20057430
F	-1.44174585	4.26509819	1.46814907
F	0.19740056	3.48451680	4.28840963
F	-1.35875523	2.20833877	3.41911911
F	0.62594330	1.39060659	3.83389887
H	-0.81485258	1.59842675	1.00993333
H	1.54728737	2.95861212	2.04135923
O	0.16642880	1.82679939	1.07755062

h6-2.xyz

72

-4741.36587775486

C	-3.40915319	-2.17704256	1.12450607
C	-4.74319808	-1.08244457	-0.81408304
C	-3.42620149	-1.75593156	-0.36382262
F	-4.36472690	-3.08599352	1.40144878
F	-2.20641417	-2.74012876	1.40921902
F	-3.57070000	-1.12210553	1.95884653
F	-5.81345089	-1.86928733	-0.57561331
F	-4.94704735	0.10394928	-0.18019046
F	-4.68936311	-0.83260137	-2.13757788
H	-2.29644107	-0.08931724	-0.14802334
H	-3.34046699	-2.68264852	-0.94828220
O	-2.32383139	-0.94662602	-0.68155256
C	4.76502979	1.45379746	0.78284460
C	4.22168259	-0.19626074	-1.13355643
C	3.78251187	1.06582847	-0.34905082
F	6.04145496	1.51316548	0.33611736
F	4.43271657	2.67609353	1.25870084
F	4.71502458	0.58281570	1.81326850
F	5.28182330	0.07937559	-1.92509266
F	4.55432122	-1.23035530	-0.32954398
F	3.20707990	-0.61399383	-1.93338023
H	2.31477531	0.07775011	0.61590915
H	3.79222603	1.89206860	-1.07236202
O	2.47933793	0.95874441	0.16374111
C	2.55571891	-2.95154170	2.59860856
C	0.86648253	-1.05721119	3.15342014
C	1.36049279	-2.09620395	2.12350201
F	2.24207722	-3.65437683	3.71196161
F	2.87600938	-3.83911946	1.62566877
F	3.65024967	-2.20983066	2.85876808
F	0.46141378	-1.63655053	4.30287944
F	1.80947892	-0.13655001	3.44655857
F	-0.19609823	-0.39940698	2.62080705
H	1.06638952	-1.63320432	0.20431193
H	0.52487422	-2.79274221	1.95591548
O	1.72445418	-1.43252712	0.93683892
C	-2.37059637	2.61690839	2.65257634
C	-3.32276063	3.29716127	0.34535633
C	-2.88785777	2.13662699	1.27411062
F	-3.18992160	3.54890027	3.19223297
F	-2.31697850	1.56819963	3.50269916
F	-1.12945059	3.14326220	2.56239722
F	-4.46712386	3.86648777	0.77108045
F	-2.37533573	4.27043023	0.25644428
F	-3.52865821	2.82684568	-0.90775897
H	-1.09926851	1.76719185	0.39968976
H	-3.79212774	1.54404367	1.46704072
O	-1.94960616	1.29210697	0.65934567
C	1.21316433	4.36395713	-1.30413896
C	0.33910735	2.33258172	-2.66760112
C	0.21340220	3.18739718	-1.38542854
F	1.14877039	5.15374652	-2.39716620
F	0.93280657	5.11797456	-0.22228178
F	2.49589387	3.92888604	-1.17811301
F	0.05863048	3.05151158	-3.77491035
F	1.57724211	1.79994754	-2.81291690
F	-0.54392324	1.30854170	-2.60124328
H	1.16639285	1.91028711	-0.14376302
H	-0.78677390	3.64116314	-1.41891272
O	0.28779767	2.39703643	-0.22610305
C	-0.18095183	-4.23740041	-1.44730140

C -0.11388563 -2.51959824 -3.38875530
C 0.37444030 -2.88979149 -1.96838389
F 0.24172788 -5.27858431 -2.18727747
F 0.24265817 -4.43171031 -0.17436314
F -1.54148504 -4.25879914 -1.42655756
F 0.11033049 -3.51980038 -4.26863981
F -1.44016760 -2.23697837 -3.39857260
F 0.54484748 -1.42526105 -3.82034677
H -0.86600348 -1.59617351 -1.00084102
H 1.46631931 -3.00873064 -2.03080075
O 0.11023566 -1.84757091 -1.06605879

m1-1.xyz
6
-115.78079514999
C 0.73156690 -0.01321351 0.00000000
H 1.12519461 -0.52204156 0.89787080
H 1.10231270 1.01936069 0.00000000
H 1.12519461 -0.52204156 -0.89787080
O -0.69473829 0.06453739 0.00000000
H -1.04249921 -0.84224589 0.00000000

m2-1.xyz
12
-231.57202417878
C 2.02728443 0.59601566 -0.08895702
H 3.10077729 0.47628173 -0.30495342
H 1.57297747 1.20294552 -0.88004089
H 1.89884284 1.11798726 0.87235879
O 1.34590982 -0.67056522 -0.10511484
H 1.72623711 -1.22908902 0.59363792
C -2.16481177 0.04943658 -0.47671349
H -1.80814072 0.65873155 -1.32844895
H -2.33570751 -0.98130156 -0.83890661
H -3.12977641 0.45884540 -0.14808150
O -1.28112491 0.09558623 0.63609098
H -0.41473597 -0.26900591 0.34680318

m2-14.xyz
12
-231.57202181315
C 2.01938355 -0.60895448 0.02828418
H 1.59052950 -1.27352246 0.78661892
H 1.84747304 -1.05129972 -0.96560080
H 3.10164645 -0.51462020 0.21009709
O 1.35187610 0.65714853 0.17219233
H 1.72279187 1.27180175 -0.48324153
C -2.16173846 -0.11295504 0.46964680
H -1.80141660 -0.83071380 1.23047225
H -2.33253141 0.85950889 0.96753025
H -3.12732420 -0.47611604 0.09212462
O -1.28196598 -0.00813055 -0.64223941
H -0.41452178 0.31541295 -0.31030831

m3-1.xyz
18
-347.37531835482
C -1.46387023 2.09370774 0.34553302
H -1.54530160 1.61064858 1.33441265
H -2.46990051 2.19087186 -0.08221990
H -1.04385870 3.10421509 0.48138089
O -0.68086458 1.32353419 -0.56828526
H 0.20798837 1.15027865 -0.15500761
C -1.51165646 -2.06716150 0.34425406
H -1.12480820 -3.07390875 0.54644504
H -2.49085541 -2.16369203 -0.15353139
H -1.65278281 -1.54626078 1.30715610
O -0.56438909 -1.40393262 -0.49476387
H -0.87676558 -0.47273051 -0.65272782
C 2.73946521 -0.02558876 -0.13654722
H 3.29949634 -0.90990626 0.21007902
H 3.31816332 0.87241636 0.11428090
H 2.63055926 -0.08137540 -1.23267994
O 1.47557214 0.08005670 0.52401110
H 0.90513544 -0.68654216 0.24614133

m4-1.xyz
24
-463.18117605714
C 0.87947720 2.44531774 1.19381200
H 0.29136800 3.42829966 1.13442920
H 1.94341040 2.60440752 1.25263310
H 0.59787650 1.93678992 2.11439180
O 0.69246250 1.66583532 0.02542490
H -0.27258640 1.46807412 -0.01271610
C 2.54281920 -1.12242184 -1.19381200
H 3.47305240 -0.62749480 -1.13442920
H 2.00437740 -0.78982903 -2.11439180
H 2.80654860 -2.20694823 -1.25263310
O 1.74136970 -0.85840867 -0.02542490
H 1.44844010 0.13053908 0.01271610
C -0.87947720 -2.44531774 1.19381200
H -0.59787650 -1.93678992 2.11439180
H -0.29136800 -3.42829966 1.13442920
H -1.94341040 -2.60440752 1.25263310
O -0.69246250 -1.66583532 0.02542490
H 0.27258640 -1.46807412 -0.01271610
C -2.54281920 1.12242184 -1.19381200
H -2.80654860 2.20694823 -1.25263310
H -3.47305240 0.62749480 -1.13442920
H -2.00437740 0.78982903 -2.11439180
O -1.74136970 0.85840867 -0.02542490
H -1.44844010 -0.13053908 0.01271610

m4-7.xyz
24
-463.17992311548
C -2.10306831 2.04316936 -0.29604892
H -3.09893318 1.95602773 0.16847969
H -2.14104630 1.60005533 -1.30600646
H -1.85783735 3.10862087 -0.39289383
O -1.09943893 1.43721954 0.52034062
H -1.29352584 0.45051403 0.58726016
C -2.30925988 -1.84730240 -0.27944895
H -2.25441358 -1.50950863 -1.32848788
H -3.29705591 -1.58773456 0.12335860
H -2.20251289 -2.94418298 -0.25830205
O -1.32385944 -1.21791362 0.54098651
H -0.41367708 -1.40528190 0.14865536
C 2.30925988 1.84730240 0.27944895

H 2.20251289 2.94418298 0.25830205
H 3.29705591 1.58773456 -0.12335860
H 2.25441358 1.50950863 1.32848788
O 1.32385944 1.21791362 -0.54098651
H 0.41367708 1.40528190 -0.14865536
C 2.10306831 -2.04316936 0.29604892
H 1.85783735 -3.10862087 0.39289383
H 2.14104630 -1.60005533 1.30600646
H 3.09893318 -1.95602773 -0.16847969
O 1.09943893 -1.43721954 -0.52034062
H 1.29352584 -0.45051403 -0.58726016

m5-1.xyz
30
-578.98065884970
C -0.32932325 2.70112123 -1.29657031
H -0.38982375 1.86530493 -2.01471163
H -1.23112544 3.32615577 -1.40434438
H 0.54822510 3.31169753 -1.54553548
O -0.17845229 2.24255397 0.04780849
H -0.93457607 1.60056514 0.24676379
C -1.29926726 -2.87500944 0.89084724
H -0.88680667 -3.84697064 0.57580665
H -0.85033406 -2.59738368 1.85926493
H -2.38354631 -2.98373871 1.02513643
O -1.07797314 -1.87647335 -0.10753853
H -0.07770870 -1.76244856 -0.22319247
C -3.33504917 0.64493514 -0.14340348
H -3.69840536 1.65576849 0.08465856
H -3.25500805 0.53640831 -1.23862760
H -4.07668933 -0.08139688 0.22791224
O -2.07656068 0.47367821 0.50693069
H -1.70863698 -0.44091140 0.27204977
C 2.59798304 1.12970212 1.66419258
H 3.50224132 0.51591692 1.76773817
H 2.87161573 2.18672123 1.81239820
H 1.88101443 0.83636752 2.44947597
O 2.07173364 0.91672538 0.35309811
H 1.21785327 1.45217395 0.25425055
C 2.10338444 -1.80901135 -1.60283620
H 3.20319817 -1.76501891 -1.54941440
H 1.80874221 -2.81963537 -1.91464338
H 1.75847967 -1.09121044 -2.36634222
O 1.51769163 -1.55519625 -0.32553072
H 1.75607438 -0.61174915 -0.04235365

m5-10.xyz
30
-578.98052123604
C -0.85033136 3.01746746 -0.98811192
H -0.65286297 2.55960668 -1.97161013
H -0.14661961 3.84671293 -0.83862994
H -1.87493841 3.42371873 -0.98680214
O -0.66585155 2.08710570 0.08107344
H -1.25778381 1.28439908 -0.09160883
C -3.36762869 -0.18944463 0.26662010
H -3.94120963 -1.04367001 -0.12867622
H -3.96802610 0.72054069 0.13696399
H -3.19844653 -0.34410875 1.34646066
O -2.14639747 -0.01654667 -0.45043631
H -1.58990161 -0.85727092 -0.35514174
C 2.26774854 1.56109556 1.65778040
H 2.32370204 2.65145336 1.80802343
H 3.27532222 1.14046916 1.77260456
H 1.61584386 1.13073739 2.43673290
O 1.80893495 1.24737379 0.34216173
H 0.86040075 1.58848324 0.23993828
C 2.50145150 -1.44860599 -1.56209671
H 3.56108564 -1.15435353 -1.49149889
H 2.02040671 -0.85061239 -2.35439824
H 2.45043314 -2.50850669 -1.84341921
O 1.84299313 -1.29743666 -0.30334221
H 1.85364653 -0.31765029 -0.04728829
C -0.76122458 -2.80183064 1.11780918
H -0.05451077 -3.64233315 1.20570248
H -1.78292093 -3.19697888 1.19535977
H -0.58649765 -2.10790237 1.95861599
O -0.63367234 -2.15503932 -0.14909172
H 0.32511426 -1.83966672 -0.24077922

m6-1.xyz
36
-694.77868429344
C -0.20151675 1.49777404 2.17629091
H -1.02101280 2.21932673 2.32705397
H 0.37188276 1.42494878 3.10940973
H -0.62890044 0.50725468 1.94521815
O 0.69002355 1.95116704 1.15162583
H 0.16561603 2.01981161 0.28767387
C 1.89474526 -2.86236559 -0.53701389
H 1.27265714 -3.62080579 -1.03049918
H 2.11450954 -3.19753810 0.49091624
H 2.84400099 -2.78092122 -1.09123487
O 1.18169148 -1.62551768 -0.55442153
H 1.75337409 -0.91912681 -0.09674285
C -1.75274840 -2.93700184 0.52876957
H -1.83803830 -3.34401526 -0.49326991
H -2.75570585 -2.90024723 0.97369145
H -1.13095853 -3.61750723 1.13317148
O -1.22105168 -1.61205296 0.53371411
H -0.29387235 -1.63425416 0.12794287
C 0.13991395 1.49429483 -2.19913490
H 0.49035644 0.46520212 -2.01176832
H 1.01139373 2.15389931 -2.30640898
H -0.42716835 1.51199524 -3.14362858
O -0.65348082 1.99753489 -1.11848694
H -1.43863446 1.37624296 -0.99576021
C -3.64766573 0.72528784 0.17543716
H -4.10173068 1.61457849 -0.28037079
H -3.25992583 0.99904683 1.17137338
H -4.43074608 -0.04101883 0.29976378
O -2.61247725 0.26772104 -0.69547610
H -2.12220164 -0.48763736 -0.24123565
C 3.60630491 0.84792772 -0.11355660
H 4.38664647 0.10106878 -0.31136731
H 4.05628939 1.68500818 0.44310440
H 3.23047660 1.22715563 -1.07965067
O 2.57815775 0.21965088 0.65285650
H 1.87604571 0.91905983 0.88487301

m6-2.xyz
36

-694.77958502177
C -3.43317326 0.49567971 0.49796669
H -2.92612678 0.76096745 1.44148288
H -3.95706555 1.38283255 0.11819113
H -4.18246399 -0.28477681 0.70839194
O -2.51057360 0.06618128 -0.50416673
H -1.99956584 -0.73074874 -0.14008532
C 3.91295922 0.35022139 -0.03717243
H 4.46146354 0.99834834 0.66557733
H 4.58086744 -0.46048439 -0.35680861
H 3.63427712 0.94701009 -0.92293429
O 2.77645881 -0.24161044 0.59119760
H 2.13341648 0.49124510 0.86217044
C 1.02920329 -1.22507005 -2.08723706
H 1.95404990 -1.30450033 -2.68159417
H 0.23463012 -1.77353316 -2.60999129
H 0.73470048 -0.16565257 -2.00994876
O 1.20072011 -1.82715309 -0.79765534
H 1.86263425 -1.26461576 -0.27813014
C -1.53929524 -3.20378503 0.57546125
H -0.81193598 -3.85737710 1.08224113
H -2.47003646 -3.19945811 1.15820019
H -1.75171210 -3.61915010 -0.42437501
O -1.05593822 -1.86206967 0.50475395
H -0.16209455 -1.86175473 0.01526697
C 0.15630977 1.09541213 2.30096634
H -0.31029936 0.12677583 2.05446098
H 0.79321470 0.96511693 3.18518887
H -0.63200490 1.82679501 2.54496350
O 0.98528749 1.57593643 1.23606148
H 0.40549209 1.68849522 0.41973193
C -1.00617854 3.03266125 -1.36077875
H -1.67020815 3.50961443 -0.61921697
H -0.13101172 3.67838860 -1.50870688
H -1.54610635 2.94865949 -2.31776284
O -0.54064722 1.75735654 -0.92084921
H -1.33222049 1.13640552 -0.80167115

m6-3.xyz
36
-694.77951869358
C 0.17491195 1.11742376 -2.29992857
H -0.65312559 1.81717683 -2.47694348
H -0.23748879 0.11191562 -2.11214734
H 0.80115108 1.08364805 -3.20532606
O 0.92558995 1.60439058 -1.18121314
H 1.67694864 0.94682100 -1.00171677
C -1.77460609 -3.11240031 -0.54717552
H -2.65126947 -3.07526910 -1.21470977
H -2.11191012 -3.39058470 0.46547035
H -1.09059679 -3.88802628 -0.91481684
O -1.06986955 -1.87006595 -0.55207267
H -1.66409632 -1.15422355 -0.15514306
C 0.99208895 -1.35373000 2.03969687
H 0.59545921 -0.32512091 2.01334306
H 1.93967243 -1.35916298 2.59432739
H 0.27591974 -1.99985189 2.57255040
O 1.25581496 -1.86032889 0.72451988
H 0.37748335 -1.89198462 0.23069065
C -0.72022464 3.06376099 1.38948378
H 0.24100538 3.53942872 1.64243898
H -1.35230985 3.05558483 2.28727013
H -1.21799527 3.66978780 0.61307219
O -0.52957096 1.71266524 0.97303206
H 0.05458849 1.69674388 0.13778636
C -3.42340608 0.68245597 -0.41857705
H -3.93330178 1.54294304 0.04464595
H -4.18237461 -0.06170948 -0.69327760
H -2.91772280 1.02295982 -1.33854619
O -2.51581865 0.06769015 0.49656784
H -1.79872961 0.74279460 0.73062874
C 3.91207566 0.17005519 0.05706839
H 4.55783773 -0.70173933 0.25110864
H 3.65939729 0.64544324 1.02111144
H 4.47431525 0.89156436 -0.55004838
O 2.74858127 -0.20849744 -0.67717225
H 2.22443825 -0.88630175 -0.13612395

m6-4.xyz
36
-694.77960191989
C 0.71460886 -1.07776829 2.39180634
H 1.48969661 -0.80266325 3.12586874
H 0.04935518 -1.81841818 2.85477207
H 0.12650081 -0.18049408 2.13692053
O 1.30210616 -1.68605494 1.23527047
H 1.85273758 -0.98741624 0.75866827
C -2.21706989 2.58519083 1.02565101
H -3.00698154 2.30152884 1.74056975
H -2.68958589 2.87310081 0.07124713
H -1.68123074 3.45470931 1.42786195
O -1.27350481 1.52693691 0.85530860
H -1.73231428 0.75117101 0.38720871
C 0.28557693 1.89569021 -2.05012243
H -0.39333274 1.03624709 -2.17509620
H 1.13960118 1.77347979 -2.72889455
H -0.25422166 2.81573330 -2.32890632
O 0.79243505 1.98387769 -0.71508159
H 0.01268087 1.87141820 -0.08245848
C 0.28295258 -2.27310580 -1.87299966
H 1.01284026 -1.44930727 -1.93358147
H -0.43669766 -2.17431267 -2.69642627
H 0.81967044 -3.22883598 -1.98733263
O -0.44731568 -2.24471385 -0.64272337
H 0.21516451 -2.09968104 0.11168486
C 3.78888086 0.74531549 0.29473175
H 4.48946722 -0.06079755 0.54893619
H 3.62460895 1.36637687 1.19167226
H 4.24556428 1.37195234 -0.48858800
O 2.57971689 0.14390764 -0.16763017
H 1.90786594 0.87137833 -0.37781346
C -3.48887023 -1.11111845 0.09195124
H -4.29961539 -0.37196983 0.14113271
H -3.30077883 -1.49766208 1.10817258
H -3.81586348 -1.94646374 -0.54709861
O -2.33749190 -0.46816182 -0.45740885
H -1.59821824 -1.16253066 -0.55080615

m6-6.xyz
36
-694.77905413214
C -3.83991741 0.16790766 0.49921671
H -4.35697945 1.11410451 0.70525610
H -3.61672861 -0.32967462 1.45819386

H -4.51495063 -0.47935553 -0.08416544
O -2.65347633 0.47002731 -0.23638551
H -2.13041858 -0.38238636 -0.36944944
C -0.45823007 1.50261856 2.02509531
H 0.40588081 2.06501856 2.40064178
H -0.13865687 0.46520540 1.82706788
H -1.23756364 1.49289521 2.80461164
O -0.92528137 2.15157372 0.83882308
H -1.64093575 1.56233488 0.43804601
C -1.23677725 -2.41417127 -1.77784876
H -0.56498857 -1.90998024 -2.49372977
H -2.25424500 -2.40459037 -2.18996363
H -0.91147182 -3.46098012 -1.66692478
O -1.26853842 -1.74999834 -0.51520334
H -0.33820604 -1.75476822 -0.11842366
C 3.85785030 0.22561285 -0.39536743
H 3.74175768 -0.11117486 -1.44001349
H 4.47196041 -0.50975755 0.14082699
H 4.38601589 1.19232463 -0.39159166
O 2.60329093 0.32732850 0.27857133
H 2.03499512 1.02887525 -0.18789219
C 0.46300053 1.42074994 -2.08576870
H 0.00904833 0.44314838 -1.84941184
H 1.26783649 1.27097562 -2.81719047
H -0.30778970 2.06405081 -2.53782769
O 1.03203388 2.03894274 -0.92728323
H 0.28095983 2.17555055 -0.26134208
C 1.27762931 -2.35219444 1.81186446
H 2.30272518 -2.71019841 2.00130730
H 0.58755929 -3.20024195 1.91146378
H 1.02087794 -1.59860246 2.57575167
O 1.14984144 -1.83555105 0.48768878
H 1.73217701 -1.00983562 0.39619251

m6-11.xyz
36
-694.77911689268
C -0.52565097 -2.00211962 -2.04020899
H 0.02414163 -1.07847577 -2.29050146
H 0.10722189 -2.86376126 -2.29083150
H -1.43869470 -2.04756892 -2.65380210
O -0.84181554 -2.07728551 -0.64729783
H -1.53100649 -1.36064003 -0.44189981
C 2.03162057 -2.71162020 1.01418497
H 1.42653134 -3.47123851 1.53399047
H 2.41765368 -3.14783720 0.07664576
H 2.88424103 -2.44785346 1.65390406
O 1.27230825 -1.52565683 0.77954485
H 0.43768062 -1.77124182 0.25161711
C -0.55876559 1.13831071 2.34894898
H -1.30542439 0.77853041 3.06913399
H 0.10494260 0.30047892 2.07740908
H 0.03597750 1.92962978 2.83313462
O -1.26357865 1.65781946 1.21464105
H -0.58086028 1.99055114 0.54581912
C -0.13102796 2.32385749 -1.91380174
H -0.72164343 1.40392283 -2.06625212
H -0.80904667 3.18487911 -1.97685241
H 0.61802749 2.40555894 -2.71707673
O 0.49307198 2.35254941 -0.62695853
H 1.21145621 1.64351463 -0.60653400
C 3.53246261 0.93196873 0.16236376
H 4.33940929 0.18328992 0.09828348
H 3.87647928 1.85573559 -0.32034859
H 3.32982687 1.14636603 1.22539868
O 2.36195968 0.48759914 -0.52561495
H 1.98621790 -0.31081798 -0.03320475
C -3.81546558 -0.44328759 0.39778187
H -4.44593577 0.45960182 0.43313184
H -4.30770122 -1.18898897 -0.24020746
H -3.73081124 -0.85661482 1.41754683
O -2.53889348 -0.15183739 -0.16956493
H -2.07647754 0.55697625 0.39483959

h1m1-1.xyz
18
-906.01152363981
C -3.57275534 -0.76505584 -0.24611073
H -1.96345270 -1.49733776 -1.13608920
H -3.38624276 0.28647301 -0.51118435
H -4.31157934 -1.19822365 -0.93686446
H -3.96849265 -0.81236933 0.77425527
O -2.35644787 -1.53705596 -0.24713857
C 1.34985102 -0.80889101 -0.01504653
C -0.02064810 1.38221213 0.03732418
C 0.48741349 0.15550159 0.83632803
F 2.37578627 -0.18161214 -0.63647149
F 1.87317095 -1.76813054 0.77877215
F 0.61208546 -1.43708814 -0.97547188
F 0.99278100 2.15442205 -0.41827810
F -0.77834788 1.02302045 -1.03975767
F -0.79766711 2.15139997 0.83220654
H -1.17857538 -0.91281948 0.81315789
H 1.16271772 0.56306548 1.60562215
O -0.54519708 -0.51679339 1.48145174

h1m1-3.xyz
18
-906.01152576438
C 3.56771979 -0.79916672 -0.22427689
H 1.95965365 -1.46764202 -1.16413206
H 4.30715602 -1.20805430 -0.92901796
H 3.39765376 0.26651433 -0.43951564
H 3.95327040 -0.90147597 0.79592027
O 2.34108946 -1.55337917 -0.27337640
C 0.04865171 1.37309294 0.03948937
C -1.37156976 -0.78562001 -0.01429028
C -0.48180607 0.15455932 0.83659446
F -0.94914655 2.17681103 -0.39466225
F 0.85955615 2.11410214 0.82755296
F 0.77974273 1.00137783 -1.05199304
F -2.38142104 -0.13041946 -0.63380752
F -0.65225763 -1.43286243 -0.97524513
F -1.91982709 -1.73021045 0.78046791
H 1.16483184 -0.94196560 0.79673632
H -1.14245395 0.57381163 1.61231053
O 0.53859054 -0.54543198 1.47147697

h1m1-6.xyz
18
-906.01152106646
C -3.55276417 -0.81027733 0.23516358
H -1.93918082 -1.49853538 1.15214011

H -3.37914002 0.24987603 0.47299672
H -4.29068091 -1.23295697 0.93321064
H -3.94183865 -0.88909095 -0.78582539
O -2.32805656 -1.56869419 0.26319006
C 1.37574537 -0.77675988 0.01608886
C -0.06001054 1.37109625 -0.04041756
C 0.48292296 0.15775994 -0.83728054
F 2.36929740 -0.11286783 0.65256138
F 1.94555856 -1.70794239 -0.77932296
F 0.65450162 -1.44143747 0.96370376
F 0.92949121 2.18952751 0.38521913
F -0.77940378 0.99347751 1.05710412
F -0.88519926 2.09844155 -0.82599121
H -1.15598579 -0.95041423 -0.80674343
H 1.14370081 0.58338962 -1.60927251
O -0.52858304 -0.54984354 -1.47805110

h1m1-7.xyz
18
-906.01152143350
C -3.57352133 0.78425087 -0.23220944
H -1.96524628 1.47741046 -1.15414556
H -4.31349278 1.20471007 -0.92956297
H -3.39859244 -0.27596307 -0.46924350
H -3.96128577 0.86362460 0.78919164
O -2.35016024 1.54459279 -0.26329877
C -0.03598892 -1.37699658 0.03855133
C 1.36296280 0.79592400 -0.01447877
C 0.48434810 -0.15455645 0.83624894
F 0.96861345 -2.16744902 -0.40455087
F -0.83252100 -2.13012485 0.82979434
F -0.77843828 -1.01067377 -1.04691768
F 2.37818206 0.15186417 -0.63672753
F 0.63557256 1.43750988 -0.97346231
F 1.90290982 1.74522867 0.78034595
H -1.17160479 0.92888373 0.80216228
H 1.15074617 -0.56849284 1.60985233
O -0.54191821 0.53380443 1.47452363

h1m2-1.xyz
24
-1021.80844213297
C -3.30345451 -0.88378159 -1.78597821
H -1.60133514 -1.60856951 -1.09043066
H -2.92422876 0.12432309 -2.01437548
H -3.32959426 -1.48289935 -2.70897794
H -4.32220347 -0.80326332 -1.39004855
O -2.51831583 -1.53319160 -0.77182089
C -2.84922932 1.01261544 1.59001017
H -2.28656896 -0.69203879 0.73865899
H -2.70020970 1.63834586 0.69463205
H -3.91773442 0.76203523 1.68457775
H -2.54417705 1.58749402 2.47250933
O -2.04747712 -0.17354062 1.55354765
C 1.53222254 -1.00566677 0.18770624
C 0.67260151 1.33362779 -0.47536228
C 1.25256289 0.44543383 0.65572918
F 2.22130656 -1.06182243 -0.97829686
F 2.25218568 -1.65604239 1.12422717
F 0.37659925 -1.71495346 0.00844864
F 1.57196408 1.55056652 -1.46434471
F -0.44052142 0.78767942 -1.05341481
F 0.31156702 2.53585369 0.02119591
H -0.45866325 0.15928052 1.64537740
H 2.24193373 0.87686750 0.88210784
O 0.49254961 0.47003934 1.81270340

h1m2-2.xyz
24
-1021.80842816506
C -3.29487163 0.87441292 -1.79380001
H -1.59814465 1.60773570 -1.09381082
H -2.91111249 -0.13320081 -2.01660859
H -3.31932960 1.46995071 -2.71915021
H -4.31491128 0.79175748 -1.40165141
O -2.51631423 1.53063845 -0.77906571
C -2.84797993 -1.00149624 1.59710610
H -2.28539360 0.69838256 0.73632432
H -2.68933896 -1.63667874 0.71009759
H -3.91771288 -0.75178679 1.67883004
H -2.55030432 -1.56597977 2.48875511
O -2.04782754 0.18561161 1.55525016
C 1.53278128 1.00480054 0.18517857
C 0.67015028 -1.33560130 -0.47159349
C 1.25084140 -0.44459000 0.65681037
F 2.23271475 1.05701545 -0.97433633
F 2.24286371 1.65981048 1.12590264
F 0.37786354 1.71245091 -0.00809089
F 1.56560218 -1.54819967 -1.46513610
F -0.44848221 -0.79526402 -1.04423350
F 0.31715678 -2.53960173 0.02670594
H -0.45988793 -0.15131909 1.64628737
H 2.23971825 -0.87643657 0.88492367
O 0.49033072 -0.46540332 1.81349128

h1m2-3.xyz
24
-1021.80843858349
C 2.84036008 -1.04119733 1.56968262
H 2.28698326 0.68099505 0.74834712
H 2.53608711 -1.62712857 2.44515109
H 2.68187790 -1.65229883 0.66589275
H 3.91109329 -0.79955781 1.66190917
O 2.04682999 0.15093347 1.55547868
C 3.29311090 0.91241558 -1.77908516
H 1.60271109 1.63956304 -1.05816023
H 2.90173087 -0.08645167 -2.02660439
H 3.32051338 1.53088453 -2.68918737
H 4.31314806 0.81265831 -1.39086403
O 2.52074732 1.54843304 -0.74684782
C -1.53085402 1.00477353 0.19411679
C -0.67055676 -1.33007777 -0.48521350
C -1.25280017 -0.45005766 0.65112252
F -2.22549407 1.07054421 -0.96793769
F -2.24422107 1.65061911 1.13859944
F -0.37408311 1.71288299 0.01381269
F -1.56477138 -1.53297832 -1.48189560
F 0.44872810 -0.78416035 -1.05088000
F -0.31844268 -2.53897450 0.00196694
H 0.45714706 -0.17452580 1.64602863
H -2.24308937 -0.88241807 0.87202767
O -0.49519081 -0.48425978 1.80934126

h1m2-5.xyz
24
-1021.80843141685
C -3.29311938 -0.90052763 1.78551166
H -1.60326637 -1.63209168 1.06753320
H -2.90155742 0.09970729 2.02726358
H -3.32001074 -1.51396187 2.69904075
H -4.31330304 -0.80272858 1.39724394
O -2.52149260 -1.54239844 0.75632890
C -2.84732685 1.03263516 -1.57628532
H -2.28847776 -0.68224100 -0.74307079
H -2.69375495 1.64926611 -0.67539577
H -3.91687045 0.78656853 -1.67045766
H -2.54252567 1.61487390 -2.45406272
O -2.04952382 -0.15641441 -1.55325016
C 1.52941897 -1.00694906 -0.19297281
C 0.67425138 1.33162985 0.48152946
C 1.25220182 0.44700843 -0.65317624
F 2.22806910 -1.07077581 0.96676965
F 2.23841976 -1.65627126 -1.13839584
F 0.37240409 -1.71319858 -0.00658615
F 1.56927158 1.53189956 1.47809532
F -0.44792690 0.79256904 1.04801750
F 0.32845419 2.54134755 -0.00802506
H -0.46052050 0.17124459 -1.64423265
H 2.24266317 0.87717127 -0.87758784
O 0.49211428 0.47920587 -1.80985181

h1m2-6.xyz
24
-1021.80842919225
C -3.29554789 0.94409066 1.76546979
H -1.60444237 1.65706498 1.03164791
H -2.90402271 -0.04936462 2.03364954
H -3.32446498 1.58070856 2.66295861
H -4.31499708 0.83597061 1.37793547
O -2.52228490 1.55962060 0.72161412
C -2.83660160 -1.07444135 -1.54419357
H -2.28848745 0.66562461 -0.75745645
H -2.67499492 -1.66656219 -0.62842079
H -3.90842675 -0.83923432 -1.64020711
H -2.53066121 -1.67656068 -2.40800070
O -2.04798976 0.12098589 -1.55471553
C 1.52848287 1.00525541 -0.20132291
C 0.67269672 -1.32530859 0.49524269
C 1.25414891 -0.45355084 -0.64802777
F 2.21280605 1.08114235 0.96612997
F 2.24974066 1.64331299 -1.14515507
F 0.37013182 1.71488115 -0.03742763
F 1.57252331 -1.52960219 1.48654220
F -0.43916623 -0.77010219 1.06639254
F 0.30975713 -2.53398220 0.01549029
H -0.45609691 -0.19445456 -1.64602408
H 2.24558768 -0.88558520 -0.86414593
O 0.49856229 -0.49766465 -1.80722000

h1m2-7.xyz
24
-1021.80812181885
C 3.01427630 0.99517846 -1.80826925
H 2.37245770 2.10476461 -0.31057817
H 2.02196658 0.63279361 -2.11424129
H 3.32458116 1.82460571 -2.46163947
H 3.74045228 0.18009640 -1.90443407
O 3.03031511 1.39705947 -0.42499611
C 2.69456564 -1.78052233 1.04565085
H 2.50257858 0.17083328 0.69705561
H 3.79103840 -1.76939009 1.14948192
H 2.27984380 -2.49002725 1.77167014
H 2.42753912 -2.11942935 0.03138533
O 2.14091064 -0.49414934 1.34189963
C -1.22861933 1.21906945 0.30841229
C -1.02548430 -1.22279533 -0.50910549
C -1.27609974 -0.28048682 0.69565195
F -2.01698889 1.51850298 -0.75142446
F -1.63908079 1.97150424 1.35174073
F 0.03930583 1.62629995 -0.00395496
F -1.99539209 -1.11815351 -1.45008550
F 0.16714131 -0.98124564 -1.12504682
F -1.00341420 -2.50620153 -0.08616814
H 0.52872469 -0.47297795 1.53055812
H -2.31900555 -0.47046914 1.00002878
O -0.45180345 -0.55463674 1.77450795

h1m2-8.xyz
24
-1021.80811795456
C -3.00285879 -0.89539209 1.87088590
H -2.33157857 -2.07590884 0.44180093
H -2.02044453 -0.49071890 2.15531011
H -3.29343202 -1.69226751 2.57210791
H -3.74915515 -0.09435832 1.91848516
O -3.00622214 -1.37849289 0.51385121
C -2.75115850 1.70455056 -1.15121506
H -2.49750452 -0.21228812 -0.68001921
H -2.49284378 2.12028678 -0.16342709
H -3.84685364 1.64702778 -1.24731895
H -2.36525433 2.37807909 -1.92581193
O -2.15224067 0.42178151 -1.36372400
C 1.22329676 -1.21956306 -0.31525234
C 1.03888819 1.22491322 0.50817290
C 1.27570900 0.28094332 -0.69748363
F 2.08985078 -1.54248337 0.67390567
F 1.52827696 -1.97448547 -1.39135236
F -0.02385989 -1.60059017 0.10069525
F 1.95116647 1.04502724 1.49455157
F -0.19630568 1.06254922 1.06142222
F 1.12755308 2.51028568 0.09935723
H -0.53570134 0.45958410 -1.52504939
H 2.31730134 0.46454433 -1.01055943
O 0.44278522 0.56995358 -1.76604946

h1m3-1.xyz
30
-1137.61422057072
C -4.58074877 1.40006290 0.63218842
H -3.44816235 -0.21899073 0.35132241
H -3.99591340 1.78938730 1.48165332
H -5.46550084 0.87103186 1.01984140
H -4.91813794 2.24492859 0.01883463
O -3.79881481 0.54112664 -0.20573138
C -1.28667359 1.31854034 -2.41359767

H -2.40926432 1.17990480 -0.75525217
H -1.45892569 0.28424076 -2.75230870
H -0.25405697 1.60139105 -2.64831786
H -1.97106748 1.99153878 -2.95209594
O -1.46415707 1.45371482 -0.99866791
C -2.59139684 -2.67216884 0.59287590
H -1.66350727 -1.00498014 1.14266901
H -1.96404631 -3.37763798 1.15938027
H -2.23999411 -2.64033145 -0.45081709
H -3.62756385 -3.03139751 0.60839050
O -2.58202724 -1.37384987 1.20088013
C 1.91069840 1.07396324 0.64268019
C 1.44522218 -1.24881069 -0.40647897
C 1.08929849 -0.23575764 0.70878441
F 3.24121787 0.83956278 0.74589143
F 1.55498400 1.87874031 1.66954291
F 1.69623222 1.75624581 -0.50858544
F 2.75634479 -1.57919840 -0.40930921
F 1.12453882 -0.79249223 -1.64105268
F 0.73801428 -2.39236374 -0.20304826
H -0.61562740 0.61354570 -0.05187717
H 1.37517450 -0.72097480 1.65613193
O -0.27870097 0.02252387 0.73473761

h1m3-2.xyz
30
-1137.61422727173
C 2.60009012 2.66691052 -0.60672811
H 1.66399134 1.00133185 -1.14789908
H 3.63797447 3.02108656 -0.62479439
H 2.24948745 2.64152656 0.43739855
H 1.97574378 3.37277136 -1.17607004
O 2.58403440 1.36584680 -1.20869488
C 4.59068287 -1.40164136 -0.61958468
H 3.44898664 0.21391622 -0.35437401
H 5.47516909 -0.87040500 -1.00498799
H 4.01317054 -1.79879359 -1.47041619
H 4.92883838 -2.24127447 0.00050106
O 3.79937439 -0.54197417 0.20857632
C 1.28493087 -1.31063291 2.41303183
H 2.40759243 -1.18048772 0.75454263
H 1.45916906 -0.27537598 2.74766129
H 1.96809189 -1.98269803 2.95434830
H 0.25185058 -1.59060518 2.64906669
O 1.46210319 -1.45185937 0.99874659
C -1.44430862 1.24819471 0.41020191
C -1.91486514 -1.07146690 -0.64465832
C -1.09152424 0.23748043 -0.70839876
F -2.75307639 1.58767192 0.40996589
F -0.72875827 2.38789447 0.21479999
F -1.13133995 0.78376194 1.64376929
F -3.24597282 -0.83468347 -0.73449377
F -1.69108870 -1.76255070 0.49953145
F -1.56957899 -1.86927354 -1.68055650
H 0.61329278 -0.61402623 0.04960576
H -1.37786897 0.72568831 -1.65407436
O 0.27598854 -0.02324556 -0.73699688

h1m3-3.xyz
30
-1137.61317024566
C 2.87593830 2.36048223 1.44871898
H 1.63254244 0.80497856 1.51092304
H 2.55902422 2.73819984 0.46296436
H 2.37839029 2.94906224 2.23624773
H 3.95999865 2.49526197 1.54819021
O 2.60556630 0.96464686 1.60198705
C 0.69125965 -3.10776291 -0.52850818
H 2.03607340 -1.80317971 0.20936165
H 1.38888092 -3.95938002 -0.52851825
H -0.30173523 -3.46600190 -0.23364083
H 0.62884204 -2.68992907 -1.54623965
O 1.10318676 -2.12729010 0.42768374
C 3.59858130 -0.48480990 -1.34810050
H 3.27516367 -0.21865741 0.59200792
H 3.74112535 -1.34181944 -2.01845531
H 2.71279543 0.08165150 -1.67999286
H 4.48551972 0.16470373 -1.42083498
O 3.44414077 -0.99517848 -0.01947878
C -0.61746728 0.95732333 -1.00556542
C -2.30768996 -0.19281614 0.58366439
C -1.01303440 0.64699926 0.45858218
F -1.55986143 1.67992889 -1.65107672
F 0.52820709 1.69232517 -1.00812426
F -0.37694051 -0.16107638 -1.73146593
F -3.35043361 0.37518340 -0.06835817
F -2.14704591 -1.44828389 0.09727329
F -2.64684113 -0.29917752 1.88814054
H 0.31587539 -0.86293640 0.78760207
H -1.24390797 1.62250616 0.91644168
O 0.03088199 0.05912388 1.16810058

h1m3-4.xyz
30
-1137.61423145815
C 2.58939955 2.66454821 -0.56399510
H 1.66208921 1.00699936 -1.14151577
H 2.24219553 2.61301166 0.48041511
H 1.95833590 3.37904846 -1.11475494
H 3.62480474 3.02600240 -0.57697211
O 2.58012586 1.37739527 -1.19555870
C 4.57456309 -1.40675099 -0.64488078
H 3.44959642 0.21631521 -0.35647021
H 5.46195632 -0.88246964 -1.03305241
H 3.98444540 -1.78868600 -1.49402678
H 4.90756141 -2.25612300 -0.03544044
O 3.80099514 -0.54509802 0.19830907
C 1.29080063 -1.29643251 2.41519125
H 2.41058165 -1.17805623 0.75344654
H 1.97631460 -1.96376981 2.95935332
H 0.25864821 -1.57640204 2.65509778
H 1.46419063 -0.25862714 2.74236265
O 1.46519984 -1.44692138 1.00132804
C -1.44606380 1.25395480 0.38960532
C -1.90957513 -1.08272621 -0.62908953
C -1.09117992 0.22790315 -0.71410986
F -2.75880258 1.57769569 0.39620008
F -0.74606676 2.39823584 0.16651852
F -1.11524755 0.81560140 1.62808882
F -3.24067103 -0.85233997 -0.73357264
F -1.69224287 -1.74902046 0.53110005
F -1.55363585 -1.90049833 -1.64551614
H 0.61610033 -0.61244618 0.04888643
H -1.38160009 0.70064757 -1.66641579

O 0.27743171 -0.02678067 -0.74079295

h1m3-5.xyz
30
-1137.61422561505
C -2.59732069 -2.66465464 0.62252298
H -1.66421597 -0.99550977 1.15478050
H -3.63349529 -3.02327085 0.64779091
H -1.96615493 -3.36701281 1.18870353
H -2.25348044 -2.63946299 -0.42393131
O -2.58287068 -1.36253367 1.22243734
C -4.57950482 1.41030735 0.62271055
H -3.45000957 -0.21481253 0.36314171
H -3.99302423 1.80965949 1.46635094
H -5.46536599 0.88897469 1.01823983
H -4.91517793 2.24761291 -0.00186422
O -3.80044352 0.53795132 -0.20392071
C -1.30099561 1.26796512 -2.43788375
H -2.41167513 1.16485973 -0.76953789
H -1.99228646 1.92742724 -2.98427605
H -1.47239428 0.22644753 -2.75419600
H -0.27138516 1.54959110 -2.68687550
O -1.46753911 1.43118627 -1.02458762
C 1.90596175 1.08007533 0.64469700
C 1.45471203 -1.24521510 -0.40591405
C 1.08867947 -0.23256449 0.70659718
F 3.23663066 0.84997033 0.75623404
F 1.54134058 1.88396679 1.66929116
F 1.69615215 1.76134069 -0.50784752
F 2.76684730 -1.57159977 -0.40042355
F 1.14060426 -0.78999691 -1.64268053
F 0.74952458 -2.39064896 -0.20673066
H -0.61674712 0.60340088 -0.06863214
H 1.37074997 -0.71609590 1.65593239
O -0.28023994 0.02077617 0.72436115

h1m3-6.xyz
30
-1137.61422129876
C 4.58035159 1.41244274 -0.61097535
H 3.45045542 -0.21315802 -0.35678385
H 5.47170561 0.89339819 -0.99708529
H 3.99945292 1.80655683 -1.46088224
H 4.90726613 2.25319125 0.01362446
O 3.79773868 0.53971391 0.21196001
C 2.60372263 -2.66601180 -0.63379655
H 1.66771530 -0.99532992 -1.15703916
H 2.25426449 -2.65067767 0.41099937
H 1.97828971 -3.36610698 -1.20910620
H 3.64132464 -3.02072638 -0.65639721
O 2.58768353 -1.35919264 -1.22312651
C 1.28654760 1.28544229 2.42979985
H 2.40630295 1.16910315 0.76814122
H 0.25445585 1.56527271 2.67019619
H 1.45959131 0.24714437 2.75543835
H 1.97210857 1.95186907 2.97496953
O 1.46104423 1.43794221 1.01625748
C -1.44927253 -1.24435383 0.41453247
C -1.90930826 1.07274564 -0.65106680
C -1.08921069 -0.23863521 -0.70613263
F -2.76194845 -1.56811480 0.42145161
F -0.74783722 -2.39237946 0.21667009
F -1.12467990 -0.78258078 1.64616619
F -3.23929174 0.83984282 -0.76465220
F -1.70350666 1.75887982 0.49943393
F -1.54409699 1.87341646 -1.67783563
H 0.61492877 0.60438091 0.06181163
H -1.37237333 -0.72930056 -1.65145326
O 0.27913292 0.01782184 -0.72871396

h1m3-7.xyz
30
-1137.61431859600
C 3.50182874 1.51985803 -0.92533384
H 3.23104341 -0.06093659 0.26087024
H 2.55646669 2.02310559 -0.66575853
H 3.71004637 1.68251509 -1.99046094
H 4.31732092 1.96813495 -0.33648655
O 3.43389035 0.10569501 -0.70916493
C 3.05541414 -1.75489556 2.24115996
H 1.62588510 -0.59089438 1.51857883
H 4.12781036 -1.64637832 2.44354202
H 2.91120511 -2.55752444 1.50003087
H 2.54554058 -2.03342810 3.17673066
O 2.58291592 -0.49019066 1.75625354
C 0.87718643 -0.94421918 -2.89280817
H 2.15196611 -0.66905544 -1.35420400
H 0.62261813 0.11406862 -3.06298212
H -0.01462764 -1.55487058 -3.07570367
H 1.66409465 -1.24642563 -3.60095030
O 1.30024867 -1.18156571 -1.54735006
C -2.26276947 -0.71309548 0.08396921
C -0.75642287 1.37808606 0.34928957
C -1.04232330 -0.07769527 0.79235515
F -3.38052176 0.03975161 0.21710133
F -2.51540809 -1.92478453 0.63022430
F -2.04186172 -0.89964119 -1.24097834
F -1.78385974 2.21008536 0.63301239
F -0.49258355 1.47763696 -0.97761704
F 0.33552437 1.83599545 1.01326044
H 0.42059740 -0.97381150 -0.31021805
H -1.31217094 -0.01546155 1.85882830
O 0.08216714 -0.89167400 0.66668855

h1m3-8.xyz
30
-1137.61432611109
C -0.85972759 1.29592638 2.75176193
H -2.14385778 0.83545865 1.26687810
H -1.64407686 1.67669132 3.42383881
H -0.59832126 0.26665225 3.04510214
H 0.02982375 1.92816997 2.85465742
O -1.29212098 1.36919063 1.39028600
C -3.06732504 1.45812182 -2.43107739
H -1.63583288 0.38952713 -1.57757609
H -2.91573873 2.34754424 -1.79847833
H -2.56328364 1.61387566 -3.39777118
H -4.14139179 1.32853218 -2.61096779
O -2.59493737 0.26320089 -1.79296598
C -3.48166985 -1.39407774 1.12644231
H -3.23258060 0.02730281 -0.25027630
H -4.29954575 -1.91774824 0.60731064
H -2.53492797 -1.91957066 0.92135178
H -3.67750453 -1.42355851 2.20581735

O -3.42707251 -0.01745624 0.73453206
C 2.25513340 0.70246975 -0.16261138
C 0.75998385 -1.41337283 -0.19649114
C 1.04282217 -0.01656604 -0.80164764
F 3.37906378 -0.05138827 -0.20690062
F 2.50190585 1.84878660 -0.83734810
F 2.02552895 1.03169357 1.13297654
F 1.79700259 -2.26436711 -0.36626621
F 0.47618297 -1.35974661 1.12906143
F -0.31867908 -1.95434345 -0.81823368
H -0.42111235 1.00508411 0.18353926
H 1.32006131 -0.19887773 -1.85234811
O -0.08694964 0.79954139 -0.77669252

h1m4-1.xyz
36
-1253.41489822373
C -4.12485380 -0.34602332 1.66019241
H -3.03560859 -1.22630645 0.23973167
H -4.53579674 -1.28627511 2.06088542
H -3.28485498 -0.02614560 2.29937474
H -4.90982026 0.42045460 1.69847477
O -3.73212397 -0.50017449 0.29484784
C -2.58795421 2.67133040 -0.12253715
H -3.17323399 0.79997590 -0.50416770
H -3.56966192 3.05227582 0.20161894
H -1.99205738 2.40969904 0.76655057
H -2.06994195 3.47109318 -0.66747104
O -2.74178228 1.56229955 -1.01167400
C -2.12507769 -3.28221962 -0.99831185
H -1.00476516 -1.93875235 -0.06845232
H -2.12878550 -2.75438944 -1.96549354
H -1.36924738 -4.08247082 -1.02809458
H -3.11167243 -3.73334717 -0.83635774
O -1.87838611 -2.38636048 0.09421027
C 0.09156842 1.53703216 -2.88027704
H -1.42416938 0.99146446 -1.66518379
H -0.59349246 1.98013499 -3.61878300
H 0.52710053 2.33833315 -2.26166653
H 0.90159802 1.02411566 -3.41277138
O -0.59691232 0.56775585 -2.08601783
C 2.72763979 -0.41442754 -0.16400555
C 0.81193174 0.50759742 1.30698082
C 1.33948994 -0.68166346 0.46721246
F 3.63235215 0.01429089 0.74894557
F 3.20070730 -1.56139631 -0.70488127
F 2.66632872 0.50898480 -1.15422361
F 1.56502776 0.71857289 2.41099576
F 0.76580117 1.66819633 0.60726610
F -0.45233631 0.23548600 1.72249123
H 0.11931725 -0.32607778 -1.14464917
H 1.48800023 -1.50594469 1.18371232
O 0.43129035 -1.09360052 -0.50439269

h1m4-2.xyz
36
-1253.41490423729
C 4.16970319 -0.37169586 -1.62504770
H 3.04754205 -1.23313917 -0.21902819
H 4.58802876 -1.31642194 -2.00724640
H 3.34335656 -0.05805045 -2.28475522
H 4.95604884 0.39371490 -1.65513647
O 3.74870747 -0.51104504 -0.26656671
C 2.11192204 -3.29638851 0.99113765
H 1.01010475 -1.92924380 0.07312125
H 2.11281333 -2.78153473 1.96525156
H 1.34994385 -4.09122350 1.00501017
H 3.09623133 -3.75286732 0.83023305
O 1.87974408 -2.38432716 -0.09101113
C -0.10262697 1.58787824 2.84590865
H 1.42424175 1.01440214 1.65824640
H 0.57524807 2.04789504 3.58063170
H -0.53149858 2.37451465 2.20425294
H -0.91822357 1.08767396 3.38188910
O 0.59348073 0.60001598 2.08153928
C 2.59470376 2.66114569 0.08593298
H 3.18149684 0.80010121 0.50968502
H 2.07021289 3.47040567 0.61011963
H 3.57764204 3.03834082 -0.23895218
H 2.00620622 2.37900298 -0.80180728
O 2.74542865 1.57132657 0.99926188
C -2.72546788 -0.42894884 0.17070043
C -0.82429607 0.51315064 -1.30824121
C -1.33569171 -0.67958485 -0.46376755
F -3.64229892 -0.03020612 -0.74374961
F -3.17435824 -1.57689939 0.72972160
F -2.67708235 0.50925556 1.14760651
F -1.59319041 0.72530202 -2.40083278
F -0.77135333 1.67198675 -0.60587189
F 0.43477269 0.24565256 -1.74206228
H -0.11385483 -0.30346688 1.14337135
H -1.47577725 -1.50778401 -1.17749935
O -0.41937981 -1.07667465 0.50628368

h1m4-3.xyz
36
-1253.41489527547
C -4.14550657 0.36275519 1.64875794
H -3.04031989 1.23153371 0.23307819
H -3.31182566 0.04586613 2.29763351
H -4.93152857 -0.40276985 1.68429737
H -4.55930309 1.30569495 2.04017419
O -3.74008736 0.50846409 0.28633312
C -2.59312933 -2.66308305 -0.10432916
H -3.17710089 -0.79565000 -0.50398531
H -3.57649492 -3.04400445 0.21501516
H -2.00518858 -2.39274697 0.78743144
H -2.06869231 -3.46583227 -0.63852920
O -2.74272070 -1.56153048 -1.00349789
C -2.12275267 3.28674186 -1.00048118
H -1.00600773 1.93514689 -0.07749051
H -1.36879936 4.08900846 -1.02337153
H -3.11107380 3.73454497 -0.83979123
H -2.12179708 2.76424805 -1.97057661
O -1.87776867 2.38550393 0.08797217
C 0.10058113 -1.57086669 -2.85674709
H -1.42271995 -1.00188278 -1.66191062
H -0.57958291 -2.02671236 -3.59198782
H 0.53043806 -2.36103872 -2.22013422
H 0.91520873 -1.06875299 -3.39242625
O -0.59272983 -0.58625656 -2.08567009
C 2.72916872 0.42485271 -0.16049454
C 0.81359236 -0.51410178 1.29981621
C 1.33572065 0.68042127 0.46412419

F 3.63475725 0.01228011 0.75911236
F 3.19177702 1.57346696 -0.70657654
F 2.68251893 -0.50541058 -1.14498124
F 1.56536230 -0.72333537 2.40499785
F 0.77602260 -1.67329772 0.59737735
F -0.45336207 -0.25058916 1.71301430
H 0.11925416 0.31537624 -1.14896504
H 1.47420119 1.50480775 1.18250070
O 0.42858922 1.08625461 -0.51104288

h1m4-4.xyz
36
-1253.41488803325
C -2.58602014 2.67249947 -0.10972836
H -3.16528875 0.80096236 -0.49878045
H -3.57005326 3.05390052 0.20684496
H -1.99797747 2.40810357 0.78371341
H -2.06242875 3.47277706 -0.64844141
O -2.73366230 1.56553208 -1.00239081
C -4.11598179 -0.35784264 1.66164993
H -3.03086044 -1.22944905 0.23305419
H -4.53460382 -1.29822295 2.05421264
H -3.27266897 -0.05123382 2.30285198
H -4.89432928 0.41484597 1.70797730
O -3.72599108 -0.50279536 0.29459985
C -2.13118899 -3.28866859 -1.01118180
H -1.00206072 -1.94442565 -0.09176570
H -2.12908050 -2.76533344 -1.98079281
H -1.38406257 -4.09728879 -1.03717651
H -3.12261911 -3.72855847 -0.84779642
O -1.87526631 -2.39059884 0.07723002
C 0.10507565 1.56491201 -2.86522483
H -1.41670996 1.00307565 -1.66492075
H -0.57652891 2.02567591 -3.59604663
H 0.54323487 2.35187784 -2.23026538
H 0.91351396 1.05849443 -3.40617326
O -0.58994631 0.58381308 -2.09125030
C 2.72711044 -0.42004014 -0.15598773
C 0.80297207 0.50787829 1.29974532
C 1.33208955 -0.68267375 0.46240364
F 3.62621175 -0.00054026 0.76690440
F 3.19860214 -1.56713884 -0.69787851
F 2.67951932 0.50811674 -1.14231006
F 1.54666643 0.71219858 2.41125529
F 0.76978354 1.67047768 0.60236230
F -0.46669731 0.24205960 1.70258789
H 0.12107739 -0.31818305 -1.15486493
H 1.47127257 -1.50832954 1.17926864
O 0.43063565 -1.08942151 -0.51729823

h1m4-5.xyz
36
-1253.41489920210
C 0.10466597 -1.54864071 -2.87213153
H -1.41602333 -0.99191071 -1.66875067
H -0.57746067 -2.00285363 -3.60658626
H 0.54036023 -2.34092249 -2.24213107
H 0.91480491 -1.04062915 -3.40896974
O -0.58863101 -0.57105177 -2.09218831
C -4.12753327 0.34604279 1.65822999
H -3.03942102 1.22877395 0.23784571
H -4.55141292 1.28248033 2.05460870
H -3.28531275 0.03913258 2.30075807
H -4.90297665 -0.42998180 1.69738596
O -3.73325371 0.50020067 0.29344211
C -2.11635495 3.27616796 -1.01229250
H -1.00870183 1.93060282 -0.07110379
H -2.10726076 2.74467658 -1.97736387
H -1.36257970 4.07859431 -1.03651076
H -3.10608424 3.72495660 -0.86353204
O -1.87953537 2.38537295 0.08637476
C -2.58169824 -2.66839441 -0.12108653
H -3.16786916 -0.79797378 -0.50512827
H -1.99728345 -2.40379835 0.77469177
H -2.05260275 -3.46432105 -0.66089642
H -3.56444701 -3.05592048 0.19204445
O -2.73291357 -1.55941572 -1.01074754
C 2.72726708 0.41386586 -0.15861910
C 0.80427112 -0.50386834 1.30609560
C 1.33656549 0.68316167 0.46606510
F 3.63066807 -0.00233604 0.76124491
F 3.19810589 1.55674369 -0.70991251
F 2.67184708 -0.51990251 -1.13942314
F 1.55616996 -0.71705281 2.41024315
F 0.75301547 -1.66497788 0.60739128
F -0.45916946 -0.22643768 1.72105636
H 0.12302251 0.32559594 -1.15047010
H 1.48289828 1.50844948 1.18190071
O 0.43233963 1.09403683 -0.50956054

h1m4-6.xyz
36
-1253.41490352831
C 4.12851032 0.36015255 1.65525540
H 3.03370958 1.23411980 0.23503407
H 4.90484958 -0.41495136 1.69537484
H 4.55265426 1.29864879 2.04640321
H 3.28842527 0.05496182 2.30132318
O 3.73020274 0.50818362 0.29091225
C 2.59376114 -2.66801567 -0.10903234
H 3.16998798 -0.79640396 -0.50187458
H 3.57873570 -3.04526927 0.20964803
H 2.00367774 -2.40421604 0.78326271
H 2.07394211 -3.47126830 -0.64699209
O 2.73851294 -1.56226509 -1.00362632
C 2.11962820 3.28901751 -1.00453593
H 1.00226844 1.93921225 -0.07995649
H 2.12691520 2.76245440 -1.97241669
H 1.36173334 4.08730435 -1.03514426
H 3.10467835 3.74262067 -0.84011026
O 1.87303111 2.39122208 0.08632338
C -0.09903201 -1.56204737 -2.86655376
H 1.41931938 -1.00026587 -1.66225278
H -0.90860822 -1.05602509 -3.40622461
H 0.58492661 -2.01724901 -3.59867831
H -0.53519115 -2.35310688 -2.23538675
O 0.59172686 -0.58185300 -2.08767575
C -2.72827178 0.42014262 -0.15723702
C -0.80653964 -0.51248563 1.29919638
C -1.33441777 0.68025476 0.46453666
F -3.62772965 -0.00908779 0.76065644
F -3.20140852 1.57095263 -0.68984431
F -2.67762821 -0.49951518 -1.15129557
F -1.55351341 -0.72173433 2.40767282

F -0.76979985 -1.67242417 0.59759063
F 0.46135220 -0.24634988 1.70716954
H -0.12109075 0.31904877 -1.15127384
H -1.47430099 1.50395134 1.18343568
O -0.43119101 1.08925539 -0.51281565

h1m4-7.xyz
36
-1253.41455219636
C 1.94347982 -2.74339270 -1.54024944
H 2.64523494 -0.93027424 -1.12831756
H 2.47648364 -2.91955392 -2.48774318
H 0.92965055 -2.37154008 -1.74880535
H 1.87431738 -3.69272651 -0.99578781
O 2.68032621 -1.82879135 -0.70933833
C 2.65549721 1.69087123 -2.37219871
H 2.36465977 1.16191026 -0.44042029
H 3.73364889 1.87219747 -2.24828256
H 2.11540265 2.64879658 -2.32778290
H 2.47991630 1.23037158 -3.35180090
O 2.17366195 0.78439595 -1.36925921
C 1.76435133 -1.85705730 2.71332743
H 2.04187250 -1.44044059 0.76471576
H 1.38746060 -1.31373843 3.58876712
H 2.80291338 -2.16991775 2.90772014
H 1.13987508 -2.75092816 2.56039344
O 1.68140105 -0.97899240 1.58703499
C 2.01659688 2.47898016 1.81561053
H 2.36127984 0.53820514 1.46838233
H 2.26551906 3.42285175 1.31535926
H 2.38164382 2.52468102 2.85353311
H 0.92216473 2.36157484 1.82132516
O 2.66748952 1.42176868 1.09915435
C -1.87224549 -1.17716470 -0.00126161
C -1.58464275 1.38395888 -0.00618526
C -0.90970213 0.00665010 -0.20761504
F -2.52109880 -1.09081672 1.18806392
F -1.16511471 -2.33875791 0.00888775
F -2.79982434 -1.27693430 -0.97511550
F -1.91675555 1.60007159 1.29370486
F -2.69313132 1.55843210 -0.75401460
F -0.69923033 2.36122109 -0.35627975
H 0.54507532 0.29858188 -1.47747449
H -0.14826153 -0.09113856 0.58947346
O -0.37169773 -0.09888232 -1.49494077

h1m4-8.xyz
36
-1253.41364411084
C 0.75279860 -2.96632359 -1.33403873
H 1.23487783 -1.62836362 0.10048698
H -0.10034963 -3.42869131 -0.81533300
H 0.54192928 -2.93629053 -2.40974116
H 1.65654740 -3.56760198 -1.15815792
O 0.94936965 -1.61488094 -0.88615457
C 2.58115637 1.08013467 -2.41401421
H 2.28330265 -0.67514259 -1.53585967
H 3.42158268 1.77940750 -2.50520701
H 2.25086613 0.79345778 -3.42473302
H 1.74756927 1.58329338 -1.89969931
O 3.05201800 -0.06153397 -1.68530511
C 3.58208843 1.51008617 1.35280463
H 3.61058218 0.15960512 -0.10761464
H 4.32795608 2.19283814 0.91507364
H 2.57245175 1.88837380 1.12280251
H 3.71636485 1.50399384 2.44203710
O 3.77556149 0.17225909 0.88361820
C 1.24031572 -1.40525958 2.70649966
H 2.67847588 -0.94349301 1.37679925
H 0.79166072 -0.40015540 2.74122207
H 0.43701328 -2.14896925 2.77360876
H 1.91139955 -1.53380204 3.57016615
O 1.94672516 -1.63426406 1.48353486
C -2.55172217 -0.52042366 0.04996697
C -1.03937284 1.57810865 0.08177411
C -1.80885585 0.55155719 -0.78548666
F -3.51126100 0.01955394 0.83905898
F -3.15414329 -1.40233532 -0.78041471
F -1.70864724 -1.22796296 0.84972890
F -1.79355146 2.11662970 1.06761550
F 0.06153702 1.02429068 0.65991519
F -0.60585032 2.59530348 -0.70304149
H -0.27929316 -0.60077622 -1.31145136
H -2.59599191 1.12365614 -1.30269363
O -0.97614022 -0.01287490 -1.74376428

h1m5-1.xyz
42
-1369.21614704113
C -0.96731823 2.67149990 -2.22330480
H -1.84789165 0.99400277 -1.62674704
H 0.02654244 2.20060017 -2.25725002
H -1.38307772 2.73047971 -3.24164059
H -0.86880765 3.68981433 -1.82781002
O -1.85938870 1.95460687 -1.35345344
C -2.53615507 -2.99864994 0.66475374
H -3.13829240 -1.08366015 0.72828128
H -2.28301434 -3.72588175 -0.11679183
H -1.64624020 -2.82809397 1.28919311
H -3.34182959 -3.41600322 1.28797783
O -2.97389984 -1.79504713 0.02101836
C -4.46052186 0.91590874 1.54407312
H -2.48850499 0.68699878 1.64920627
H -4.59190472 1.65724292 2.34847029
H -4.40156055 1.44232039 0.57716366
H -5.33461327 0.25287872 1.53401524
O -3.30148188 0.10744711 1.78064857
C -0.78665711 2.72742654 1.90318797
H -1.40067144 1.83018762 0.20829150
H 0.12849193 3.16856764 1.48046679
H -1.58202356 3.49041705 1.92051047
H -0.57936852 2.40822457 2.93192127
O -1.18437301 1.56863511 1.16096221
C -1.90917700 -1.29835780 -3.08619945
H -2.10659016 -1.17968283 -1.07602835
H -1.55297082 -2.33903097 -3.12149469
H -2.99481989 -1.27937121 -3.26349553
H -1.40524796 -0.72632557 -3.87506405
O -1.58931123 -0.69156886 -1.82485811
C 1.75903012 -1.52065858 0.44946797
C 2.41433957 0.94110245 0.05857499
C 1.33497015 -0.14310952 -0.11064769
F 1.90476700 -1.50063042 1.79866932
F 0.77729272 -2.42482447 0.16740576

F 2.90451220 -1.99099462 -0.08680771
F 2.80995665 1.05866209 1.35155977
F 3.51079859 0.71823557 -0.69380488
F 1.90444478 2.14810016 -0.31502692
H 0.07306175 -0.48520321 -1.57010493
H 0.47698225 0.19339292 0.50274593
O 1.03625213 -0.23557997 -1.47453314

h1m5-2.xyz
42
-1369.21579258810
C -0.73259225 -3.48151330 1.57131459
H -1.52463383 -1.64405619 1.53042317
H 0.30899112 -3.13644498 1.47991925
H -0.95236631 -3.69849203 2.62823606
H -0.84945047 -4.40826249 0.99562200
O -1.66251552 -2.52314990 1.05052697
C -2.53392399 1.32667004 -2.49365634
H -2.59344799 -0.41050670 -1.53958339
H -1.46574109 1.48988255 -2.28454009
H -2.64735671 0.94246938 -3.51991475
H -3.06909127 2.28155763 -2.42099311
O -3.13097643 0.42698571 -1.54506181
C -3.02148012 2.71999751 0.99335061
H -3.17330392 0.99065658 0.00371601
H -3.90133977 3.18313943 0.51963811
H -2.99113261 3.03261194 2.04505654
H -2.10880238 3.07850272 0.49112873
O -3.12352753 1.29261542 0.96545202
C -0.98499183 0.18940199 3.38294105
H -1.94945571 0.47421487 1.63756954
H -0.18417456 -0.46358978 3.75233367
H -0.66263429 1.23550408 3.50280661
H -1.89134412 0.01997788 3.98672841
O -1.20651071 -0.12111041 2.00427553
C -1.46858680 -2.79929244 -2.34353372
H -1.52320066 -2.10792293 -0.44216219
H -1.42669781 -2.36151112 -3.34821607
H -0.59597817 -3.45601939 -2.20694081
H -2.39150256 -3.39071000 -2.24825141
O -1.45311480 -1.72397661 -1.39243719
C 2.30441253 -0.74806629 -0.03097547
C 1.47972496 1.69029980 -0.16179757
C 1.11387338 0.20177263 -0.29333871
F 2.72883529 -0.69213826 1.25837796
F 1.89628659 -2.03083512 -0.25869059
F 3.36667368 -0.51752976 -0.82918233
F 2.03884305 1.96621016 1.04424173
F 2.32862828 2.11523817 -1.12001027
F 0.34744300 2.44064040 -0.26051156
H -0.12519760 -0.68879112 -1.51548430
H 0.37960191 0.00550811 0.51192322
O 0.59981433 -0.00285235 -1.57879491

h1m5-3.xyz
42
-1369.21582218157
C 2.90251876 -2.76587619 1.13199055
H 3.12689720 -1.08501515 0.07462840
H 2.86572184 -3.03549613 2.19533090
H 1.97128306 -3.10231685 0.64888257
H 3.75752198 -3.28602505 0.67223622
O 3.06793929 -1.34650399 1.04763275
C 2.49479148 -1.48187459 -2.42471421
H 2.59401683 0.28117943 -1.52290259
H 1.41571910 -1.59387846 -2.23991760
H 2.65157083 -1.14001181 -3.46024921
H 2.98695100 -2.45482145 -2.30369082
O 3.10335320 -0.57353532 -1.49215597
C 1.60018209 2.67029360 -2.44403972
H 1.58606549 2.05988949 -0.51413665
H 2.55175932 3.21503622 -2.35254770
H 1.55572540 2.19173157 -3.42988132
H 0.76211787 3.37828157 -2.35604771
O 1.50622753 1.64011902 -1.44857582
C 0.85564022 3.54305344 1.44360661
H 1.56650967 1.67179144 1.47172999
H -0.20026271 3.24278254 1.35944352
H 1.08211978 3.78614027 2.49340387
H 1.01690086 4.44308879 0.83702510
O 1.74212923 2.52585226 0.96029775
C 0.92259804 -0.06942922 3.38077346
H 1.92104522 -0.45670239 1.67313033
H 0.56197686 -1.09880559 3.53199880
H 1.81754343 0.09413822 4.00290695
H 0.13460706 0.62352164 3.70157840
O 1.19177580 0.17876347 1.99793880
C -2.27787511 0.79402553 -0.01436161
C -1.51530962 -1.66249838 -0.16928524
C -1.11622632 -0.18284177 -0.30705391
F -2.66552927 0.74869264 1.28715822
F -1.84927827 2.06704497 -0.25630333
F -3.36794353 0.58567753 -0.78077657
F -2.05405498 -1.92763038 1.04847291
F -2.39493612 -2.06496662 -1.10914005
F -0.40414650 -2.44063027 -0.29431180
H 0.12788667 0.66605674 -1.55363871
H -0.36077831 -0.00325790 0.48244019
O -0.62677114 0.01215125 -1.60362175

h1m5-4.xyz
42
-1369.21582796440
C -2.94123223 2.75324868 1.08149159
H -3.14049836 1.05238143 0.05130913
H -2.02065935 3.09977952 0.58537984
H -3.80976289 3.25020595 0.62139065
H -2.90055675 3.03968578 2.14026965
O -3.08047692 1.33000349 1.01980497
C -2.51424571 1.41844246 -2.45213757
H -2.59813255 -0.33131891 -1.52404679
H -2.66071107 1.05765560 -3.48277819
H -3.01914319 2.38702061 -2.35002501
H -1.43772558 1.54696314 -2.26313260
O -3.11694925 0.51803386 -1.50806712
C -1.56759462 -2.72522920 -2.39879645
H -1.57055853 -2.07981678 -0.48054277
H -0.71971749 -3.41912969 -2.29392513
H -2.51151507 -3.28222133 -2.30152921
H -1.52543056 -2.26442029 -3.39310800
O -1.49359396 -1.67559425 -1.42204023
C -0.81590235 -3.52257097 1.49531037
H -1.55144278 -1.66086463 1.50000240
H -1.03468350 -3.75388438 2.54939770

H	-0.96742940	-4.43319265	0.90218682
H	0.23547779	-3.20926202	1.40233810
O	-1.71817424	-2.52441698	1.00153857
C	-0.93169397	0.12080424	3.38151415
H	-1.92291292	0.46591783	1.66108119
H	-0.57732877	1.15489011	3.51474328
H	-1.83008083	-0.03504593	4.00061415
H	-0.14234402	-0.56138393	3.72177798
O	-1.18963996	-0.15630161	2.00214460
C	2.29975983	-0.77245067	-0.02288364
C	1.49187738	1.67031982	-0.17049474
C	1.11827790	0.18356160	-0.30300001
F	2.71635479	-0.70557119	1.26846951
F	1.88346976	-2.05447138	-0.23951470
F	3.36878847	-0.55832767	-0.81670330
F	2.02715664	1.94881660	1.04575140
F	2.36345521	2.08519899	-1.11266214
F	0.36699129	2.42807749	-0.29652811
H	-0.12729342	-0.68485534	-1.53565960
H	0.37359240	-0.00715669	0.49395517
O	0.61934307	-0.02261541	-1.59428035

h1m5-6.xyz
42
-1369.21583620945

C	-0.90615603	-3.56362760	-1.40539847
H	-1.57565601	-1.67803431	-1.46439736
H	0.15530700	-3.28456311	-1.31928038
H	-1.13182641	-3.81756541	-2.45282814
H	-1.09037578	-4.45071036	-0.78648570
O	-1.77344599	-2.52059829	-0.94274642
C	-2.82000591	2.79585710	-1.19271602
H	-3.09246119	1.13282835	-0.11795666
H	-1.88738818	3.11724282	-0.70224229
H	-3.66843491	3.34083710	-0.74983171
H	-2.76449104	3.05141070	-2.25873134
O	-3.01859425	1.38162953	-1.09316928
C	-0.85179741	0.04098979	-3.37746187
H	-1.88743297	0.45842371	-1.69768876
H	-0.46488286	1.06188847	-3.52119837
H	-1.73110283	-0.10611892	-4.02543389
H	-0.06936788	-0.66951713	-3.67247893
O	-1.16685478	-0.19635441	-2.00266210
C	-1.67019255	-2.62516553	2.46388993
H	-1.62038017	-2.03903530	0.52738015
H	-0.84926868	-3.35480640	2.39230561
H	-2.63407328	-3.14751599	2.36980758
H	-1.62302367	-2.13622688	3.44444844
O	-1.54159727	-1.60964717	1.45734454
C	-2.49508484	1.53551491	2.39462177
H	-2.60426468	-0.23020788	1.49983464
H	-1.40972751	1.62464549	2.23609773
H	-2.68491302	1.20460726	3.42824730
H	-2.96389143	2.51740374	2.25451967
O	-3.09817558	0.63280123	1.45338874
C	2.26064214	-0.81572697	0.01722620
C	1.52333453	1.64738298	0.18381830
C	1.11041669	0.17145301	0.31889782
F	2.65306024	-0.76006726	-1.28244300
F	1.81534261	-2.08603308	0.24335544
F	3.35068300	-0.62974780	0.78912554
F	2.04379788	1.91477714	-1.04154821
F	2.42338083	2.03433899	1.11071338
F	0.42308495	2.43679413	0.33265940
H	-0.14186399	-0.66262534	1.56681701
H	0.34995019	0.00094199	-0.46774892
O	0.62589852	-0.02460427	1.61734153

h1m5-7.xyz
42
-1369.21582976396

C	0.93227484	-0.08836286	-3.38089905
H	1.92480701	-0.45549801	-1.66571395
H	0.57132110	-1.11893045	-3.52327715
H	1.83080467	0.06780068	-3.99972210
H	0.14677194	0.60197508	-3.71334307
O	1.19402583	0.17385361	-1.99924991
C	2.92610491	-2.75516258	-1.10984546
H	3.12702702	-1.06700095	-0.05861014
H	1.99698007	-3.10061471	-0.62925451
H	3.78519665	-3.26329135	-0.64429410
H	2.89721316	-3.02945623	-2.17223886
O	3.07449531	-1.33362370	-1.03063812
C	2.49365781	-1.44614194	2.44454146
H	2.58282406	0.30912631	1.52547401
H	2.98586948	-2.41955196	2.32703939
H	1.41383703	-1.56048653	2.26595033
H	2.65594390	-1.09752684	3.47701176
O	3.09632360	-0.54334662	1.50298789
C	0.82520109	3.53570814	-1.46836936
H	1.55881822	1.67329142	-1.48520893
H	-0.22661758	3.22338963	-1.37711029
H	1.04431617	3.77278337	-2.52107553
H	0.97827058	4.44254993	-0.86988036
O	1.72605725	2.53327610	-0.98062682
C	1.58874300	2.70520777	2.42112985
H	1.57444118	2.07769455	0.49656007
H	0.75037067	3.41213823	2.32767835
H	2.54003449	3.24943686	2.32368006
H	1.54554827	2.23559965	3.41128810
O	1.49418676	1.66613600	1.43490263
C	-2.29889987	0.77351516	0.01745805
C	-1.48752144	-1.66810653	0.16866631
C	-1.11756454	-0.18079135	0.30472899
F	-2.70576229	0.70842906	-1.27725181
F	-1.88687444	2.05545246	0.23968933
F	-3.37374868	0.55570570	0.80252577
F	-2.01485099	-1.94643336	-1.05089317
F	-2.36378187	-2.08532681	1.10536372
F	-0.36203604	-2.42435365	0.30065999
H	0.12112642	0.68548187	1.54538734
H	-0.36833969	0.01232655	-0.48745456
O	-0.62787919	0.02561448	1.59955610

h1m5-8.xyz
42
-1369.21583438420

C	1.65165152	-2.65779704	2.44820715
H	1.61162077	-2.05760629	0.51551314
H	0.83230352	-3.38823284	2.36699480
H	2.61688751	-3.17782040	2.35554300
H	1.59837913	-2.17596723	3.43200756
O	1.52676568	-1.63525325	1.44833293

C 0.88404895 -3.55947682 -1.42295484
H 1.57708032 -1.68253505 -1.47212963
H -0.17365695 -3.26936979 -1.32610503
H 1.09896115 -3.80541680 -2.47448768
H 1.06294684 -4.45477927 -0.81429862
O 1.76652816 -2.53091462 -0.95638101
C 2.85069765 2.78449020 -1.17025272
H 3.08785549 1.11691289 -0.09295078
H 3.70206607 3.31591124 -0.71667290
H 2.81267919 3.04056716 -2.23688397
H 1.91721062 3.12055102 -0.69159884
O 3.02563899 1.36740450 -1.06858672
C 0.87954445 0.04795703 -3.38049215
H 1.89337659 0.45392324 -1.68583440
H 0.49740003 1.07065469 -3.52417477
H 1.76762093 -0.09808164 -4.01665181
H 0.09963504 -0.65900360 -3.69037186
O 1.17452484 -0.19734760 -2.00245014
C 2.47430184 1.50534982 2.42028345
H 2.58746232 -0.25345384 1.51179844
H 1.38898961 1.59157878 2.26063897
H 2.66414850 1.16955414 3.45230561
H 2.93984185 2.48961350 2.28591543
O 3.08172737 0.60966716 1.47492412
C -2.28703635 -0.79363059 0.01793499
C -1.49207886 1.65271710 0.17624999
C -1.11352380 0.16753714 0.31241750
F -2.69155047 -0.72391490 -1.27727064
F -1.86642026 -2.07392362 0.23416241
F -3.36485044 -0.58743793 0.80196665
F -2.01188436 1.93125124 -1.04670551
F -2.37752885 2.06291758 1.10717350
F -0.37192950 2.41536125 0.31825815
H 0.13262740 -0.68294262 1.55664566
H -0.35987499 -0.02078768 -0.47661822
O -0.62925035 -0.03797758 1.60949450

h2m1-1.xyz
30
-1696.23969936664
C 0.86430400 3.65165752 -0.05929598
H 2.52823867 2.87266729 0.69393839
H 1.12735442 4.70354097 0.12098850
H -0.20182366 3.50659828 0.14184808
H 1.07464665 3.38408997 -1.10511614
O 1.57315485 2.78979798 0.85911947
C -2.60249589 -1.53238214 -0.10119033
C -2.93973276 1.02294155 0.13820857
C -2.70605681 -0.32555235 0.86333900
F -3.64138752 -1.60278714 -0.96542342
F -2.58575421 -2.67986994 0.61684781
F -1.45662120 -1.49952094 -0.83426294
F -4.12186748 1.04790267 -0.52196188
F -1.95860508 1.31075147 -0.75592304
F -2.96015246 2.02686618 1.04878894
H -0.77917652 -0.08405868 1.23027872
H -3.61037525 -0.49900112 1.46770433
O -1.61685301 -0.27716779 1.72981185
C 2.83827697 -0.82756016 0.98098969
C 2.15047213 -0.09949135 -1.40870217
C 1.67846779 -0.56614258 -0.01013611
F 3.75335494 -1.69158821 0.48891836
F 2.34179799 -1.35089460 2.12234077
F 3.48713691 0.32390697 1.30894328
F 2.91583900 -1.02867428 -2.02006037
F 2.87355043 1.05664868 -1.34584676
F 1.07953515 0.14126324 -2.19340744
H 1.13980424 1.24941452 0.67927298
H 1.17560925 -1.53253130 -0.16556307
O 0.75154693 0.32264534 0.53833303

h2m1-6.xyz
30
-1696.23969020703
C -0.99805212 -3.69283455 0.10846852
H -2.58884773 -2.86155110 -0.74138171
H 0.08226479 -3.57534559 -0.02366971
H -1.26755659 -3.42340084 1.14016808
H -1.27693435 -4.73660179 -0.09374591
O -1.62344263 -2.80887988 -0.84869116
C 2.56518842 1.52233043 0.15356075
C 2.99476171 -1.01387185 -0.15667669
C 2.70338685 0.34413457 -0.84147700
F 3.63145146 1.63870969 0.97819693
F 2.45168444 2.68102375 -0.53752191
F 1.45329480 1.40645949 0.92947302
F 4.15513378 -0.99782731 0.54144196
F 2.00376756 -1.38842982 0.69358929
F 3.10038627 -1.97946932 -1.10121447
H 0.78175931 0.04336023 -1.19118229
H 3.59312361 0.56801437 -1.45099874
O 1.60660230 0.27249867 -1.69718327
C -2.74608401 0.85312045 -1.02471891
C -2.22252488 0.09631728 1.39797132
C -1.65562265 0.54672984 0.02985952
F -3.65169883 1.75411801 -0.58416543
F -2.16495851 1.35433589 -2.13545485
F -3.42136884 -0.27184737 -1.39005862
F -2.98243687 1.05467739 1.97021763
F -2.98673638 -1.02972321 1.29129325
F -1.20671828 -0.18896844 2.23936965
H -1.14707932 -1.28543205 -0.63764027
H -1.12651948 1.49329658 0.21692851
O -0.73194286 -0.37566242 -0.46674599

h2m2-1.xyz
36
-1812.03757348097
C -0.67837895 2.46652854 2.33162110
H 0.01059953 0.67253094 1.85395979
H 0.25083050 2.81704172 2.80680085
H -0.73049000 2.85874742 1.30424462
H -1.53395380 2.84763542 2.90152964
O -0.75683034 1.03436391 2.35933543
C 4.06215346 -1.00481799 2.87357663
H 4.50114124 0.38554035 1.52752958
H 4.09666447 -1.85565916 2.17766074
H 5.00577457 -0.93803154 3.43348704
H 3.23601760 -1.14766953 3.57806825
O 3.79593130 0.23425053 2.18057994
C -3.04768470 0.87796321 -0.61984619
C -2.67236799 -1.54537943 0.19051168
C -3.39424652 -0.19792252 0.44054936
F -3.46463479 0.52990842 -1.86078865
F -3.64636857 2.04533933 -0.29924384

```
F  -1.70544219  1.12422709 -0.69665006
F  -2.82594974 -1.99868964 -1.07866091
F  -1.33468384 -1.45583340  0.43209537
F  -3.16764446 -2.48649442  1.02395535
H  -2.22891533  0.48188776  1.90711138
H  -4.46839277 -0.40509807  0.30322733
O  -3.19921582  0.24943636  1.73803373
C   2.11367579  1.30675127 -1.05193626
C   2.25836801 -1.26883916 -0.85221618
C   1.52196634  0.02308272 -0.42289532
F   2.18460086  1.23774088 -2.39774932
F   1.33888006  2.36594094 -0.72798558
F   3.36731807  1.56860283 -0.58247494
F   2.15384871 -1.49081813 -2.17940644
F   3.58884228 -1.22886872 -0.54350339
F   1.73231090 -2.33016279 -0.20691504
H   2.37607693  0.20523554  1.38591240
H   0.49503060 -0.06857777 -0.80580412
O   1.45611730  0.13600933  0.96976816
```

h2m2-2.xyz

```
36
-1812.03756942489
C   0.67938181  2.65778035  2.11537292
H  -0.01386629  0.83156894  1.78853471
H  -0.25126674  3.04947419  2.55435045
H   1.53297665  3.08333275  2.65598069
H   0.73819172  2.96213824  1.05896200
O   0.75322857  1.23284331  2.26380173
C  -4.10689415 -0.65825757  2.94179000
H  -4.49537775  0.59655734  1.45442597
H  -5.05450476 -0.50999113  3.47889134
H  -4.15626575 -1.57562944  2.33727495
H  -3.29259556 -0.74784986  3.66848807
O  -3.80054201  0.49501307  2.12790847
C   2.66339400 -1.52486616  0.31953169
C   3.05135652  0.82286373 -0.68135679
C   3.39073801 -0.16496094  0.46392433
F   2.80728115 -2.07251191 -0.91316173
F   3.16059943 -2.40254582  1.21834623
F   1.32740701 -1.41259665  0.56147585
F   3.48431631  0.38059317 -1.88643283
F   1.70884601  1.05366783 -0.79197887
F   3.64075418  2.01521011 -0.44760594
H   2.22420043  0.63680683  1.86646251
H   4.46448176 -0.38665094  0.34760462
O   3.19344668  0.38473264  1.72110370
C  -2.26554378 -1.35102892 -0.72611638
C  -2.08973306  1.19107196 -1.17758354
C  -1.51669197 -0.03119803 -0.42215550
F  -2.17525677 -1.69383279 -2.02847610
F  -1.74040448 -2.35284474  0.00859062
F  -3.59265879 -1.27322820 -0.41028606
F  -2.14203992  0.99692469 -2.51181600
F  -3.34841562  1.50360124 -0.75348094
F  -1.31160087  2.27094725 -0.94246702
H  -2.37715012  0.34626763  1.35290852
H  -0.48904443 -0.17126484 -0.78801140
O  -1.45654885  0.21590657  0.95329700
```

h2m2-3.xyz

```
36
-1812.03757524413
C  -0.69618255 -2.61898134 -2.17178598
H   0.01535142 -0.80510468 -1.81875257
H  -0.73739080 -2.93997754 -1.11949673
H  -1.56424981 -3.02755513 -2.70230682
H   0.22190587 -3.01239085 -2.63506499
O  -0.75847119 -1.19113299 -2.29555027
C   4.10212606  0.74184203 -2.91619266
H   4.51040305 -0.53291781 -1.45132627
H   4.12749238  1.64771519 -2.29316501
H   5.05588245  0.62615028 -3.45046576
H   3.29019419  0.82787449 -3.64596685
O   3.81762945 -0.43423194 -2.12740039
C   2.09109328 -1.21784800  1.16035114
C   2.23550954  1.33188609  0.74342936
C   1.50731259  0.00613783  0.41598090
F   2.13841467 -1.03649359  2.49671251
F   1.32459800 -2.30333711  0.91287034
F   3.35351769 -1.51269366  0.73573411
F   2.11643468  1.66307485  2.04641224
F   3.56906586  1.27191155  0.45202436
F   1.71270984  2.33366027  0.00679285
H   2.38603706 -0.33179605 -1.35827349
H   0.47533375  0.12800344  0.77553711
O   1.46081841 -0.22564594 -0.96266223
C  -3.04383509 -0.83848748  0.65575783
C  -2.64149707  1.53417789 -0.27893256
C  -3.37965387  0.18417515 -0.45961928
F  -3.48100281 -0.43254818  1.87181453
F  -3.63246954 -2.02293506  0.38363941
F  -1.70167456 -1.07350010  0.76431139
F  -2.76121564  2.03878506  0.97460531
F  -1.31074418  1.42176289 -0.54671186
F  -3.14708848  2.44639989 -1.13793247
H  -2.22410805 -0.58725296 -1.88689571
H  -4.45127646  0.41007149 -0.33273723
O  -3.19039065 -0.32974991 -1.73305577
```

h2m2-5.xyz

```
36
-1812.03757637967
C  -0.67133613 -2.80123695 -1.90367814
H   0.02601335 -0.95692621 -1.72020386
H   0.25434503 -3.22979711 -2.31798725
H  -0.72197878 -3.02121530 -0.82616759
H  -1.53092446 -3.26489109 -2.40178847
O  -0.74198474 -1.39206183 -2.16319653
C   4.13907879  0.33376376 -2.99233745
H   4.50740322 -0.75497389 -1.37443683
H   4.20182969  1.31124202 -2.49231151
H   5.08451572  0.11344500 -3.50811342
H   3.32649802  0.35494084 -3.72621780
O   3.81525696 -0.71834106 -2.05727103
C  -3.03744232 -0.78873636  0.73118297
C  -2.69507119  1.49329407 -0.42596620
C  -3.39558860  0.11255052 -0.47769065
F  -3.48320766 -0.27619669  1.90302991
F  -3.59993427 -2.00691846  0.57694363
F  -1.69063876 -0.98241904  0.85895218
F  -2.85461491  2.12135441  0.76584805
F  -1.35626578  1.39107835 -0.65641678
F  -3.20609279  2.29706231 -1.38411134
```

H -2.21538587 -0.76984177 -1.81859471
H -4.47339701 0.32099280 -0.37534978
O -3.18715225 -0.51655221 -1.69514803
C 2.07762408 -1.06704386 1.28243579
C 2.27959390 1.42159852 0.60123485
C 1.52136587 0.08549111 0.41365296
F 2.09992550 -0.75844842 2.59566414
F 1.30542391 -2.16481973 1.12404943
F 3.34563453 -1.41235645 0.91477347
F 2.19595795 1.87684061 1.86923701
F 3.60477400 1.30793281 0.28938497
F 1.75763861 2.35904570 -0.21619818
H 2.39104825 -0.46682493 -1.31089375
H 0.49217949 0.26657813 0.75665636
O 1.46957416 -0.28380239 -0.93468112

h2m2-6.xyz
36
-1812.03704801526
C 0.47700034 -3.22909412 0.86204836
H -0.07642094 -1.40354231 1.39764176
H -0.44553028 -3.74214521 1.17775577
H 0.43515889 -3.04397836 -0.22222376
H 1.33224824 -3.88011706 1.07826276
O 0.66909488 -2.01803174 1.60367836
C -4.18572988 -2.52868174 1.37952625
H -4.54463039 -0.57763107 1.34431537
H -5.17940907 -2.81989374 1.74879544
H -4.13380673 -2.67163259 0.29015489
H -3.42880071 -3.15453609 1.86429634
O -3.88537452 -1.16711849 1.75017556
C 2.91504694 -0.62954058 -1.04626460
C 2.82376606 1.13619424 0.83462227
C 3.39638009 -0.22665506 0.37088611
F 3.37517870 0.21126103 -2.00277395
F 3.35662269 -1.87059558 -1.34522766
F 1.55203776 -0.65422274 -1.14874321
F 2.97178376 2.11425937 -0.09359981
F 1.49638440 1.05167100 1.12912711
F 3.45863430 1.53651895 1.95746751
H 2.18709367 -1.41825866 1.41022565
H 4.48458279 -0.07748836 0.27663793
O 3.17297783 -1.21693620 1.31515855
C -2.10860607 -0.27389553 -1.39233096
C -2.17115748 1.78748301 0.16823931
C -1.49602640 0.43457483 -0.15993939
F -1.91787535 0.42878709 -2.52703477
F -1.53193770 -1.48777168 -1.55149835
F -3.44996164 -0.48567814 -1.25544886
F -2.20351162 2.61379791 -0.89975735
F -3.45682216 1.61906765 0.59848564
F -1.49682010 2.40449945 1.15755242
H -2.41562169 -0.65102449 1.25564079
H -0.45405017 0.66445219 -0.42559190
O -1.48081684 -0.39731257 0.96498529

h2m2-7.xyz
36
-1812.03704452812
C -0.47451660 -3.23180943 0.85767760
H 0.08522009 -1.40818840 1.39427842
H -1.33090920 -3.88084375 1.07542061
H -0.43384378 -3.04750077 -0.22687842
H 0.44728188 -3.74690892 1.17223806
O -0.66279635 -2.02012970 1.59892261
C 4.20584157 -2.50829941 1.39594923
H 4.54971861 -0.55500828 1.34290045
H 4.14845244 -2.66225840 0.30830496
H 5.20446648 -2.78727268 1.76135770
H 3.45767880 -3.13636801 1.89130992
O 3.89584909 -1.14593767 1.75536602
C -2.81477241 1.13775307 0.83601019
C -2.93091058 -0.63153172 -1.03915645
C -3.39611003 -0.22466049 0.38238449
F -2.96367416 2.11225466 -0.09587069
F -3.44067927 1.54635762 1.96087446
F -1.48603717 1.04863349 1.12263432
F -3.40801099 0.20405294 -1.99208979
F -1.56937264 -0.65021461 -1.15979719
F -3.37086259 -1.87548914 -1.32803727
H -2.17994780 -1.41603158 1.41442660
H -4.48486755 -0.07287619 0.29923557
O -3.16603025 -1.21297612 1.32715904
C 2.16167050 1.78656970 0.15934861
C 2.11556075 -0.28095663 -1.39451654
C 1.49684204 0.42783298 -0.16530169
F 2.18825729 2.61011253 -0.91085556
F 1.48249824 2.40156757 1.14675203
F 3.44834298 1.62872390 0.59065299
F 1.92544377 0.41766428 -2.53165675
F 3.45743370 -0.48756553 -1.25341932
F 1.54373142 -1.49740641 -1.55177639
H 2.42314626 -0.64603784 1.25489177
H 0.45351901 0.64933377 -0.43288099
O 1.48659830 -0.40069494 0.96223531

h2m2-8.xyz
36
-1812.03705381073
C 0.46883500 3.24959909 0.73901625
H -0.07400704 1.43712009 1.33034889
H -0.47416422 3.75134905 1.00865134
H 0.46582786 3.03087697 -0.33979132
H 1.30270753 3.92563251 0.96203333
O 0.66266576 2.06627598 1.52377660
C -4.14871835 2.50464489 1.52431549
H -4.52290286 0.55993626 1.39958363
H -5.13126610 2.78362309 1.93089495
H -4.12084960 2.70192091 0.44242824
H -3.37583487 3.10014481 2.02208140
O -3.85025539 1.12425900 1.81891426
C 2.94295390 0.57904546 -1.05826376
C 2.81100371 -1.11107786 0.88824021
C 3.39984913 0.22893985 0.38076372
F 3.40592497 -0.30594641 -1.97261392
F 3.40381798 1.80141007 -1.40154548
F 1.58176816 0.61548937 -1.18112807
F 2.97735016 -2.12802170 0.00588044
F 1.47776306 -1.01053104 1.14751089
F 3.41846088 -1.46550278 2.04142808
H 2.18306810 1.46428392 1.36000745
H 4.48836649 0.06956815 0.30913514
O 3.16935796 1.25759964 1.28145417
C -2.13929952 0.28957947 -1.40361954

C -2.16983694 -1.78458459 0.14283793
C -1.50262602 -0.42872396 -0.18938321
F -2.00681945 -0.42004144 -2.54240008
F -1.53705738 1.48776524 -1.58699608
F -3.46890703 0.53635512 -1.21993349
F -2.19353567 -2.61514649 -0.92208249
F -3.45780388 -1.62340210 0.56903246
F -1.49379852 -2.39346711 1.13600489
H -2.39603112 0.63385087 1.26099790
H -0.46567808 -0.65604475 -0.47652736
O -1.46669586 0.39808178 0.93878559

h2m3-1.xyz
42
-1927.84662095862
C 0.59078572 -0.45279759 -3.19108612
H -0.65607322 0.61004558 -2.03272671
H -0.20621250 -0.85932934 -3.82987831
H 1.45762126 -0.20864672 -3.81646697
H 0.88402957 -1.20667008 -2.44667176
O 0.16225001 0.76910565 -2.56966976
C 2.43204540 3.12763288 -1.73684050
H 1.14793367 1.56916642 -1.63980893
H 2.86400727 3.84031658 -1.02447428
H 3.24743557 2.61418334 -2.26768810
H 1.80789189 3.67133029 -2.46063906
O 1.64028294 2.18735315 -0.99116166
C -0.38888471 2.85985497 1.79790547
H 0.26361286 2.69303930 -0.08139768
H 0.28290819 2.06867465 2.16290013
H 0.04614342 3.84130280 2.04067821
H -1.36196668 2.77252533 2.29343552
O -0.61648399 2.75056553 0.38272914
C 1.71386438 -0.92550565 1.30644104
C 3.98328085 -0.64865592 0.09326398
C 3.05733930 -0.16012430 1.23395061
F 1.86424681 -2.26699776 1.35074938
F 1.04268678 -0.55216533 2.42616183
F 0.91033678 -0.63173128 0.24775905
F 4.30744166 -1.95684886 0.21563055
F 3.41906386 -0.47426263 -1.13284980
F 5.13321898 0.06296644 0.11263834
H 2.41148027 1.47007540 0.30501700
H 3.58826730 -0.38950263 2.17161692
O 2.84518178 1.21365138 1.17584876
C -3.77158404 0.39850754 0.74743468
C -2.67883516 -1.39607712 -0.74499091
C -2.47073552 -0.10483709 0.07114374
F -4.19580301 -0.45748845 1.70824352
F -3.51641261 1.59079058 1.35215189
F -4.78163406 0.59187524 -0.12269094
F -3.22529339 -2.37852614 0.00605110
F -3.45989699 -1.20618445 -1.82790378
F -1.47347583 -1.84558690 -1.18941645
H -1.49663048 1.60752935 -0.24102904
H -1.78054357 -0.36860284 0.89306058
O -1.95021876 0.86863860 -0.78833185

h2m3-2.xyz
42
-1927.84663888092
C -0.58534301 -0.55003158 3.15112359
H 0.66369070 0.54835986 2.02893497
H -0.87276652 -1.28104040 2.38209253
H 0.20840624 -0.97485379 3.78202863
H -1.45646190 -0.32648556 3.77817766
O -0.15479298 0.69098441 2.57016897
C -2.39891688 3.09361047 1.80930845
H -1.13676120 1.52141804 1.66406578
H -2.82743179 3.82868351 1.11790586
H -3.21629865 2.57941283 2.33630654
H -1.76007161 3.61149027 2.53921960
O -1.62774518 2.15996533 1.03429056
C 0.38224206 2.85585716 -1.76537738
H -0.25885494 2.67473667 0.11746674
H 1.35279951 2.77259467 -2.26666273
H -0.29069971 2.06629766 -2.13162110
H -0.05505457 3.83825829 -1.99979842
O 0.61796324 2.73836188 -0.35225137
C -1.75230085 -0.89942567 -1.32922794
C -4.00505413 -0.62737218 -0.08475366
C -3.08742264 -0.12236839 -1.22529687
F -1.91455152 -2.23972627 -1.36811941
F -1.10451458 -0.53244977 -2.46458105
F -0.92159851 -0.61130393 -0.28994221
F -4.37164753 -1.91905279 -0.25427593
F -3.41211900 -0.52116483 1.13571286
F -5.13268048 0.11841151 -0.05089547
H -2.41512509 1.48187617 -0.27091560
H -3.63155538 -0.32708336 -2.16107826
O -2.85962977 1.24748131 -1.14257578
C 3.79832624 0.42224602 -0.73175090
C 2.70305181 -1.41319299 0.71060790
C 2.49430155 -0.10840081 -0.08316777
F 4.25173772 -0.41374840 -1.69684287
F 3.53424575 1.61839045 -1.32470827
F 4.79086764 0.61914576 0.15764832
F 3.27262834 -2.37505511 -0.04954535
F 3.46334291 -1.23606657 1.81045273
F 1.49485480 -1.88338544 1.12538110
H 1.49705014 1.58826426 0.25157061
H 1.81766491 -0.36277853 -0.91940798
O 1.95323291 0.84257234 0.78842422

h2m3-4.xyz
42
-1927.84665061159
C 0.60146579 -0.55534673 3.14337759
H -0.65117541 0.54571455 2.02899270
H 0.88191974 -1.28598678 2.37153021
H -0.18911402 -0.97900342 3.77908074
H 1.47734478 -0.33469265 3.76475029
O 0.16981610 0.68731177 2.56672691
C 2.39880364 3.10339282 1.80399333
H 1.14667547 1.52307450 1.65908272
H 2.82161069 3.84180250 1.11264372
H 3.22023259 2.59381385 2.32918694
H 1.75789353 3.61640599 2.53548214
O 1.63227515 2.16556814 1.02914298
C -0.39742720 2.86489962 -1.75621164
H 0.25835625 2.67653926 0.12071484
H 0.04234433 3.84636900 -1.99001045
H -1.37229598 2.78828875 -2.25023274
H 0.26881771 2.07409351 -2.13184252

O -0.62220043 2.74197122 -0.34197373
C 1.74984168 -0.87534349 -1.35399606
C 3.98491058 -0.64355308 -0.07123083
C 3.09426712 -0.11868657 -1.22412264
F 1.89302584 -2.21763957 -1.39823273
F 1.12558444 -0.49250133 -2.49712446
F 0.90716369 -0.58111710 -0.32590545
F 4.33135274 -1.94101737 -0.23888593
F 3.37446841 -0.53056912 1.14009710
F 5.12484275 0.08186219 -0.01691715
H 2.43007418 1.48980037 -0.27230407
H 3.65074982 -0.32675963 -2.15187662
O 2.88552023 1.25393599 -1.13772442
C -3.80007951 0.42001059 -0.71410140
C -2.68430385 -1.42176195 0.70289226
C -2.48835081 -0.10760153 -0.07882032
F -4.24560124 -0.40434779 -1.69293760
F -3.55246448 1.62968328 -1.28627060
F -4.79247759 0.58852403 0.18123793
F -3.25692332 -2.37796356 -0.06226110
F -3.43560339 -1.25948069 1.81119471
F -1.47066833 -1.89129281 1.10191052
H -1.49718036 1.58912082 0.26494773
H -1.81506570 -0.34897693 -0.92138845
O -1.94809072 0.83838923 0.79869323

h2m3-6.xyz
42
-1927.84663437469
C -0.58017221 0.55216495 -3.15072174
H 0.66604230 -0.54873156 -2.02835507
H 0.21483307 0.97830694 -3.77915435
H -1.45031241 0.33053202 -3.77977443
H -0.86853872 1.28128911 -2.38027266
O -0.15095589 -0.69046456 -2.57218905
C -2.39034557 -3.09894675 -1.81333397
H -1.13366386 -1.52205660 -1.66813147
H -2.82264634 -3.83074531 -1.12081482
H -3.20480853 -2.58839889 -2.34833615
H -1.74626825 -3.62000079 -2.53632782
O -1.62511156 -2.16033603 -1.03824303
C 0.38514919 -2.86183343 1.76432084
H -0.25876382 -2.67315493 -0.11728703
H -0.28773534 -2.07422308 2.13466541
H -0.05097765 -3.84545236 1.99590754
H 1.35636592 -2.77973091 2.26462775
O 0.61856204 -2.73882844 0.35135908
C -1.75711367 0.88596203 1.33962035
C -4.00174155 0.63515167 0.07837221
C -3.09518415 0.11655645 1.22165363
F -1.91489419 2.22607928 1.39951601
F -1.11258112 0.49882896 2.46997724
F -0.92570486 0.61136846 0.29730532
F -4.35969256 1.92866197 0.25300469
F -3.40124392 0.53168598 -1.13843679
F -5.13488264 -0.10148667 0.03263856
H -2.42278026 -1.48475391 0.26181781
H -3.64439818 0.31768534 2.15520390
O -2.87457347 -1.25385004 1.13056585
C 3.80056346 -0.41901681 0.72824701
C 2.69886178 1.41673188 -0.70727407
C 2.49359776 0.11049921 0.08526056
F 4.25504723 0.41610328 1.69357285
F 3.54194216 -1.61736522 1.31942546
F 4.79018925 -0.61100876 -0.16534287
F 3.27689822 2.37590751 0.04975351
F 3.45000314 1.23966687 -1.81343824
F 1.48848778 1.89056358 -1.11128174
H 1.49688367 -1.58668332 -0.25006049
H 1.81919970 0.36251792 0.92382622
O 1.95122398 -0.83965750 -0.78639307

h2m3-7.xyz
42
-1927.84924333866
C 0.41312896 -1.47850046 2.61373602
H 1.19964999 0.08098469 1.66934598
H 1.34782215 -1.92824094 2.97940721
H -0.36867460 -1.62083168 3.36963198
H 0.10790172 -1.98108139 1.68407011
O 0.57445542 -0.06424408 2.42527224
C -2.40385516 1.49400225 2.97813068
H -0.73375900 0.78954264 2.09601676
H -3.18160319 2.22379965 2.72372468
H -2.88441446 0.53178758 3.21594760
H -1.85180799 1.85343656 3.85906412
O -1.52342595 1.38173237 1.85468816
C 0.34569067 3.12706857 -1.20185573
H -0.66254498 1.41597900 -1.19531377
H -0.30651717 3.65598995 -1.91155402
H 0.05812607 3.39081977 -0.17274138
H 1.38516160 3.42798152 -1.37405154
O 0.26799749 1.70459344 -1.41791339
C -2.50218387 -1.31410687 -0.47044717
C -4.49116583 0.33796310 -0.55425263
C -3.02226998 0.06072727 -0.95677461
F -3.13697967 -2.33730305 -1.08531043
F -1.18069581 -1.41817702 -0.75924541
F -2.63912086 -1.48150794 0.86862782
F -5.31642130 -0.67857132 -0.89776404
F -4.61994036 0.54798620 0.77860951
F -4.92492700 1.45364248 -1.18622054
H -2.04259927 1.15020615 0.48131785
H -3.01399539 0.01036293 -2.05733200
O -2.19984162 1.11158955 -0.55188650
C 4.21630714 0.97765269 -0.23553562
C 3.27162104 -1.43099484 -0.35110540
C 3.01862580 0.07056264 -0.60565242
F 5.32853548 0.66877983 -0.94438099
F 3.89435516 2.26635111 -0.52712975
F 4.52352917 0.92030208 1.07710768
F 4.36113350 -1.88431453 -1.01243668
F 3.43661324 -1.70522695 0.96503513
F 2.20197909 -2.14277936 -0.78459276
H 1.22744945 0.95635088 -0.50892627
H 2.87528586 0.17826851 -1.69445300
O 1.88894633 0.46749709 0.11266371

h2m3-8.xyz
42
-1927.84743603284
C 1.06619029 3.24142613 1.37569675
H 0.05414967 2.97841977 -0.34318468
H 0.79701303 4.30628811 1.43733009

H	2.10654895	3.12210038	1.70089597
H	0.40694217	2.66749518	2.04621053
O	0.97795792	2.78230079	0.02195827
C	-0.56356950	-1.18113571	-2.92297425
H	0.29217906	0.01624641	-1.55656141
H	-1.50482440	-1.16779445	-3.48496643
H	-0.50027771	-2.11531213	-2.34665624
H	0.27737246	-1.12840224	-3.62954053
O	-0.56479266	-0.03253409	-2.06295407
C	-1.24316011	3.50314334	-2.43765434
H	-1.95789212	2.48591186	-0.89721958
H	-0.53152972	4.32690644	-2.56861711
H	-2.20822153	3.79116887	-2.88143641
H	-0.85808872	2.60642772	-2.94782341
O	-1.38152102	3.28178846	-1.02321032
C	-2.23964736	0.42475996	1.46772184
C	-3.50925699	-1.27012206	-0.02219820
C	-3.16608459	0.21460115	0.24548057
F	-2.70140234	-0.17476890	2.58618898
F	-2.14913856	1.75712134	1.72400761
F	-0.98097588	-0.02465792	1.23874720
F	-4.19988651	-1.81580576	1.00564385
F	-2.40318464	-2.02559094	-0.23096011
F	-4.28098172	-1.36006855	-1.13040634
H	-1.81826408	0.35737363	-1.27474582
H	-4.12028897	0.70083295	0.50383378
O	-2.64685329	0.83794627	-0.88980650
C	3.86989847	0.16221190	0.35061503
C	2.21062262	-1.76739406	-0.07090375
C	2.38430929	-0.26968735	0.25360201
F	4.49042229	-0.41927852	1.40609890
F	3.92260743	1.50730960	0.54147929
F	4.57571202	-0.12039942	-0.76232596
F	2.88631637	-2.55199642	0.79790767
F	2.61775435	-2.07485944	-1.32211700
F	0.89491257	-2.09601462	0.01863463
H	1.46968385	1.40933671	-0.37007783
H	1.95713522	-0.12460547	1.26264559
O	1.72275613	0.46915494	-0.73098602

h2m4-1.xyz

48

-2043.64987240813

C	0.16707989	-0.10185421	-3.57823970
H	0.66748060	-1.46134026	-2.16914856
H	-0.49519977	-0.73121914	-4.19387415
H	-0.22003927	0.92299780	-3.57245231
H	1.17664810	-0.10104145	-4.01538110
O	0.21032971	-0.55732163	-2.21858515
C	0.80595283	-4.02465161	-2.34262554
H	1.37378625	-2.84731362	-0.84733093
H	1.38420014	-4.91947493	-2.06485241
H	-0.22385194	-4.12057596	-1.96411227
H	0.78624225	-3.94861233	-3.43658000
O	1.44096540	-2.83655823	-1.84165223
C	-2.66492565	-3.74823260	0.81253825
H	-1.93676485	-2.19858391	-0.23453248
H	-2.84363513	-3.20446434	1.75228841
H	-3.60733134	-3.81428415	0.24921952
H	-2.31844880	-4.76323713	1.04223214
O	-1.64719684	-3.11798065	0.02171213
C	1.25548676	-3.22647905	1.94524411
H	-0.18922658	-2.80234530	0.59985681
H	2.30687236	-2.95329201	2.08352228
H	0.68267790	-2.90864325	2.82887598
H	1.17708130	-4.31811194	1.83029105
O	0.78311116	-2.55365574	0.76483055
C	1.97431717	2.13062955	0.03129039
C	3.53718700	0.15222373	0.56059838
C	2.12605572	0.59985972	0.11308035
F	2.90122882	2.67881575	-0.79041686
F	0.75147220	2.43584896	-0.48288866
F	2.06921820	2.73344601	1.23327127
F	4.49383177	0.51567978	-0.32574027
F	3.88701100	0.62251179	1.77528874
F	3.55830696	-1.21194709	0.63290566
H	1.01863907	-0.85114185	0.87042443
H	1.98556379	0.22752095	-0.91786986
O	1.14145372	0.13360981	0.99312477
C	-2.74425634	1.69919126	-0.72288622
C	-2.66200162	0.16767019	1.34754548
C	-2.00034259	0.56266131	0.01564705
F	-2.69324659	2.86397913	-0.03773437
F	-2.14486452	1.90678359	-1.92607880
F	-4.04256504	1.40858156	-0.96324192
F	-2.86181137	1.23099651	2.15256147
F	-3.85210853	-0.45645223	1.17069672
F	-1.85298396	-0.70399374	2.01261353
H	-1.10353525	-0.52937316	-1.39469211
H	-0.99681001	0.94173596	0.27266668
O	-1.94127321	-0.57930732	-0.80365804

h2m4-4.xyz

48

-2043.64781365871

C	-2.11696612	3.86365223	0.99316090
H	-1.62079206	2.87756776	-0.66756627
H	-2.90892522	4.53756783	0.62935257
H	-1.55304533	4.37794726	1.78117414
H	-2.57733279	2.95789699	1.41379474
O	-1.19729984	3.54881371	-0.06333269
C	-3.07799616	1.91944269	-2.70718674
H	-1.39926761	1.10540555	-1.95071022
H	-3.47707343	1.01296239	-3.18678412
H	-2.52118014	2.51204298	-3.44786174
H	-3.91520562	2.51561106	-2.32497820
O	-2.23517319	1.57815812	-1.59448340
C	-0.07062336	-0.57306909	-3.42299135
H	0.66934871	0.49021408	-1.89666038
H	-0.32621546	-1.49041414	-2.87321020
H	0.91024248	-0.69844634	-3.90391160
H	-0.82461749	-0.39648846	-4.20007187
O	-0.06434367	0.57603573	-2.56419932
C	2.06730415	3.91387017	0.22826529
H	0.27395283	3.04171471	0.34251591
H	3.11058506	3.60447010	0.35671352
H	1.85196313	4.73970252	0.92292535
H	1.91938232	4.26456859	-0.80520002
O	1.23626204	2.78183276	0.52493806
C	3.91167116	-0.02530110	0.47173627
C	2.13634355	-1.68636535	-0.38146737
C	2.40893578	-0.28065767	0.19062737
F	4.38831545	-0.84248551	1.44138286
F	4.06668931	1.25247905	0.91021165
F	4.68516710	-0.17546640	-0.62395385
F	2.64549742	-2.65987090	0.40590929

F 2.64558544 -1.84293114 -1.62569477
F 0.79639420 -1.88890965 -0.46710836
H 1.64666953 1.53304013 -0.19526103
H 1.90208684 -0.24158395 1.17101536
O 1.91405115 0.66842858 -0.70932702
C -1.76403068 -0.26340645 1.74847409
C -3.09605706 -1.76449486 0.10514575
C -3.12626060 -0.55829351 1.07426892
F -1.20742652 -1.35029465 2.32996826
F -1.93767577 0.67109800 2.71570988
F -0.86370676 0.23918593 0.86218277
F -2.71807624 -2.91123156 0.71530835
F -2.25800283 -1.55366746 -0.94397889
F -4.33577119 -1.96054995 -0.40267264
H -3.04955587 0.84829734 -0.31996018
H -3.81177641 -0.84346021 1.88795101
O -3.63088270 0.58300379 0.45641858

h2m4-6.xyz
48
-2043.64990121074
C 0.77079376 -4.02993898 2.36056284
H 1.35537604 -2.85850998 0.86682755
H 1.34550513 -4.92913044 2.08963688
H -0.25659939 -4.12047408 1.97414761
H 0.74269211 -3.95098562 3.45412542
O 1.41709899 -2.84707153 1.86145115
C 1.25308111 -3.24519733 -1.92212773
H -0.19492202 -2.80105354 -0.58828326
H 0.69049357 -2.92671217 -2.81209369
H 1.16092896 -4.33508251 -1.80066204
H 2.30851780 -2.98529805 -2.05458478
O 0.77981742 -2.55914137 -0.74966007
C -2.67368458 -3.73734731 -0.81412031
H -1.94473580 -2.19048369 0.23660352
H -2.84359577 -3.19457990 -1.75606874
H -3.62001188 -3.79695184 -0.25668528
H -2.33148427 -4.75477909 -1.03950381
O -1.65754623 -3.11115096 -0.01819414
C 0.17039985 -0.08205019 3.57258229
H 0.65203430 -1.46417088 2.17870434
H -0.50490922 -0.68926034 4.19629906
H -0.19474887 0.95075553 3.55382325
H 1.17982758 -0.09709252 4.00972540
O 0.20374765 -0.55543280 2.21889296
C 1.97733598 2.11751286 -0.00186231
C 3.54607419 0.15757574 -0.57834174
C 2.13706207 0.58907085 -0.10837098
F 2.91637620 2.66066239 0.80929071
F 0.76252545 2.40788258 0.53972543
F 2.04483391 2.73731807 -1.19702887
F 4.50880860 0.50633197 0.30754447
F 3.88292467 0.65588878 -1.78546478
F 3.57260789 -1.20438540 -0.68020611
H 1.02108822 -0.85500894 -0.86365772
H 2.01071845 0.20042880 0.91836194
O 1.14606998 0.12953613 -0.98442976
C -2.76169414 1.70229535 0.70852296
C -2.63686371 0.17053677 -1.35932523
C -1.99961709 0.56886552 -0.01656284
F -2.70331952 2.86706798 0.02359503
F -2.18518670 1.91300389 1.92215432
F -4.06305005 1.40648992 0.92530210
F -2.82689274 1.23290063 -2.16785862
F -3.82800597 -0.45753121 -1.20238709
F -1.81420438 -0.69879825 -2.01024352
H -1.11045964 -0.52418202 1.39746545
H -0.99402317 0.95302193 -0.25706719
O -1.94746008 -0.57195618 0.80475316

h2m4-7.xyz
48
-2043.64840849567
C 2.49739620 -2.57760790 2.40652506
H 1.42373297 -2.23174965 0.73098567
H 2.78215361 -2.01668080 3.30497734
H 3.40830829 -2.91349648 1.89017424
H 1.89977125 -3.45296170 2.69908159
O 1.73109335 -1.70013529 1.56187138
C -2.60618245 -3.89524584 -0.92850561
H -2.04755127 -2.35444989 0.20986966
H -2.23100484 -4.91134179 -1.10214742
H -2.64337390 -3.35600221 -1.88829616
H -3.62333095 -3.96041353 -0.51338506
O -1.71696799 -3.26631829 0.00160415
C 1.21327182 -2.87760983 -1.73875111
H -0.12335405 -3.21881068 -0.27856329
H 2.30202903 -2.76846519 -1.80320750
H 0.74174528 -1.95359420 -2.10523282
H 0.90496352 -3.72010054 -2.37698832
O 0.88047666 -3.13940547 -0.36970159
C -0.20393070 0.45120584 3.43137973
H 0.32949846 -1.08149609 2.25278394
H -1.14559545 0.96028847 3.66421766
H 0.47327144 1.14821555 2.91594509
H 0.26339376 0.12117846 4.37202026
O -0.51843154 -0.68167959 2.60584342
C -3.40070179 1.43986646 0.67617004
C -2.62162650 0.10925867 -1.39248676
C -2.36154042 0.45395978 0.08702892
F -3.35806384 2.64518809 0.05879916
F -3.11024717 1.64711630 1.98864740
F -4.66484522 0.97541847 0.60681353
F -2.67972508 1.21335974 -2.16869255
F -3.76630566 -0.58961900 -1.56711294
F -1.60418272 -0.66500765 -1.85981001
H -1.64345229 -0.65995456 1.57104464
H -1.39137142 0.97907095 0.11429798
O -2.35204671 -0.73329659 0.82807275
C 1.72097948 1.56864885 -0.46040791
C 4.12874994 0.61836084 -0.49637989
C 3.03865503 1.35393350 0.32101625
F 1.89480201 2.21670813 -1.63173081
F 0.86809712 2.31179778 0.29438914
F 1.09046192 0.39205211 -0.72361574
F 4.43840002 1.26258038 -1.64609120
F 3.75438852 -0.64870124 -0.81791066
F 5.25846277 0.53060150 0.24198556
H 2.43588554 -0.20281274 1.39848324
H 3.43953255 2.36037183 0.52158968
O 2.80201508 0.72527868 1.53887578

h3m1-1.xyz
42

-2486.47051929220
C 0.10338837 1.33542842 3.31124409
H 0.87351880 1.90707723 1.56979310
H -0.90713670 1.33710032 3.73291457
H 0.48822151 0.30581236 3.28576590
H 0.75613394 1.96298029 3.93367918
O -0.00905654 1.89094425 1.98314954
C 2.85522722 1.96658349 -1.02832335
C 3.79103831 0.28150965 0.68609946
C 3.54116226 0.59601157 -0.81049713
F 3.48453890 2.97683304 -0.38321406
F 2.82578200 2.26317825 -2.34066003
F 1.55932784 1.94818961 -0.58793027
F 4.67615361 1.13847883 1.24539643
F 2.64036157 0.34577644 1.42615338
F 4.28000100 -0.96608693 0.81945298
H 1.94109471 -0.53921975 -1.08030750
H 4.53989072 0.67809105 -1.26790858
O 2.85370749 -0.42167648 -1.46204306
C -3.65032207 1.23760183 0.93831954
C -2.27585393 1.89820335 -1.16731145
C -2.60981900 0.80860470 -0.12125174
F -4.80804188 1.64700622 0.37470893
F -3.92962000 0.18280744 1.74011090
F -3.18712157 2.23815036 1.72675903
F -3.37190648 2.30155665 -1.84728471
F -1.70062499 2.98612944 -0.60499236
F -1.39787856 1.38648264 -2.06456668
H -0.96602209 1.04920023 1.06233428
H -3.05938204 -0.02681833 -0.67759941
O -1.44716474 0.33773874 0.50519870
C 0.06988832 -3.03754928 0.97258748
C -0.87975607 -2.98157419 -1.44153981
C 0.25270844 -2.54287190 -0.48331436
F 0.10695541 -4.38769140 1.04644712
F 1.08032348 -2.55536209 1.73591461
F -1.09332245 -2.61693370 1.52137131
F -1.09162775 -4.31488628 -1.41129429
F -2.05587462 -2.36550879 -1.14386786
F -0.55304177 -2.63875245 -2.70709986
H -0.36217291 -0.64385856 -0.18758676
H 1.17380550 -3.01886733 -0.85265116
O 0.43220975 -1.15652135 -0.54132377

h3m1-3.xyz

42
-2486.47051272116
C 0.08168894 -1.29994317 3.31039806
H 0.84164891 -1.90907417 1.57662877
H 0.49062991 -0.28009584 3.27241385
H 0.71776433 -1.93450513 3.94295295
H -0.92970567 -1.27199972 3.72908816
O -0.04048397 -1.87041621 1.98949091
C -3.67324280 -1.14178501 0.94827158
C -2.32617394 -1.90921652 -1.13772727
C -2.62442603 -0.77650285 -0.12703449
F -4.83849356 -1.55296830 0.40197755
F -3.93248970 -0.04977962 1.70563500
F -3.22837380 -2.11720931 1.77783964
F -3.43305692 -2.28451329 -1.81666583
F -1.80374675 -3.00439057 -0.53936366
F -1.41951740 -1.46174805 -2.04048775
H -0.98045453 -1.02466717 1.05526672
H -3.05229554 0.05260609 -0.70956180
O -1.44830951 -0.31740122 0.48220210
C 0.23145699 3.01240666 0.92635657
C -0.89782702 3.00987198 -1.41060514
C 0.29033882 2.53043882 -0.54357242
F 0.26462098 4.36153518 1.01166252
F 1.30767383 2.53103731 1.59450470
F -0.87662852 2.57539281 1.56892622
F -1.05363453 4.35060845 -1.37281409
F -2.06969103 2.44248743 -1.01609598
F -0.68683566 2.65015658 -2.69613749
H -0.35984623 0.64492729 -0.23308620
H 1.19372567 2.98557803 -0.97752038
O 0.42739147 1.14007930 -0.62590025
C 2.82097853 -2.03470249 -1.00924814
C 3.76871271 -0.30632346 0.65253401
C 3.51791586 -0.66369930 -0.83457904
F 3.43377455 -3.02599207 -0.31978023
F 2.80250576 -2.38027539 -2.30981454
F 1.52112165 -1.98804042 -0.58359460
F 4.65884388 -1.14396403 1.23321559
F 2.61955081 -0.35650393 1.39613097
F 4.25257473 0.94614156 0.75081834
H 1.93041519 0.48119805 -1.14018916
H 4.51651679 -0.76842261 -1.28759448
O 2.84028352 0.33837281 -1.51971133

h3m1-5.xyz

42
-2486.47051351596
C 0.07522371 -1.27009915 -3.32836157
H 0.85543942 -1.88144053 -1.60505864
H 0.47401850 -0.24641425 -3.28594946
H 0.71252773 -1.89314296 -3.97103012
H -0.93972810 -1.24916502 -3.73887916
O -0.03032213 -1.85184937 -2.01083186
C -3.66530828 -1.18418416 -0.94755164
C -2.29783584 -1.90569183 1.14142176
C -2.61880900 -0.78923142 0.11961114
F -4.82944570 -1.58510068 -0.39119408
F -3.92803153 -0.11226209 -1.73184839
F -3.21688974 -2.17834587 -1.75245729
F -3.39905580 -2.30754351 1.81422020
F -1.73951372 -2.98962358 0.55510071
F -1.41153340 -1.42638538 2.04829542
H -0.97659838 -1.02157030 -1.06905259
H -3.05844377 0.03932870 0.69404854
O -1.45183583 -0.31769777 -0.49778847
C 2.82062920 -2.01432797 1.02503951
C 3.77415668 -0.31488422 -0.66480112
C 3.51912641 -0.64731626 0.82728859
F 3.44268657 -3.02018347 0.36580948
F 2.78852139 -2.32964685 2.33324947
F 1.52590767 -1.97903303 0.58441206
F 4.65643095 -1.16938587 -1.23249860
F 2.62475020 -0.36466639 -1.40885067
F 4.26940548 0.93145501 -0.78155153
H 1.92921851 0.49941835 1.11452227
H 4.51680696 -0.74488273 1.28392195
O 2.84131890 0.36783987 1.49271315
C 0.20263158 3.03888794 -0.92558027
C -0.91214675 2.99648983 1.41771577
C 0.27414908 2.53737960 0.53753360

F 0.22911162 4.38939744 -0.99263258
F 1.27629972 2.57134054 -1.60742681
F -0.90783509 2.60596479 -1.56676011
F -1.07299973 4.33712421 1.40312614
F -2.08345777 2.43100704 1.01817326
F -0.69493777 2.61574386 2.69610617
H -0.36689068 0.65076719 0.21280738
H 1.17691253 2.99344916 0.97182032
O 0.42246340 1.14734070 0.59954098

h3m1-6.xyz
42
-2486.47053160878
C -0.06221382 1.20803789 -3.32279407
H -0.78903280 1.87931080 -1.59714300
H -0.48055628 0.19405126 -3.24856148
H -0.70160298 1.83048624 -3.96392149
H 0.94228292 1.15745044 -3.75589948
O 0.08636092 1.81552343 -2.02123393
C 3.72371974 1.13506367 -0.90443978
C 2.30962892 1.88203781 1.14414899
C 2.65087071 0.75435817 0.14160420
F 4.86551583 1.56754511 -0.32558506
F 4.02361191 0.04591395 -1.65083238
F 3.28571561 2.10038400 -1.74865296
F 3.39052775 2.26862030 1.85708525
F 1.79296406 2.97207015 0.53125543
F 1.38057082 1.42305284 2.01828515
H 1.03263960 0.98567710 -1.07967545
H 3.07298074 -0.07000424 0.73495202
O 1.49795033 0.28117651 -0.50058887
C -2.80259646 2.09488183 0.94526857
C -3.77523560 0.31107217 -0.64113858
C -3.49744778 0.71649270 0.82913896
F -3.42165500 3.05704360 0.22107249
F -2.77628990 2.49131143 2.23105214
F -1.50471657 2.03431271 0.51402404
F -4.70115790 1.11036868 -1.21972962
F -2.64834201 0.36756991 -1.41608370
F -4.23059538 -0.95553626 -0.69229973
H -1.90685479 -0.42275872 1.13430161
H -4.48778175 0.83338008 1.29695682
O -2.80351448 -0.25924820 1.53576135
C -0.16469071 -3.02465322 -0.96010099
C 0.76144685 -3.00089333 1.46123450
C -0.33975386 -2.51184573 0.49066631
F -0.30466317 -4.36834596 -1.02850377
F -1.11833878 -2.47097550 -1.74781328
F 1.03885255 -2.69827638 -1.48543242
F 0.92756556 -4.34045450 1.41834915
F 1.96208834 -2.42234535 1.18877823
F 0.42670112 -2.66121455 2.72579941
H 0.37132416 -0.64639390 0.19728688
H -1.28674207 -2.94035303 0.85231484
O -0.45208126 -1.11826995 0.54153248

h3m1-7.xyz
42
-2486.47007193000
C -0.66321657 -3.58610017 1.47215585
H -1.15325203 -2.66121015 -0.21845031
H 0.28637358 -3.85944081 1.94235212
H -1.22755813 -2.92314090 2.14326853
H -1.24327943 -4.49811334 1.27243920
O -0.33110197 -2.92418264 0.23396129
C 0.44310398 2.69068017 1.31380992
C 1.10658698 3.00180151 -1.17221603
C 0.02087610 2.54500129 -0.16779436
F 0.62747108 3.98772423 1.64869688
F -0.52607260 2.18971210 2.11050899
F 1.59050655 2.02074125 1.59111501
F 1.53460458 4.25919084 -0.92251450
F 2.19123510 2.18199859 -1.15439937
F 0.59837757 2.97443422 -2.42278701
H 0.24090079 0.53498406 -0.18431330
H -0.83226943 3.22647439 -0.30843683
O -0.41086810 1.24822104 -0.47308930
C -3.58252714 -1.05633092 -1.31300410
C -3.56617969 -0.19971796 1.12623729
C -3.81581535 0.14361590 -0.36305409
F -4.21926559 -2.18367484 -0.91809945
F -4.00521476 -0.75077322 -2.55260926
F -2.24661689 -1.36170850 -1.40892143
F -4.41237283 -1.15577127 1.58022040
F -2.29417371 -0.64492113 1.33681964
F -3.74224156 0.90013704 1.88394607
H -2.10687575 1.08674123 -0.66886526
H -4.88665694 0.38971192 -0.44153371
O -3.08491068 1.25190436 -0.77685158
C 3.19971834 -1.64009631 1.16991095
C 2.72208334 -1.48342553 -1.37340655
C 2.47652142 -0.88317750 0.03280539
F 4.52152128 -1.77762469 0.92413083
F 3.05986452 -0.95410411 2.32688710
F 2.67947306 -2.87874616 1.36672476
F 4.01517010 -1.34645715 -1.74647616
F 2.39987592 -2.79559138 -1.44314200
F 1.95799541 -0.82557393 -2.27734776
H 0.61766550 -1.65882866 0.30073718
H 2.90740874 0.12783838 0.01348089
O 1.10890752 -0.76370729 0.32051666

h3m2-1.xyz
48
-2602.27914587551
C -0.22636964 3.18050599 -2.47488550
H -1.12499532 2.11786368 -1.04426732
H -0.25060672 2.38987977 -3.24062981
H -1.07246525 3.86481332 -2.63014124
H 0.70965886 3.74250628 -2.56469928
O -0.27088180 2.63008226 -1.14604772
C -2.59362176 -2.16692463 -2.17913204
H -0.90089201 -1.30528936 -1.53315883
H -2.87243541 -2.67332191 -1.24398309
H -3.49927102 -1.80551432 -2.67841234
H -2.07438038 -2.87398462 -2.84112718
O -1.76363889 -1.02054940 -1.92880420
C -4.79036179 1.04193296 -0.18573166
C -2.81063332 0.29410657 1.29812137
C -3.30378501 1.25509425 0.18801863
F -5.59035546 1.01224384 0.90514155
F -5.20265595 2.06149205 -0.97510919
F -4.97705986 -0.10924088 -0.87537515
F -3.34926778 0.61139677 2.49686995

F -3.10659779 -1.00137084 1.03672886
F -1.46298539 0.39134046 1.41199917
H -2.32127101 0.24607312 -1.29530497
H -3.23637907 2.26795827 0.61504354
O -2.50939681 1.20628783 -0.96079686
C 3.37334993 2.69792870 -0.59402714
C 2.32458388 1.64595480 1.52956783
C 2.71462338 1.45203347 0.04472621
F 4.54326405 3.00528482 0.01080435
F 3.63566287 2.44649235 -1.89824748
F 2.57461354 3.79176262 -0.54210374
F 3.36910482 2.04523429 2.28748526
F 1.31961366 2.53080162 1.68896916
F 1.89586061 0.44945678 2.02704779
H 0.87062217 1.73249560 -0.83033994
H 3.48552421 0.66683384 0.03092435
O 1.62028506 1.01262786 -0.70546148
C 0.28351172 -3.44552140 0.27853350
C 2.70164085 -2.78413292 -0.33046659
C 1.28349155 -2.29479723 0.05435240
F 0.74793903 -4.34244575 1.17481946
F -0.87887093 -2.94007802 0.76192625
F -0.00708239 -4.10382245 -0.86688060
F 3.29176509 -3.45550877 0.68378918
F 2.71179589 -3.56798241 -1.42474295
F 3.47896085 -1.69479814 -0.60068400
H 1.14211069 -0.52940265 -0.83608046
H 1.38496738 -1.77791019 1.02366301
O 0.76705259 -1.46051204 -0.94895218

h3m2-3.xyz
48
-2602.27937390999
C 0.47318943 -2.98349872 1.81935712
H 0.95121895 -2.01428057 0.14436238
H -0.43720577 -3.44621405 2.21623278
H 0.76595267 -2.13821230 2.46032315
H 1.27919358 -3.72937081 1.80153459
O 0.17565062 -2.54270959 0.47889560
C 2.37253020 1.54136217 2.55097292
H 0.70150081 1.00241972 1.59598381
H 2.61225977 2.40748233 1.91757926
H 3.30064331 1.03542245 2.83692882
H 1.85222880 1.88157874 3.45778789
O 1.56549430 0.58280239 1.84584447
C 4.29868534 -1.74069866 0.32766251
C 4.01396589 0.32322271 -1.21819960
C 3.41340800 -1.00854449 -0.70891555
F 5.52906025 -2.02191559 -0.15172422
F 3.70744503 -2.91876934 0.65611543
F 4.44390994 -1.02901806 1.47215471
F 5.24238980 0.15237771 -1.75759113
F 4.11601793 1.24350782 -0.22695733
F 3.21030815 0.83791720 -2.17535041
H 2.07361431 -0.18513645 0.59230659
H 3.35853005 -1.67280086 -1.58545164
O 2.11848801 -0.81397901 -0.22194826
C -3.57877287 -2.66282363 0.57164995
C -2.84335978 -1.44673510 -1.59871512
C -3.01205499 -1.37063437 -0.06278976
F -4.78565520 -2.98858839 0.05562063
F -3.73686628 -2.47022893 1.90326031
F -2.75077311 -3.72151028 0.40770223
F -4.00804028 -1.71255235 -2.22805973
F -1.93450768 -2.37120265 -1.97434208
F -2.40322221 -0.23778739 -2.05166199
H -1.03497102 -1.69130382 0.45451854
H -3.76176644 -0.58676305 0.12345442
O -1.81529068 -0.99761460 0.55522127
C -0.15088053 3.31858697 -0.09097628
C -2.66717878 2.91207340 0.33266379
C -1.28168178 2.28160319 0.05061376
F -0.45501781 4.27108954 -0.99808017
F 0.97799105 2.69155563 -0.51077410
F 0.13701893 3.92673646 1.08205790
F -3.11014966 3.63841154 -0.71763471
F -2.67779377 3.69544637 1.42810912
F -3.56690831 1.90687601 0.53881021
H -1.34658390 0.49405250 0.89085365
H -1.36461085 1.77517061 -0.92569669
O -0.93548261 1.39733555 1.08593888

h3m2-4.xyz
48
-2602.27890702071
C -0.43042635 -2.67406572 -2.87067447
H 0.57771159 -2.47177810 -1.17135120
H 0.09797931 -3.57372236 -3.21650334
H -1.48726360 -2.74998488 -3.14922940
H 0.01110353 -1.78212174 -3.34073174
O -0.37469428 -2.58761360 -1.43318601
C 2.67263770 1.03957033 -2.87802194
H 1.05581458 0.85228613 -1.70916044
H 3.25892621 1.66758398 -2.19232835
H 3.34383964 0.33934512 -3.38840079
H 2.17726247 1.67395160 -3.62710576
O 1.70104536 0.25449203 -2.16871345
C 4.34513055 -1.33553754 0.03926184
C 2.12315785 -1.48175356 1.36537765
C 2.96376286 -2.01572315 0.18041530
F 5.12962563 -1.57156633 1.11461741
F 4.97954371 -1.82742015 -1.05087630
F 4.24328232 0.00768920 -0.11894912
F 2.79754771 -1.49946133 2.53478117
F 1.69078508 -0.21433879 1.14768814
F 1.02499388 -2.26399798 1.50729106
H 2.12563306 -0.99504367 -1.37033763
H 3.16945729 -3.07376174 0.40628561
O 2.23698413 -1.95391674 -1.01233408
C 0.94512787 3.31549952 0.06031185
C -1.62574230 3.29549377 0.21562531
C -0.32979459 2.45007808 0.13334067
F 1.00029605 4.20369523 1.07603621
F 2.03887410 2.51620007 0.15066426
F 1.03808541 3.99447163 -1.10366573
F -1.71312628 3.95947955 1.38927290
F -1.74331321 4.18471912 -0.78816174
F -2.70060978 2.45683977 0.14459228
H -0.94559631 0.82523455 -0.83702343
H -0.27030327 1.87733169 1.07502323
O -0.35066503 1.62597165 -1.00304097
C -3.89837821 -1.66767075 -0.79522605
C -2.64193901 -1.28048044 1.44201215
C -2.92139365 -0.77654544 0.00656163
F -5.09613469 -1.77840070 -0.17696310
F -4.11149805 -1.10871405 -2.01183833

F -3.41414142 -2.91495474 -1.00014375
F -3.77136482 -1.35003034 2.18225365
F -2.04777055 -2.49182464 1.46047965
F -1.80445496 -0.40162731 2.05738531
H -1.20982277 -1.45091644 -0.92767926
H -3.42573366 0.19410600 0.11984441
O -1.72892559 -0.57566759 -0.69552396

h3m2-7.xyz
48
-2602.27742813493
C 0.94939309 -3.55816904 1.65744974
H 1.24634454 -1.70527097 0.93405242
H 1.95133999 -3.63035588 2.10351505
H 0.93369063 -4.09313542 0.69588186
H 0.22132193 -4.01919412 2.33372055
O 0.57595961 -2.18106212 1.49972767
C 1.50285325 0.90183434 3.11988759
H 0.49264027 1.48359592 1.50604730
H 1.04145883 1.65360798 3.77542071
H 2.56283724 0.80291304 3.37860772
H 1.00341802 -0.06990767 3.25769490
O 1.44251046 1.33496006 1.74781190
C 4.43755055 -0.82692678 0.52115533
C 3.57578505 0.71762471 -1.37707015
C 3.32592500 -0.54430422 -0.51661478
F 5.65135016 -0.98208865 -0.04919108
F 4.14171686 -1.98546271 1.17358267
F 4.52938115 0.14786299 1.45538418
F 4.74652450 0.63665285 -2.05349424
F 3.59382266 1.84658769 -0.63560143
F 2.58174116 0.83616430 -2.28859944
H 1.94965468 0.31244909 0.74041947
H 3.33855433 -1.39494866 -1.21612218
O 2.07182480 -0.49155644 0.09494389
C -2.98684102 -3.15277765 0.21671192
C -1.58782307 -1.86056786 -1.54271432
C -2.40349670 -1.79124229 -0.22948163
F -3.73590147 -3.72468957 -0.75278522
F -3.77607436 -2.96757493 1.29757038
F -2.01342933 -4.03173907 0.56614116
F -2.34009928 -2.26591969 -2.58758160
F -0.51685120 -2.68420847 -1.44923111
F -1.11711563 -0.61792783 -1.83537799
H -0.80002971 -1.72971501 1.03692208
H -3.26978289 -1.14663139 -0.44296347
O -1.66655740 -1.20918327 0.80669423
C -1.00352531 3.46168224 -0.10824956
C -3.30962789 2.35218274 0.26502777
C -1.79746846 2.14518671 0.01071585
F -1.54896631 4.28633876 -1.03019322
F 0.26295876 3.18098759 -0.50115663
F -0.93319102 4.12444112 1.06531137
F -3.91954839 2.93687489 -0.79066648
F -3.56903138 3.08004311 1.36593351
F -3.88870335 1.12632592 0.43706342
H -1.44831778 0.41899002 0.92542853
H -1.71310135 1.64493460 -0.96911610
O -1.22595041 1.39349842 1.05148512

h3m2-8.xyz
48
-2602.27775455079
C 0.25083051 -2.05202828 -2.99076604
H 1.35555285 -1.71061661 -1.37154064
H 1.03190478 -2.60129738 -3.53524844
H -0.72114789 -2.51387515 -3.19450826
H 0.23552537 -1.00533172 -3.33277318
O 0.47825957 -2.14165895 -1.57214530
C 2.38913972 2.77141384 -1.79466626
H 0.82736537 1.56046715 -1.51083239
H 2.44599673 3.14831337 -0.76265464
H 3.40452414 2.65422548 -2.18822604
H 1.84655746 3.49280388 -2.42455939
O 1.75723854 1.48417163 -1.85161509
C 5.02120987 -0.42607622 -0.41067492
C 3.00808194 -0.19609215 1.20084029
C 3.58983356 -0.89296283 -0.05345981
F 5.85679902 -0.47931016 0.65162011
F 5.52536392 -1.22972393 -1.37570513
F 5.03323564 0.84282500 -0.88946261
F 3.66826070 -0.56320346 2.32207998
F 3.05604884 1.15659829 1.11151646
F 1.71071497 -0.55067098 1.34809962
H 2.47505530 0.19754898 -1.36524180
H 3.67064391 -1.96027440 0.20445943
O 2.75325178 -0.77206782 -1.16746021
C -3.18489572 -2.18125435 -0.77430006
C -1.89587550 -2.48450558 1.45514361
C -2.33288331 -1.51340522 0.33181532
F -4.37621728 -2.60859732 -0.30054776
F -3.43096164 -1.27940192 -1.75593520
F -2.55588758 -3.23903564 -1.34517669
F -2.94676702 -3.16807596 1.96792025
F -0.98061211 -3.38134960 1.02410764
F -1.33438405 -1.77528605 2.46213509
H -0.57152742 -1.47028382 -0.73612537
H -2.98481453 -0.76647827 0.80812951
O -1.22113985 -0.85519302 -0.20079546
C -2.98840164 2.63348420 -0.61095294
C -1.05154152 2.70704319 1.11424657
C -1.45847941 2.64626537 -0.37787953
F -3.59304730 3.70772112 -0.05724450
F -3.23963681 2.65295453 -1.93789808
F -3.57074393 1.51728485 -0.09982541
F -1.53195245 3.81364878 1.72431486
F -1.47579672 1.62469274 1.80585079
F 0.30120172 2.74534686 1.19874946
H -1.11644462 0.67331900 -0.64311996
H -1.09257580 3.57977227 -0.83212712
O -0.84715067 1.57220247 -1.03412641

h3m3-1.xyz
54
-2718.08089377457
C 0.82197897 1.66031075 -3.12178351
H -0.02709404 0.12298694 -2.18504800
H 0.17878164 2.42978524 -2.66970573
H 0.44206511 1.40239361 -4.12151802
H 1.83721345 2.05922857 -3.21990294
O 0.89797262 0.48334240 -2.29864184
C 0.35306168 -0.59732954 2.75863237
H 1.01431291 0.63722383 1.31646496
H 1.14295094 -0.43374324 3.50452447

H	0.58217672	-1.49577779	2.17051826
H	-0.60599382	-0.73621346	3.27038085
O	0.22276960	0.56316507	1.91632028
C	-2.67340093	-0.00468344	-2.83116624
H	-1.83988142	0.11467875	-1.00042928
H	-2.68940913	1.07702308	-3.01927646
H	-3.65919486	-0.32980769	-2.47156891
H	-2.43493466	-0.53667364	-3.75913560
O	-1.64891291	-0.34357037	-1.87237644
C	3.90964073	-0.36042313	0.98497535
C	4.20940764	1.83806402	-0.34041168
C	3.42966475	0.50763960	-0.19690350
F	5.24132267	-0.58579662	0.94439638
F	3.28617083	-1.56344976	0.94242343
F	3.61590448	0.20577339	2.18084174
F	5.51818850	1.62941739	-0.61854060
F	4.12902645	2.61083483	0.76204459
F	3.68290604	2.54387627	-1.37708385
H	1.58273745	0.66717163	-0.94077431
H	3.63540892	-0.07317393	-1.11232490
O	2.06521453	0.76854074	-0.03677730
C	-2.02507830	-3.56060275	0.40225530
C	0.34482900	-3.37985922	-0.62398686
C	-1.14056843	-3.74328670	-0.85567116
F	-1.58127218	-4.28112102	1.46077608
F	-3.28442399	-3.96952713	0.12815685
F	-2.09456714	-2.25887706	0.78782597
F	0.90076535	-4.06348711	0.40312308
F	0.49795918	-2.05038731	-0.36536920
F	1.05946967	-3.65725496	-1.73695874
H	-1.61739671	-2.06505845	-1.78282100
H	-1.16105986	-4.81943029	-1.08901061
O	-1.66182159	-3.05071721	-1.94568463
C	-3.92225873	1.93966486	0.92210904
C	-1.88850629	3.19480640	-0.07697466
C	-2.38623489	2.07770363	0.87081854
F	-4.52785260	3.11796883	1.19751066
F	-4.26069782	1.06227519	1.89442542
F	-4.42798815	1.47359099	-0.24574946
F	-2.27842110	4.42209069	0.32850342
F	-2.31723748	3.02081262	-1.35514805
F	-0.53258307	3.17920659	-0.10356956
H	-0.99498586	0.66427751	1.07445201
H	-2.06339362	2.37614725	1.88279061
O	-1.84727065	0.84105894	0.50383370

h3m3-3.xyz

54

-2718.07984081326

C	0.74520305	1.05462262	2.96416750
H	0.82537792	-0.32130394	1.49990342
H	-0.15020977	0.62624894	3.43689990
H	1.64363751	0.73470211	3.51361601
H	0.67550090	2.14787344	2.99780526
O	0.81191818	0.67223675	1.58529805
C	-2.90578703	-2.72038961	0.89490930
H	-2.10986119	-0.91375980	0.56398084
H	-2.90007988	-3.04356428	-0.15663753
H	-2.65622263	-3.57422494	1.53458700
H	-3.90612949	-2.35092406	1.16003217
O	-1.92188770	-1.70410737	1.13830820
C	-0.39273812	1.40228620	-3.13392444
H	0.22907396	1.97656430	-1.31568084
H	0.28637856	0.53942239	-3.18410608
H	-1.35957703	1.12347950	-3.56928124
H	0.02998548	2.23756368	-3.71231347
O	-0.63647025	1.80419142	-1.77689461
C	3.64605051	2.99228804	0.69373965
C	3.60124068	1.08258864	-1.05675137
C	2.93033073	2.37610689	-0.53341193
F	4.91556380	3.35918351	0.40182180
F	2.97871362	4.10006262	1.09158233
F	3.68867944	2.13945031	1.74689988
F	4.91579264	1.24966486	-1.32072898
F	3.47861402	0.05192153	-0.18226553
F	2.99592730	0.70876202	-2.21248817
H	1.38773784	1.54049776	0.51443223
H	3.03366347	3.11714250	-1.34210369
O	1.57402134	2.17738972	-0.28221341
C	-4.65397031	0.84762290	-1.03133360
C	-3.59782638	1.74278370	1.15934430
C	-3.38927313	1.38987090	-0.33063383
F	-5.70360656	1.69472499	-0.92200730
F	-4.39201414	0.68789595	-2.35357785
F	-5.03523201	-0.35573074	-0.54404043
F	-4.52139269	2.71975627	1.32062863
F	-3.98504996	0.67500540	1.89545694
F	-2.42889101	2.19500055	1.67517342
H	-1.58779224	0.90349033	-0.96134647
H	-3.13343851	2.33595091	-0.83600432
O	-2.36008652	0.44876184	-0.46329052
C	1.20808945	-4.23553686	0.75780899
C	0.81496586	-2.42789847	-1.04186681
C	1.27392486	-2.72984758	0.40659534
F	1.80296426	-5.00329512	-0.18640202
F	1.84780685	-4.45109454	1.93029436
F	-0.06842853	-4.66514235	0.90333355
F	1.72518754	-2.84868372	-1.94793422
F	-0.37563431	-2.99461369	-1.35316826
F	0.67265902	-1.08536322	-1.19485552
H	-0.44699481	-2.00624014	1.22533625
H	2.33825924	-2.45057132	0.44572532
O	0.58183852	-1.98099138	1.36016732

h3m3-4.xyz

54

-2718.07983957805

C	0.77079598	-1.06402459	2.97602249
H	0.84318258	0.29659101	1.49794675
H	1.67218012	-0.73119615	3.51271848
H	0.71024911	-2.15744828	3.02198177
H	-0.12312149	-0.63738418	3.45315827
O	0.81994788	-0.69607788	1.59211764
C	-2.88885417	2.69293235	0.95878148
H	-2.08901942	0.88962099	0.62230960
H	-2.90665511	3.00484460	-0.09585039
H	-2.62864044	3.55425976	1.58410142
H	-3.88210607	2.32299104	1.24909263
O	-1.89651963	1.68232097	1.19171048
C	-0.42817888	-1.49046389	-3.07508406
H	0.23276136	-2.03210442	-1.26166542
H	0.23939799	-0.62079357	-3.15350963
H	-1.40593824	-1.23156771	-3.49768422
H	-0.00546284	-2.33162342	-3.64485948
O	-0.64286936	-1.86957035	-1.70630826
C	3.71480499	-2.95727186	0.69797216

C 3.58349758 -1.06987775 -1.07180439
C 2.96124763 -2.37740580 -0.52436118
F 4.99212699 -3.28324378 0.39163554
F 3.09151836 -4.08338793 1.11497410
F 3.74246177 -2.09344069 1.74256355
F 4.89759104 -1.19899031 -1.35696617
F 3.44426713 -0.03499356 -0.20386770
F 2.94714685 -0.72473872 -2.21950800
H 1.41179840 -1.57106384 0.53472246
H 3.07436961 -3.12404253 -1.32648686
O 1.60373620 -2.21744987 -0.25279976
C -4.61271221 -0.85262967 -1.08204799
C -3.70008563 -1.68006914 1.19840606
C -3.40170550 -1.39267402 -0.28966907
F -5.67915265 -1.68319967 -1.01921660
F -4.26427846 -0.73214290 -2.38924305
F -5.00668517 0.36796589 -0.65190764
F -4.66936704 -2.61342137 1.34745862
F -4.08172001 -0.56994844 1.87268944
F -2.57848176 -2.15533152 1.79195377
H -1.57933870 -0.94851308 -0.89148522
H -3.13960626 -2.36299317 -0.74318434
O -2.34443936 -0.47992413 -0.39596274
C 1.24756178 4.20444560 0.73307544
C 0.77007537 2.40755091 -1.05703260
C 1.28192903 2.69925156 0.37563103
F 1.81990040 4.96726088 -0.22900352
F 1.93043990 4.40789724 1.88327773
F -0.01725150 4.65010027 0.92380741
F 1.65429553 2.81941152 -1.99257009
F -0.42258635 2.99090862 -1.32625095
F 0.60544518 1.06745063 -1.20889363
H -0.41914211 1.98674944 1.24313206
H 2.34311358 2.40606335 0.37810488
O 0.61232797 1.95717884 1.35072620

h3m3-6.xyz
54
-2718.07984446571
C -0.78129063 1.05415177 2.98359868
H -0.83660029 -0.30536103 1.50287973
H -1.67964721 0.71301632 3.52022787
H -0.73014969 2.14801000 3.03086146
H 0.11630228 0.63464339 3.46013241
O -0.82793569 0.68747423 1.59938820
C 2.90014230 -2.70684185 0.90263496
H 2.10439957 -0.89505887 0.60429358
H 2.64438167 -3.57646004 1.51819356
H 3.89829678 -2.34489180 1.18607782
H 2.90364607 -3.00292347 -0.15672468
O 1.91466702 -1.69610153 1.16297702
C 0.42610219 1.49243804 -3.06623278
H -0.23491385 2.03172129 -1.25229573
H 1.40448439 1.23880882 -3.49061532
H -0.00214946 2.33122765 -3.63534434
H -0.23692588 0.61914755 -3.14341182
O 0.64102656 1.87284716 -1.69786554
C -3.71529472 2.95646794 0.70492983
C -3.59413861 1.07734876 -1.07385537
C -2.96389212 2.37830914 -0.51930417
F -4.99195947 3.28631366 0.40054253
F -3.08901363 4.07975675 1.12531723
F -3.74428275 2.08948918 1.74690368
F -4.90558378 1.21903327 -1.36579789
F -3.46904530 0.03898137 -0.20836124
F -2.95473789 0.73034648 -2.21931922
H -1.41874352 1.56201286 0.54079966
H -3.07165528 3.12941856 -1.31797363
O -1.60772211 2.20915227 -0.24681731
C 4.61525857 0.86308770 -1.08578781
C 3.71377744 1.67779193 1.20211692
C 3.40716934 1.39892846 -0.28613384
F 5.68288789 1.69189229 -1.01968326
F 4.26369265 0.75149972 -2.39286454
F 5.00836085 -0.36097506 -0.66482187
F 4.67535302 2.61955968 1.34946238
F 4.11196091 0.56677817 1.86552810
F 2.59256807 2.13762910 1.80803030
H 1.58281903 0.95641357 -0.88624259
H 3.14368389 2.37204575 -0.73277867
O 2.34952818 0.48705952 -0.39340380
C -1.23315366 -4.20986246 0.73911323
C -0.79358012 -2.40786340 -1.05581500
C -1.27787803 -2.70407810 0.38553773
F -1.82737787 -4.97140128 -0.21052943
F -1.88776256 -4.41623032 1.90502300
F 0.03646937 -4.65451309 0.89869545
F -1.69387273 -2.82117538 -1.97537297
F 0.39598710 -2.98615801 -1.34860551
F -0.63626391 -1.06694256 -1.20769142
H 0.43722572 -1.99339293 1.22888729
H -2.33974240 -2.41386494 0.40805051
O -0.59300804 -1.96194389 1.34973861

h3m3-8.xyz
54
-2718.07983480623
C 0.66199409 1.06233697 -2.90562001
H 0.87198107 -0.32716751 -1.46908227
H 1.53173367 0.77302390 -3.51488433
H 0.56151724 2.15355547 -2.91938885
H -0.25046736 0.61508815 -3.32527964
O 0.82691521 0.66564365 -1.53864137
C -2.92391638 -2.55727806 -1.13421081
H -2.05692726 -0.80679372 -0.70256414
H -2.98579791 -2.91330653 -0.09528004
H -2.68561958 -3.40188075 -1.79028179
H -3.89040175 -2.12870336 -1.43369024
O -1.87942914 -1.58550209 -1.29555158
C -0.37022860 1.39600217 3.11658584
H 0.29712466 1.99481963 1.32368655
H 0.04425452 2.21663371 3.72133851
H 0.30217802 0.52786905 3.16433090
H -1.34825738 1.11430292 3.52387699
O -0.58016127 1.82730687 1.76286816
C 3.73233120 2.92738851 -0.70779742
C 3.66272103 1.01996348 1.04561330
C 3.02072859 2.33183368 0.53185897
F 5.02173939 3.24345917 -0.44484549
F 3.09882019 4.06273962 -1.08354047
F 3.71647255 2.08002975 -1.76563030
F 4.98264371 1.15450764 1.30222555
F 3.50993546 -0.00363887 0.16768948
F 3.05583998 0.65545515 2.20343591
H 1.44416132 1.53456248 -0.49052347
H 3.15623471 3.06995489 1.33831316

O 1.65594716 2.17059230 0.29944045
C -4.55502557 0.86464227 1.08844702
C -3.63839976 1.84799384 -1.12722739
C -3.34057005 1.45108244 0.33531872
F -5.60324810 1.71999637 1.11293631
F -4.19801754 0.61770961 2.37471989
F -4.97978882 -0.30333233 0.55301071
F -4.62177632 2.77451485 -1.20988494
F -3.99890930 0.78672555 -1.88668865
F -2.52169260 2.38459828 -1.67731299
H -1.52087177 0.95471965 0.89796213
H -3.07144066 2.38399896 0.85799320
O -2.28880671 0.52705731 0.37110714
C 1.07761778 -4.28465967 -0.73777907
C 0.71512376 -2.44817076 1.04002581
C 1.21942988 -2.78436744 -0.38514662
F 1.58382562 -5.08502895 0.23034272
F 1.75151783 -4.54228236 -1.88218925
F -0.21594198 -4.63565956 -0.93676857
F 1.53871184 -2.94460335 1.98969744
F -0.53335732 -2.91287029 1.28585082
F 0.67860145 -1.09854595 1.19293783
H -0.41782067 -1.97623104 -1.29061163
H 2.29936423 -2.56964057 -0.37778680
O 0.61598948 -1.99851366 -1.36875828

h4m1-1.xyz
54
-3276.69981558441
C 2.83928384 2.08499132 -2.52397725
H 2.27360473 2.85891956 -0.77537668
H 3.30811534 3.00421791 -2.90161213
H 1.83333130 1.97378373 -2.95176993
H 3.45333140 1.22432686 -2.80897324
O 2.80123047 2.09891415 -1.08157289
C -2.95085885 -1.37234692 2.34897566
C -4.23531447 -0.59432565 0.23313454
C -3.28664001 -0.20354607 1.39230462
F -4.06060540 -1.85152884 2.95519983
F -2.10606768 -0.94014093 3.30893754
F -2.34887610 -2.40363365 1.70347863
F -5.38906984 -1.13709316 0.67774559
F -3.65685214 -1.48417477 -0.61841447
F -4.54110208 0.50919947 -0.48300628
H -1.51989171 -0.22551889 0.42046549
H -3.83165599 0.53797935 1.99598708
O -2.12592130 0.41154651 0.90678213
C -1.08673897 3.17021279 -1.61060797
C -0.07404010 3.69230856 0.70833445
C -1.33803311 3.71941359 -0.18458761
F -0.19496149 3.93339529 -2.29942034
F -2.23707913 3.15779041 -2.30814303
F -0.60030515 1.89967438 -1.58789348
F 1.01768748 4.25484424 0.08768146
F 0.27489071 2.43254992 1.05369666
F -0.28940470 4.39096793 1.83658054
H -2.19916751 2.10225374 0.54990321
H -1.60770641 4.77978066 -0.30933428
O -2.39659049 3.07368458 0.44462974
C 2.82174934 0.01028662 2.17931522
C 4.41828306 -0.88871240 0.34224927
C 2.95948496 -0.75849781 0.84369599
F 3.62331334 -0.49571425 3.14284849
F 1.54132622 -0.08554017 2.61106314
F 3.10933695 1.32597057 2.03895192
F 5.15295850 -1.65827073 1.17787413
F 5.03596118 0.30930857 0.22084048
F 4.42519165 -1.47739676 -0.87615704
H 2.41243284 0.73438646 -0.43488999
H 2.61533257 -1.78468558 1.04047843
O 2.12587142 -0.20170059 -0.13594376
C 0.44326115 -3.36438137 -0.83537412
C -0.38921074 -1.72638818 -2.65975287
C -0.49580711 -2.18387904 -1.18486548
F 0.04502574 -4.50216902 -1.44405608
F 0.42391900 -3.57898195 0.50202047
F 1.73223052 -3.12842074 -1.18650785
F -0.47532432 -2.76497641 -3.52135043
F 0.77353795 -1.07411925 -2.90160621
F -1.40116523 -0.87169004 -2.93585559
H 0.62888293 -0.74497696 -0.31529180
H -1.51859566 -2.56138021 -1.04765964
O -0.31910818 -1.09966822 -0.31587421

h4m1-2.xyz
54
-3276.70817185473
C -0.04567655 -3.19438778 -2.82027765
H 0.63340412 -1.89552470 -1.45929157
H 0.87973804 -3.20703323 -3.41395638
H -0.07649428 -4.07375682 -2.15912219
H -0.90502880 -3.22902456 -3.49859633
O -0.13983037 -1.97007704 -2.07582588
C -3.59543039 -3.04961034 -0.51226555
C -1.72143894 -2.53886422 1.20552842
C -2.74633354 -1.96884397 0.19513978
F -4.18576386 -3.88738051 0.36982545
F -4.56857599 -2.45264591 -1.23529646
F -2.85246069 -3.79813045 -1.36681174
F -2.33149420 -3.14204583 2.24887987
F -0.87566409 -3.43739278 0.63961588
F -0.97116682 -1.52355837 1.69532988
H -1.38755047 -1.59776033 -1.29114665
H -3.44931748 -1.36501190 0.78725689
O -2.14147661 -1.13325502 -0.75214117
C -3.17486238 2.48186247 -1.04654531
C -2.75866106 2.08415801 1.48546492
C -2.13620406 2.28915364 0.08371546
F -3.97413404 3.54677943 -0.80746748
F -2.52976078 2.69990629 -2.21355217
F -3.96200557 1.39203674 -1.20712535
F -3.52825334 3.12814360 1.85727350
F -3.52426230 0.95941515 1.54464782
F -1.77079020 1.94671389 2.39713670
H -1.70235871 0.35645064 -0.38099187
H -1.56070807 3.22440110 0.13642108
O -1.24622977 1.25168673 -0.22542786
C 4.01936375 -1.69403151 -0.99090150
C 3.45050366 -1.88208134 1.53437280
C 2.98961204 -2.13125403 0.07902854
F 5.19613080 -2.34152377 -0.86310006
F 3.52287323 -1.97514566 -2.22017814
F 4.26753773 -0.35930226 -0.94837625
F 4.62255315 -2.50055856 1.80082950
F 3.60479559 -0.55898884 1.79365041
F 2.52149492 -2.36258683 2.38730859

H 1.71314165 -0.56655037 0.05667044
H 2.88407595 -3.22174061 -0.02635297
O 1.73935146 -1.55069707 -0.16039078
C 1.93363860 2.46884457 -1.50400756
C 2.00363834 3.23169767 0.97091834
C 2.14285081 2.05728907 -0.02545790
F 2.94385349 3.25414226 -1.93940448
F 1.90337864 1.36084548 -2.27840096
F 0.77007568 3.13710098 -1.69670358
F 2.76241091 4.29050806 0.61285732
F 0.71555654 3.65617217 1.07176201
F 2.39482122 2.83068129 2.19852675
H 0.32943553 1.19721873 0.17657603
H 3.18162708 1.70662534 0.05215518
O 1.30367743 0.99919498 0.35673761

h4m1-6.xyz
54
-3276.70919257782
C -0.17582911 1.00174674 3.27098944
H 0.53791444 1.85070087 1.61226499
H -0.09286872 -0.03737430 2.91817232
H 0.69399418 1.24632658 3.89607909
H -1.08897835 1.10838101 3.86704124
O -0.27767166 1.92911682 2.17244230
C -3.54491506 3.16711410 0.46099270
C -1.62931755 2.65170116 -1.21262348
C -2.71721544 2.08447374 -0.26978819
F -4.07948213 4.05993107 -0.40430105
F -4.56386911 2.57343655 1.12552248
F -2.80476736 3.84703966 1.36574029
F -2.16553339 3.35228666 -2.23511530
F -0.75063629 3.45415181 -0.56576716
F -0.92639045 1.61930839 -1.74338010
H -1.44012436 1.60201314 1.26580230
H -3.42199669 1.53621573 -0.91185375
O -2.17607378 1.18451726 0.65836106
C 1.99645479 -2.63732823 1.14249022
C 1.91441181 -3.05309433 -1.41281842
C 2.13332357 -2.02906993 -0.27449464
F 2.98335763 -3.52066292 1.40037279
F 2.07240152 -1.64771930 2.06466618
F 0.80893836 -3.26852350 1.32197849
F 2.69131519 -4.14776177 -1.26223522
F 0.61956740 -3.46410717 -1.47557637
F 2.21685693 -2.48216773 -2.59650642
H 0.32329751 -1.13186920 -0.28402932
H 3.17312945 -1.68313383 -0.36234024
O 1.29586234 -0.91865495 -0.45437386
C 4.05395960 1.55695531 1.17494016
C 3.38702696 2.15251250 -1.26065139
C 2.98277490 2.15738788 0.23249563
F 5.21521843 2.24159616 1.13033600
F 3.59605746 1.59847203 2.44863072
F 4.32181662 0.25653887 0.88143502
F 4.56528424 2.78211563 -1.46427899
F 3.49658571 0.88866849 -1.74441755
F 2.44253033 2.78844179 -1.98451028
H 1.71885295 0.59268688 0.05666203
H 2.87596044 3.21363145 0.52203587
O 1.74491497 1.53150838 0.41801512
C -3.01222079 -2.45275844 1.21288618
C -2.94671002 -2.05351615 -1.35154384
C -2.13784999 -2.23594800 -0.04580590
F -3.75650225 -3.57707355 1.10881193
F -2.20892827 -2.58689912 2.29450191
F -3.84518796 -1.41335338 1.44677881
F -3.77598279 -3.09160290 -1.58579813
F -3.69485228 -0.91700514 -1.32828167
F -2.09448519 -1.95319430 -2.39560595
H -1.71495571 -0.27212511 0.27240068
H -1.54735770 -3.15461150 -0.17183282
O -1.24764561 -1.16924283 0.13891827

h4m1-7.xyz
54
-3276.70853284206
C -0.55018969 1.47835198 3.36285775
H 0.23072983 2.10957561 1.63880632
H 0.10602408 2.10507392 3.98293427
H -0.15220884 0.45304443 3.33085103
H -1.55300344 1.46445914 3.80242020
O -0.67388263 2.04136329 2.04303857
C -3.60653046 2.19379569 -0.46616180
C -4.13908453 0.70217343 1.58466233
C -3.36989441 0.84134541 0.24857864
F -4.92665700 2.44841514 -0.62951178
F -3.03817373 2.15478091 -1.69319288
F -3.05830095 3.22898110 0.20839821
F -5.47580066 0.67797599 1.38973839
F -3.85928301 1.70899304 2.45061653
F -3.78728907 -0.45834218 2.18840419
H -1.53948132 1.24940545 1.09470558
H -3.77327733 0.06563084 -0.41807648
O -1.99996952 0.61015978 0.41633199
C 1.57696330 2.91670040 -1.33876266
C 3.85288900 2.76855331 -0.09449527
C 2.31996201 2.93516199 0.01928172
F 1.95590542 3.94696536 -2.12723173
F 0.24737032 3.03488388 -1.11111438
F 1.77911931 1.76468249 -2.01636816
F 4.40477200 3.65870658 -0.94394561
F 4.19176402 1.52056405 -0.51291357
F 4.41335453 2.95086751 1.12459271
H 1.89181782 1.04393704 0.55112531
H 2.14643836 3.93158130 0.45202408
O 1.79700403 1.98183236 0.90743150
C 3.26535022 -2.50044128 -0.27195549
C 2.55456738 -1.86456085 2.14354356
C 2.90775751 -1.37011241 0.72085398
F 4.28937672 -3.25721151 0.17483686
F 3.62420389 -1.96306061 -1.45555142
F 2.20625300 -3.32563309 -0.50131781
F 3.57727314 -2.53856870 2.71097106
F 1.46171452 -2.66007967 2.15454491
F 2.28511972 -0.78736583 2.92597828
H 0.99903655 -1.01812753 0.11728193
H 3.81449051 -0.75557124 0.82288911
O 1.89583069 -0.55393223 0.19427677
C -1.10199277 -1.77187932 -2.36584333
C -2.17941149 -3.21670752 -0.49783521
C -0.93704082 -2.43017669 -0.97543115
F -1.29440637 -2.69390986 -3.33404752
F 0.02226853 -1.08243471 -2.66456674
F -2.14057234 -0.90032602 -2.40191616
F -2.55415130 -4.15568312 -1.39337724

```
F -3.24345047 -2.40033494 -0.27817088
F -1.89682150 -3.83809764 0.66683111
H -1.21701682 -0.73660902 0.10865655
H -0.12824661 -3.16709445 -1.07938116
O -0.54696577 -1.49546050 -0.00363416
```

h4m1-8.xyz

```
54
-3276.70919334016
C 0.18276927 1.04204770 -3.25843035
H -0.54111160 1.86165440 -1.58912862
H 0.10493691 -0.00208357 -2.91970876
H -0.68615346 1.28983420 -3.88347598
H 1.09750788 1.16249249 -3.84936212
O 0.27455110 1.95527935 -2.14708718
C 3.51647753 3.21074395 -0.40487341
C 1.60661155 2.63737022 1.25590529
C 2.70415553 2.10286290 0.30495306
F 4.04215758 4.09140783 0.47810093
F 4.54097825 2.64424030 -1.08426147
F 2.76566450 3.90061350 -1.29324144
F 2.13053027 3.32975505 2.29022277
F 0.71575183 3.43569754 0.62116985
F 0.91986046 1.58522718 1.76955756
H 1.43836991 1.62865427 -1.24299252
H 3.41622009 1.55412563 0.93860587
O 2.17763101 1.21102860 -0.63936416
C -1.95979420 -2.66568977 -1.07766641
C -1.87886945 -3.03648248 1.48213471
C -2.10752269 -2.03249467 0.32764506
F -2.96170015 -3.53206536 -1.33540482
F -1.99897642 -1.69178863 -2.01791293
F -0.78376406 -3.32682499 -1.22717857
F -2.61768986 -4.15743237 1.32925053
F -0.57354205 -3.40443129 1.57341306
F -2.22242060 -2.46289092 2.65352634
H -0.30975056 -1.11499141 0.32065600
H -3.15147480 -1.69725214 0.40649280
O -1.28336888 -0.90990996 0.49384211
C -4.03341995 1.51932303 -1.23975063
C -3.46227627 2.11968538 1.21855973
C -3.00539269 2.13339849 -0.25932141
F -5.21212032 2.17471614 -1.22070382
F -3.53978248 1.58905011 -2.49934801
F -4.27628963 0.20928553 -0.97084001
F -4.64267463 2.75654738 1.38363520
F -3.59866782 0.85328955 1.68986628
F -2.54015318 2.74337733 1.98039720
H -1.72109268 0.58942799 -0.04096367
H -2.90473137 3.19206770 -0.54229711
O -1.75225406 1.52743191 -0.40440327
C 2.97252287 -2.41945428 -1.28771885
C 3.01698654 -2.05351794 1.28114969
C 2.15193307 -2.21714455 0.00933694
F 3.70056896 -3.55796942 -1.23683495
F 2.12596966 -2.51634288 -2.34008214
F 3.81376404 -1.38923622 -1.53219739
F 3.86414171 -3.08875857 1.45756529
F 3.75447263 -0.91052117 1.24707982
F 2.21179403 -1.98087978 2.36411480
H 1.72052936 -0.24770857 -0.26067421
H 1.56531379 -3.13634681 0.14840642
O 1.25747226 -1.14643746 -0.12200687
```

h4m2-1.xyz

```
60
-3392.50512649894
C 0.74996916 -1.09400357 3.63222546
H 0.29099657 0.65615538 2.79207694
H 1.66705722 -1.68111812 3.75332494
H 0.30585722 -0.91866359 4.62319470
H 0.04002636 -1.65513398 3.00519129
O 1.12199831 0.15646392 3.02300616
C -1.98149677 2.09868806 3.04330241
H -1.63284005 0.53612394 1.80545221
H -1.36840234 2.92876246 3.40987037
H -2.85619196 2.49803795 2.51484032
H -2.30850507 1.48072318 3.89085797
O -1.14972370 1.33391250 2.14493481
C -2.46920040 2.16577657 -1.35873824
C -1.21019217 4.31540715 -0.67416871
C -1.09487281 2.84673281 -1.15262372
F -3.22211011 2.78225121 -2.29372659
F -2.28741125 0.88153720 -1.76719957
F -3.19563544 2.11123953 -0.20782542
F -2.01537448 5.06237412 -1.46507800
F -1.69375581 4.38793480 0.59610197
F 0.01391775 4.88503654 -0.67496312
H -0.66489427 2.02391848 0.59812582
H -0.62226647 2.88011506 -2.14639520
O -0.26732145 2.11080579 -0.31019308
C -2.98133408 -2.97450965 0.04109175
C -4.04625757 -1.26842749 1.65153780
C -3.05058676 -1.49709002 0.49613078
F -4.18177169 -3.42724368 -0.37860446
F -2.12534644 -3.05719148 -1.02053191
F -2.52066832 -3.79888595 0.99782723
F -5.30270697 -1.60284177 1.29713449
F -3.71760458 -1.95538290 2.76527647
F -4.05105451 0.05396551 1.97831859
H -1.11122608 -1.07100398 0.20019740
H -3.43218721 -0.92181603 -0.36563110
O -1.79654470 -1.04425454 0.93864321
C 3.71904843 1.63479969 0.58134993
C 4.49748988 -0.64716637 1.54100105
C 3.55829163 0.09483123 0.55993174
F 5.00803062 2.01244912 0.40534507
F 2.99179110 2.17261704 -0.42315884
F 3.27856315 2.17270891 1.74255439
F 5.79946583 -0.45858911 1.22706426
F 4.31279020 -0.25646377 2.82557622
F 4.24908070 -1.97740911 1.48183639
H 1.87059812 -0.04053823 1.72467417
H 3.84914763 -0.23399689 -0.44833493
O 2.22625095 -0.26982897 0.77708276
C 1.19430077 -0.77898639 -2.97683803
C 1.45675860 -2.94440367 -1.58696923
C 0.57588443 -1.70819171 -1.90476699
F 1.58674098 -1.46470274 -4.07409247
F 0.27560675 0.13549244 -3.36747030
F 2.26641660 -0.09960651 -2.50126767
F 1.47270398 -3.80985664 -2.62676183
F 2.74091182 -2.60949280 -1.30448876
F 0.95762431 -3.59381511 -0.51329593
H 1.07396725 -0.64893486 -0.26825140
H -0.35380532 -2.10191375 -2.33766328
O 0.24846703 -0.97849903 -0.75744820
```

h4m2-2.xyz

60

-3392.50546982881

C	0.62764868	-0.92028956	3.66964091
H	0.25004306	0.80447381	2.75102909
H	1.49398630	-1.58586804	3.75172536
H	0.29819288	-0.63644362	4.67996300
H	-0.18654466	-1.45197819	3.15304641
O	1.05071132	0.24316764	2.93548579
C	-1.97951772	2.46407294	2.97013738
H	-1.73349924	0.84424758	1.78233496
H	-1.29524255	3.20316861	3.40018039
H	-2.72870971	2.97705189	2.35341246
H	-2.47827394	1.91161914	3.77872704
O	-1.18083781	1.57561310	2.16361938
C	-1.53898808	2.69192404	-1.60574878
C	0.25481901	4.28241447	-0.64843775
C	-0.08300096	2.84078671	-1.10265008
F	-1.76989878	3.40463324	-2.72989404
F	-1.79269172	1.38833004	-1.89196094
F	-2.44547887	3.07901317	-0.67023384
F	-0.09376422	5.21479432	-1.56566304
F	-0.37359630	4.59181147	0.51826816
F	1.58341896	4.39078401	-0.43328361
H	-0.35688454	2.06910346	0.70313225
H	0.56565800	2.62186975	-1.96416755
O	0.21499444	1.92390629	-0.09794214
C	0.85887681	-1.82745192	-2.81585075
C	0.83522701	-3.44119253	-0.79839188
C	0.13604808	-2.25660142	-1.51738633
F	1.15018928	-2.88609844	-3.60481232
F	0.06210348	-0.99009030	-3.51940688
F	2.01365765	-1.16598050	-2.55522387
F	0.60849342	-4.59876960	-1.46220709
F	2.17733577	-3.27986778	-0.69714639
F	0.34109200	-3.57481427	0.45158739
H	0.80717460	-0.84707696	-0.25270091
H	-0.84962718	-2.63301943	-1.82298563
O	-0.06040196	-1.14349886	-0.68932833
C	-3.69397296	-2.42022068	0.30865667
C	-4.43139296	-0.07098282	1.06860956
C	-3.37988069	-0.90351929	0.30653904
F	-4.83140387	-2.69497750	-0.36624168
F	-2.67957271	-3.07568994	-0.32586138
F	-3.79507236	-2.93386264	1.54831514
F	-5.68173269	-0.31565474	0.62921268
F	-4.39455065	-0.30052543	2.40100584
F	-4.17672995	1.24956285	0.87946787
H	-1.38639791	-0.87095237	0.25764483
H	-3.42185931	-0.57203473	-0.74532924
O	-2.13219103	-0.65404110	0.90190363
C	3.72797985	1.07312712	0.13150292
C	4.27100198	-0.99287537	1.59685967
C	3.39991960	-0.40459113	0.45999974
F	5.05584473	1.26770872	-0.05496094
F	3.10232880	1.42173799	-1.02032565
F	3.31205961	1.91235450	1.10223072
F	5.57143754	-1.07491636	1.23418892
F	4.19595302	-0.26383589	2.73596612
F	3.84678758	-2.24636707	1.88848935
H	1.72896028	-0.15031070	1.62759304
H	3.64419814	-0.98543095	-0.44151314
O	2.03966022	-0.56890009	0.73338972

h4m2-4.xyz

60

-3392.50546215009

C	-0.60540666	-0.95965711	-3.66331270
H	-0.24704618	0.77819391	-2.76238922
H	-1.46792342	-1.62966831	-3.74915095
H	-0.26387470	-0.68571186	-4.67236178
H	0.20431427	-1.48182091	-3.13024597
O	-1.04279093	0.21030194	-2.94786527
C	1.97852821	2.44991030	-2.97355929
H	1.73180805	0.83146163	-1.78465672
H	1.29375004	3.18370663	-3.41181560
H	2.72012222	2.96894168	-2.35274340
H	2.48663667	1.89722036	-3.77620463
O	1.17816485	1.55993159	-2.17015145
C	1.56251214	2.69421260	1.58137230
C	-0.24179288	4.28378779	0.64130320
C	0.10089016	2.84289783	1.09451658
F	1.80724470	3.41063660	2.70031295
F	1.81860478	1.39135012	1.86906663
F	2.45870572	3.07694364	0.63419074
F	0.11247746	5.21761146	1.55490292
F	0.37719784	4.59273769	-0.53046074
F	-1.57219011	4.39003219	0.43636253
H	0.35767413	2.06290728	-0.71059779
H	-0.53782490	2.62613648	1.96397010
O	-0.20952564	1.92450873	0.09504201
C	-3.73737666	1.07482466	-0.18383891
C	-4.26820873	-1.03209081	-1.59489271
C	-3.40189865	-0.40951500	-0.47276129
F	-5.06745788	1.27030477	-0.01492947
F	-3.12306598	1.45380890	0.96394686
F	-3.31475502	1.89073258	-1.17177674
F	-5.56811840	-1.11388262	-1.23043463
F	-4.19795561	-0.33112512	-2.75195533
F	-3.83524919	-2.28953453	-1.85491797
H	-1.72827040	-0.16940048	-1.63943702
H	-3.64493718	-0.96743566	0.44342789
O	-2.04034203	-0.57395577	-0.73939319
C	3.67047743	-2.44994371	-0.31642740
C	4.43255193	-0.11035681	-1.08587297
C	3.37672971	-0.92917171	-0.31519429
F	4.81633062	-2.73901082	0.33813085
F	2.65869342	-3.08806479	0.33954586
F	3.74085827	-2.96962026	-1.55562939
F	5.68187446	-0.36048455	-0.64667257
F	4.39146913	-0.34999472	-2.41633244
F	4.18759885	1.21353464	-0.90630180
H	1.38366233	-0.87401225	-0.25375384
H	3.42996147	-0.59615896	0.73575551
O	2.12817228	-0.66594855	-0.90257583
C	-0.84339834	-1.73379256	2.84745983
C	-0.86069595	-3.41007078	0.88029375
C	-0.14019079	-2.21275036	1.55568911
F	-1.13722783	-2.76229595	3.67422079
F	-0.02892355	-0.88288243	3.51412165
F	-1.99332312	-1.06786811	2.57781005
F	-0.64284055	-4.54978797	1.57697124
F	-2.20186639	-3.23401945	0.78684014
F	-0.38065110	-3.58787603	-0.36934840
H	-0.80946824	-0.83798024	0.25164013
H	0.84462070	-2.59121497	1.86178324
O	0.05847520	-1.12837774	0.69162244

h4m2-5.xyz
60
-3392.50545455087

C	-0.68092024	0.85652650	3.69707475
H	-0.25451322	-0.83322847	2.73707868
H	-0.34958135	0.54736686	4.69929085
H	0.12177405	1.42679014	3.20406501
H	-1.56509472	1.49574750	3.79609735
O	-1.06887670	-0.29196762	2.92133241
C	2.01939031	-2.44423655	2.96381761
H	1.74375199	-0.82589090	1.78154721
H	1.35057854	-3.20062707	3.38800625
H	2.78025834	-2.93708112	2.34484618
H	2.50540822	-1.88660629	3.77666665
O	1.20347073	-1.56737253	2.16190649
C	-0.83290930	1.88581032	-2.79912919
C	-0.84381004	3.46133747	-0.75097738
C	-0.12908620	2.29459241	-1.48399677
F	-1.11812212	2.95697370	-3.57330241
F	-0.02304430	1.06422510	-3.50610469
F	-1.98823500	1.21534122	-2.56507893
F	-0.60941071	4.63353185	-1.38601035
F	-2.18717784	3.29449912	-0.67653021
F	-0.37155858	3.56910877	0.50966589
H	-0.80557419	0.86080494	-0.24723173
H	0.85797103	2.68297067	-1.76957047
O	0.06357059	1.16600354	-0.67607508
C	-3.71872547	-1.07690716	0.08942011
C	-4.27970569	0.98044264	1.56122938
C	-3.39456517	0.39822787	0.43219260
F	-5.04329722	-1.26848118	-0.12144963
F	-3.07306274	-1.41876565	-1.05385879
F	-3.32030567	-1.92262679	1.06138550
F	-5.57547699	1.06417303	1.18320258
F	-4.21845884	0.24515271	2.69730908
F	-3.85916220	2.23232316	1.86471549
H	-1.73502058	0.12484906	1.61382662
H	-3.62629953	0.98512759	-0.46862105
O	-2.03778067	0.55950212	0.72441423
C	3.68635054	2.44999046	0.36189283
C	4.43408394	0.06529355	0.99556386
C	3.37021320	0.93521899	0.29532375
F	4.81553369	2.75667790	-0.31300815
F	2.66405078	3.13309388	-0.22950897
F	3.80218980	2.90587953	1.62252981
F	5.67478981	0.32075241	0.53496814
F	4.43252624	0.24078615	2.33709947
F	4.16513462	-1.24421500	0.75954334
H	1.37876959	0.88599732	0.26909631
H	3.38978006	0.65238765	-0.77131016
O	2.13295192	0.66440216	0.90256611
C	1.51687088	-2.73460721	-1.62801890
C	-0.24910728	-4.30624065	-0.59271342
C	0.07443488	-2.87368051	-1.08382241
F	1.71166604	-3.45783313	-2.75295344
F	1.76754201	-1.43485825	-1.93131910
F	2.44774699	-3.11961639	-0.71638739
F	0.08333741	-5.25595846	-1.49798089
F	0.40478897	-4.59008724	0.56654947
F	-1.57241246	-4.41454238	-0.34672323
H	0.38445433	-2.07308693	0.70381831
H	-0.59671818	-2.67263244	-1.93229207
O	-0.19887937	-1.93673338	-0.09055722

h4m2-6.xyz
60
-3392.50542496467

C	-0.58443704	0.98363446	-3.65719926
H	-0.23828586	-0.76416263	-2.76957070
H	-0.23230422	0.72399167	-4.66641332
H	0.22055888	1.49560087	-3.10751142
H	-1.44482583	1.65644394	-3.74271442
O	-1.03195686	-0.19624571	-2.96444334
C	1.97421884	-2.43679224	-2.95385999
H	1.72384152	-0.80777855	-1.77766655
H	1.28856170	-3.17153832	-3.38904022
H	2.71473576	-2.95415888	-2.33062164
H	2.48293273	-1.88772343	-3.75845951
O	1.17363720	-1.54298264	-2.15423145
C	-0.91885180	1.61713102	2.87662486
C	-0.96289718	3.37103706	0.97856363
C	-0.21840187	2.16521351	1.61087257
F	-1.24251375	2.60301753	3.74321030
F	-0.08899125	0.75847158	3.51411204
F	-2.05086245	0.93648757	2.57120793
F	-0.79524305	4.48257420	1.73238681
F	-2.29583267	3.15776157	0.84832858
F	-0.46481885	3.62282850	-0.25109654
H	-0.84726458	0.82230282	0.25408097
H	0.75485677	2.55394146	1.94000448
O	0.01173850	1.12338832	0.70395878
C	-3.72229328	-1.16276917	-0.24712734
C	-4.28786078	0.95911202	-1.62227182
C	-3.41891959	0.33482664	-0.50304362
F	-5.05067519	-1.39642786	-0.11616279
F	-3.12678652	-1.54687998	0.90776585
F	-3.25313897	-1.94914072	-1.23888158
F	-5.59165104	1.01329048	-1.26625645
F	-4.19719626	0.27616007	-2.78863299
F	-3.87510338	2.22749884	-1.86093911
H	-1.73149440	0.15687804	-1.65921582
H	-3.68377427	0.86891547	0.42115853
O	-2.05886009	0.53480550	-0.75253718
C	3.57313998	2.53578239	-0.28144105
C	4.39804571	0.26815069	-1.18816749
C	3.33789022	1.00645037	-0.34584483
F	4.73560337	2.83795183	0.33620243
F	2.56869434	3.09860626	0.45109552
F	3.56605580	3.11965145	-1.49346854
F	5.64937687	0.52849874	-0.76146018
F	4.31929309	0.58341292	-2.50037805
F	4.19541836	-1.07171636	-1.08202010
H	1.34675429	0.90791318	-0.24460442
H	3.43854514	0.62628304	0.68555193
O	2.08310686	0.71934711	-0.90821579
C	1.69046590	-2.62108047	1.57239280
C	-0.04958595	-4.30368506	0.67461065
C	0.22588011	-2.84747868	1.12469622
F	2.00146025	-3.32183473	2.68462163
F	1.88534363	-1.30599536	1.85337666
F	2.58226645	-2.95663692	0.60281201
F	0.38758987	-5.22023021	1.56969007
F	0.54666801	-4.57179085	-0.51896078
F	-1.37779169	-4.48421986	0.51264836
H	0.38259697	-2.05365414	-0.68453255
H	-0.39778063	-2.66961131	2.01385366
O	-0.16449862	-1.94486553	0.13961146

h4m2-7.xyz

60

-3392.50756372403

C	-1.86562061	-2.97280007	-2.25828664
H	-0.60426870	-1.36884819	-2.30216727
H	-1.13888648	-3.61615038	-2.77198977
H	-2.61507491	-2.60666671	-2.97410448
H	-2.36636640	-3.54897676	-1.47337288
O	-1.18864635	-1.87063780	-1.62627082
C	-0.04801102	0.39238563	-4.09972216
H	1.01543254	-0.07648603	-2.48052961
H	0.78015613	0.75668473	-4.72530061
H	-0.54896432	1.24686277	-3.62306220
H	-0.76682388	-0.13453478	-4.73793921
O	0.43359295	-0.55203017	-3.12904864
C	2.27232824	-3.48219308	-0.70926792
C	0.71997296	-4.08302139	1.27361426
C	1.52850231	-2.97814421	0.55093359
F	3.16529050	-4.45840074	-0.42592619
F	2.95211700	-2.45828314	-1.27525383
F	1.41275561	-3.96390948	-1.64770641
F	1.46622102	-5.17346820	1.56663011
F	-0.34045065	-4.49374769	0.52658047
F	0.23089096	-3.59523217	2.43550152
H	0.01781249	-2.12979738	-0.40611235
H	2.30626400	-2.64886246	1.25660933
O	0.71641468	-1.88383785	0.26127335
C	-4.52896620	-0.48391963	-0.01983442
C	-3.41454843	1.16887860	-1.67935535
C	-3.37867242	0.51738834	-0.27763414
F	-5.74210692	0.06837579	-0.24610805
F	-4.48855535	-0.88980758	1.26703039
F	-4.41950156	-1.58831768	-0.80244682
F	-4.53172238	1.90040256	-1.87255201
F	-3.33534598	0.25139689	-2.67833532
F	-2.34924380	1.99872459	-1.80912519
H	-1.91339977	-0.82108757	-0.70106236
H	-3.51393200	1.33628699	0.44385180
O	-2.14252991	-0.08953999	-0.02319096
C	3.88201584	1.51243079	-0.10378550
C	2.51085241	3.08058288	-1.64736062
C	2.94427777	1.62864182	-1.32987587
F	5.01379460	2.23234800	-0.26801147
F	4.23155795	0.22258886	0.07867626
F	3.27989769	1.93943801	1.03892524
F	3.56867871	3.88787723	-1.88174201
F	1.77689955	3.62568856	-0.64858731
F	1.74100418	3.07613579	-2.76432012
H	1.24574785	1.02594935	-0.42989441
H	3.53379417	1.28732084	-2.19427383
O	1.83910123	0.78196287	-1.20375833
C	0.29406636	0.64223489	2.95456934
C	-1.03503375	2.77107944	2.29458485
C	0.12363214	1.80690824	1.94780489
F	0.39541807	1.09350580	4.22777352
F	1.43287747	-0.03030451	2.66734464
F	-0.73340953	-0.22993965	2.89573001
F	-0.83690857	3.39470865	3.47419681
F	-2.23417024	2.12716757	2.35832385
F	-1.13869428	3.71784656	1.33524685
H	-0.82758726	0.77229646	0.49821616
H	1.04380113	2.40514994	2.00241458
O	-0.00160227	1.33326861	0.63447482

h4m2-8.xyz

60

-3392.50716507201

C	-0.70735359	0.69197613	3.92202726
H	-0.32835777	-0.84893613	2.71027001
H	-1.57082549	1.33752460	4.11607087
H	-0.44858685	0.15678379	4.84727930
H	0.14570274	1.31324613	3.60911031
O	-1.09237776	-0.23812787	2.89645644
C	1.72309583	-2.79554638	2.86881356
H	1.62129790	-1.17159318	1.67422774
H	0.98202482	-3.47353369	3.30541862
H	2.37896580	-3.36219235	2.19434689
H	2.31957860	-2.33691720	3.66994978
O	0.99534914	-1.78977975	2.13801254
C	-0.76282902	1.70413119	-2.91403390
C	0.21144007	3.44192180	-1.25692179
C	0.28126731	1.99810644	-1.81002105
F	-0.62377842	2.54095843	-3.96575845
F	-0.60423434	0.44147841	-3.36099851
F	-2.03683032	1.82070928	-2.45491529
F	0.42166576	4.36523431	-2.21760216
F	-0.96993473	3.71075468	-0.65874514
F	1.18608283	3.60126000	-0.31732759
H	-0.67627694	1.06495991	-0.27004591
H	1.26649070	1.89867161	-2.28923299
O	0.20507279	1.05482832	-0.78044073
C	-3.79315562	-0.30913959	0.12158963
C	-3.97964091	1.75016357	1.68744825
C	-3.22020923	1.07342537	0.52137429
F	-5.12242252	-0.24597790	-0.13478069
F	-3.18086070	-0.73065395	-1.01231852
F	-3.59135467	-1.24004576	1.07526201
F	-5.25972291	2.02875223	1.35440946
F	-3.99313717	0.98908169	2.80887254
F	-3.37166172	2.92083354	1.99874269
H	-1.61940479	0.43556575	1.63776289
H	-3.36104728	1.72914092	-0.35005448
O	-1.85020520	0.99733181	0.79663894
C	3.78157446	0.80264512	2.15407012
C	4.21754520	0.97754796	-0.38913726
C	3.17355884	1.00583876	0.75343060
F	4.77440985	1.67791704	2.40762369
F	2.81272721	0.99114930	3.09117260
F	4.26370342	-0.45011122	2.32509140
F	5.08679646	2.00899035	-0.29791192
F	4.91817098	-0.17051028	-0.43043047
F	3.55979591	1.10371493	-1.57671804
H	1.46006649	0.36856507	-0.00976327
H	2.73203927	2.01597820	0.74703312
O	2.21350766	0.00232972	0.55041665
C	1.02672944	-3.04739906	-1.60142199
C	-0.92068979	-4.33086494	-0.47461379
C	-0.40995339	-2.97937214	-1.02892468
F	1.14605031	-3.95454857	-2.59735765
F	1.36958956	-1.83927027	-2.10603225
F	1.93971810	-3.35900752	-0.64213041
F	-0.84276834	-5.33266250	-1.38027475
F	-0.21994259	-4.71248032	0.62904556
F	-2.21523216	-4.20719947	-0.10775289
H	0.04727019	-2.17658317	0.72078716
H	-1.06683126	-2.71903983	-1.87257195
O	-0.52553429	-1.97619929	-0.06794608

h5m1-1.xyz
66
-4066.93659806290
C 1.28177999 2.44061300 -2.79065383
H 1.41377501 1.41165337 -1.04833406
H 2.16186650 1.99386105 -3.26876856
H 1.54315698 3.40881420 -2.34457862
H 0.49051596 2.58062461 -3.53370717
O 0.75580758 1.54738938 -1.78340945
C -3.42568728 -1.74580613 -0.72762221
C -4.23225670 -0.22365609 1.21317332
C -3.11791825 -1.14757216 0.66724574
F -4.63845362 -2.34589842 -0.76091136
F -2.49675004 -2.68659445 -1.02234726
F -3.39132877 -0.80644233 -1.69557150
F -5.39430797 -0.88298068 1.39459648
F -4.46947468 0.82857047 0.38236420
F -3.85181987 0.28661133 2.40663885
H -1.87476357 0.33402431 0.08261156
H -3.04756038 -1.99359060 1.36527134
O -1.88468461 -0.48249719 0.67213179
C 2.04322968 -2.09762312 -2.64314559
C -0.05879127 -1.82359611 -4.11640266
C 0.49819454 -2.01683677 -2.68395221
F 2.51537472 -3.19792296 -3.26804603
F 2.46378957 -2.15072968 -1.35241157
F 2.62686654 -1.00969329 -3.20962632
F 0.46244312 -2.70381316 -5.00212644
F 0.19358726 -0.56915865 -4.57750272
F -1.39914206 -1.98985482 -4.10780786
H 0.34272048 -0.13387222 -2.09010140
H 0.12718393 -2.98978673 -2.32895988
O 0.00764740 -1.03235007 -1.82800567
C 3.92115189 0.80352937 2.09604324
C 4.30506261 1.46242961 -0.36743147
C 3.52870110 0.56479270 0.61699175
F 5.20187632 0.44542752 2.33512584
F 3.12930787 0.01966184 2.88500496
F 3.75008652 2.07760848 2.48774073
F 5.63789903 1.35835706 -0.19380141
F 3.95886146 2.76327729 -0.25496926
F 4.02200491 1.07615526 -1.63900560
H 1.61529230 0.05338424 0.79537709
H 3.80963470 -0.47548933 0.37795226
O 2.15927699 0.81213749 0.42356212
C -2.28403086 4.06733175 -1.04276399
C -0.84906070 3.39058984 1.01053328
C -1.93600448 2.97210919 -0.00818354
F -2.59552301 5.24219426 -0.45320799
F -3.34815325 3.67042659 -1.77379312
F -1.25658591 4.28884271 -1.90311567
F -1.26497252 4.40862196 1.79330259
F 0.30520429 3.76881551 0.40511170
F -0.56354506 2.33814085 1.81150023
H -0.69505980 1.83534940 -1.12503067
H -2.85147815 2.79049170 0.57316211
O -1.59969283 1.77957495 -0.66946321
C 0.37460500 -3.45095933 2.05177456
C -0.11592047 -1.46158819 3.63194173
C 0.70380321 -1.98637731 2.42518967
F 0.40867470 -4.26999090 3.12538278
F 1.28221856 -3.90065500 1.15488394
F -0.85099691 -3.55939173 1.47844468
F 0.27953230 -2.06101959 4.77806543
F -1.44929469 -1.67906409 3.48939150
F 0.07118952 -0.13391673 3.76797405
H -0.36959580 -1.03923808 1.01831991
H 1.75384713 -1.97518657 2.74775655
O 0.59053515 -1.16802418 1.29278785

h5m1-2.xyz
66
-4066.93660402062
C -1.30839355 2.48313233 -2.73995945
H -1.42696442 1.41565934 -1.01998089
H -2.18080875 2.03110390 -3.22713603
H -1.58573097 3.43837603 -2.27599115
H -0.51914828 2.64976483 -3.47960226
O -0.76885111 1.57987763 -1.74909695
C 3.46667453 -1.66171325 -0.75488947
C 4.21059798 -0.20855389 1.26223259
C 3.12226323 -1.12688492 0.65709475
F 4.69131775 -2.23733522 -0.78891508
F 2.56170243 -2.60645224 -1.10519539
F 3.43203336 -0.68455219 -1.68492651
F 5.37563366 -0.86111090 1.45025666
F 4.45642632 0.87438393 0.47426632
F 3.79435407 0.25576426 2.46215355
H 1.86181083 0.35197291 0.10492903
H 3.05421876 -2.00222093 1.31819296
O 1.87721729 -0.48420562 0.66592402
C -0.34425138 -3.54761803 1.90631393
C 0.10403735 -1.61098706 3.56135842
C -0.70008669 -2.10274961 2.33015657
F -0.35664563 -4.40290760 2.95176191
F -1.24661632 -3.98572362 0.99850234
F 0.88042029 -3.61145995 1.32445303
F -0.30011262 -2.24884294 4.68353358
F 1.44008336 -1.81779091 3.42569048
F -0.09073481 -0.28946346 3.73795810
H 0.36736823 -1.08632970 0.96823377
H -1.75227899 -2.11966308 2.64552810
O -0.59232850 -1.24232211 1.22919536
C -3.90108032 0.72658539 2.12620375
C -4.31034544 1.46833717 -0.30941463
C -3.53410721 0.52838140 0.63473152
F -5.18336184 0.37982185 2.37397519
F -3.10913020 -0.09267177 2.87788906
F -3.70536317 1.98554368 2.55373808
F -5.64155449 1.38586348 -0.11348459
F -3.93593167 2.75835796 -0.16684295
F -4.05857162 1.11285052 -1.59644062
H -1.62408371 -0.01312695 0.77266329
H -3.83467213 -0.49959063 0.36778102
O -2.16433920 0.76128538 0.42840705
C 2.23228294 4.11493967 -0.94420570
C 0.80195150 3.38411099 1.09279255
C 1.89665075 2.99705701 0.06990519
F 2.52812869 5.28300043 -0.33346914
F 3.30194393 3.74496350 -1.68129567
F 1.20260038 4.33842409 -1.80142390
F 1.20296748 4.39419376 1.89364414
F -0.35511430 3.75808825 0.49016946
F 0.52757795 2.31505027 1.87509575
H 0.67464892 1.86948468 -1.07552888
H 2.81306958 2.81602215 0.65003384
O 1.57595458 1.81289978 -0.61368082

C -2.01127953 -2.04842881 -2.69102196
C 0.08186676 -1.71030181 -4.16538038
C -0.46749496 -1.95084032 -2.73745497
F -2.47912411 -3.12085599 -3.36551707
F -2.42263918 -2.16674815 -1.40190772
F -2.60772911 -0.93891865 -3.20016072
F -0.41907841 -2.58210873 -5.07069315
F -0.20152458 -0.45189962 -4.59707512
F 1.42587438 -1.84375124 -4.16171131
H -0.33097805 -0.08390717 -2.09213148
H -0.08436234 -2.92961460 -2.41229766
O 0.01561600 -0.98546018 -1.85591719

h5m1-5.xyz
66
-4066.93659569496
C 1.27741663 2.43182868 -2.80562393
H 1.40832672 1.40755681 -1.06109810
H 2.16076285 1.98453372 -3.27745178
H 1.53466252 3.40319256 -2.36396900
H 0.48919658 2.56585012 -3.55296157
O 0.74958483 1.54316101 -1.79560590
C -3.40546036 -1.75823640 -0.70712159
C -4.22927307 -0.23444786 1.22534870
C -3.10697193 -1.15275461 0.68644272
F -4.61403840 -2.36620280 -0.74351116
F -2.46927071 -2.69474481 -0.99342507
F -3.37223935 -0.82260438 -1.67858965
F -5.38862977 -0.90047357 1.40067431
F -4.46768644 0.81591682 0.39302201
F -3.85832070 0.27795059 2.42094718
H -1.86956364 0.33195096 0.09675453
H -3.03348916 -1.99562916 1.38800477
O -1.87796934 -0.47961719 0.69339577
C 2.02168526 -2.12576116 -2.62917177
C -0.07475415 -1.84310203 -4.10792006
C 0.47753180 -2.03023728 -2.67306581
F 2.48303388 -3.23695316 -3.24332741
F 2.44019148 -2.17076347 -1.33782409
F 2.61659676 -1.04927362 -3.20556633
F 0.44291305 -2.73260910 -4.98621494
F 0.18699575 -0.59344470 -4.57696643
F -1.41630141 -2.00034165 -4.10176510
H 0.33397218 -0.14033438 -2.09701365
H 0.09653373 -2.99662008 -2.31083435
O -0.00498054 -1.03438066 -1.82564414
C 3.91779398 0.84321783 2.07892333
C 4.30399371 1.44889260 -0.39799917
C 3.52845617 0.57114819 0.60468548
F 5.19694226 0.48739427 2.32957680
F 3.12143748 0.08031278 2.88363768
F 3.74947095 2.12669575 2.44057072
F 5.63649405 1.35874558 -0.21447282
F 3.94814090 2.74996661 -0.31923075
F 4.03038483 1.02981420 -1.66090167
H 1.61710697 0.05108124 0.78101382
H 3.81264325 -0.47335854 0.38901419
O 2.15862007 0.80962769 0.40430901
C -2.30887590 4.04869347 -1.05735828
C -0.84022890 3.40852821 0.98247231
C -1.93756738 2.96797848 -0.01549709
F -2.61348697 5.23003356 -0.47689682
F -3.38495498 3.63960128 -1.76357940
F -1.29808870 4.26226574 -1.93890240
F -1.25779471 4.42680143 1.76399023
F 0.29887735 3.79808272 0.35568859
F -0.52751469 2.36652559 1.78669397
H -0.70039270 1.82754436 -1.13260064
H -2.84323100 2.78547719 0.58082429
O -1.60057235 1.77100238 -0.66830655
C 0.39940752 -3.43755154 2.09228915
C -0.10399679 -1.42349536 3.63544404
C 0.71963534 -1.96413178 2.43798726
F 0.42618598 -4.23344558 3.18353510
F 1.31686831 -3.90306925 1.21363887
F -0.82031545 -3.56215576 1.51013230
F 0.30914746 -1.98641361 4.79391013
F -1.43363441 -1.67013978 3.50612629
F 0.05830454 -0.08964255 3.73755585
H -0.36011686 -1.03975251 1.02096901
H 1.76953133 -1.94021128 2.76048472
O 0.60162782 -1.16862186 1.28988426

h5m1-6.xyz
66
-4066.93660151606
C -1.25368027 -2.47562567 -2.76582219
H -1.39828923 -1.43916924 -1.02936651
H -2.14075451 -2.04184867 -3.24295759
H -1.50103751 -3.44601500 -2.31641746
H -0.46243469 -2.60703081 -3.51043769
O -0.73720238 -1.57271679 -1.76207489
C 3.41089015 1.76633685 -0.73364208
C 4.23365911 0.26933656 1.21907630
C 3.10879119 1.17643718 0.66610916
F 4.61921007 2.37507106 -0.77217098
F 2.47505499 2.69801785 -1.03485571
F 3.38286353 0.81947540 -1.69455989
F 5.38522269 0.94610938 1.40351138
F 4.48955940 -0.78173888 0.39255485
F 3.85576474 -0.24318804 2.41257740
H 1.88071498 -0.32157137 0.09203643
H 3.02963847 2.02739582 1.35714509
O 1.88266046 0.49862991 0.67667124
C -2.05760842 2.05718655 -2.65531610
C 0.05110737 1.78637657 -4.11872570
C -0.51179067 1.98812748 -2.68973820
F -2.53512860 3.15151596 -3.28656767
F -2.48418731 2.11218492 -1.36666966
F -2.63060702 0.96258280 -3.21998589
F -0.48176393 2.64779951 -5.01582212
F -0.18114422 0.52303030 -4.56538130
F 1.38873966 1.97311896 -4.10981688
H -0.34076222 0.11180632 -2.07944549
H -0.14958341 2.96715405 -2.34246353
O -0.01595488 1.01559381 -1.82318123
C -3.93026503 -0.82533089 2.09679187
C -4.29839836 -1.50361989 -0.36345770
C -3.53071045 -0.59532310 0.61808231
F -5.20946565 -0.45639082 2.32869042
F -3.13560467 -0.04468994 2.88602142
F -3.77092722 -2.09916830 2.49443565
F -5.63213721 -1.41356113 -0.18885677
F -3.93813828 -2.80027884 -0.24730260
F -4.02055444 -1.11818586 -1.63637237
H -1.62093109 -0.07469178 0.80292913

H -3.81494279 0.44208289 0.37050280
O -2.15936789 -0.83802385 0.43285356
C 2.34624745 -4.05297821 -0.99903683
C 0.87823203 -3.38554835 1.03299140
C 1.96966323 -2.95575059 0.02371217
F 2.65439479 -5.22313665 -0.39812147
F 3.42194951 -3.65212326 -1.71081179
F 1.33728393 -4.28501277 -1.87795629
F 1.30832370 -4.38118173 1.83656295
F -0.25739008 -3.80159440 0.41700095
F 0.55465727 -2.32930597 1.81420218
H 0.71827131 -1.84232282 -1.10512404
H 2.87545467 -2.75556378 0.61418504
O 1.62240222 -1.77360866 -0.65050324
C -0.43441741 3.45889829 1.98961687
C 0.10934183 1.52730197 3.62305177
C -0.72883341 1.99874990 2.40719195
F -0.47427974 4.30798474 3.03925670
F -1.36070605 3.86396205 1.09025252
F 0.78243614 3.57517161 1.40001309
F -0.28959577 2.15326257 4.75363508
F 1.43726004 1.76712320 3.46306927
F -0.04959355 0.20100562 3.80085754
H 0.35912959 1.03701927 1.02168584
H -1.77663838 1.97262113 2.73639091
O -0.60194390 1.15088391 1.29865505

h1a2-10.xyz

32

-1176.74154732250

C -2.51357830 -0.75720866 -0.17573684
C -1.18240358 1.42293026 0.24928721
C -1.66194938 0.37969141 -0.78977994
F -3.65214448 -0.28957880 0.39125274
F -2.86775559 -1.63039673 -1.14409551
F -1.83577588 -1.45466729 0.77485042
F -2.18310569 1.90637206 1.02013742
F -0.22587612 0.92478803 1.07324609
F -0.63118605 2.47877826 -0.40823508
H -0.02097792 -0.73744686 -1.02671976
H -2.34102271 0.92493336 -1.46519773
O -0.59726085 -0.09936969 -1.54391863
C 1.92165233 -2.14570554 0.27525999
O 1.22843374 -1.81673938 -0.68693123
C 1.54650236 -1.81478084 1.69364102
C 3.21107716 -2.89418943 0.06437880
H 3.37033819 -3.08999770 -1.00090098
H 4.03621598 -2.28315532 0.45891805
H 3.20388081 -3.84017372 0.62616748
H 0.51483202 -1.45863469 1.75606957
H 1.69403674 -2.68311934 2.35106198
H 2.22699082 -1.02185021 2.04135531
C 3.09562734 1.27178046 -0.22781128
O 3.51202325 0.37821003 0.49988306
C 2.74800568 1.02711208 -1.67612952
C 2.89502943 2.67906547 0.28982953
H 3.03332132 2.70955000 1.37565545
H 3.62350288 3.35147894 -0.19035953
H 1.89576754 3.04999079 0.02176363
H 3.11686916 0.04946417 -2.00222848
H 1.65161804 1.04262702 -1.78949187
H 3.14712327 1.82622355 -2.31767959

h1a3-1.xyz

42

-1370.00570943617

C 2.68730783 -0.75930867 0.43154460
C 1.69288904 1.50518191 -0.29556557
C 1.57846697 -0.03403462 -0.35251570
F 2.74916536 -0.33691141 1.72298393
F 2.42368753 -2.09131620 0.45629122
F 3.91236085 -0.59797476 -0.11310493
F 1.49104902 1.98846445 0.96359573
F 2.87644285 1.98021956 -0.73206962
F 0.72097565 2.04939341 -1.08277751
H 0.67417506 -0.62227292 -1.96464838
H 0.63290994 -0.29244281 0.16073614
O 1.61963846 -0.47119040 -1.68120323
C -1.47894107 -1.92967231 -1.66595149
O -1.00319708 -0.87239951 -2.08801417
C -2.96512327 -2.04887671 -1.44603627
C -0.62323260 -3.13024812 -1.38168674
H 0.42613933 -2.94742107 -1.63469441
H -0.70866099 -3.36444942 -0.31052332
H -1.00696790 -3.99983966 -1.93740645
H -3.31789844 -1.18480515 -0.86468698
H -3.46742390 -2.01616612 -2.42532677
H -3.23881659 -2.98148199 -0.93983456
C -1.75614625 -0.86131594 2.07808835
O -1.07429071 -1.25004058 1.13628939
C -1.34381307 0.31983140 2.92158313
C -3.04957457 -1.54274859 2.44976341
H -3.11697032 -2.52809609 1.97631148
H -3.16110645 -1.63109033 3.53960938
H -3.87101815 -0.90776225 2.08328572
H -0.52484871 0.87089390 2.44809805
H -2.20335401 0.98027759 3.10025695
H -1.00639810 -0.04529936 3.90505967
C -3.05493657 1.78826082 -0.31091037
O -3.56410985 0.88917084 0.35069444
C -3.07023176 1.75616881 -1.81990437
C -2.35570825 2.95627104 0.34152677
H -2.35970228 2.85581833 1.43142417
H -1.32140893 3.02530992 -0.02673851
H -2.85449828 3.89448027 0.05306716
H -2.22973779 1.11849767 -2.14394267
H -3.99557949 1.28815538 -2.17697578
H -2.94318057 2.75040843 -2.26583644

h1a3-9.xyz

42

-1370.00570905364

C -2.72594717 0.73657416 -0.40367111
C -1.68573212 -1.52422748 0.27038928
C -1.59217011 0.01540694 0.34805149
F -2.80417034 0.33614973 -1.70095039
F -2.48265950 2.07293297 -0.40917022
F -3.93860897 0.54764894 0.15929261
F -1.50428591 -1.98562998 -0.99956400
F -2.85260842 -2.02307783 0.72519591
F -0.68925840 -2.06509746 1.02885609
H -0.66494162 0.60084328 1.94821646
H -0.65975258 0.29319512 -0.17911520
O -1.61284005 0.43159951 1.68401100
C 3.13566342 -1.73915778 0.36675747
O 3.62497875 -0.84463227 -0.31570583

C 3.15463801 -1.67393226 1.87472306
C 2.45665115 -2.93417709 -0.25767319
H 2.46204998 -2.86093678 -1.34973597
H 1.42209892 -3.00896197 0.10912352
H 2.96848261 -3.85724063 0.05553439
H 2.30005186 -1.04887368 2.18627719
H 4.06964890 -1.17655718 2.21862229
H 3.05204902 -2.66071400 2.34300221
C 1.75171461 0.83753078 -2.08786360
O 1.04626205 1.24454755 -1.17135790
C 3.03295931 1.53708986 -2.46763729
C 1.38311246 -0.38614024 -2.88989313
H 0.57392295 -0.94217571 -2.40561956
H 2.26355924 -1.02749084 -3.03232320
H 1.04862358 -0.06842488 -3.89071477
H 3.06308797 2.54597758 -2.04236049
H 3.16518804 1.57502631 -3.55800025
H 3.86339924 0.94431198 -2.05364610
C 1.44231258 1.96695492 1.60997477
O 1.00817701 0.89441158 2.03828272
C 0.54327528 3.13984794 1.34325220
C 2.91915090 2.13964473 1.36713136
H 3.32827595 1.23626261 0.89470287
H 3.41601792 2.26583616 2.34263654
H 3.13575384 3.02196900 0.75414834
H 0.56424897 3.34175287 0.26207066
H 0.93357424 4.03569994 1.84982349
H -0.48636704 2.93907321 1.65627938

h1a3-10.xyz
42
-1370.00571091660
C -2.66827614 0.75805191 0.43883320
C -1.68056598 -1.50586944 -0.29990867
C -1.56836316 0.03321550 -0.35785011
F -2.70303988 0.34622027 1.73498600
F -2.41372998 2.09167668 0.44722143
F -3.90229180 0.58351313 -0.08125615
F -1.47512443 -1.98876188 0.95904900
F -2.86405306 -1.98291814 -0.73376070
F -0.70964450 -2.04909839 -1.08912569
H -0.67964210 0.62803653 -1.97461589
H -0.61772499 0.29269908 0.14543730
O -1.62221808 0.46901191 -1.68663374
C 1.47134113 1.94159867 -1.66423596
O 0.99997197 0.88280668 -2.08731269
C 2.95417853 2.06333769 -1.42595735
C 0.61272125 3.14468255 -1.39931025
H -0.42758242 2.97080253 -1.69277496
H 0.65867249 3.36322040 -0.32230070
H 1.02132410 4.01981107 -1.92754319
H 3.32246026 1.16265202 -0.91547839
H 3.45758511 2.12491145 -2.40399978
H 3.20854307 2.95888105 -0.84764393
C 1.74611721 0.86827574 2.07207013
O 1.06763088 1.26983263 1.13317735
C 3.04564981 1.53531760 2.44792712
C 1.32385321 -0.31549643 2.90690631
H 0.50283810 -0.85893920 2.42835636
H 2.17893690 -0.98200288 3.08436735
H 0.98561500 0.04615424 3.89139140
H 3.11649200 2.52825758 1.99117720
H 3.16525647 1.60339059 3.53823545
H 3.86012114 0.90162571 2.06381468
C 3.03067476 -1.80353571 -0.31118897
O 3.54688751 -0.90046952 0.33951675
C 2.34403516 -2.97086084 0.35561554
C 3.02566644 -1.77712158 -1.82046522
H 2.19502572 -1.12251539 -2.13566804
H 3.95540062 -1.32851332 -2.19121680
H 2.87339356 -2.76978040 -2.26180140
H 2.36308859 -2.86634556 1.44500059
H 1.30513471 -3.04475985 0.00170193
H 2.84209357 -3.90849663 0.06371020

h1a4-1.xyz
52
-1563.26560101473
C -2.57156699 0.68158661 0.86030053
C -1.36986896 2.19228223 -0.82662338
C -1.35247271 0.84174454 -0.06551097
F -2.73199488 1.75451481 1.67291767
F -2.40673313 -0.40657404 1.65877209
F -3.72677841 0.50016338 0.17954611
F -1.12545516 3.23737657 0.00985824
F -2.53849285 2.43702193 -1.46169273
F -0.39077669 2.19402758 -1.76505072
H -0.43649055 -0.36194619 -1.32589473
H -0.46336316 0.85814332 0.59396006
O -1.35462251 -0.24403469 -0.94689192
C -1.53229695 -3.31166591 -0.47536333
O -0.55744357 -3.41981557 0.26027828
C -1.39480381 -3.30620282 -1.98081529
C -2.93393592 -3.18336313 0.06821223
H -2.92586874 -3.19536690 1.16235002
H -3.56272929 -4.00313470 -0.31206894
H -3.37806197 -2.24340266 -0.28893588
H -0.34988407 -3.44865500 -2.27429486
H -1.76370016 -2.34739036 -2.37343508
H -2.01783765 -4.09759595 -2.42410637
C 2.06113728 -1.09923457 -2.10409235
O 1.00509860 -0.47356769 -2.21158655
C 3.21429640 -0.80237790 -3.02461602
C 2.28502052 -2.13086914 -1.03795269
H 1.35653265 -2.39539265 -0.51976640
H 3.01072397 -1.70191570 -0.32962610
H 2.75217601 -3.03291438 -1.45890826
H 2.91313137 -0.09606361 -3.80524284
H 3.60857643 -1.72596127 -3.47240871
H 4.02052607 -0.36653184 -2.41249767
C 3.71068341 1.45364742 0.39055537
O 4.21331206 0.42982163 -0.06045642
C 4.10557974 1.99455753 1.74581389
C 2.68680394 2.25248818 -0.37609214
H 2.27838898 1.67654660 -1.21162701
H 3.16582278 3.16453820 -0.76826535
H 1.87397140 2.57741870 0.28631243
H 4.96642089 1.44684154 2.14416231
H 3.24673015 1.88155298 2.42427882
H 4.33216546 3.06956878 1.68852652
C 1.48093125 -0.91228555 2.34340380
O 1.33412928 0.14333647 1.73281065
C 2.84470716 -1.54302858 2.51011357
C 0.31675642 -1.64914986 2.94932334
H -0.49833771 -0.95690585 3.18259140
H 0.61316533 -2.21972137 3.83976353

```
H -0.05356458 -2.37120257  2.19849467
H  3.59118024 -1.04923556  1.87861513
H  2.79844330 -2.61836049  2.28306284
H  3.14627398 -1.46062055  3.56690673
```

h1a4-4.xyz

```
52
-1563.26723075980
C -1.47857799  0.26854731  1.49491613
C -3.34503301 -0.15754302 -0.23289485
C -1.82814450 -0.00441482  0.02159868
F -1.96981507 -0.68297150  2.32796826
F -0.12400214  0.26941116  1.64093944
F -1.92641593  1.46740794  1.93312123
F -3.86462290 -1.22467794  0.42836414
F -4.05390853  0.93724063  0.12892530
F -3.55292786 -0.36050203 -1.55676480
H -0.89137130  0.66529835 -1.56957810
H -1.36701649 -0.98003266 -0.22712413
O -1.31313266  1.05133600 -0.74152985
C  1.35673013  3.31267793 -0.55143851
O  2.42001476  2.82943815 -0.17617196
C  0.29851389  3.77130112  0.41782654
C  1.02700437  3.44498210 -2.02127512
H  1.88861208  3.16275454 -2.63582101
H  0.17137748  2.79588016 -2.26179659
H  0.71706220  4.47292599 -2.25988987
H  0.65385831  3.68473884  1.44968787
H  0.00057223  4.80887570  0.20550404
H -0.59529145  3.14366353  0.27818631
C  0.79759176 -3.17777961  0.08339528
O  0.08973805 -2.60772832 -0.74213995
C  2.08073231 -3.86536802 -0.31928943
C  0.47009270 -3.17798766  1.55380661
H -0.54750016 -2.81685012  1.73059285
H  0.60286817 -4.17748904  1.99140661
H  1.19228628 -2.50893467  2.05021574
H  2.19035817 -3.87737470 -1.40917003
H  2.92122211 -3.31972637  0.13872573
H  2.11296020 -4.89324442  0.07025032
C  0.96825900 -0.52066309 -2.71026709
O -0.15387936 -0.03051032 -2.85428163
C  1.84712012 -0.18125173 -1.54153325
C  1.51237641 -1.47578496 -3.73641976
H  0.93550614 -1.41912498 -4.66544213
H  2.58127715 -1.30250534 -3.92328533
H  1.41173744 -2.48862792 -3.31496333
H  1.25897096  0.17003980 -0.68819984
H  2.47596346 -1.02796937 -1.24363950
H  2.51985235  0.63871425 -1.84280415
C  3.63695937 -0.11325992  1.51237503
O  3.25441422 -1.27252811  1.39077885
C  4.72396911  0.45994044  0.63312525
C  3.03037015  0.82832781  2.52171376
H  3.80987890  1.21620459  3.19529666
H  2.61491160  1.69675642  1.98597011
H  2.24895596  0.32645656  3.10057723
H  5.11099473 -0.30494797 -0.04869636
H  4.31403943  1.31073443  0.06715633
H  5.54341618  0.85913107  1.24983793
```

h1a4-5.xyz

```
52
-1563.26727343510
C -1.47356080 -0.19030360 -1.52357731
C -3.29685949  0.26830537  0.24102269
C -1.78882227  0.08206812 -0.04320558
F -1.95700770  0.77751224 -2.34161384
F -0.12308858 -0.22564309 -1.69556760
F -1.96119164 -1.37560789 -1.95953374
F -3.80553514  1.35097914 -0.40230727
F -4.03702553 -0.80819175 -0.11443827
F -3.47360026  0.46536807  1.57016357
H -0.86907286 -0.61457144  1.54293954
H -1.30034759  1.04456935  0.20261580
O -1.28297912 -0.98921486  0.70403518
C  1.07582348 -3.35411584  0.59344386
O  2.17520317 -2.87645318  0.33235595
C  0.13275974 -3.82721401 -0.48273353
C  0.58622082 -3.48440578  2.01745030
H  1.29356967 -3.02360915  2.71468355
H -0.40520575 -3.02046542  2.11841475
H  0.46593147 -4.54943260  2.27038074
H  0.61834330 -3.80623269 -1.46380667
H -0.23643515 -4.83991494 -0.26281993
H -0.74236605 -3.15984434 -0.48697748
C  0.97928828  3.14932082 -0.14418013
O  0.22303752  2.64332200  0.67990911
C  0.67740131  3.13710978 -1.62004763
C  2.29668594  3.76435071  0.26636612
H  2.38768620  3.79555191  1.35761975
H  3.10821816  3.15520565 -0.16304243
H  2.40131213  4.77840963 -0.14585181
H -0.35837937  2.83831993 -1.80771745
H  0.88239502  4.11574506 -2.07676968
H  1.36313747  2.41245434 -2.08924461
C  0.97070446  0.55387345  2.70695209
O -0.16935939  0.10127214  2.83168514
C  1.52140633  1.50758978  3.73076166
C  1.86352685  0.16571258  1.56395796
H  1.28004012 -0.13176717  0.68722793
H  2.56346924  0.96589312  1.29874071
H  2.45657764 -0.71097520  1.87346536
H  2.57350984  1.28801164  3.96049791
H  1.48527797  2.51362976  3.28357855
H  0.91067461  1.49908652  4.63961084
C  3.66588221 -0.10046542 -1.45254240
O  3.36349845  1.08633750 -1.38598941
C  3.00550295 -1.04236983 -2.42700594
C  4.70205754 -0.70932472 -0.53605705
H  5.11447047  0.04918711  0.13789679
H  4.23909457 -1.52666963  0.03753335
H  5.51327725 -1.16025918 -1.12792911
H  2.28567367 -0.51015322 -3.05640577
H  3.76316788 -1.53797020 -3.05292410
H  2.50109171 -1.83709397 -1.85385118
```

h1a5-1.xyz

```
62
-1756.53345827803
C -1.87057378 -2.15890032  0.67112728
C -2.47762625 -0.98785203 -1.54031389
C -1.48001288 -1.08664192 -0.36891556
F -1.89153755 -3.40682947  0.14604111
F -0.94895534 -2.16029106  1.67500499
```

F -3.07665318 -1.92692058 1.24229225
F -2.62758077 -2.16819109 -2.18897403
F -3.70442779 -0.57011252 -1.14858699
F -2.01791210 -0.08390222 -2.44678814
H -0.43728922 0.44779870 0.31098120
H -0.51256558 -1.40684057 -0.80177031
O -1.40668143 0.16170937 0.25722727
C 1.72776454 1.15637989 1.31721873
O 1.03701763 1.05342532 0.29810857
C 3.03648118 1.88573325 1.27411499
C 1.29607979 0.58174213 2.63260202
H 0.48163376 -0.13799577 2.49988056
H 2.14925467 0.11468564 3.14061997
H 0.92945957 1.41759403 3.25015242
H 3.16845678 2.39216322 0.31266743
H 3.09673703 2.60474605 2.10410149
H 3.83967080 1.14811581 1.42686090
C -1.90273814 2.60230326 2.19497740
O -0.77287369 3.06441738 2.30981762
C -2.82743637 3.03071028 1.08012553
C -2.42904352 1.54368914 3.13472566
H -1.71226730 1.35046196 3.93957165
H -3.39789894 1.84611778 3.55864231
H -2.60054938 0.62109769 2.55988171
H -2.41575353 3.89629194 0.55087530
H -2.93233619 2.18558931 0.38209757
H -3.83176379 3.26162370 1.46306736
C 3.54612945 -2.37163979 1.14616916
O 3.90358726 -1.27689172 1.56613053
C 2.29461797 -3.04806234 1.64655867
C 4.32334857 -3.10501867 0.07700085
H 4.64719077 -4.08822757 0.45120918
H 3.66457141 -3.28693224 -0.78496897
H 5.19653718 -2.52168024 -0.23329071
H 1.92208966 -2.55728415 2.55109716
H 1.53463905 -2.96826554 0.85502456
H 2.46018989 -4.11865242 1.83240653
C 2.11259964 -0.64166592 -2.02259102
O 1.64447727 -1.51797677 -1.29867731
C 1.37079350 -0.11397862 -3.22232587
C 3.46938821 -0.03272342 -1.77010626
H 3.86213089 -0.33200245 -0.79348844
H 4.16466479 -0.35926727 -2.56042417
H 3.39973636 1.06203615 -1.84154974
H 0.39030300 -0.58829459 -3.32488620
H 1.27303201 0.97855531 -3.12663641
H 1.96645993 -0.29064510 -4.13181046
C 0.90218443 3.49548540 -1.54649461
O 1.91666836 3.10205679 -2.11049278
C 0.95320023 4.42848055 -0.36169977
C -0.47440542 3.05905884 -1.99645204
H -0.44330725 2.67628106 -3.02214778
H -0.81756280 2.25000437 -1.33281681
H -1.20614636 3.87476574 -1.91808462
H 1.99066259 4.63287889 -0.07679857
H 0.45402005 5.37608081 -0.61979535
H 0.39626003 4.00405020 0.49006548

h1a5-3.xyz
62
-1756.53067249312
C 2.04193583 -1.71672298 -1.49064162
C 1.17082232 -2.52583920 0.79105747
C 0.83522009 -1.81096847 -0.53483704
F 3.10034641 -1.07705237 -0.91668451
F 1.69248036 -0.99236465 -2.58768196
F 2.47530991 -2.91835953 -1.92169340
F 2.11054475 -1.84399431 1.51082686
F 1.61852519 -3.78715244 0.62812598
F 0.05470610 -2.58581080 1.56491588
H -0.99725407 -1.87322856 -1.20606466
H 0.57139612 -0.77187188 -0.25730609
O -0.20493536 -2.48603672 -1.18117182
C 0.52847508 3.53549090 -1.80767402
O -0.59939595 3.46008857 -2.28435239
C 0.87470811 4.53667329 -0.73479334
C 1.63076533 2.59766896 -2.24427255
H 1.41083483 2.17137567 -3.22903016
H 1.66913304 1.77718861 -1.51223341
H 2.61428603 3.08600650 -2.25131340
H 0.02694360 5.20345136 -0.54442808
H 1.75541061 5.12665377 -1.03000289
H 1.14321494 4.00977280 0.19722199
C 2.34654152 2.15783190 2.42644625
O 1.59259784 3.12214522 2.38638597
C 2.46662935 1.29307068 3.66104257
C 3.18294508 1.75038114 1.23731902
H 3.27823806 2.58094022 0.53056076
H 2.67072241 0.91611132 0.73341660
H 4.17328968 1.38202200 1.53718647
H 1.73562035 1.59666009 4.41746043
H 3.48262594 1.38572033 4.07585779
H 2.33073599 0.23300950 3.40025412
C -2.31642817 0.20299054 -1.86568296
O -2.24293259 -0.76550791 -1.10144417
C -1.45604246 0.32777028 -3.08601647
C -3.29500163 1.31294554 -1.60524643
H -3.72868174 1.22601658 -0.60333787
H -4.09870703 1.26345300 -2.35782464
H -2.79794312 2.28384788 -1.74090183
H -0.76887921 -0.51770286 -3.18628578
H -0.90771143 1.28206236 -3.02946756
H -2.09708137 0.39919904 -3.97889188
C -4.21578612 -1.05961491 1.20931918
O -4.07954141 0.08326219 1.62970463
C -5.22911318 -1.39633649 0.13878782
C -3.38144695 -2.20734921 1.72585318
H -2.90233206 -1.94414864 2.67460130
H -2.59809746 -2.42243408 0.98299649
H -3.97869161 -3.12258091 1.84118114
H -5.70033229 -0.48591122 -0.24594152
H -6.00257522 -2.05762848 0.56009345
H -4.74321711 -1.94859459 -0.67810512
C -0.78591735 1.54361191 1.17877104
O -0.08749832 1.19799605 0.22681104
C -0.90366789 0.71653490 2.42859361
C -1.58545117 2.81953166 1.15391999
H -1.58904960 3.26130266 0.15265281
H -1.12480269 3.52513162 1.86288307
H -2.61162267 2.63012386 1.49951421
H -0.21508078 -0.13345443 2.41231756
H -1.94443890 0.35790269 2.49711886
H -0.72201365 1.34297446 3.31353175

h1a5-4.xyz
62

-1756.52756638002
C 2.29977362 0.67492310 -2.17729078
C 2.39030047 1.09387428 0.35831432
C 1.59463299 0.49180036 -0.81116874
F 2.41141552 1.98352218 -2.52023467
F 1.56550920 0.06416933 -3.13860402
F 3.54092642 0.13249091 -2.20706079
F 2.69010933 2.40012546 0.16056158
F 3.55076515 0.43841348 0.60128773
F 1.64931096 1.02304597 1.50086055
H 0.58298339 -1.19824040 -0.98701393
H 0.65037506 1.06750869 -0.86599912
O 1.40944879 -0.86460083 -0.52258223
C 1.85080716 -2.69159885 2.31783718
O 0.63915143 -2.78959548 2.47057764
C 2.70079914 -1.84682850 3.23953982
C 2.56877799 -3.38427799 1.18405002
H 1.97270078 -4.22423300 0.81009649
H 3.57214915 -3.72272619 1.47625552
H 2.68465398 -2.64677943 0.37262887
H 2.07129911 -1.29505571 3.94521824
H 3.31774782 -1.14951158 2.65518723
H 3.39473135 -2.49588043 3.79655470
C -1.54806174 3.22569557 0.04421860
O -1.19046513 2.31725039 -0.69917460
C -0.60655986 3.87352702 1.02847529
C -2.96386442 3.75041936 0.02510592
H -3.51204308 3.35337027 -0.83559184
H -2.97939385 4.84971190 0.01085072
H -3.45531223 3.42778843 0.95537840
H 0.36544428 3.37243711 1.02845743
H -1.04841337 3.83867493 2.03463653
H -0.47218167 4.93495713 0.76804903
C -1.63434505 0.56631534 2.51945780
O -2.36681555 1.54922975 2.43481097
C -0.89893602 0.23988014 3.79674002
C -1.42453573 -0.37247853 1.36244807
H -2.14928488 -1.19713648 1.45586131
H -0.42682766 -0.82464505 1.38101272
H -1.59898422 0.14060424 0.41154592
H -1.34068935 0.77078275 4.64718940
H 0.14670603 0.56539850 3.67407933
H -0.86382586 -0.84494494 3.96832093
C -4.15563146 -0.49881905 -0.73020391
O -4.03662275 -1.71042806 -0.57654421
C -4.83261039 0.35915311 0.31238707
C -3.59748050 0.21415047 -1.93678150
H -3.12224022 -0.49206939 -2.62432813
H -2.85238236 0.96290090 -1.61724654
H -4.39784735 0.76471616 -2.45473551
H -5.39442722 -0.26728791 1.01390183
H -5.49221135 1.10847868 -0.14771548
H -4.05752524 0.91064220 0.87330687
C -1.32370125 -2.84164376 -1.65696514
O -0.67967796 -1.81151823 -1.86571103
C -2.23148696 -3.39477472 -2.72279079
C -1.27239259 -3.58113190 -0.35079192
H -0.54994724 -3.15149897 0.35127015
H -1.04149636 -4.64291834 -0.52359908
H -2.27835197 -3.54644598 0.09588463
H -2.08258772 -2.86278240 -3.66832242
H -3.26837548 -3.26013221 -2.37847787
H -2.06508141 -4.47280361 -2.86017918

h1a5-7.xyz
62
-1756.53121457694
C 2.09791948 -0.87763389 -1.79071256
C 2.90281164 -0.85240566 0.65465267
C 1.72217820 -0.64966681 -0.30959198
F 2.47350634 -2.15458465 -2.03775368
F 1.00768842 -0.61961755 -2.56396549
F 3.09206874 -0.06093373 -2.21711699
F 3.49443235 -2.05981424 0.49548154
F 3.85746541 0.09887294 0.52250111
F 2.45497838 -0.79000797 1.94014474
H 0.25052721 0.68649921 -0.26738505
H 0.97031769 -1.42077447 -0.05504705
O 1.25055659 0.65646321 -0.12466483
C 0.96302820 3.70988271 0.74702475
O -0.25629872 3.82565439 0.69825558
C 1.67645050 3.34096997 2.02645745
C 1.83012971 3.88109564 -0.47739909
H 1.26147988 4.34252215 -1.29145821
H 2.72900528 4.47288850 -0.25512786
H 2.16703046 2.87914945 -0.78808311
H 0.96923311 3.29668804 2.86083208
H 2.15569751 2.35924538 1.89198712
H 2.47687876 4.06203029 2.24814790
C -1.21294077 -3.14298127 1.32037743
O -0.70307106 -2.63348882 0.32852266
C -0.44743316 -3.31636476 2.60938942
C -2.64030657 -3.63097077 1.31214364
H -2.99474829 -3.76566620 0.28479156
H -2.75514747 -4.56213671 1.88402954
H -3.24901090 -2.85812138 1.80667653
H 0.52205593 -2.80995323 2.55872842
H -1.04035857 -2.92959447 3.45030030
H -0.28664534 -4.39062351 2.79333505
C -1.94671915 0.44763543 2.70120317
O -2.57892660 -0.57985787 2.48384052
C -0.46903025 0.42182132 3.00452087
C -2.59524838 1.81027129 2.68704886
H -3.61478173 1.74684319 2.29278502
H -2.63235556 2.19721187 3.71865404
H -1.98977361 2.51845289 2.10167809
H -0.20544044 1.14778069 3.78599625
H -0.14620183 -0.58237544 3.29363678
H 0.07724409 0.71554389 2.09497368
C -1.70531304 1.92281056 -1.09365377
O -1.32767831 1.04733005 -0.30713700
C -2.98620838 2.66594116 -0.85952321
C -0.92583296 2.27583933 -2.32585705
H 0.10227554 1.90557060 -2.27253338
H -1.43857367 1.78888614 -3.17177184
H -0.94419731 3.35864722 -2.50485342
H -2.71877844 3.67023107 -0.49372394
H -3.54531896 2.77635517 -1.79700413
H -3.59752151 2.15962767 -0.10723458
C -3.29701087 -0.78983658 -2.17557116
O -3.36835306 0.20607686 -2.88770312
C -4.06977003 -0.89016174 -0.87993526
C -2.41276773 -1.95970864 -2.52583480
H -1.81760366 -1.73755025 -3.41755431
H -1.75924250 -2.20731692 -1.67387175
H -3.03685845 -2.84841941 -2.71323550
H -4.84584893 -0.11820153 -0.83731270
H -4.51837003 -1.88568311 -0.75548244

```
H  -3.37147491 -0.74585083 -0.03915067
```

h2a1-1.xyz

```
34
-1773.71099044827
C   2.73365166  0.14347233 -1.25901914
C   2.62765962  1.29440682  1.06428379
C   1.86235611  0.88548354 -0.21742684
F   3.78333293  0.88554006 -1.67283616
F   1.97521888 -0.14006714 -2.34910676
F   3.21106812 -1.03446709 -0.78454065
F   3.70435039  2.06678628  0.78210141
F   3.05427081  0.21882986  1.76566695
F   1.80563947  2.01004971  1.86418858
H   0.92119401 -0.75249290  0.52922790
H   1.54441467  1.82333341 -0.69820643
O   0.71672427  0.15102431  0.10260149
C  -2.71030113  1.79327092 -0.10288654
C  -3.07560987 -0.76628679 -0.01594488
C  -2.67210559  0.45344233 -0.87919701
F  -3.96364868  2.09843884  0.31347323
F  -2.29623637  2.79360567 -0.91724379
F  -1.89913779  1.78630572  0.98124412
F  -4.25135183 -0.59471296  0.62774670
F  -2.13180201 -1.06989685  0.91066057
F  -3.20901949 -1.85730530 -0.82013591
H  -0.70382538  0.22223242 -0.83709193
H  -3.44204127  0.53839055 -1.66207339
O  -1.44582606  0.23872445 -1.50465184
C   0.58517080 -3.19406213  0.53092630
O   0.96554105 -2.21026732  1.16832911
C   0.12855884 -3.10690742 -0.89917022
C   0.55425849 -4.54881462  1.18567589
H   0.73920190 -4.46127643  2.26095293
H   1.33059772 -5.18474764  0.73137240
H  -0.40844422 -5.04645515  0.99859573
H   0.41211646 -2.15753831 -1.36275131
H  -0.96985393 -3.18597747 -0.91842257
H   0.51882415 -3.95049783 -1.48520488
```

h2a1-6.xyz

```
34
-1773.71049113462
C  -2.89932544  1.08062516  0.19762215
C  -2.87775645 -1.46918090 -0.22153510
C  -2.79409556 -0.05648876 -0.84943930
F  -4.10763471  1.09848991  0.80893867
F  -2.74072946  2.27913603 -0.42094085
F  -1.94814128  0.99603214  1.16121962
F  -3.95555431 -1.62019433  0.58304363
F  -1.77045728 -1.76176638  0.51547736
F  -2.96266991 -2.39501440 -1.20319527
H  -0.83422141 -0.01204424 -1.13403702
H  -3.68752847  0.04388897 -1.48599083
O  -1.67360709  0.07294559 -1.66382512
C   2.09628982 -0.58828958  1.48623424
C   2.45903244 -1.57733077 -0.88545538
C   1.45382373 -1.00675182  0.14314540
F   2.76216065 -1.60456997  2.07647069
F   1.12301089 -0.18257403  2.33874541
F   2.95894501  0.44968616  1.33770729
F   3.10005984 -2.67103261 -0.40915080
F   3.39292013 -0.66706195 -1.24751340
F   1.78884055 -1.94625825 -2.00166586
H   1.29117229  0.84785030 -0.66725681
H   0.75583096 -1.82386036  0.37776428
O   0.71669901  0.05129482 -0.39738960
C   1.62707240  3.27744029 -0.54617898
O   1.93878010  2.23609819 -1.12705360
C   0.64554382  3.30223115  0.59342980
C   2.22622345  4.58933776 -0.97595820
H   2.76492604  4.47623015 -1.92211095
H   2.92674797  4.93150933 -0.19734076
H   1.44986285  5.36329798 -1.06165865
H   0.47240662  2.30174473  1.00113058
H  -0.31378396  3.69120988  0.21551124
H   0.97812086  3.98955902  1.38340631
```

h2a1-10.xyz

```
34
-1773.71140678702
C  -2.76739596  0.34519661  1.19767892
C  -2.61153762  1.08415275 -1.28486467
C  -1.87357527  0.89885656  0.06304027
F  -3.82983068  1.13882836  1.45206439
F  -2.03485929  0.25995897  2.33702686
F  -3.22958173 -0.90170974  0.92260128
F  -3.69225907  1.89121520 -1.16046759
F  -3.02513050 -0.09619035 -1.80193791
F  -1.77165296  1.65469245 -2.17745759
H  -0.92574059 -0.84134713 -0.39299142
H  -1.55971766  1.90413875  0.38304025
O  -0.72818197  0.11728012 -0.10502188
C   2.75496082  1.76075844  0.41401865
C   2.94510796 -0.69114359 -0.40166906
C   2.67546132  0.26112970  0.78936129
F   3.91513317  2.07759425 -0.20765879
F   2.67692983  2.50649360  1.54274060
F   1.73207983  2.13525340 -0.39241481
F   4.19338906 -0.54494256 -0.90191278
F   2.06292082 -0.52642905 -1.41185041
F   2.83217071 -1.98006469  0.02808840
H   0.69455289  0.14123720  0.82238867
H   3.49157906  0.08305598  1.50675257
O   1.47046398 -0.03536391  1.42723358
C  -0.44104481 -3.23484875 -0.03441405
O  -0.83038338 -2.37197811 -0.82532545
C  -0.09435218 -4.61017536 -0.52878905
C  -0.28287589 -2.94957994  1.43543841
H  -1.17767063 -2.43971642  1.81514546
H   0.56108675 -2.25360215  1.57212576
H  -0.09348559 -3.85757741  2.01899148
H  -0.20898230 -4.67540433 -1.61527933
H  -0.73752599 -5.35408893 -0.03317222
H   0.94017837 -4.85204788 -0.23942947
```

h2a2-1.xyz

```
44
-1966.97358922067
C  -1.90867066 -1.59004335 -1.19778501
C  -2.63340122 -1.20625120  1.24420464
```

C	-2.08116336	-0.56819237	-0.05624004
F	-1.15988100	-2.65050708	-0.81913125
F	-1.26211234	-0.99447860	-2.24384239
F	-3.08406564	-2.05095834	-1.66794380
F	-1.76375582	-2.09090911	1.78926006
F	-3.81365204	-1.83315767	1.06498340
F	-2.83077886	-0.21860051	2.16324917
H	-2.70937006	1.29285601	-0.05418583
H	-1.06569769	-0.20563451	0.18325538
O	-2.93187591	0.43824108	-0.51644496
C	3.13472164	-0.97958861	-0.66763765
C	2.43651227	-0.20876260	1.69627793
C	3.21596978	0.13439118	0.40380256
F	3.57478776	-2.17307208	-0.21081618
F	3.90897050	-0.63674082	-1.72862509
F	1.86990674	-1.14921875	-1.13141798
F	2.88111472	-1.34085119	2.28466673
F	1.10356299	-0.35453357	1.46705841
F	2.58077450	0.80109226	2.58969754
H	1.89803258	1.37627017	-0.44178591
H	4.27555674	0.19101099	0.70012276
O	2.84581892	1.37437830	-0.10944624
C	0.11381235	1.83407204	-2.24071718
O	0.37666657	1.79448970	-1.03411005
C	-1.22646698	2.31593036	-2.71577048
C	1.09958976	1.37831038	-3.28018606
H	2.04803446	1.06847199	-2.83201396
H	1.27272889	2.18605793	-4.00688596
H	0.66355211	0.53866626	-3.84322983
H	-1.66922775	3.01090983	-1.99397500
H	-1.89488258	1.44075062	-2.76333554
H	-1.17220275	2.75730468	-3.71856400
C	-1.28405641	3.30763975	0.99042721
O	-2.27050371	2.89756496	0.38050751
C	-0.59908866	2.50741370	2.06149435
C	-0.70103432	4.66195403	0.67617256
H	-1.37449593	5.23188137	0.02762659
H	0.26003543	4.50724466	0.16048609
H	-0.48170676	5.22654717	1.59327433
H	-1.04409597	1.51441058	2.16610042
H	-0.67821809	3.04195909	3.02120344
H	0.47331100	2.42112712	1.83763316

h2a2-4.xyz

44

-1966.97360916416

C	1.92411436	1.57561301	-1.21302677
C	2.57289300	1.23257182	1.25531493
C	2.06640903	0.57098372	-0.05229783
F	1.17193323	2.64545623	-0.86845797
F	1.29843993	0.96663163	-2.26429031
F	3.11214057	2.02358306	-1.66314637
F	1.67426078	2.10856731	1.76614698
F	3.74718230	1.87734154	1.10118793
F	2.76119898	0.25898120	2.19104867
H	2.70677720	-1.28652556	-0.00818344
H	1.04652097	0.20483629	0.16072542
O	2.93746170	-0.43536855	-0.47248780
C	-3.09983818	0.96767306	-0.70263785
C	-2.44314860	0.23088483	1.68377146
C	-3.20326916	-0.12934690	0.38414581
F	-3.54295193	2.16945350	-0.27162775
F	-3.85710828	0.61147591	-1.77171110
F	-1.82684740	1.12549627	-1.14760719
F	-2.89460350	1.37272914	2.24782154
F	-1.10662174	0.37045348	1.47382562
F	-2.60425015	-0.76510678	2.58965953
H	-1.88158663	-1.38569776	-0.43387024
H	-4.26762511	-0.17727603	0.66440914
O	-2.83014723	-1.37861461	-0.10408869
C	-0.09154006	-1.86747264	-2.22349677
O	-0.35789785	-1.80530437	-1.01863045
C	1.25313645	-2.34998202	-2.68541816
C	-1.07814883	-1.44151851	-3.27467533
H	-2.02293851	-1.10976378	-2.83466361
H	-1.26015694	-2.27532301	-3.96941666
H	-0.63881844	-0.62756847	-3.87165414
H	1.70209205	-3.02140618	-1.94539865
H	1.91341044	-1.47058344	-2.75877108
H	1.20178389	-2.81898691	-3.67586776
C	1.28033413	-3.30175024	1.03135138
O	2.27257769	-2.89236869	0.43041814
C	0.57550814	-2.49378061	2.08340983
C	0.71091509	-4.66385293	0.72512925
H	1.39722339	-5.23652708	0.09266580
H	-0.24406805	-4.52230085	0.19456805
H	0.48292667	-5.21923364	1.64576912
H	1.00905216	-1.49483594	2.17907756
H	0.65019233	-3.01408954	3.05129959
H	-0.49530827	-2.42177358	1.84724251

h2a2-6.xyz

44

-1966.97356729875

C	3.03413437	-1.03226182	0.68052670
C	2.48491073	-0.10650795	-1.66736643
C	3.20960701	0.13470358	-0.32094878
F	3.42121301	-2.22170562	0.16873186
F	3.79125864	-0.79392155	1.78163967
F	1.74766934	-1.15470085	1.09707169
F	2.94219244	-1.20310893	-2.31103370
F	1.14117486	-0.24688109	-1.50949023
F	2.68339484	0.95869176	-2.48239730
H	1.89614279	1.37693164	0.53048018
H	4.28397845	0.16690921	-0.56241263
O	2.85918733	1.35695255	0.24600079
C	-1.90212469	-1.61895257	1.15091553
C	-2.53399259	-1.21285320	-1.31217408
C	-2.04837885	-0.58271866	0.01849678
F	-1.12729397	-2.66473029	0.78473797
F	-1.30004706	-1.03055767	2.22756734
F	-3.08729170	-2.10033476	1.57293159
F	-1.62044429	-2.06485832	-1.83689702
F	-3.70286133	-1.87353719	-1.18868540
F	-2.72298659	-0.21591367	-2.22311637
H	-2.71416941	1.26660878	0.02490469
H	-1.03178711	-0.19641965	-0.17418522
O	-2.93770540	0.39824816	0.45987676
C	0.06696596	1.79721366	2.28254053
O	0.35679242	1.79709884	1.08129651
C	1.03055326	1.31052576	3.32895358
C	-1.28419083	2.26141375	2.74407123
H	-1.72682913	2.95652460	2.02273260
H	-1.94269265	1.37841452	2.78221922
H	-1.24459129	2.69619820	3.75069764
H	1.98458897	1.00290552	2.89115793

H 1.19773536 2.10216814 4.07473466
H 0.57809419 0.46364462 3.86732325
C -1.30734439 3.33493025 -0.93598992
O -2.29556109 2.89051530 -0.35359213
C -0.59420457 2.57829734 -2.02007568
C -0.75193713 4.68822950 -0.57197047
H -1.44878387 5.23020021 0.07583710
H 0.19841187 4.53228654 -0.03706310
H -0.51875488 5.28098134 -1.46755202
H -1.01770222 1.58001120 -2.15780511
H -0.67326294 3.13792531 -2.96545126
H 0.47696837 2.50677635 -1.78567046

h2a2-7.xyz
44
-1966.97359323817
C 1.93808182 1.50638757 1.28797594
C 2.72770995 1.23239852 -1.14913295
C 2.12753568 0.54001994 0.10150378
F 1.22053592 2.59924625 0.94153850
F 1.24857060 0.87241087 2.28240677
F 3.10661111 1.91982031 1.81594561
F 1.88755254 2.15707829 -1.67386169
F 3.91152363 1.82979890 -0.90566608
F 2.93664808 0.28831924 -2.11070954
H 2.71500169 -1.33212393 0.03008041
H 1.11382644 0.20824592 -0.18454201
O 2.94767185 -0.50360212 0.53327564
C -3.25942795 0.89076522 0.67537666
C -2.38254898 0.38609400 -1.70174995
C -3.21932618 -0.11537860 -0.50003072
F -3.75805999 2.09310838 0.31141392
F -4.05553815 0.39844752 1.65875186
F -2.03083498 1.09349506 1.21674438
F -2.82291596 1.56767791 -2.18660673
F -1.06840664 0.53016516 -1.38318788
F -2.45421644 -0.52131609 -2.70725245
H -1.89296609 -1.37376475 0.30793161
H -4.25527815 -0.19062080 -0.86717561
O -2.81647711 -1.38210717 -0.08598927
C -0.15429004 -1.92457381 2.14702701
O -0.38935456 -1.80182207 0.94054037
C 1.17299170 -2.44552714 2.61778739
C -1.16163774 -1.53771278 3.19336834
H -2.08832661 -1.16517698 2.74805015
H -1.37608791 -2.40487622 3.83619737
H -0.72474314 -0.76602821 3.84571548
H 1.61609380 -3.11262706 1.87015386
H 1.85256059 -1.58297003 2.71270032
H 1.09950428 -2.93206724 3.59812180
C 1.25192145 -3.24332511 -1.13483414
O 2.24276501 -2.89937045 -0.49193146
C 0.60536459 -2.36304558 -2.16639157
C 0.62381082 -4.59442290 -0.90499103
H 1.26030927 -5.21236423 -0.26324610
H -0.35168094 -4.44043571 -0.41716092
H 0.42440562 -5.10853389 -1.85590918
H 1.09200978 -1.38551631 -2.22075402
H 0.66453880 -2.85127851 -3.15172732
H -0.46322414 -2.24329856 -1.93935570

h2a2-10.xyz
44
-1966.97361536081
C 1.88602930 1.55332666 -1.24879170
C 2.66452154 1.25177098 1.18875062
C 2.09596927 0.56819150 -0.08149100
F 1.13569795 2.61770348 -0.88427625
F 1.22061215 0.92062470 -2.26053067
F 3.04514542 2.01054333 -1.76100312
F 1.78723125 2.13028634 1.73182234
F 3.82551125 1.90012613 0.96606655
F 2.90597223 0.29317573 2.12786875
H 2.74182131 -1.28461645 -0.04212275
H 1.09054584 0.19937354 0.18862873
O 2.95086597 -0.44035881 -0.52891747
C -3.18881493 0.90440633 -0.68618148
C -2.42689312 0.28767919 1.70406669
C -3.21419998 -0.15096152 0.44554139
F -3.68464469 2.09868276 -0.29291331
F -3.94924187 0.46731601 -1.72263218
F -1.93531505 1.10841423 -1.16584969
F -2.88311588 1.44833483 2.22417838
F -1.09947069 0.43839997 1.45010074
F -2.54573846 -0.66507508 2.66190976
H -1.87168301 -1.39231056 -0.36369135
H -4.26643775 -0.22700077 0.76283694
O -2.80914767 -1.40479958 -0.00429264
C -0.10656030 -1.89879082 -2.18130730
O -0.35356279 -1.80829764 -0.97419483
C 1.23086146 -2.39125961 -2.65375398
C -1.11003065 -1.49935455 -3.22677486
H -2.04557432 -1.15046280 -2.78059774
H -1.30771440 -2.35316534 -3.89249459
H -0.67831148 -0.70607926 -3.85621268
H 1.68540386 -3.05756022 -1.91248152
H 1.89404747 -1.51519816 -2.74056992
H 1.16728888 -2.86896703 -3.63922365
C 1.32163361 -3.25494632 1.08052133
O 2.30577966 -2.87275087 0.44901497
C 0.64941135 -2.41271034 2.12742679
C 0.72817300 -4.61583999 0.81865811
H 1.38494850 -5.20544175 0.17063456
H -0.24587457 -4.47490984 0.32401279
H 0.53217131 -5.15356919 1.75703772
H 1.11652451 -1.42774993 2.20985469
H 0.70748238 -2.92378401 3.10105735
H -0.41888529 -2.30695584 1.89167632

h2a3-1.xyz
54
-2160.24174136511
C -3.54464119 1.43115493 0.57318611
C -3.72063597 -0.53589813 -1.08638904
C -3.08262371 0.00365770 0.21489709
F -3.29795792 2.31185863 -0.44183779
F -2.85183341 1.87384537 1.65391287
F -4.85532460 1.50602693 0.86958599
F -3.34040145 0.18847867 -2.17594942
F -5.06699718 -0.55923544 -1.05461729
F -3.29322276 -1.81193233 -1.29289236
H -2.53776227 -1.36138598 1.48198440
H -2.00010022 0.08329691 0.01088024
O -3.37331916 -0.84319963 1.28366447
C 3.66053216 0.25615112 -1.42908398

C 3.47351996 0.88699161 1.05898528
C 3.04918181 -0.09542118 -0.04999673
F 3.15348715 1.42316030 -1.92051226
F 3.33677833 -0.71957181 -2.32004352
F 5.00295976 0.36387731 -1.41370932
F 3.23306975 2.17968679 0.70860034
F 4.77520185 0.78327229 1.38458290
F 2.74972074 0.63980702 2.18522810
H 2.72104623 -2.02578586 0.03274196
H 1.95877586 0.03678640 -0.16393459
O 3.41574414 -1.37904931 0.35072685
C -0.22603647 -1.60637410 2.47168120
O -1.06411511 -2.11015858 1.71401851
C -0.41367436 -0.22314718 3.02791334
C 1.02550887 -2.34855509 2.83097590
H 1.07152445 -3.31464848 2.32074169
H 1.06295392 -2.48594599 3.92312906
H 1.90753567 -1.74624790 2.56309907
H -1.47663607 -0.01133535 3.19445931
H -0.05462260 0.48378650 2.25997653
H 0.16861154 -0.05815164 3.94172522
C -0.03486982 2.16708038 -0.63740569
O 0.01718838 1.09646329 -0.03207288
C 0.07961526 3.48054212 0.08719148
C -0.22315045 2.21460370 -2.13078968
H -0.04397661 1.23166094 -2.57763927
H 0.43004199 2.96925505 -2.58976585
H -1.26230614 2.51540180 -2.33854577
H 0.00200341 3.33370866 1.16875817
H -0.68683072 4.18684811 -0.26183378
H 1.05915491 3.92640241 -0.14758180
C 0.44843742 -3.09162655 -0.86789111
O 1.51332464 -3.16496999 -0.25173885
C 0.11397903 -1.88686149 -1.70964884
C -0.56139700 -4.19830382 -0.79526611
H -0.20828993 -5.00810505 -0.14940668
H -0.77092738 -4.57852288 -1.80723358
H -1.50672502 -3.79100730 -0.40705218
H -0.78259626 -2.03380949 -2.32106366
H 0.97248491 -1.63722845 -2.34705859
H -0.05425379 -1.02439080 -1.04393105

h2a4-1.xyz
64
-2353.50029652775
C -3.36567316 1.43604975 -0.84621188
C -3.50535239 0.14473023 1.37025889
C -2.93346928 0.17326591 -0.06578473
F -3.00938182 2.58816085 -0.21415220
F -2.75765508 1.44659020 -2.06325768
F -4.69608075 1.48654776 -1.06275417
F -3.07448389 1.19533229 2.11697138
F -4.85425049 0.14213352 1.40257195
F -3.08708160 -0.98824627 2.00360494
H -2.60289294 -1.53059443 -1.01018562
H -1.83503315 0.22366931 0.03698074
O -3.38241797 -0.95178783 -0.76205689
C 3.55543464 -0.20680877 1.46823218
C 3.48221819 1.19911085 -0.68188357
C 2.94958468 -0.05392482 0.05901468
F 3.27134137 0.86849301 2.25454169
F 3.02841227 -1.29764992 2.08394052
F 4.89499764 -0.36134208 1.45549231
F 3.19860798 2.36019641 -0.02547089
F 4.81201909 1.16728145 -0.89500615
F 2.87801741 1.28049004 -1.90125294
H 2.59512530 -1.35705052 -1.38448010
H 1.86444646 0.10690202 0.20313236
O 3.26117881 -1.20963076 -0.65278336
C 0.15227231 2.92243187 -0.15528434
O 0.04124369 1.71502378 0.02552027
C 0.38258306 3.87356517 0.99372197
C 0.06831122 3.52795108 -1.53478751
H -0.38270249 2.82182932 -2.23806285
H -0.49671964 4.47024520 -1.52356071
H 1.09086374 3.76587257 -1.86899903
H 0.68283051 3.32745535 1.89356378
H 1.13739760 4.62970993 0.73641626
H -0.55772820 4.41159619 1.19522093
C 0.67920760 -1.45097068 -3.24888589
O 1.68076516 -1.90074780 -2.69210326
C 0.04637094 -2.19590218 -4.39446097
C 0.05514299 -0.14347215 -2.85686192
H 0.26642684 0.11604982 -1.81448030
H 0.48709258 0.64201432 -3.49922168
H -1.02719705 -0.15114883 -3.02926866
H 0.67697676 -3.03370472 -4.70996318
H -0.93177345 -2.57096426 -4.05709695
H -0.14457267 -1.51908649 -5.24039269
C -1.01511538 -3.42580390 -0.59953916
O -1.32628553 -2.53678609 -1.39732662
C -1.96856752 -3.89196296 0.46681939
C 0.31944048 -4.10730264 -0.68374164
H 0.77215417 -3.98140034 -1.67192609
H 0.97477199 -3.61159764 0.05048291
H 0.24959040 -5.16811474 -0.40780611
H -2.75780388 -3.15527176 0.64628988
H -2.43070391 -4.83079255 0.11794004
H -1.43554077 -4.12279870 1.39722674
C 0.02108123 -1.26172520 2.39778288
O -0.03140085 -1.44066995 1.18658048
C 0.00769890 0.11939783 3.00005033
C 0.08477779 -2.42405203 3.36449910
H 0.35037575 -3.34873659 2.84102342
H 0.80437518 -2.23031801 4.17143249
H -0.90350439 -2.54642396 3.83588926
H -0.08131877 0.87266681 2.21040294
H -0.82546897 0.20948161 3.71306526
H 0.93499212 0.28002412 3.56990296

h2a4-2.xyz
64
-2353.50149407992
C -3.74730812 -0.92710793 0.78639320
C -3.47975529 0.25712745 -1.48728605
C -3.02553204 0.17972146 -0.01448982
F -3.63377095 -2.15486316 0.21071344
F -3.18591922 -1.01336844 2.02492435
F -5.06149957 -0.67802544 0.95647853
F -3.21404397 -0.88666104 -2.16968173
F -4.79528636 0.52447378 -1.62473720
F -2.79698788 1.25901319 -2.11492766
H -2.39143400 1.76717280 0.94813693
H -1.95969618 -0.10572517 -0.03783820
O -3.26489052 1.40794548 0.60568744

C 3.85391977 0.67810218 -0.71893385
C 3.22266470 -1.75752775 -0.17266932
C 3.06597288 -0.27287336 0.21587017
F 3.46487043 0.57115722 -2.02186556
F 3.62668418 1.96283310 -0.33954457
F 5.18631489 0.47417722 -0.67698503
F 2.75735776 -2.01079702 -1.42608733
F 4.50002610 -2.18808253 -0.11699108
F 2.50016952 -2.53277917 0.68269863
H 2.87743115 0.44647410 2.04586666
H 1.99820619 -0.02180834 0.07309987
O 3.51972431 -0.12226707 1.52616830
C -0.50225517 -3.03434820 0.05579430
O -0.50191211 -1.81372186 -0.05015697
C -0.19825328 -3.93003740 -1.12060822
C -0.80363894 -3.71674499 1.36844775
H -1.29066747 -3.02375000 2.06085551
H -1.42758182 -4.60890985 1.22014326
H 0.14785596 -4.05798269 1.80717484
H 0.23096192 -3.35417180 -1.94598540
H 0.48099133 -4.74421629 -0.83106856
H -1.13758034 -4.39874588 -1.45566340
C 0.78423809 0.77036455 3.40480074
O 1.83413485 1.30088317 3.03301638
C 0.38821768 -0.59988139 2.93475031
C -0.14384010 1.47789339 4.35058198
H 0.16729386 2.51671056 4.49907557
H -1.17208812 1.43711495 3.96637119
H -0.13734866 0.94910111 5.31761583
H -0.15262605 -0.49747196 1.97924270
H 1.27553311 -1.21430702 2.74117903
H -0.28512125 -1.09897805 3.64237041
C 0.37722864 1.21582402 -2.33263267
O 0.48825275 1.41204241 -1.12740292
C 0.40502911 2.35867790 -3.32208749
C 0.21073388 -0.16983549 -2.89800232
H -0.04337001 -0.87358073 -2.09781500
H -0.55046698 -0.18326574 -3.69064736
H 1.16474690 -0.47232640 -3.35870344
H -0.62183033 2.54465996 -3.67463013
H 0.79558038 3.26825157 -2.85334428
H 1.00540974 2.10089058 -4.20574019
C -0.45274039 3.28949647 0.77901403
O -0.92912007 2.35291308 1.42724235
C 0.93439364 3.79081801 1.04555277
C -1.25525250 4.00148349 -0.27801831
H -2.07471062 3.37500293 -0.64539253
H -1.68689355 4.90814890 0.17807391
H -0.61437552 4.32872226 -1.10550291
H 1.34397366 3.36494152 1.96664794
H 1.56451771 3.47222985 0.20116698
H 0.95390436 4.89006357 1.07087739

h2a4-9.xyz
64
-2353.49799000591
C -3.21874076 -1.10945898 0.66745173
C -3.40911368 0.51377930 -1.30935307
C -2.64642652 0.15091375 -0.01031377
F -3.28568346 -2.16077467 -0.19216804
F -2.42242428 -1.48349774 1.70042261
F -4.45787777 -0.91431397 1.17035811
F -3.20407941 -0.40318619 -2.29197634
F -4.74363751 0.63320706 -1.13195303
F -2.95892957 1.70791316 -1.77941645
H -1.99736569 1.84895245 0.79051562
H -1.61189149 -0.08541299 -0.31815099
O -2.73586079 1.19291211 0.92114656
C 3.12355967 1.03709952 -0.07965153
C 3.43171127 -1.48404358 -0.45439205
C 2.67676234 -0.38547184 0.32634020
F 3.11397821 1.24253633 -1.42303067
F 2.26337247 1.94521530 0.46854533
F 4.35902828 1.34495718 0.36729225
F 3.10294530 -1.47003658 -1.77789139
F 4.77709882 -1.36521087 -0.37013020
F 3.10275129 -2.70707504 0.02431570
H 2.02388440 -0.58413385 2.17265440
H 1.61485464 -0.48960879 0.04366593
O 2.90301018 -0.55159329 1.69469284
C 0.34826181 0.25614494 -2.92292804
O 0.05100112 0.75536037 -1.84231471
C 1.09086503 1.04256996 -3.97758859
C -0.01726812 -1.16605328 -3.26096325
H -0.18213575 -1.73348984 -2.33766436
H -0.95322622 -1.14825490 -3.84281505
H 0.74977828 -1.64102148 -3.88733664
H 0.61950792 0.91548734 -4.96320463
H 1.14423296 2.10345859 -3.71196232
H 2.11322075 0.64172366 -4.05846014
C -0.14793112 -3.29020163 0.31245554
O -0.00341394 -2.15980305 -0.13886773
C 0.23464675 -3.63177653 1.73010907
C -0.71086529 -4.40907743 -0.53367375
H -0.78715580 -4.10605873 -1.58302838
H -0.09449797 -5.31536052 -0.43944441
H -1.71430240 -4.66549251 -0.15930077
H 0.20350118 -2.73426637 2.35681273
H -0.40161431 -4.42625760 2.14288785
H 1.26991931 -4.01006152 1.71964611
C 0.10262025 0.45828410 3.50313366
O 0.56587174 -0.56890184 3.00106849
C 0.89222995 1.73401551 3.57695048
C -1.30639608 0.48234678 4.02354172
H -1.63497791 -0.52649352 4.29445605
H -1.42337395 1.17680898 4.86521082
H -1.95318442 0.83746288 3.20196221
H 0.96077545 2.06755540 4.62335627
H 1.89359731 1.60826122 3.15317140
H 0.34299335 2.51129535 3.02171467
C -0.23921007 3.84253701 0.31001232
O -0.81462557 3.07809744 1.08727240
C 0.81241082 4.79672628 0.80817397
C -0.54237074 3.84051348 -1.15916363
H -0.14930482 2.89305606 -1.57123750
H -1.62776021 3.82302165 -1.31797854
H -0.09112324 4.68967634 -1.68530247
H 0.99400064 4.66496272 1.87946539
H 1.74546408 4.64122559 0.24659818
H 0.49113458 5.83071157 0.60691293

h3a1-1.xyz
46
-2563.93917595984
C -2.98996451 -0.72222674 -1.45235108
C -4.07221633 0.49091010 0.56430307

C -3.75882350 -0.86392380 -0.11623092
F -3.61325502 0.10670546 -2.32175129
F -2.88957027 -1.93607385 -2.04080137
F -1.73147396 -0.25515180 -1.25785767
F -4.83199267 1.29727567 -0.20862505
F -2.94365833 1.17260162 0.89711144
F -4.75600458 0.26634593 1.71555486
H -2.18831654 -1.40078325 0.95270371
H -4.73446436 -1.30779036 -0.36737824
O -3.10346278 -1.73479733 0.75646191
C 2.97066528 2.81118566 -0.40381134
C 0.39878027 3.07760069 -0.35797065
C 1.64455156 2.70718651 -1.19897365
F 3.23262681 4.08431077 -0.02038239
F 3.99758437 2.39889537 -1.18126035
F 2.97108194 2.03575977 0.71329835
F 0.52686539 4.27159907 0.26941678
F 0.14061101 2.13972055 0.59720098
F -0.68608975 3.14655631 -1.15747870
H 1.41134637 0.74605445 -1.13299831
H 1.71277763 3.46866985 -1.99173276
O 1.49094926 1.46693849 -1.81252541
C 1.25529984 -2.79423604 -1.24559288
C 2.80870382 -2.43908257 0.80526816
C 2.14715720 -1.81413443 -0.44542089
F 1.93508690 -3.90054316 -1.62658659
F 0.81038749 -2.17628204 -2.36150001
F 0.17492987 -3.18823040 -0.53029771
F 3.58190492 -3.50219883 0.49984733
F 1.88850891 -2.83748206 1.72252162
F 3.59709073 -1.51288847 1.40367235
H 0.65531842 -0.84827043 0.50203846
H 2.96898469 -1.52170076 -1.11611055
O 1.43952984 -0.65568667 -0.10080691
C -0.49722799 -0.38282501 2.69590217
O -0.60722438 -0.88434154 1.56832219
C -1.67290236 -0.32418978 3.62340545
C 0.80741535 0.17960298 3.17288543
H 1.50418339 0.34640235 2.34622339
H 1.25441536 -0.54424448 3.87408840
H 0.64693252 1.10921455 3.73537049
H -2.47737774 -0.99010071 3.29464563
H -2.05286550 0.71046329 3.62325960
H -1.36940375 -0.55108934 4.65462941

h3a1-4.xyz

46
-2563.93914840401
C 3.14687246 0.54924622 -1.43143175
C 4.12716356 -0.79163903 0.55625151
C 3.89314030 0.60195771 -0.07609532
F 3.74254495 -0.28245074 -2.31770534
F 3.12561301 1.78644044 -1.97777899
F 1.86077122 0.14575913 -1.27876175
F 4.87555767 -1.59801067 -0.22778372
F 2.96106463 -1.43674217 0.82726952
F 4.78479136 -0.64419530 1.73498398
H 2.32745284 1.17569160 0.98214459
H 4.89400211 1.00695515 -0.29075611
O 3.26201108 1.47007735 0.81673501
C -3.31655246 -2.42417231 -0.42347065
C -0.82404088 -3.10510660 -0.41491082
C -2.00352916 -2.53499817 -1.24037265
F -3.80382503 -3.64383857 -0.08954523
F -4.25893299 -1.79484378 -1.16089810
F -3.16069500 -1.71476992 0.72657723
F -1.15296589 -4.23314943 0.26009965
F -0.36775800 -2.20146926 0.49757369
F 0.20556596 -3.40132841 -1.23605602
H -1.46356864 -0.63683034 -1.18072130
H -2.20791981 -3.27472228 -2.03017822
O -1.65378790 -1.33819156 -1.85910853
C -0.95256479 2.91921878 -1.16186639
C -2.61043590 2.56626659 0.80567585
C -1.93893143 1.96236414 -0.45004751
F -1.54327841 4.08426177 -1.51428350
F -0.49708380 2.32770165 -2.28774143
F 0.11721424 3.20735177 -0.38253889
F -3.29474617 3.69585445 0.52769389
F -1.70614445 2.85091796 1.77957364
F -3.48535893 1.66840010 1.31996108
H -0.54119576 0.85080106 0.48549660
H -2.74959474 1.75989334 -1.16603871
O -1.32302791 0.74339657 -0.14106723
C 0.54636155 0.21743602 2.67337094
O 0.70654699 0.74270720 1.56264848
C -0.80542397 -0.24118281 3.12889525
C 1.70770922 0.02439273 3.60066006
H 2.56537427 0.63599752 3.30211837
H 2.00290636 -1.03669090 3.55558613
H 1.41854800 0.23026773 4.64023099
H -1.51205869 -0.31282122 2.29705115
H -1.18643910 0.49299045 3.85774623
H -0.73193740 -1.20228980 3.65549160

h3a1-10.xyz

46
-2563.93918415226
C 3.09776622 0.68029280 1.40570564
C 4.07182978 -0.68088859 -0.57094643
C 3.83383416 0.71957863 0.04462556
F 3.72046979 -0.11580597 2.30579621
F 3.04958039 1.92848657 1.92435046
F 1.82105169 0.24187826 1.27155283
F 4.80956630 -1.48243288 0.22779230
F 2.90683984 -1.32516301 -0.84810195
F 4.74149595 -0.54581956 -1.74451577
H 2.25737495 1.26533727 -1.01206616
H 4.83340213 1.13427706 0.24669221
O 3.19044557 1.57015525 -0.85630467
C -3.14711779 -2.63825472 0.38631949
C -0.60110049 -3.07943455 0.46196283
C -1.85440874 -2.61190597 1.24110654
F -3.49104651 -3.90005967 0.03141195
F -4.17237136 -2.11961542 1.09873467
F -3.03509108 -1.90601659 -0.75473106
F -0.79048268 -4.25932400 -0.17678293
F -0.22286844 -2.16405703 -0.47428770
F 0.43337138 -3.23357218 1.31492445
H -1.47817679 -0.67462759 1.16103758
H -2.01143261 -3.35466621 2.03893455
O -1.64071470 -1.37639159 1.84607486
C -1.09940072 2.86262762 1.21131986
C -2.69809112 2.53160200 -0.80897068
C -2.04464402 1.90567423 0.44539701
F -1.72824204 4.00078621 1.58587985

F	-0.65706041	2.24607347	2.32898978
F	-0.01872782	3.20217850	0.46906415
F	-3.41983378	3.63361703	-0.51516524
F	-1.77659010	2.86969200	-1.74844222
F	-3.53531583	1.62771587	-1.37439159
H	-0.60795761	0.85318702	-0.49846283
H	-2.86791662	1.66334560	1.13402655
O	-1.39308492	0.71043366	0.11656084
C	0.49934999	0.26009772	-2.68605286
O	0.64438257	0.79648552	-1.57851694
C	-0.83596405	-0.25194795	-3.13336012
C	1.66288160	0.10509729	-3.61789027
H	2.49885985	0.74956165	-3.32710728
H	1.99778254	-0.94393405	-3.56724387
H	1.36136831	0.29320420	-4.65721638
H	-1.53914578	-0.33676209	-2.29992772
H	-1.24303231	0.45665039	-3.87335221
H	-0.72803062	-1.21762695	-3.64571385

h3a2-1.xyz
56
-2757.20994085759

C	-4.31560251	1.11861635	1.22908929
C	-4.15650997	0.35660139	-1.24404902
C	-3.63298268	0.18423387	0.20212713
F	-5.66207279	1.00566065	1.21285163
F	-3.89176454	0.78885710	2.47729593
F	-3.99825397	2.41846252	1.02530696
F	-5.47361773	0.07073656	-1.34161701
F	-3.96517830	1.61644032	-1.71373816
F	-3.48822989	-0.48708801	-2.06446119
H	-1.91151634	1.22507200	-0.06524683
H	-3.89306272	-0.84407156	0.49810970
O	-2.24232665	0.31654909	0.26744013
C	4.37678819	1.95905441	0.65443929
C	3.79653138	0.21305982	-1.17296048
C	3.62717559	1.66637601	-0.66795792
F	5.69965108	1.69454803	0.56911519
F	4.24110005	3.27630954	0.95685026
F	3.87323849	1.25301986	1.69678013
F	5.09147866	-0.10339194	-1.39765988
F	3.28629073	-0.69366116	-0.30603991
F	3.13091431	0.07400952	-2.34803787
H	1.79032115	1.43623607	0.08860023
H	4.10182482	2.30587073	-1.42890423
O	2.28213347	2.02273648	-0.57435992
C	0.68023665	-3.52382669	1.23362526
C	0.02889362	-2.94429334	-1.19617768
C	0.10217977	-2.46805255	0.27287301
F	1.89486935	-3.96137246	0.82332878
F	0.84318782	-2.97036431	2.46495897
F	-0.12486652	-4.59783003	1.37549396
F	1.25434480	-3.18099248	-1.72125580
F	-0.72027955	-4.05373702	-1.36081238
F	-0.54287907	-1.95396153	-1.94279444
H	-1.38246958	-1.19518925	0.48585060
H	0.80129289	-1.61490887	0.29333887
O	-1.17206568	-2.12765785	0.74635657
C	-0.61506464	2.67343319	-1.55031923
O	-1.25092936	2.59058627	-0.49311116
C	-0.42553702	1.50171683	-2.46602784
C	0.02344867	3.97487835	-1.93758508
H	-0.42037217	4.80594283	-1.37959749
H	1.09094003	3.89320416	-1.67630807
H	-0.03872665	4.15023235	-3.01981092
H	-0.80665500	0.57449179	-2.02893944
H	-0.94774212	1.69795211	-3.41548383
H	0.64250843	1.39215329	-2.70115551
C	0.34306948	1.11482829	2.09538012
O	0.83299159	0.57856219	1.09499707
C	0.45845478	2.59582367	2.32405004
C	-0.40370881	0.30777356	3.11312361
H	-0.60485748	-0.70432589	2.74943605
H	0.20876142	0.25365135	4.02825007
H	-1.34012367	0.81269712	3.38822391
H	1.29586679	3.02287875	1.76175970
H	-0.47145927	3.05706410	1.95234192
H	0.54270283	2.83383834	3.39215536

h3a2-2.xyz
56
-2757.20996217179

C	4.34089873	1.02538810	1.25136510
C	4.19768008	0.36657839	-1.25221688
C	3.65840069	0.14099718	0.18067402
F	5.68631607	0.89976087	1.24260103
F	3.90223807	0.64907579	2.48092865
F	4.03761258	2.33557132	1.09794952
F	5.51399744	0.07619824	-1.34738183
F	4.01853810	1.64496995	-1.67360288
F	3.53304762	-0.43972698	-2.11262990
H	1.94846664	1.20649103	-0.07032936
H	3.90480975	-0.90115056	0.43713210
O	2.26887711	0.28623555	0.23861271
C	-4.38728834	1.90723051	0.67757354
C	-3.79713316	0.24719852	-1.22411040
C	-3.65004240	1.68303012	-0.66495780
F	-5.70434383	1.61287331	0.59982885
F	-4.27921603	3.21485583	1.02806598
F	-3.85170141	1.17516597	1.68532293
F	-5.08805803	-0.08353828	-1.45133468
F	-3.26373574	-0.68264557	-0.39661774
F	-3.13868431	0.16492842	-2.40880379
H	-1.80285285	1.47179925	0.07222502
H	-4.14734592	2.34197848	-1.39411006
O	-2.31108853	2.06255100	-0.57446434
C	-0.75954312	-3.45616362	1.23221910
C	-0.01706816	-2.94772153	-1.18757833
C	-0.12353459	-2.43747224	0.26787670
F	-1.97407912	-3.86733383	0.79478877
F	-0.94224983	-2.87278207	2.44665789
F	0.00948377	-4.54942348	1.41935845
F	-1.23116043	-3.16586087	-1.74611782
F	0.70858755	-4.07889843	-1.30403926
F	0.60248085	-1.98884331	-1.93706403
H	1.38526455	-1.19843788	0.49905164
H	-0.80015216	-1.56638164	0.24884296
O	1.14435599	-2.11972525	0.77316916
C	0.64203042	2.67746233	-1.52138647
O	1.29546176	2.58413948	-0.47572344
C	0.00331809	3.98397609	-1.89005198
C	0.43071165	1.51272180	-2.44173680
H	-0.64040166	1.41529964	-2.66776939
H	0.80894111	0.57951561	-2.01472497
H	0.94580919	1.70989565	-3.39502640
H	0.44685477	4.80768462	-1.32112957

H -1.06529245 3.89859314 -1.63418778
H 0.06774370 4.17333913 -2.96998821
C -0.34956888 1.16053792 2.07011388
O -0.82748076 0.62085328 1.06576066
C -0.48016454 2.64046464 2.29923419
C 0.39884242 0.36006559 3.09150690
H 0.60281014 -0.65313987 2.73249631
H -0.21346809 0.30918474 4.00689608
H 1.33368992 0.86915110 3.36481860
H -1.32220690 3.05908376 1.73741080
H 0.44512315 3.10996967 1.92603105
H -0.56581120 2.87836001 3.36718867

h3a2-4.xyz
56
-2757.20994996915
C -4.39354452 -0.96943372 1.23770401
C -4.20123899 -0.36814813 -1.27808949
C -3.68227570 -0.11957060 0.15816419
F -5.73448910 -0.80086524 1.22367229
F -3.94594850 -0.59208887 2.46318547
F -4.13213960 -2.29041770 1.10050091
F -5.52001289 -0.09812206 -1.39478035
F -3.99829005 -1.64873084 -1.68211957
F -3.53604081 0.43753811 -2.13853688
H -1.98386563 -1.21833329 -0.03288842
H -3.91636865 0.93152149 0.38841147
O -2.29577318 -0.28332442 0.23757105
C 4.43796173 -1.80681363 0.70123646
C 3.79084284 -0.28947149 -1.29842876
C 3.69202449 -1.69154171 -0.65009871
F 5.74830711 -1.49251022 0.59606609
F 4.35803683 -3.08884432 1.14238083
F 3.89140653 -1.01707022 1.65817690
F 5.06993240 0.07714192 -1.53547031
F 3.21188511 0.66867103 -0.53677002
F 3.14281041 -0.30850103 -2.49198436
H 1.84295166 -1.50213383 0.08887344
H 4.20896039 -2.37845519 -1.33856854
O 2.36542041 -2.10631813 -0.53297749
C 0.77329701 3.42252028 1.23321005
C 0.00643895 2.92758993 -1.18199846
C 0.12044853 2.41248138 0.27100835
F 1.98511874 3.82938490 0.78445585
F 0.96621742 2.83136350 2.44242885
F 0.01262416 4.51905110 1.43475168
F 1.21818949 3.13426164 -1.74992839
F -0.70756711 4.06737653 -1.28863896
F -0.63012424 1.97821359 -1.92870690
H -1.39881385 1.18766923 0.50855233
H 0.78936304 1.53565292 0.24412585
O -1.14575122 2.10451676 0.78688830
C -0.65715940 -2.72480234 -1.41964313
O -1.32832260 -2.61040640 -0.38721083
C -0.43259378 -1.58015270 -2.36200686
C -0.00800040 -4.03653918 -1.74899270
H -0.44989870 -4.84785829 -1.16132010
H 1.05947025 -3.93751868 -1.49221830
H -0.06530659 -4.25464179 -2.82398292
H -0.81440579 -0.63702290 -1.96048823
H -0.93681898 -1.79754440 -3.31674450
H 0.64113289 -1.49007524 -2.57863857
C 0.37413476 -1.18609394 2.07368305
O 0.83957212 -0.65246406 1.06024312
C 0.54430152 -2.65775537 2.33035025
C -0.40108949 -0.38935677 3.07787356
H -0.61146085 0.61967803 2.71109428
H 0.19195498 -0.32812887 4.00510607
H -1.33524415 -0.90863296 3.33517741
H 1.39946991 -3.06299968 1.77840221
H -0.36725101 -3.15803007 1.96332361
H 0.63336255 -2.87425783 3.40245211

h3a2-10.xyz
56
-2757.20927806520
C 4.06786423 -2.36174242 0.60047716
C 4.37443717 -0.35375762 -0.99719806
C 3.88051828 -0.84002219 0.38523117
F 5.33127413 -2.77582884 0.36844691
F 3.76996036 -2.66178192 1.89469622
F 3.23480546 -3.09791619 -0.17876527
F 5.71054086 -0.51616406 -1.13208222
F 3.77084335 -1.01779329 -2.01992302
F 4.09755605 0.95854123 -1.14348838
H 1.98739645 -0.52703839 -0.16938253
H 4.53264665 -0.34946965 1.12607532
O 2.55988706 -0.47591622 0.64924407
C 0.86664094 3.80598190 0.66931901
C -1.51517172 3.16778948 -0.10835233
C -0.02840479 2.76358359 -0.03073486
F 0.45574649 4.04640563 1.94455530
F 2.13394396 3.32512728 0.74259069
F 0.91197993 4.98757090 0.01985046
F -2.05953979 3.36851902 1.11786611
F -1.72867420 4.27529200 -0.84665593
F -2.21415650 2.14920817 -0.69123000
H 0.67868982 1.57590878 -1.39809222
H -0.00307004 1.86140510 0.60370344
O 0.45620443 2.53848269 -1.32678388
C -4.17572636 -1.18564641 -1.27236998
C -3.95372392 -1.88435957 1.20125348
C -3.40956711 -0.99862385 0.05272906
F -5.51245722 -1.07083027 -1.10800214
F -3.79005991 -0.23181262 -2.15787474
F -3.92505409 -2.38856476 -1.84149164
F -5.21749134 -1.55384647 1.55037955
F -3.92714714 -3.20222362 0.90374428
F -3.17084498 -1.70120752 2.30239161
H -1.49000279 -0.79334455 0.46995634
H -3.57771631 0.04786799 0.36283409
O -2.06436987 -1.28398066 -0.19758506
C 0.15951895 -0.66587285 2.35628166
O -0.51607957 -0.03331013 1.53798334
C 0.97717934 0.05677718 3.38646179
C 0.18240661 -2.16690403 2.37315822
H 0.01530657 -2.54237837 3.39300325
H -0.56213782 -2.58697605 1.68989490
H 1.18908072 -2.49596171 2.07525891
H 1.08991221 1.11310138 3.12393822
H 0.45916002 -0.01860957 4.35675121
H 1.95919023 -0.41868129 3.50363306
C 0.21196508 -0.88287696 -2.15589007
O 0.86832703 -0.16810034 -1.38375190
C 0.27965839 -2.37453818 -2.07361065
C -0.66270040 -0.27001022 -3.20380365

H -0.89100931 0.77495329 -2.96927719
H -0.11730046 -0.30853637 -4.16197493
H -1.58530129 -0.84829755 -3.33112628
H 1.13166727 -2.70498288 -1.47190946
H -0.65719313 -2.70817833 -1.59802319
H 0.31322384 -2.82386271 -3.07551001

h3a3-4.xyz
66
-2950.47039612587
C 1.73157619 -3.71829808 1.13968665
C 1.64385347 -3.12103970 -1.36587498
C 1.77153231 -2.58571222 0.08312408
F 2.63895804 -4.69331902 0.89617786
F 2.00808648 -3.20102538 2.36299796
F 0.50824569 -4.30402232 1.21995048
F 2.70777282 -3.88310536 -1.71578624
F 0.52396229 -3.85712521 -1.56279859
F 1.59481226 -2.07716713 -2.23561298
H -0.08776995 -1.87938715 0.22708185
H 2.78193326 -2.15097090 0.14695628
O 0.85795217 -1.58288773 0.37860166
C 2.81209368 3.63574307 0.42024315
C 3.64688746 1.42438709 -0.63068646
C 2.44780591 2.37623975 -0.39364794
F 3.83664383 4.32334998 -0.13738489
F 1.74127844 4.46358939 0.46921525
F 3.15698773 3.33851732 1.69664476
F 4.64471571 2.01093956 -1.33417354
F 4.16911382 0.94602140 0.52094484
F 3.21807698 0.35502618 -1.35596397
H 0.80111967 1.29366428 -0.37934707
H 2.15027318 2.74280790 -1.39306192
O 1.42759492 1.71436441 0.27962397
C -4.45347754 0.39618528 -0.13664758
C -3.00483930 2.48966954 -0.52759117
C -3.17073901 1.16383894 0.24846954
F -4.43613175 -0.01468997 -1.43443823
F -4.55155272 -0.72679904 0.62999019
F -5.58270798 1.10784852 0.05349460
F -3.00582929 2.29699068 -1.87864187
F -3.97008537 3.38919436 -0.24532629
F -1.81445877 3.05423331 -0.20473832
H -2.39450058 0.97815109 2.02775319
H -2.32061415 0.52841309 -0.05837738
O -3.19085450 1.43399399 1.61772519
C -0.06290097 0.90540747 2.90934428
O -1.02114899 0.22336782 2.53115811
C 1.25246623 0.26099931 3.22642381
C -0.16745969 2.39622634 3.06945411
H -1.04790296 2.79218219 2.55282483
H -0.25331333 2.61892037 4.14609036
H 0.74383722 2.88731409 2.70835873
H 1.13760884 -0.81663442 3.37503237
H 1.89934484 0.42836464 2.35055515
H 1.73706367 0.73674593 4.08987534
C -2.44256168 -2.88988047 0.64275565
O -1.67328354 -2.34184191 -0.14904699
C -2.25603616 -2.79801824 2.12952820
C -3.60325231 -3.70172094 0.13196109
H -3.77760327 -3.49844867 -0.92940218
H -3.35697165 -4.76906546 0.25536687
H -4.51304504 -3.51059984 0.71536354
H -1.36654816 -2.21368840 2.38180622
H -3.13976794 -2.30934890 2.56759476
H -2.20158464 -3.80666310 2.56584066
C -0.39046861 0.29676813 -2.54582340
O -0.34876473 0.58600397 -1.35058829
C -1.41929957 -0.66642552 -3.06480641
C 0.56738765 0.89287977 -3.54295812
H 1.05281728 1.78847331 -3.14154995
H 1.33998625 0.14330190 -3.77424484
H 0.05453063 1.13099211 -4.48496670
H -1.76384318 -1.32674773 -2.26161772
H -2.27677382 -0.07959609 -3.43307406
H -1.03167724 -1.24839513 -3.91126098

h3a3-7.xyz
66
-2950.47045405399
C 1.63197691 3.68334559 -1.14704351
C 1.51690587 3.11203356 1.36179359
C 1.67294453 2.56182032 -0.07889804
F 2.50065912 4.68824795 -0.88410928
F 1.96247463 3.16446682 -2.35571035
F 0.39415072 4.23013519 -1.27031891
F 2.58335656 3.86286650 1.72756101
F 0.40342869 3.86619719 1.52530051
F 1.43276219 2.07909488 2.24168473
H -0.17230357 1.82142592 -0.22925447
H 2.68995574 2.14099194 -0.12363816
O 0.77861561 1.54273529 -0.37970533
C -4.49765470 -0.48844195 0.16852225
C -3.02515896 -2.57838013 0.47975873
C -3.22014114 -1.23904266 -0.26649530
F -4.44877786 -0.10274231 1.47308536
F -4.62528594 0.64789054 -0.57341786
F -5.62563087 -1.20615447 -0.00516756
F -2.99642043 -2.41219973 1.83381565
F -3.98965511 -3.48007209 0.20232593
F -1.83820922 -3.12626880 0.11854807
H -2.49874698 -1.01683147 -2.06806990
H -2.36565273 -0.60385478 0.02849372
O -3.27891911 -1.48345139 -1.63930470
C 3.13782355 -3.47824539 -0.40824137
C 3.75056496 -1.18288404 0.61654770
C 2.65756509 -2.26089837 0.41042417
F 4.23397747 -4.05692324 0.13677554
F 2.15472353 -4.41122477 -0.43862592
F 3.43032780 -3.15119507 -1.68817237
F 4.83147460 -1.65737301 1.27930321
F 4.17461552 -0.63990031 -0.54623029
F 3.22897404 -0.17316163 1.36828803
H 0.89601695 -1.35191229 0.41367861
H 2.42467461 -2.65087692 1.41815048
O 1.55813141 -1.71974209 -0.24527769
C -0.15428845 -0.93401339 -2.90750688
O -1.14374076 -0.25409091 -2.61319540
C -0.20258722 -2.43526758 -2.93577035
C 1.15771985 -0.27410211 -3.20854604
H 1.02239572 0.79225876 -3.41116850
H 1.77908070 -0.38516048 -2.30541499
H 1.68356613 -0.77478522 -4.03245996
H -1.20332941 -2.81025103 -2.69902024
H 0.11738045 -2.80072349 -3.92289959
H 0.52025423 -2.81903199 -2.20110026

C -2.54296522 2.81679321 -0.60096841
O -1.76082112 2.25224961 0.16656959
C -3.68930547 3.62509254 -0.05371560
C -2.38713445 2.74815800 -2.09226377
H -1.50493062 2.16590822 -2.37338054
H -3.28107077 2.26794799 -2.51906676
H -2.34006719 3.76324553 -2.51412350
H -3.84583650 3.40214880 1.00644148
H -3.43787887 4.69305811 -0.16045030
H -4.61055490 3.45162093 -0.62460723
C -0.37472470 -0.41954676 2.52022517
O -0.32223551 -0.70134521 1.32331994
C 0.60480357 -0.98914177 3.51252900
C -1.43232938 0.50784307 3.04455905
H -1.80695825 1.15495700 2.24434025
H -2.26508343 -0.10697901 3.42354006
H -1.05626973 1.10483700 3.88584601
H 1.08531285 -1.89181362 3.12004347
H 1.37943710 -0.23095577 3.70718499
H 0.11570055 -1.20847531 4.47115380

h3a3-8.xyz
66
-2950.47229571060
C -4.92572346 0.93039568 0.16249703
C -4.76714577 -1.63114365 0.53131126
C -4.17010923 -0.23720411 0.84447399
F -6.23309045 0.96374711 0.52233089
F -4.36737280 2.10301206 0.54847045
F -4.86399030 0.86842832 -1.18586814
F -6.06624412 -1.73275939 0.89126878
F -4.66810083 -1.95817678 -0.77886669
F -4.07785463 -2.57335223 1.23429456
H -2.60149932 -0.29358918 -0.40869481
H -4.32087730 -0.09074649 1.92674095
O -2.80410837 -0.19592090 0.58344830
C 2.51007531 -3.72023677 0.65501577
C 4.11868361 -1.73440493 0.32868767
C 2.75144044 -2.36245759 -0.03072795
F 2.63513773 -3.63495578 2.00635291
F 1.23389806 -4.13130969 0.40360302
F 3.33792044 -4.69001864 0.22202274
F 4.20968729 -1.43670462 1.65076718
F 5.16746317 -2.52185295 0.00665955
F 4.25930217 -0.56740882 -0.35460986
H 2.14297511 -1.76920478 -1.79341291
H 1.98509709 -1.67700889 0.37399345
O 2.64285326 -2.54829535 -1.41298730
C 2.86131762 3.46353230 0.62179626
C 0.34636379 3.94703819 0.24281400
C 1.41875389 2.91286669 0.66223151
F 2.99506174 4.59879952 1.34899875
F 3.70448021 2.54076499 1.14104702
F 3.27160062 3.72913474 -0.64186103
F 0.29988921 5.00624682 1.08573112
F 0.53929796 4.41865997 -1.01088535
F -0.87401622 3.35147400 0.27139116
H 0.93925095 1.03162624 0.34202325
H 1.21597900 2.68084748 1.72229880
O 1.34724533 1.79348815 -0.16483994
C 1.46380574 0.52861367 -2.80292394
O 1.12087093 -0.56861249 -2.35138049
C 2.90913038 0.90173016 -2.95672904
C 0.43352514 1.53460716 -3.22791117
H 0.41377247 1.57038969 -4.32989232
H -0.55839045 1.26129041 -2.85407760
H 0.71310315 2.53687258 -2.87928570
H 3.55708270 0.02101188 -2.90326493
H 3.07765423 1.45689180 -3.88975812
H 3.16279770 1.57829430 -2.12627522
C -1.62528353 -1.54726493 -2.30076833
O -2.15514541 -0.49128688 -1.94427477
C -1.40102311 -1.82691999 -3.76163720
C -1.22431743 -2.61740458 -1.32840331
H -1.47939893 -2.33976373 -0.30197565
H -1.71466610 -3.56682590 -1.59100387
H -0.14163547 -2.79102135 -1.40602088
H -1.55806052 -0.92081627 -4.35543102
H -0.39478173 -2.23155565 -3.92938669
H -2.12038530 -2.59628391 -4.08606735
C -0.34956959 -0.44757901 2.09736728
O 0.42219897 -0.32051988 1.14330650
C -0.92333568 0.75099919 2.80458774
C -0.70985206 -1.80655518 2.61803468
H -0.11254223 -2.58871472 2.14022455
H -0.58514605 -1.84353591 3.71055634
H -1.77714345 -1.97923074 2.41142033
H -0.18859538 1.08598130 3.55604736
H -1.10420757 1.56453081 2.09432021
H -1.85069874 0.50740564 3.33540212

h3a3-9.xyz
66
-2950.47767999035
C -5.36676012 -0.89170966 -1.22693850
C -4.95250857 -1.39071681 1.28125483
C -4.44417005 -1.55071655 -0.17272786
F -6.61574615 -1.41102117 -1.21093691
F -4.85185325 -1.10473436 -2.46470412
F -5.47137813 0.45067992 -1.05994706
F -6.19785280 -1.89540822 1.44724172
F -4.96353287 -0.10192944 1.68869401
F -4.12350797 -2.07339422 2.11546656
H -3.02850611 -0.13269898 -0.14148479
H -4.48090905 -2.63159209 -0.38305025
O -3.12311378 -1.12828385 -0.30599600
C 1.12583636 3.79156341 1.06466210
C 2.91019291 3.01783324 -0.62687837
C 1.58148674 2.68547321 0.09141656
F 2.06467529 4.07774892 1.99575965
F 0.01248494 3.37573439 1.72774675
F 0.80337693 4.94417352 0.43040002
F 3.93937571 3.16079429 0.24158199
F 2.83305780 4.14343648 -1.37252592
F 3.22741862 1.99614231 -1.46575720
H 0.35921250 1.49879090 -0.90314410
H 1.77131600 1.79327708 0.71309077
O 0.56947159 2.48133964 -0.85424367
C 4.76837501 -1.60153283 -0.03476460
C 3.34407081 -3.74960769 0.21739003
C 3.47889546 -2.24373354 0.52919579
F 5.88870814 -2.19591735 0.44422656
F 4.80649359 -0.29918938 0.33568988
F 4.81666006 -1.64815143 -1.38611630
F 4.39435713 -4.47102010 0.66898237
F 3.20754055 -3.99090097 -1.10752386

F 2.22726565 -4.23007427 0.83168160
H 1.92092284 -1.04516115 0.73741396
H 3.55337758 -2.16298413 1.62777092
O 2.36460831 -1.58615896 0.00850335
C 0.26063545 -0.83780735 -1.86824582
O -0.14897703 -0.05155286 -1.00792031
C 1.30860474 -0.44966256 -2.86915924
C -0.27614225 -2.23916078 -1.92014942
H -0.38324276 -2.59229314 -2.95434947
H -1.22916399 -2.29724325 -1.38369608
H 0.46373680 -2.89090795 -1.42922221
H 1.61312036 0.59397184 -2.74813079
H 0.93720890 -0.62403932 -3.89005014
H 2.18302315 -1.10239107 -2.72770478
C -2.62138014 2.28531889 -0.72003396
O -2.77985315 1.45251075 0.17655292
C -2.51133373 3.74652253 -0.40118165
C -2.53312690 1.88715140 -2.16814978
H -2.55540668 0.79954238 -2.28324811
H -1.60829684 2.29600458 -2.60054019
H -3.37129472 2.33499593 -2.72326504
H -2.66884443 3.92445404 0.66658607
H -3.23639207 4.32076607 -0.99706905
H -1.50775846 4.09198720 -0.68969823
C -0.01422206 -0.50743859 2.13186416
O 1.13528935 -0.15753351 1.82910330
C -0.93472730 0.44488846 2.83172123
C -0.54812041 -1.86675292 1.79618252
H 0.19265601 -2.47187081 1.26493131
H -1.45496704 -1.75335314 1.18063875
H -0.85847190 -2.37720168 2.72048007
H -1.58471104 0.90475579 2.06684578
H -0.36946759 1.24092608 3.32640575
H -1.59400478 -0.07452920 3.53896442

h4a1-1.xyz

58
-3354.17113489647
C -0.24158733 2.97632583 -2.04139913
C -2.70794412 2.79380695 -1.25577860
C -1.41588257 2.04529200 -1.66068295
F -0.57215657 3.84657146 -3.02234481
F 0.79640212 2.23284326 -2.48140356
F 0.18912318 3.69255596 -0.97114830
F -3.14469107 3.63932711 -2.21410510
F -2.53806942 3.50673938 -0.11157704
F -3.69073646 1.88867481 -1.02898108
H -0.67933369 1.62527582 0.12075248
H -1.66096428 1.46433013 -2.56213310
O -1.03975276 1.14068890 -0.66049947
C -3.66127686 -0.40293502 1.77798169
C -3.38982455 -2.57153190 0.42479240
C -2.72863468 -1.25382802 0.89791944
F -4.86821362 -0.21187671 1.20424403
F -3.10151366 0.82602320 1.97008757
F -3.85677622 -0.94194475 3.00133993
F -4.37806717 -2.34437614 -0.47110382
F -3.89581477 -3.30802498 1.43505022
F -2.44785593 -3.33723027 -0.20091533
H -0.82845159 -1.70259385 1.01867287
H -2.52178778 -0.65530823 -0.00655858
O -1.57350008 -1.50985727 1.64802826
C 3.84333946 -0.14651983 1.94734639
C 3.90703813 1.72434810 0.15894085
C 3.42147650 0.30216191 0.52831535
F 5.17831328 -0.30662851 2.04857519
F 3.25852612 -1.33433856 2.23546508
F 3.45163425 0.73824164 2.90399019
F 5.22707158 1.88570505 0.40985125
F 3.22896636 2.67513579 0.84488548
F 3.70411064 1.94051069 -1.15796432
H 1.49778836 0.78410170 0.95495997
H 3.91103647 -0.39041571 -0.17087186
O 2.03772663 0.19472229 0.33787610
C 2.03729805 -3.45426549 -1.13069457
C 0.32775243 -1.98455191 -2.41482277
C 0.65440010 -2.76153269 -1.11675792
F 2.17215113 -4.31186244 -2.16361693
F 2.20163397 -4.15142853 0.01433233
F 3.05290246 -2.54850350 -1.20519275
F 0.37835407 -2.78140275 -3.50612164
F 1.16911671 -0.94497129 -2.61033265
F -0.93109150 -1.48564011 -2.33220251
H 1.18171893 -1.17588141 0.00721758
H -0.09436971 -3.56244021 -1.03733668
O 0.53235353 -1.94314701 0.01248509
C -0.16626294 1.58547114 2.82093114
O 0.30853087 1.65318972 1.67710846
C 0.13873217 0.43612589 3.72601910
C -1.08311011 2.65976960 3.31856600
H -1.44310887 3.28532300 2.49612351
H -0.51027490 3.28640356 4.02303445
H -1.92209987 2.23206924 3.88229755
H 1.11061555 -0.01161471 3.49647736
H -0.63629400 -0.32444236 3.51977404
H 0.07338583 0.71238649 4.78528189

h4a1-3.xyz

58
-3354.17115003153
C -0.31326271 2.98586153 2.03393288
C -2.75474390 2.89538729 1.15821453
C -1.51183256 2.09952153 1.62197720
F -0.64064925 3.87758400 2.99685846
F 0.67700389 2.20361548 2.51529085
F 0.18238821 3.67398167 0.97409923
F -3.18298828 3.78119503 2.08334830
F -2.51403746 3.57535149 0.00597076
F -3.76953021 2.03172154 0.91606770
H -0.75797738 1.62076573 -0.13709011
H -1.81454034 1.54515410 2.52259185
O -1.13701468 1.16261762 0.65151415
C -3.70628938 -0.53239788 -1.70649155
C -3.34157662 -2.65514748 -0.30424264
C -2.72829861 -1.33377317 -0.82881737
F -4.90031455 -0.35312660 -1.10203709
F -3.18342562 0.70280991 -1.95209386
F -3.92442906 -1.11365853 -2.90635880
F -4.31468147 -2.43046225 0.60863579
F -3.84976357 -3.43310691 -1.28162788
F -2.36454232 -3.37753296 0.31974993
H -0.82027317 -1.74197810 -0.98925278
H -2.51509349 -0.70505547 0.05366392
O -1.58627808 -1.57963812 -1.60199434
C 2.13691369 -3.46777136 1.01318849
C 0.48378507 -2.03357455 2.40765041

C	0.75270528	-2.78126096	1.07960181
F	2.33751899	-4.32146418	2.03894986
F	2.23507080	-4.16793516	-0.13780821
F	3.15063286	-2.55769767	1.02359735
F	0.54133291	-2.86498595	3.47243271
F	1.36131070	-1.02444961	2.61088917
F	-0.76038993	-1.49694804	2.37406742
H	1.21111178	-1.16144874	-0.02470306
H	0.00376522	-3.58347883	1.01822924
O	0.57596852	-1.94063829	-0.02619833
C	3.85693036	-0.04180194	-1.90717001
C	3.80805662	1.86285232	-0.15391418
C	3.39380607	0.41339338	-0.50312128
F	5.19989657	-0.12860002	-1.99091825
F	3.34285486	-1.26652989	-2.17475532
F	3.42708730	0.79798266	-2.88736584
F	5.12683854	2.07410601	-0.37217010
F	3.11057054	2.76884825	-0.88027241
F	3.55889880	2.09945156	1.15123548
H	1.45504378	0.80941948	-0.95607478
H	3.90357604	-0.24271920	0.21647314
O	2.01355176	0.24487244	-0.33168341
C	-0.23143941	1.49440821	-2.83332483
O	0.23453390	1.61785856	-1.69045953
C	0.12327490	0.32996007	-3.70017823
C	-1.18805156	2.51377922	-3.36977258
H	-1.57901105	3.14967870	-2.56974936
H	-0.63678656	3.14092225	-4.09076100
H	-2.00507013	2.03420084	-3.92389340
H	1.09932154	-0.08902965	-3.43667939
H	-0.64066130	-0.44206510	-3.49505387
H	0.07708210	0.57833260	-4.76752147

h4a1-4.xyz

58

-3354.17123958760

C	-2.39939769	3.43088375	0.83041291
C	-0.78500268	2.07570640	2.34180973
C	-0.99359991	2.80798119	0.99448699
F	-2.72111049	4.25063603	1.85317123
F	-2.43910911	4.15161809	-0.31164306
F	-3.36614634	2.47563450	0.74160495
F	-0.88973494	2.92408941	3.38958693
F	-1.67588555	1.07187549	2.52072349
F	0.45537281	1.53524493	2.37090716
H	-1.27981987	1.14902152	-0.10783658
H	-0.27844269	3.64248396	0.98139941
O	-0.70615972	1.97554491	-0.09484108
C	-3.80017075	-0.30187754	-1.94452394
C	-3.58908919	-2.07828291	-0.07056864
C	-3.30482017	-0.62369745	-0.51488584
F	-5.14370724	-0.35772234	-2.03818208
F	-3.41123058	0.95241752	-2.28204016
F	-3.27634678	-1.14751085	-2.87202894
F	-4.88808709	-2.41234801	-0.25058861
F	-2.82731958	-2.96580216	-0.75304683
F	-3.30322236	-2.20976571	1.24191568
H	-1.33547236	-0.86859151	-0.95595308
H	-3.87229145	0.02977498	0.16277651
O	-1.94492566	-0.32325427	-0.36037653
C	0.54941225	-2.86962269	2.12525220
C	2.85549540	-3.09815281	0.95645242
C	1.78979061	-2.14633496	1.54873668

F	0.87960121	-3.78398319	3.06624841
F	-0.26893788	-1.96182927	2.70109187
F	-0.15334911	-3.50130219	1.15227781
F	3.28701111	-4.01667696	1.84723420
F	2.38236170	-3.76152562	-0.13363011
F	3.92736250	-2.37568849	0.55606118
H	0.94939122	-1.60560909	-0.15182689
H	2.26889528	-1.62665277	2.39160500
O	1.41340086	-1.17900770	0.60925734
C	3.69730417	0.86129844	-1.61456921
C	3.18105597	2.86807239	-0.09349333
C	2.64206439	1.57316181	-0.74956913
F	4.84118076	0.63577001	-0.93268930
F	3.21291215	-0.35126033	-2.00957022
F	4.00269957	1.54776330	-2.73618404
F	4.10597926	2.60097341	0.85795735
F	3.72073212	3.73184349	-0.97693235
F	2.14705427	3.51411106	0.52200001
H	0.73465569	1.92899710	-1.00581877
H	2.39481639	0.87671640	0.07132811
O	1.54418735	1.84609408	-1.57710689
C	0.37919875	-1.40607541	-2.81465055
O	-0.07120212	-1.59465274	-1.67344593
C	-0.05058113	-0.23741599	-3.64002036
C	1.39065693	-2.34867188	-3.38844331
H	1.84223235	-2.96878058	-2.60821922
H	0.86448827	-3.00267133	-4.10456112
H	2.15907642	-1.80760589	-3.95491425
H	-1.04242946	0.12316160	-3.35046654
H	0.67588407	0.56784341	-3.42482188
H	-0.00874251	-0.45242047	-4.71479079

h4a1-5.xyz

58

-3354.17123168094

C	0.73788971	2.82889455	-2.16547314
C	2.99407071	3.05403666	-0.90369331
C	1.94585033	2.10257094	-1.52705413
F	1.11671968	3.72618687	-3.10469594
F	-0.06471284	1.92046003	-2.76256710
F	0.00110023	3.48112393	-1.23232492
F	3.48031400	3.94629301	-1.79308285
F	2.47811719	3.74854385	0.14728552
F	4.03526184	2.32813856	-0.43513974
H	1.03680564	1.58970040	0.14842667
H	2.45603223	1.56804399	-2.34192613
O	1.52096660	1.15059899	-0.59296293
C	3.64080160	-1.03988172	1.60910201
C	3.03382454	-3.00576979	0.06988850
C	2.54848199	-1.70209296	0.75056793
F	4.77465993	-0.82904750	0.90577262
F	3.19911661	0.17552355	2.04314205
F	3.95278987	-1.76036177	2.70712731
F	3.95599313	-2.75708769	-0.88918556
F	3.55364333	-3.90044589	0.93420517
F	1.97089349	-3.60699501	-0.54143524
H	0.63492332	-2.00915828	1.02858390
H	2.31605849	-0.98602288	-0.05780478
O	1.45311418	-1.94822065	1.59010156
C	-3.78494744	0.54074137	1.97213656
C	-3.45973921	2.19638406	0.00453102
C	-3.27243813	0.75238814	0.52840507
F	-5.11799708	0.71466742	2.06849865

F -3.50107685 -0.72545234 2.36643994
F -3.18426595 1.38084273 2.85702049
F -4.73713357 2.61957786 0.14612925
F -2.65244451 3.07031808 0.65126654
F -3.15004592 2.23898617 -1.30837783
H -1.29031534 0.89529151 0.95916061
H -3.88216900 0.10211145 -0.11490825
O -1.93531876 0.35477592 0.39636588
C -2.57903552 -3.37747975 -0.81327215
C -0.90647609 -2.06519419 -2.29748375
C -1.14778332 -2.81085567 -0.96254903
F -2.93810374 -4.14902049 -1.86105537
F -2.64806377 -4.13250676 0.30513368
F -3.50313311 -2.38480052 -0.69029496
F -1.01908947 -2.89721447 -3.35744199
F -1.77189439 -1.03861248 -2.47337216
F 0.34594191 -1.55316142 -2.30532918
H -1.35887487 -1.15567289 0.15990246
H -0.46781033 -3.67425368 -0.96295503
O -0.82982824 -2.01118581 0.14331998
C 0.43555695 1.39908717 2.79649319
O -0.00316610 1.59429812 1.65162357
C -0.03969356 0.25265391 3.62808182
C 1.47742420 2.30934742 3.36743290
H 1.95121214 2.91057344 2.58557663
H 0.97290681 2.98370013 4.08024458
H 2.22590739 1.74470498 3.93768642
H -1.04636199 -0.06797321 3.34245754
H 0.65160607 -0.58335073 3.41395356
H 0.01367809 0.46993447 4.70174658

h4a1-6.xyz
58
-3354.17122253351
C -0.49364649 2.87142803 -2.13067028
C -2.83747612 3.03079462 -1.02710543
C -1.72258486 2.11131642 -1.57863340
F -0.83047147 3.76578256 -3.08809777
F 0.36942481 1.98740444 -2.67705975
F 0.16227883 3.53515424 -1.14608093
F -3.27806713 3.92178385 -1.94132659
F -2.41921181 3.72417047 0.06643946
F -3.89264755 2.27364625 -0.64698865
H -0.89467598 1.61558519 0.14267728
H -2.15985201 1.56552496 -2.42768998
O -1.33880789 1.16810790 -0.61793843
C -3.68261045 -0.77653675 1.65036132
C -3.22135959 -2.81549512 0.15476476
C -2.65152208 -1.52027512 0.78325617
F -4.83329986 -0.54664494 0.98204899
F -3.17438833 0.43600212 2.01499816
F -3.98073856 -1.43704183 2.78953288
F -4.15707807 -2.54833619 -0.78595994
F -3.76018141 -3.65752847 1.05945540
F -2.20686275 -3.48618381 -0.46713714
H -0.75016889 -1.92083014 1.02033462
H -2.40214677 -0.84092312 -0.05119724
O -1.54806445 -1.79990640 1.60108643
C 2.34106803 -3.47293087 -0.90151547
C 0.72557803 -2.03925261 -2.33707255
C 0.94343212 -2.82127219 -1.01930950
F 2.63292889 -4.25961482 -1.95866016
F 2.38780754 -4.23581142 0.21253188

F 3.32454339 -2.53665598 -0.79406800
F 0.81254995 -2.84849214 -3.41706744
F 1.62053144 -1.03544149 -2.48845748
F -0.51232117 -1.49078367 -2.33253427
H 1.27610873 -1.20626434 0.13393813
H 0.21415466 -3.64364782 -1.02240416
O 0.68917550 -2.02256910 0.10319553
C 3.81178266 0.30770785 2.01136101
C 3.61850570 1.97410253 0.03534863
C 3.32397978 0.54988132 0.56396253
F 5.15368587 0.38200123 2.11325163
F 3.43237215 -0.93199404 2.40955140
F 3.27168333 1.19449179 2.88983939
F 4.92102280 2.30708247 0.18984077
F 2.86737830 2.90628702 0.66780377
F 3.32798393 2.03083181 -1.28155596
H 1.35524113 0.82766444 0.99017768
H 3.88960282 -0.14530507 -0.07267257
O 1.96305340 0.24874712 0.42462688
C -0.34679417 1.45397123 2.82045571
O 0.11254675 1.61207713 1.67826550
C 0.04891607 0.28720689 3.66560718
C -1.33481422 2.43227710 3.37492726
H -1.76078635 3.05707376 2.58408908
H -0.79688298 3.07729849 4.09040565
H -2.12426547 1.91949713 3.93897600
H 1.04039503 -0.09121124 3.39783579
H -0.68437106 -0.50984348 3.44292807
H -0.00941215 0.51277246 4.73730209

h4a2-1.xyz
68
-3547.44490719967
C -3.32789919 -2.20455654 -1.84029315
C -3.75550586 -1.91315252 0.69392045
C -2.70912882 -2.02760332 -0.43846304
F -4.15582428 -3.26980957 -1.90620691
F -2.33391128 -2.40113602 -2.74620560
F -4.02219166 -1.10973546 -2.23718472
F -4.51873739 -3.02716328 0.79421266
F -4.58026077 -0.85414258 0.53224056
F -3.10661211 -1.75662658 1.87548871
H -1.01566233 -1.11072505 -0.07252915
H -2.13601069 -2.94595887 -0.23506677
O -1.90240040 -0.88217869 -0.46224544
C 4.13000002 2.29786728 -1.15504647
C 4.89517546 0.38294398 0.39983942
C 3.70843807 1.13251171 -0.23149185
F 4.76711646 3.27985627 -0.47441377
F 3.00608788 2.84635048 -1.69914207
F 4.92248469 1.91010975 -2.17697446
F 5.69288094 1.22151561 1.10322320
F 5.65659938 -0.25716225 -0.51139023
F 4.42446236 -0.55284689 1.26739012
H 2.02003638 0.53808958 -1.05509685
H 3.14382160 1.57765453 0.60572993
O 2.94735090 0.18866880 -0.93445331
C 1.17914591 -3.17142418 -0.77211291
C 1.79525066 -3.20692246 1.73405467
C 1.51071881 -2.34560880 0.48166644
F 2.18330721 -4.00747593 -1.10171167
F 0.97251639 -2.33674419 -1.82407570
F 0.04940193 -3.91259043 -0.62784578

F 2.87266389 -4.00532665 1.56482890
F 0.74497269 -3.98311446 2.08420338
F 2.05489150 -2.37959380 2.78070802
H 0.80965409 -0.62093380 1.16425100
H 2.43531546 -1.79123884 0.24716020
O 0.43855712 -1.47409284 0.74397132
C -2.41459048 3.74345357 -0.59435443
C -3.09626580 2.53539533 1.56961086
C -2.32316539 2.43883378 0.23943003
F -3.64831338 3.91279325 -1.12583675
F -1.53426689 3.67103466 -1.63173584
F -2.10464135 4.84922158 0.11517111
F -4.35409404 2.99353513 1.38493535
F -2.48047819 3.35463034 2.46062044
F -3.17978406 1.31344575 2.14426104
H -0.57475280 1.67400848 -0.22831860
H -2.81998208 1.64511824 -0.34563715
O -0.99552239 2.13120475 0.54807284
C 0.74457855 1.24035702 2.70393652
O 1.35241447 0.72687347 1.75647076
C 1.04841917 2.64658149 3.12096516
C -0.29978107 0.49060700 3.47437060
H 0.16460819 0.13730579 4.41036321
H -0.67125996 -0.37650668 2.91978552
H -1.13017781 1.14976201 3.75409961
H 2.01427817 2.97695951 2.72434128
H 1.01211046 2.76242587 4.21234569
H 0.25446413 3.28204065 2.69642672
C 0.10250735 0.71539863 -2.69240043
O 0.40278105 0.93533777 -1.50970683
C -1.30399869 0.89055589 -3.17777696
C 1.13157551 0.28122226 -3.69157326
H 2.14425214 0.30207824 -3.27824717
H 1.06535358 0.91689648 -4.58741690
H 0.88984537 -0.74182177 -4.01914496
H -2.01356308 0.70414279 -2.36644175
H -1.52090900 0.23151700 -4.02656425
H -1.42027386 1.93058298 -3.52176557

h4a2-2.xyz
68
-3547.44486145728
C 3.10345555 -2.28172793 1.95833610
C 3.83780079 -1.89778421 -0.49156700
C 2.66017783 -2.04017052 0.49935257
F 3.81776857 -3.42016969 2.09374541
F 2.00312871 -2.39391211 2.74870788
F 3.84735517 -1.25955660 2.44869150
F 4.66650795 -2.96798652 -0.45961655
F 4.57731234 -0.78992654 -0.25243231
F 3.34237402 -1.80080228 -1.74963342
H 1.00392119 -1.09478756 0.02599074
H 2.10552491 -2.94082294 0.19357529
O 1.87422240 -0.88081942 0.45793519
C -4.12740263 2.23152323 1.37367107
C -4.90329455 0.45245716 -0.33024720
C -3.71157419 1.16365099 0.33598400
F -4.80354432 3.25748684 0.80491070
F -2.99852535 2.75281908 1.93414707
F -4.88087946 1.73419914 2.37739395
F -5.74022470 1.33606061 -0.92407866
F -5.62150668 -0.29018532 0.53713026
F -4.44234993 -0.38427526 -1.29864663
H -1.98916933 0.52525552 1.04703519
H -3.17714389 1.69365586 -0.47095771
O -2.91684872 0.17485574 0.93046468
C -1.21618573 -3.17647508 0.54728044
C -1.79275789 -3.04552562 -1.96486917
C -1.52644084 -2.26911513 -0.65457518
F -2.22481021 -4.03294220 0.80256622
F -1.02787924 -2.41439914 1.65463700
F -0.08335320 -3.90803911 0.37197958
F -2.87098678 -3.85421797 -1.86409825
F -0.73625395 -3.79537732 -2.35139489
F -2.04056555 -2.15098685 -2.95846662
H -0.82131976 -0.50278055 -1.19976682
H -2.45349580 -1.72916914 -0.39791200
O -0.44977574 -1.38296126 -0.84189476
C 2.61002256 3.66575972 0.52089234
C 3.11058744 2.39372613 -1.65633708
C 2.38815757 2.36632046 -0.29495322
F 3.87677117 3.75240955 0.99090693
F 1.77795031 3.66168898 1.59941333
F 2.34387722 4.78621500 -0.18434464
F 4.40696983 2.75270591 -1.53028948
F 2.52318191 3.25357473 -2.52901656
F 3.07307137 1.16520475 -2.22180197
H 0.61081616 1.70738060 0.23414431
H 2.84648729 1.54042129 0.27646268
O 1.02921080 2.15840722 -0.54711138
C -0.83543098 1.42587756 -2.66446311
O -1.38838843 0.87855009 -1.70295107
C 0.12877027 0.69275432 -3.54747261
C -1.12434071 2.86075447 -2.98443801
H -2.04854465 3.19554329 -2.50165252
H -1.16219932 3.03554941 -4.06789274
H -0.28305385 3.44996301 -2.58499269
H 0.49924884 -0.22146376 -3.07391063
H 0.96476342 1.34081825 -3.83713205
H -0.40550057 0.41922759 -4.47268129
C -0.05912972 0.68315549 2.67249943
O -0.37318248 0.94268212 1.50145610
C 1.33093077 0.93174010 3.17363682
C -1.05365734 0.12662688 3.64495873
H -2.06766148 0.10425552 3.23518412
H -1.02417211 0.70956593 4.57765696
H -0.74159253 -0.89606061 3.90877122
H 2.05713494 0.81213836 2.36492843
H 1.58345625 0.26611892 4.00689471
H 1.38092315 1.96876555 3.54185324

h5a1-1.xyz
70
-4144.40025239672
C 2.14611677 -1.89918407 2.86277977
C -0.43320604 -1.96216050 2.74476826
C 0.85932482 -1.21559745 2.33808109
F 2.21599319 -1.86697457 4.21113637
F 3.23484179 -1.23800079 2.38757163
F 2.24368622 -3.18693789 2.46048518
F -0.43811607 -2.32978036 4.04403305
F -0.59813340 -3.07924150 1.99021647
F -1.50149886 -1.16344303 2.53649919
H 0.77164571 -1.86777864 0.47985789
H 0.82083594 -0.22253275 2.80773575
O 0.90359802 -1.01753221 0.94893592

C 4.90267388 0.17242709 -1.33795701
C 3.95282004 -2.19007198 -0.97685747
C 3.68246506 -0.67254085 -0.89821306
F 5.99306235 -0.06523213 -0.57708182
F 4.59619560 1.49810822 -1.19571690
F 5.23470561 -0.02036570 -2.62963731
F 4.97733440 -2.55970438 -0.18027175
F 4.23376978 -2.60190874 -2.23703492
F 2.85020651 -2.87185976 -0.56963451
H 2.00071808 0.28760538 -1.23895020
H 3.50293635 -0.44850997 0.16461217
O 2.57648846 -0.37345061 -1.70624467
C 2.01808953 2.92188210 1.12871994
C 1.09400116 4.08468848 -0.99477672
C 1.75828068 2.82369938 -0.39145300
F 2.72337298 4.02215032 1.45817465
F 2.72540883 1.83663901 1.53273413
F 0.86238430 2.93259083 1.84313741
F 1.90771536 5.15914921 -0.90420866
F -0.07877442 4.40289641 -0.38109814
F 0.82631784 3.87115755 -2.29929317
H 0.17526755 1.58694756 -0.22277217
H 2.74616720 2.75474756 -0.86783303
O 1.05228111 1.65561291 -0.70945565
C -3.11117183 2.00882373 1.95526489
C -3.11941519 2.71000608 -0.53102239
C -2.26169642 2.34798029 0.70870782
F -4.05182784 2.95164808 2.19069952
F -2.30958049 1.93883783 3.04130753
F -3.73690821 0.81287527 1.82867579
F -3.68522338 3.92785826 -0.39130553
F -4.11743283 1.81317202 -0.75510697
F -2.34221687 2.73273261 -1.63567140
H -1.79805520 0.47794259 0.13376846
H -1.69102185 3.25224187 0.95875061
O -1.34296849 1.32115877 0.44851250
C -3.82599246 -2.75518486 -0.15337291
C -3.81591893 -1.44221753 -2.38443471
C -3.56916513 -1.39910994 -0.85641504
F -5.03256757 -3.27270087 -0.48450408
F -3.80794990 -2.57558383 1.18546823
F -2.87735439 -3.66812145 -0.46302448
F -5.11211476 -1.67642765 -2.67674412
F -3.06712931 -2.39589756 -3.00047144
F -3.47861186 -0.25108596 -2.93421143
H -1.53019572 -1.49354849 -0.92436424
H -4.30460695 -0.69179040 -0.44813139
O -2.28967866 -0.91702583 -0.55478646
C 0.18425212 -1.99383602 -2.58491073
O -0.27077211 -2.26486195 -1.46476241
C -0.15757906 -0.71738153 -3.29722745
C 1.07933448 -2.96203538 -3.29393248
H 1.27649727 -3.83935491 -2.67176791
H 2.02211802 -2.46806915 -3.56464701
H 0.59754691 -3.27055508 -4.23515663
H -0.74803123 -0.04402664 -2.66902607
H -0.72381733 -0.94871770 -4.21209095
H 0.77043926 -0.21940012 -3.61016167

h5a1-3.xyz
70
-4144.39991732466
C 2.14654269 -1.92824892 2.88344934
C -0.40570323 -1.51003551 2.98314304
C 0.96010935 -1.04605262 2.42698118
F 2.29131548 -1.93602481 4.22619861
F 3.29884682 -1.43597703 2.35570567
F 2.02050153 -3.20867360 2.46254795
F -0.39480804 -1.65993163 4.32614722
F -0.78425296 -2.68935685 2.42947908
F -1.34832110 -0.59112213 2.67799519
H 0.64565375 -1.78425359 0.62422901
H 1.14452612 -0.04135856 2.83302087
O 0.92046440 -0.93208439 1.02964911
C 4.83252082 0.13847067 -1.51175078
C 3.86950167 -2.20781349 -1.11801688
C 3.62087032 -0.68990780 -1.01303393
F 5.90032127 0.01372938 -0.69182697
F 4.49232790 1.46176302 -1.52657110
F 5.21698528 -0.18666591 -2.76229579
F 5.04038170 -2.57494102 -0.55707539
F 3.86768925 -2.63784112 -2.40390312
F 2.87870799 -2.87852716 -0.47350676
H 1.97216573 0.33639277 -1.31015312
H 3.50216377 -0.46862149 0.06016898
O 2.47251660 -0.39589054 -1.75691756
C 2.05126829 2.88206632 1.22942533
C 1.29317838 4.19928357 -0.86905784
C 1.87019704 2.87816448 -0.30501547
F 2.73938675 3.95636467 1.66569882
F 2.74346997 1.77080628 1.59728645
F 0.86319964 2.84726604 1.88232817
F 2.15013330 5.22706489 -0.68409972
F 0.10850692 4.54244127 -0.28754986
F 1.07386994 4.06942549 -2.19175442
H 0.22724665 1.70952660 -0.30611347
H 2.87877202 2.79405934 -0.73237561
O 1.13845629 1.76027824 -0.72492909
C -3.29332390 2.30078169 1.44523198
C -2.97544084 2.76578201 -1.08199833
C -2.29343022 2.52132199 0.28698051
F -4.24000877 3.26560002 1.48122320
F -2.63195577 2.32093640 2.62178742
F -3.92195449 1.10145545 1.34773342
F -3.60787488 3.95824009 -1.11126564
F -3.88806150 1.80403761 -1.38764653
F -2.04254720 2.76310458 -2.05901411
H -1.78011698 0.60288046 -0.02292235
H -1.74953901 3.44533894 0.52324756
O -1.35742800 1.47786055 0.23553419
C -3.88013020 -2.50542752 0.21733317
C -3.75943051 -1.80698954 -2.27246700
C -3.54328559 -1.38575738 -0.79828461
F -5.12642663 -2.99671778 0.01952579
F -3.82582333 -2.00660594 1.47038173
F -3.00643250 -3.53604364 0.14320857
F -5.05785454 -2.06161363 -2.53560430
F -3.04098904 -2.91392555 -2.60521597
F -3.35879050 -0.80800694 -3.09380188
H -1.52146402 -1.57899356 -0.73006813
H -4.25482129 -0.57040243 -0.60799195
O -2.25027795 -0.88369073 -0.59602742
C 0.11357392 -2.91742998 -2.02806583
O -0.32003192 -2.61935143 -0.90524648
C 0.01706176 -1.97738195 -3.18752951
C 0.76270468 -4.24806967 -2.25464434
H 0.98758997 -4.74172004 -1.30487827

H	1.66761623	-4.14787615	-2.86803348
H	0.05452476	-4.87040363	-2.82696274
H	-0.71219370	-1.18130738	-3.00758740
H	-0.20591155	-2.50778358	-4.12227672
H	1.01371657	-1.51525554	-3.29382465

[1]Redlich, O.; Kister, A. T. *Ind. Eng. Chem. Res.* **1948,** *40,* 345-348.
